# Synthesis and hyperpolarisation of eNOS substrates for quantification of NO production by ^1^H NMR spectroscopy

**DOI:** 10.1016/j.bmc.2017.03.041

**Published:** 2017-05-15

**Authors:** Fernando Fernandez Diaz-Rullo, Francesco Zamberlan, Ryan E. Mewis, Marianna Fekete, Lionel Broche, Lesley A. Cheyne, Sergio Dall'Angelo, Simon B. Duckett, Dana Dawson, Matteo Zanda

**Affiliations:** a Centre for Therapeutics and School of Medicine, Medical Sciences and Nutrition, University of Aberdeen, Foresterhill, Aberdeen, Scotland AB25 2ZD, United Kingdom; b Centre for Hyperpolarisation in Magnetic Resonance, University of York, Heslington, York YO10 5NY, United Kingdom; c C.N.R.- I.C.R.M., via Mancinelli 7, 20131 Milan, Italy

**Keywords:** SABRE, Hyperpolarization, MRI, Real-time imaging, l-Arginine

## Abstract

Hyperpolarization enhances the intensity of the NMR signals of a molecule, whose *in vivo* metabolic fate can be monitored by MRI with higher sensitivity. SABRE is a hyperpolarization technique that could potentially be used to image nitric oxide (NO) production *in vivo*. This would be very important, because NO dysregulation is involved in several pathologies, including cardiovascular ones. The nitric oxide synthase (NOS) pathway leads to NO production via conversion of l-arginine into l-citrulline. NO is a free radical gas with a short half-life *in vivo* (≈5 s), therefore direct NO quantification is challenging. An indirect method – based on quantifying conversion of an l-Arg- to l-Cit-derivative by ^1^H NMR spectroscopy – is herein proposed. A small library of pyridyl containing l-Arg derivatives was designed and synthesised. *In vitro* tests showed that compounds **4a**–**j** and **11a**–**c** were better or equivalent substrates for the eNOS enzyme (NO_2_^−^ production = 19–46 μM) than native l-Arg (NO_2_^−^ production = 25 μM). Enzymatic conversion of l-Arg to l-Cit derivatives could be monitored by ^1^H NMR. The maximum hyperpolarization achieved by SABRE reached 870-fold NMR signal enhancement, which opens up exciting future perspectives of using these molecules as hyperpolarized MRI tracers *in vivo*.

## Graphical abstract

**Figure:**
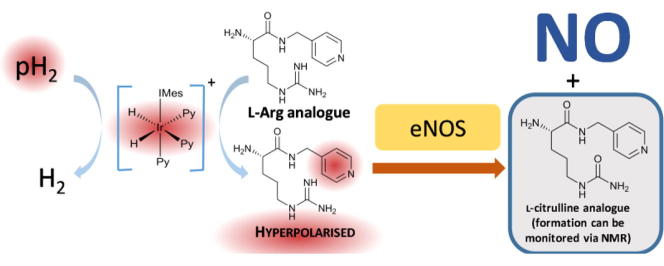

## 1 Introduction

Nuclear magnetic resonance (NMR) is the most powerful technique used for identifying and characterizing organic molecules.^1^ Unfortunately, NMR is inherently affected by a lack of sensitivity.^2^ Magnetic resonance imaging (MRI) is a widely used clinical imaging technique that shares the same principles of NMR, and presents the same inherent problem of low sensitivity, which limits its application in many fields.^3^ Recently, several hyperpolarization techniques have been developed to overcome the sensitivity issue of NMR and MRI, allowing detection of trace amounts of a certain compound in a complex mixture,^4^, ^5^ detection of a metabolite in cells^6^ or real-time imaging in small rodents.^7^ The most used hyperpolarisation techniques are dynamic nuclear polarization (DNP),^8^, ^9^ spin-exchange optical pumping (SEOP), metastability exchange optical pumping (MEOP),^10^ parahydrogen induced polarization (PHIP)^11^ and spontaneous amplification by reversible exchange (SABRE),^11^, ^12^, ^13^ which recently emerged as a promising modality for *in vivo* pre-clinical and clinical MRI.^14^, ^15^

**Fig. 1:**
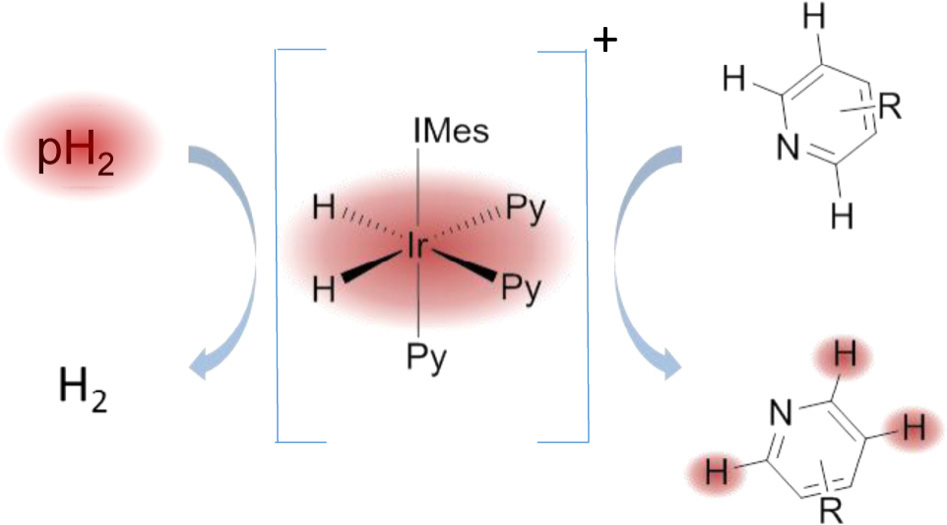
In the SABRE process, the iridium catalyst mediates polarization transfer from *para*-hydrogen to the pyridyl substrate. This process repeats allowing the creation of a solution of hyperpolarised pyridyl substrate.

SABRE exploits the singlet spin state of parahydrogen (pH_2_) – one of the spin states of the H_2_ molecule – in order to increase the NMR signal. Compared to other hyperpolarization techniques, SABRE has the remarkable advantages that the substrate to hyperpolarize remains structurally unchanged and it is relatively non-expensive. Polarization transfer occurs through an iridium(I) complex that acts as a catalyst. Generally the catalytic system contains a carbene group that aids the process (Fig. 1).^16^, ^17^ SABRE has been shown to be effective in the hyperpolarisation of various spin ± ½ nuclei such as ^1^H,^18^ ^15^N^19^, ^20^, ^21^, ^22^ or ^31^P.^23^ Biologically relevant molecules have been hyperpolarised,^24^, ^25^ the catalyst can be deactivated^26^ and hyperpolarisation in aqueous and biologically compatible media has also been achieved.^27^, ^28^, ^29^

**Scheme 1:**
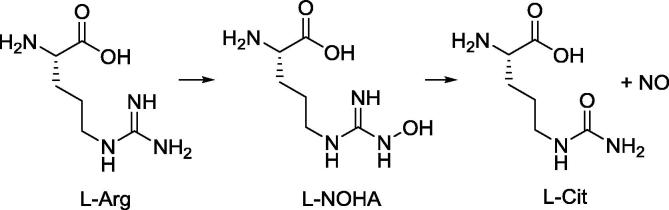
Reaction catalysed by NOS enzymes leading to NO production.

Nitric oxide synthases (NOS) are a family of enzymes that catalyse the synthesis of nitric oxide (NO) from l-arginine through an N^ω^-hydroxy-l-arginine (l-NOHA) intermediate, stoichiometrically producing l-citrulline, a reaction co-product (Scheme 1).^30^ Three NOS isoforms are known: neuronal (nNOS, NOS-1), inducible (iNOS, NOS-2) and endothelial (eNOS, NOS-3).^31^ nNOS and eNOS are constitutive isoforms and Ca^2+^ dependent, while iNOS is Ca^2+^-insensitive and activated by pro-inflammatory cytokines in certain situations of stress or disease as a response of the immune system.^32^ NO is a free radical molecule involved in many functions, such as vascular tone and blood pressure regulation,^33^ blood flow in the kidney,^34^ penile and clitoral erection,^35^ immune response^36^ and neuronal transmission.^37^ It has been reported that abnormal NO formation or NO dysregulation play an important role in certain conditions such as type 2 diabetes,^38^ heart failure,^39^ haemolytic disorders^40^ or critical illness myopathy.^41^ Therefore, detection of NO production *in vivo* would be highly beneficial. Direct *in vivo* detection of NO is challenging because of its very short biological half-life (a few seconds). Moreover, current methods for detecting and quantifying NO *in vivo* are affected by significant drawbacks.^42^ Conversion of l-arginine or l-NOHA to l-citrulline derivatives could be indirectly but effectively tracked by the shifting of the hyperpolarized NMR signal from the enzymatic substrate (l-arginine or l-NOHA) to the product l-citrulline. However, preliminary experiments showed that native l-arginine cannot be efficiently hyperpolarised via SABRE because – unlike pyridine and its derivatives – it is not a sufficiently good ligand for the iridium(I) catalyst.

Herein we report on the effective hyperpolarization of l-arginine-type pyridyl-amide substrates of endothelial nitric oxide synthase (eNOS). These molecular probes can be used to detect *in vitro* the activity of eNOS by ^1^H NMR spectroscopy and might find future use as MRI probes in *in vivo* studies.

## 2 Results and discussion

### 2.1 Design of l-arginine derivatives as eNOS substrates

It has been previously demonstrated that the guanidine moiety of eNOS substrates cannot be modified without causing a dramatic loss of efficiency in the enzymatic conversion to urea and consequent release of NO. In fact, small structural modifications of the l-arginine side chain, such as guanidine methylation or replacement of the δ-methylene with oxygen or carbonyl function afforded substrates with significantly lower affinity or even converted them into potent inhibitors of the NOS enzyme.^43^, ^44^, ^45^, ^46^, ^47^, ^48^

**Scheme 2:**
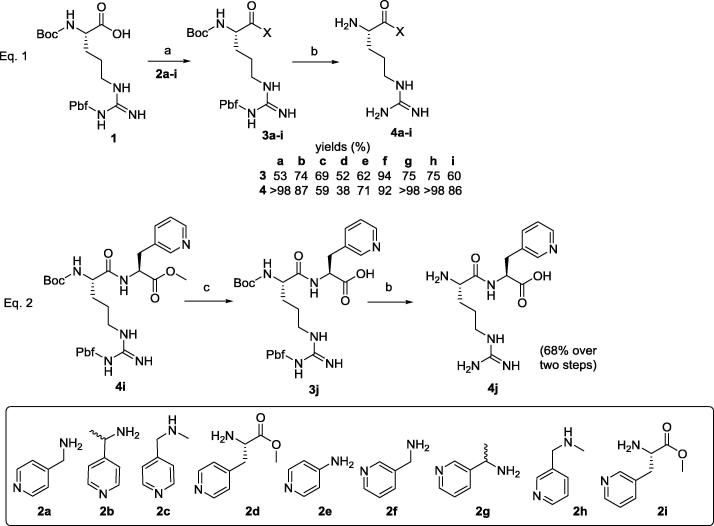
Synthesis of first generation compounds **4a**–**j**. Reagents and conditions: (a) HATU, TEA, CH_2_Cl_2_, r.t., 5 h.; (b) TFA/CH_2_Cl_2_ 95:5, r.t., 2 h.; (c) LiOH, THF/H_2_O 4:1, r.t., overnight.

On the other hand, Grant et al.^47^ reported that the α-amino moiety is involved in the binding through the formation of a hydrogen bond in the active pocket of NOS. We therefore decided to focus on the l-arginine carboxylic function as a plausible site for the introduction of the iridium(I)-binding pyridine group, by installing a pyridyl-amide on l-arginine analogues. Diverse pyridyl amines differently substituted and equipped with a spacer were selected (structures **2a**–**i**, Scheme 2), since the length of the spacer and the pyridine substitution was expected to affect the level of polarization transfer by SABRE.

In addition, considering that l-NOHA activity is similar or higher than that of l-arginine,^46^, ^48^ we decided to explore also l-NOHA analogues incorporating a pyridine ring, such as **11a** (Scheme 4). Since it has been shown that neither the carboxylic nor the α-amino functions of l-NOHA are required for enzymatic recognition – in fact, some *N*-substituted alkyl hydroxyguanidines displayed comparable activity to that of native l-arginine (i.e. *N*-butylhydroxyguanidine)^49^, ^50^ – we included in our study also l-NOHA derivatives lacking the α-amino function (such as **11b**, Scheme 4) or having a protected α-amino group (**11c**, Scheme 4).

### 2.2 Synthesis of l-arginine derivatives

**Scheme 3:**
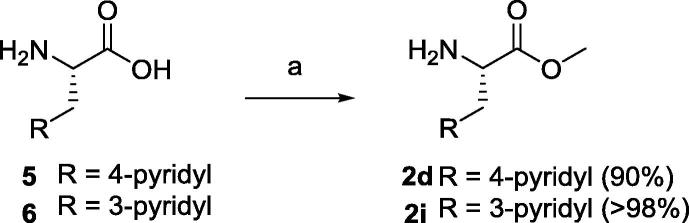
Formation of **2d** and **2i**. Reagents and conditions: (a) SOCl_2_, MeOH, r.t., overnight.

Compounds **4a**–**i** were synthesised starting from commercially available Boc-l-Arg(Pbf)-OH **1** (Scheme 2, Eq. 1), that was coupled with the corresponding amino pyridines **2a**–**i** with HATU as the coupling agent to afford the protected amides **3a**–**i** in good yields. Pbf and Boc protecting groups were cleaved from **3a**–**i** in a TFA/CH_2_Cl_2_ 95:5 mixture to yield the final free compounds **4a**–**i**. Purification of **4a**–**i** was performed on a Sep-Pak® C-18 cartridge using water as the eluent. The TFA counter-ion was exchanged to hydrochloride by dissolving **4a**–**i** in diluted HCl and freeze-drying as many times as necessary. In order to obtain **4d** and **4i**, commercially available 3-(4-pyridyl)alanine **5** and 3-(3-pyridyl)alanine **6** respectively (Scheme 3) were dissolved in methanol and treated with SOCl_2_ to afford the methyl ester derivatives **2d** and **2i**, which were coupled to **1** as previously described. Compound **4j** required deprotection of the methyl ester from **4i** (Scheme 2, Eq. 2), using LiOH overnight to yield **3j**, which was converted into **4j** as described above.

**Scheme 4:**
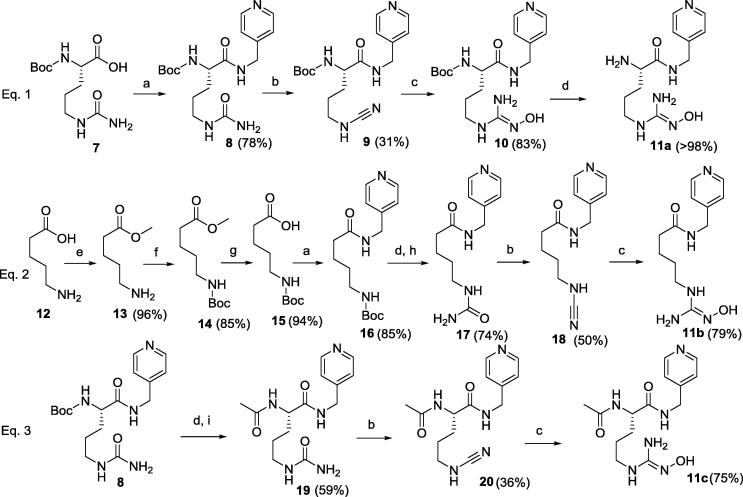
Synthesis of second generation compounds **11a**–**c**. Reagents and conditions: (a) 4-picolylamine, HATU, TEA, DCM, r.t., 5 h.; (b) CH_3_SO_2_Cl, Py, 40 °C, 2 h; (c) NH_2_OH·HCl, K_2_CO_3_, EtOH, r.t., 1 h.; (d) HCl 4 M in dioxane, r.t., 1 h.; (e) SOCl_2_, MeOH, r.t., overnight; (f) Boc_2_O, TEA, DCM, r.t., 1.5 h.; (g) LiOH, THF/H_2_O 4:1, r.t., overnight; (h) KOCN, HCl, 70 °C, 1 h.; (i) Ac_2_O, NaOH, H_2_O, r.t., 3 h.

Synthesis of the second generation of compounds, e.g. l-NOHA analogues **11a**–**c**, is shown in Scheme 4. Compound **11a** was prepared by starting from the commercially available amino acid Boc-Cit-OH **7** which was coupled to 4-picolylamine to afford **8** (Scheme 4, Eq. 1). Compound **11b** (Scheme 4, Eq. 2) was prepared from 5-aminovaleric acid **12**, whose acid function was protected as methyl ester **13**, followed by *N*-Boc function formation **14**. The methyl ester was then hydrolysed to yield **15**, which was coupled to 4-picolylamine to give **16**. After acidic *N*-Boc removal, the *N*-substituted urea **17** was prepared using potassium cyanate in aqueous HCl. Finally, **11c** (Scheme 4, Eq. 3) was synthesised from **8** by deprotecting the *N*-Boc group in acidic conditions and treating it with acetic anhydride in basic aqueous solution to yield urea **19**.

The three urea derivatives **8**, **17** and **19** were treated with CH_3_SO_2_Cl in pyridine at 40 °C to afford the *N*-cyanamides **9**, **18** and **20** (Scheme 4), which were purified by flash chromatography, when possible. These *N*-cyanamides were poorly stable, to the extent that **10** was used as a crude, without purification or characterisation. l-NOHA derivatives **10** and **11b**–**c** were prepared by nucleophilic addition of hydroxylamine to **9**, **18** and **20** in good yields and purified by reverse phase HPLC. The Boc protecting group in **10** was cleaved in dioxane with 4 M HCl (quantitative yield) to afford **11a**. **11b**–**c** were obtained as TFA salts after HPLC purification, then TFA was exchanged to hydrochloride following the procedure described above.

### 2.3 Synthesis of l-citrulline derivatives

**Scheme 5:**
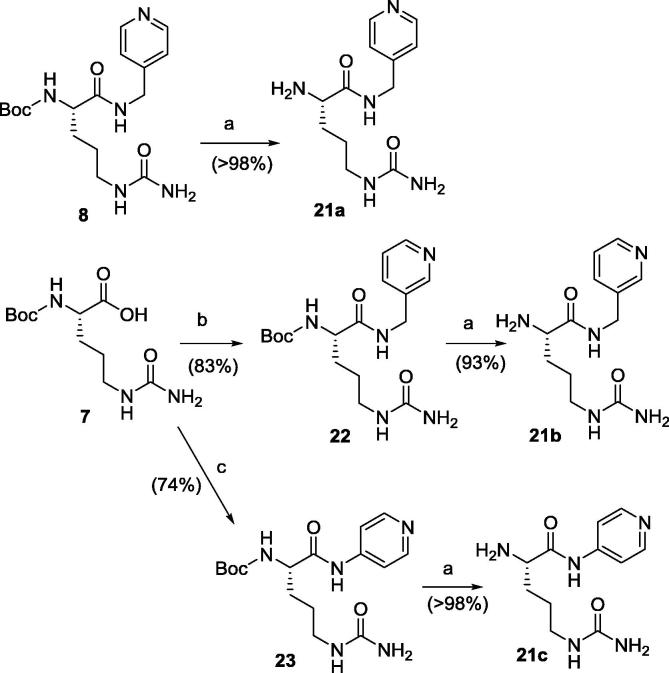
Synthesis of l-citrulline derivatives **21a**–**c**. Reagents and conditions: (a) HCl 4 M in dioxane, r.t., 1 h; (b) 3-picolylamine, HATU, TEA, DCM, r.t., 5 h.; (c) 4-aminopyridine, HATU, TEA, DCM, r.t., 5 h.

The three l-citrulline derivatives **21a**–**c** (Scheme 5) were synthesised by coupling commercially available Boc-l-Cit-OH **7** and the corresponding pyridine function, namely 4-picolylamine, 3-picolylamine or 4-aminopyridine, using HATU to yield **8** (described above), **22** and **23**, respectively, in good yields. The Boc protecting group was cleaved by reaction with 4 M HCl in dioxane to afford l-citrulline derivatives **21a**–**c**.

### 2.4 Enzymatic activity assays

NO production experiments were performed using (bovine recombinant) eNOS enzyme. NO Production was measured by the well-established nitrate/nitrite – also called lactate dehydrogenase (LDH) – colorimetric assay, which exploits the Griess reaction to quantify the nitrites generated from NO.^51^ The concentrations of NO-derived nitrites produced from **4a**–**j** and **11a**–**c** were higher or comparable to that of l-arginine when incubated in the presence of eNOS (Table 1). l-Arginine produced a 25 μM nitrites concentration, while for **4a**, **4g**, **4j** and **11a**–**c** higher concentrations of nitrites were measured. The remaining compounds displayed NO production similar to l-arginine. Dipeptide derivatives **4d** and **4i** were not tested for enzymatic activity because – as explained below – they did not polarise successfully.

### 2.5 SABRE hyperpolarisation

Hyperpolarization experiments were performed on **4a**–**j** and **11a**–**c**. All experiments were done in 0.6 mL of methanol-*d*_4_ in Young tubes previously degassed and filled with pH_2_ with a pressure of 3 bar.^1^, ^15^ The catalyst precursor used was [Ir(COD)(IMes)Cl] [COD = cyclooctadiene; IMes = 1,3-bis(2,4,6-trimethylphenyl)imidazole-2-ylidene], since it was previously demonstrated that this catalyst is one of the most versatile precursors for polarization transfer.^16^ We used the so called “shake-and-drop” method, according to which the sample was shaken for 10 s in a given magnetic field prior to dropping it into the NMR magnet and rapidly acquiring the NMR spectrum.^15^ For these experiments a single 90° radiofrequency (RF) pulse was applied. All of the samples contained a concentration of 40 mM of the ligand **4a**–**j** or **11a**–**c** and 5 mM of the catalyst precursor. Polarization transfer was performed at both 65 G and Earth’s magnetic field (MF) in order to study the effect of MF on the polarization transfer onto the ligands. These MF were chosen based on literature reports.^12^, ^18^, ^24^

Polarization transfer is maximised when the externally applied MF allows the couplings *J* (between the hydrides and the accepting protons) and the chemical shift difference between them to become optimally aligned. The effect of a co-ligand was also studied. In this context, a co-ligand is a small molecule that has lower binding affinity for the iridium catalyst centre than that of the ligand to be hyperpolarized. When the residence time on iridium is too long, the co-ligand can help driving dissociation of the already hyperpolarized ligand, thereby enhancing the build-up rate of this hyperpolarised agent in solution. In a second approach, the co-ligand can enable the hyperpolarization of more bulky targets which might not initially bind due to steric hindrance. The co-ligand, being a small molecule, helps freeing up space on the iridium centre to enable the bulky target to form an active catalyst. Acetonitrile generally behaves as a good co-ligand for SABRE, and in these experiments 2 μL of acetonitrile-*d*_3_ was used (final concentration of 60 mM) to avoid polarization being lost into acetonitrile protons during catalysis.

**Fig. 2:**
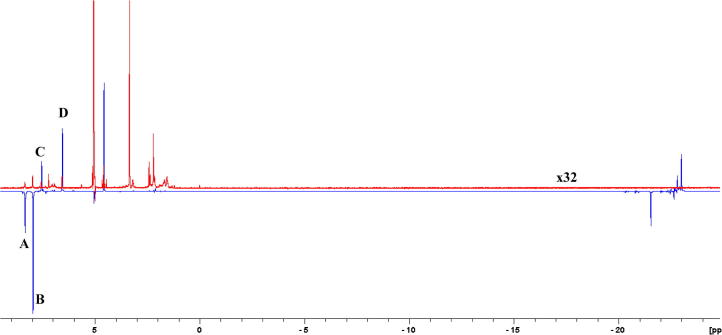
Hyperpolarized ^1^H spectrum for **4e** (in blue) compared to its thermal ^1^H spectrum (in red). Thermal spectrum is vertically enlarged by 32 times. Four hyperpolarized signals can be observed: **A** and **C** correspond to the free compound in solution, while **B** and **D** are those of the fraction bound to the catalyst.

The signal enhancements obtained during this study can be seen in Table 2. Generally, the 4-substituted pyridines hyperpolarized better in the Earth’s MF, while the 3-substituted pyridines showed better performance at 65 G. As expected, when hyperpolarizing with acetonitrile-*d*_3_ the results followed the trend that bulkier molecules polarize slightly better in the presence of acetonitrile-*d*_3_. Conversely, **4d** and **4i**–**j**, the dipeptides derivatives, did not show any significant enhancement of signal, possibly due to steric hindrance even when using a co-ligand. The second generation of compounds also failed to show good performance under these SABRE conditions as only **11b** was hyperpolarized, while **11a** and **11c** failed to hyperpolarize significantly. We believe that the hydroxyl-guanidyl group, despite being less basic than the guanidyl, may bind the metal in an even stronger manner, partially inactivating the catalyst. Compound **4e** was therefore the best performing compound in the SABRE experiment, giving the highest enhancements (Fig. 2). The hyperpolarized ^1^H NMR spectrum (in blue) can be compared to its thermal (in red), where thermal spectrum is vertically enlarged 32 times.

Two enhanced signals were expected, but four were actually observed in the spectrum. Signals labelled as **A** and **C** on Fig. 2 correspond to the free compound **4e** in solution, while signals **B** and **D** correspond to the fraction bound to the catalyst. The bound fraction may readily be dissociated by using a catalyst deactivator, such as bipyridine.^26^ Deactivation of the catalyst may be achieved by adding a chelator to the solution, which is a molecule with a high affinity for the iridium centre. The total enhancement for all four observed enhanced signals was 870-fold, which would correspond to only two peaks if the catalyst were deactivated. This corresponds to a 2.8% achieved polarization at 9.4 T. Hydride signals can be observed in the *δ* −21 to −24 region, indicating that as proposed pH_2_ effectively enters the catalytic complex and is coupled to the pyridine of the ligand to hyperpolarise.

### 2.6 In vitro spectroscopy

**Fig. 3:**
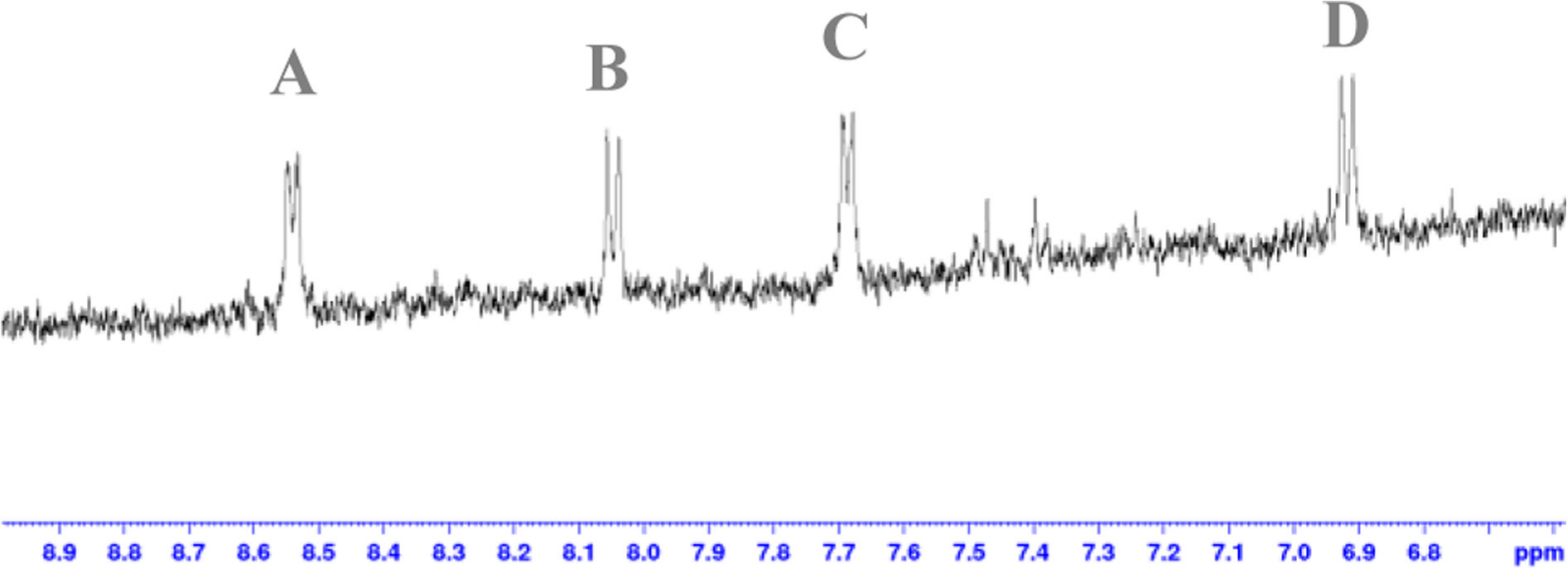
Analogue **4e** was unstable during *in vitro* spectroscopy test, as the amide bond was cleaved producing l-arginine and 4-aminopyridine. Signals **A** and **C** belong to compound **4e**, while **B** and **D** correspond to 4-aminopyridine.

The first *in vitro* spectroscopy attempt was performed using **4e**, *i.e.* the compound that showed the highest level of hyperpolarisation. The experiment was carried out under conditions used for an optimal performance of the eNOS enzyme. The reaction was monitored by NMR. The l-citrulline derivative **21c** was expected to give similar, yet distinguishable, peaks to those of **4e** with the maximum difference being 0.05 ppm in the aromatic region. Fig. 3 shows spectroscopically the fate of analogue **4e** after 3 h of reaction. Signals **A** and **C** correspond to **4e** and after 25 min new signals **B** and **D** started to form, with a 41% conversion after 3 h. However, HPLC analysis of the crude enzymatic mixture revealed that l-arginine and 4-aminopyridine were the main components of the mixture. Further analyses showed that peaks **B** and **D** belong to 4- aminopyridine, as confirmed independently by recording the spectrum of 4-aminopyridine alone (not shown), and that – to our surprise – compound **4e** was simply being hydrolysed by the PBS buffer solution, even in the absence of eNOS.

Analogues **4a** and **4f** also gave good levels of hyperpolarisation (268 and 270-fold enhancement respectively), therefore investigation of their suitability for NMR spectroscopy was carried out next. Both **4a** and **4f** proved to be stable under the reaction conditions overnight. However, when PBS buffered D_2_O solutions of a 1:1 mixture of **4a** or **4f** and their corresponding citrulline derivatives **21a** and **21b** respectively were analysed by ^1^H NMR spectroscopy at 400 MHz we could not discriminate between enzymatic product and starting material via peaks in the aromatic peaks region which would receive SABRE hyperpolarisation.

**Fig. 4:**
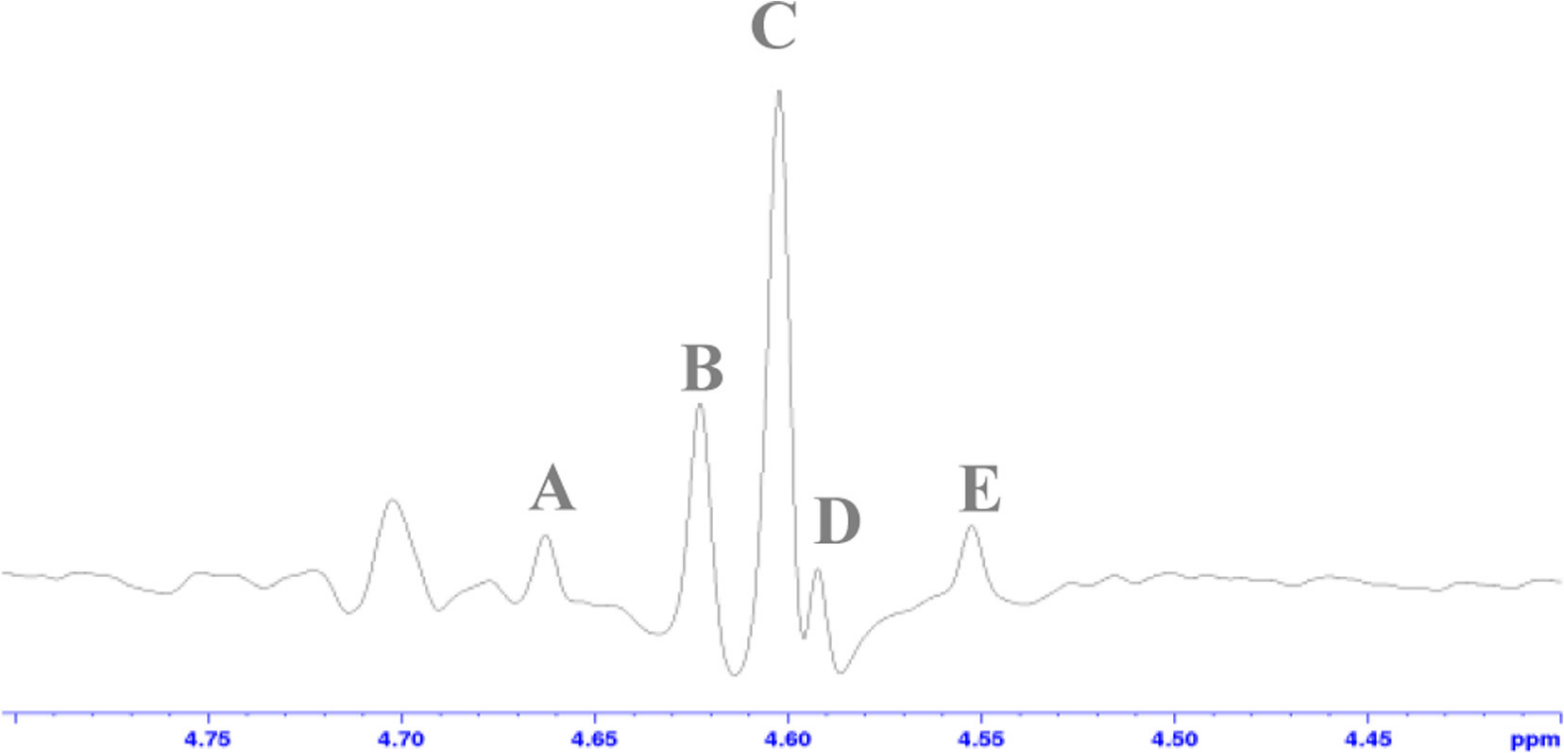
1D COSY corresponding to the benzylic methylene of a 1:1 mixture of **4f** and the corresponding citrulline derivative **21b** when overlapping aromatic signals at *δ* 8.70 are irradiated. Signals **A**, **B**, **D** and **E** correspond to **4f**, whilst signal **C** belong to **21b**. The broad signal at *δ* 4.70 is a solvent artefact.

Gratifyingly, when a 2D COSY or NOESY spectrum of a 1:1 mixture of **4f** and the enzymatic product **21b** was recorded, the amino acid back-bone signals can be distinguished as they exhibit chemical shift differences of 48 Hz and 46 Hz at a field of 9.4 T, while the corresponding benzylic protons appear as a singlet at *δ* 4.60 and an AB doublet at *δ* 4.57 and 4.68 respectively (Fig. 4). Hence, selective excitation of the overlapping signals at *δ* 8.70 for the H-5 ring protons of the pyridyl unit of **4f** in conjunction with 1D COSY or NOE methods enabled the observation of a resolved connection to the distinguished benzylic protons. It is worth noting that similar 1D COSY methods have been used by Tessari et al. to establish the magnitude of hydride substrate couplings in the catalyst,^52^ whilst use of the NOE approach has been described by some of us.^5^ The ratio of these peaks is proportional to the amount of material in solution. Hence, this enhanced hyperpolarized response can be used to probe NO production *in vitro* by ^1^H NMR spectroscopy.

**Fig. 5:**
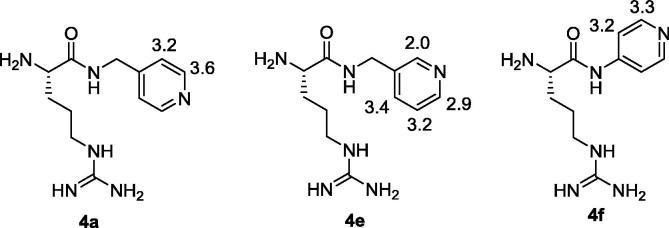
*T*_1_ relaxation times (in seconds) for pyridyl protons in compounds **4a,e**,**f** at pH = 7.4 and 37 °C.

Finally, relaxation times *T*_1_ ranging from 2.0 to 3.6 s were experimentally determined by ^1^H NMR in physiological conditions for selected pyridyl protons of compounds **4a,e,f** (Fig. 5). Although these *T*_1_ values are currently too low for *in vivo* MRI applications of these probes (*T*_1_ of at least 8–10 s would be required to guarantee sufficient polarization half-life), it is known that deuteration of adjacent protons could significantly increase *T*_1_ values,^6^ therefore deuterated versions of molecules **4** might be viable probes for future *in vivo* studies.

## 3 Conclusions

Novel eNOS substrates, l-arginine or l-NOHA analogues incorporating a pyridyl group, have been successfully synthesised for SABRE hyperpolarization. Compounds **4a**–**j** and **11a**–**c** showed a comparable or higher NO production by eNOS than l-arginine. Polarization transfer by SABRE was optimized to yield a maximum hyperpolarization of 870-fold enhancement on **4e**. *In vitro* spectroscopy was successfully carried out in the presence of eNOS, and a future application of MRI spectroscopy for imaging NO production using SABRE-hyperpolarised tracers *in vivo* might be possible, provided further increase of both hyperpolarisation – for achieving sufficient signal-to-noise ratio – and *T*_1_ – for achieving sufficiently long hyperpolarization half-life – can be achieved. The potential to resolve such a reaction *in vivo* depends on how the relaxation times of these resonances respond to the biochemical environment. It is well known, however, that ^13^C detection *in vivo* is also possible with hyperpolarised agents and hence should further tests reveal this to be a problem we expect to be able to harness a ^13^C labelling strategy to ensure success with these agents. To our knowledge, the library of compounds reported herein is the first attempt to develop SABRE-hyperpolarised tracers for quantification of NO production.

## 4 Experimental section

^1^H (400.13 MHz), ^13^C (100.58 MHz) NMR spectra were recorded on a Bruker ADVANCE III spectrometer. ^1^H NMR chemical shifts are reported relative to TMS, and the solvent resonance was employed as the internal standard (CDCl_3_ *δ* = 7.26, MeOD *δ* = 3.31, D_2_O *δ* = 4.79, DMSO-*d*_6_ *δ* = 2.50). ^13^C NMR spectra were recorded with complete proton decoupling, and the chemical shifts are reported relative to TMS with the solvent resonance as the internal standard (CDCl_3_, *δ* = 77.0, MeOD *δ* = 49.00, DMSO-*d*_6_ *δ* = 39.52). The following abbreviations are used to describe spin multiplicity: s = singlet, d = doublet, dd = doublet-doublet, dt = doublet-triplet, t = triplet, q = quartet, m = multiplet, bs = broad singlet. All chemical shifts (*δ*) are expressed in parts per million and coupling constants (*J*) are given in Hertz. LC–MS experiments were performed on an Agilent Technologies 1200 Series HPLC system equipped with a DAD and a 6120 MS detector composed by a ESI ionization source and a Single Quadrupole mass selective detector using an Analytical C18 RP Column (Phenomenex Luna C18 (2), 250 × 4.60 mm, 5 μm, 100 Å). HPLC purifications were performed on an Agilent 1260 system using a semi preparative C18 RP Column (Phenomenex Luna C18 (2), 250 × 10.00 mm, 5 μm, 100 Å). Optical rotation values were measured on an AA-65 Angular Scale automatic polarimeter (Optical Activity Limited) with a 1 dm cell at the sodium D line. Samples were freeze-dried on an Edwards Modulyo freeze drier. All commercially available reagents were used as received. Reactions were magnetically stirred and monitored by TLC on silica gel (60 F254 pre-coated glass plates, 0.25 mm thickness). Visualization was accomplished by irradiation with a UV lamp and/or staining with a ceric ammonium molybdate or potassium permanganate solution. Flash chromatography was performed on silica gel (60 Å, particle size 0.040–0.062 mm). Yields refer to chromatographically and spectroscopically pure compounds, unless stated otherwise. Abbreviations used: DCM for dichloromethane, EtOAc for ethyl acetate, Et_2_O for diethyl ether, MeOH for methanol, THF for tetrahydrofuran, MeOD for deuterated methanol, HATU for 1-[Bis(dimethylamino)methylene]-1*H*-1,2,3-triazolo[4,5-*b*]pyridinium 3-oxid hexafluorophosphate, TEA for triethylamine, TFA for trifluoroacetic acid, Boc for *tert*-butyloxycarbonyl, Pbf for 2,2,4,6,7-pentamethyldihydrobenzofuran-5-sulfonyl.

### 4.1 General procedure for the synthesis of compounds **2d** and **2i**

A solution of the corresponding pyridyl-l-alanine (150 mg; 0.90 mmol) in 2.5 mL of methanol was cooled down to −10 °C. SOCl_2_ (328 mL; 4.51 mmol) was added dropwise and the mixture stirred at r.t. for 36 h. Solvent was evaporated to yield the dihydrochloride products in 90%-quantitative yields.

#### 4.1.1 Methyl (2*S*)-2-amino-3-(pyridin-4-yl)propanoate (**2d**)

Precursor was 3-(4-pyridyl)-l-alanine **5**. Final product was a white solid (202 mg; 90%). R_f_ 0.5 (DCM/MeOH 9:1); ^1^H NMR (D_2_O, 400 MHz) *δ* 8.70–8.71 (d, *J* = 7.0 Hz, 2H), 7.97–7.99 (d, *J* = 6.1 Hz, 2H), 4.60–4.63 (t, *J* = 7.2 Hz, 1H), 3.73 (s, 3H), 3.56–3.62 (dd, *J* = 14.6, 8.0 Hz, 1H), 3.48–3.53 (dd, *J* = 14.6, 6.6 Hz, 1H); ^13^C NMR (D_2_O, 100 MHz) *δ* 168.9, 156.4, 141.3, 128.1, 53.8, 52.2, 35.4; MS (ESI), *m*/*z* calculated for [C_9_H_12_N_2_O_2_+H^+^] = 181.1, found 181.1; [α]_D_^20^ in CH_3_OH = +29.0 (c = 1.0).

#### 4.1.2 Methyl (2*S*)-2-amino-3-(pyridin-3-yl)propanoate (**2i**)

Precursor was 3-(3-pyridyl)-l-alanine **6**. Final product was a white solid (226 mg, quantitative). R_f_ 0.5 (DCM/MeOH 9:1); ^1^H NMR (D_2_O, 400 MHz) *δ* 8.73 (m, 1H), 8.67–8.6 9 (d, *J* = 5.6 Hz, 1H), 8.51–8.53 (d, *J* = 8.2 Hz, 1H), 7.98–8.02 (dd, *J* = 8.1, 5.9 Hz, 1H), 4.50–4.54 (t, *J* = 7.2 Hz, 1H), 3.69 (s, 3H), 3.49–3.55 (dd, *J* = 14.8, 7.7 Hz, 1H), 3.40–3.45 (dd, *J* = 14.8, 6.6 Hz, 1H); ^13^C (D_2_O, 100 MHz) *δ* 168.9, 148.1, 141.6, 140.5, 135.4, 127.6, 53.9, 52.7, 32.3; MS (ESI), *m*/*z* calculated for [C_9_H_12_N_2_O_2_+H^+^] = 181.1, found 181.1; [α]_D_^20^ in CH_3_OH = +22.0 (c = 1.0).

### 4.2 General procedure for the synthesis of compounds **3a**–**i**

To a solution of the amino acid Boc-l-Arg(Pbf)-OH (1 eq.; 150 mg; 0.28 mmol) in DCM (1.5 mL) was added, in the stated order, HATU (1.4 eq.; 152 mg; 0.40 mmol), TEA (2.9 eq.; 116 μL; 0.83 mmol) and the corresponding aminopyridine (1.2 eq.), unless stated otherwise. These mixtures were stirred at r.t. for 5 h. Crude mixtures were washed with HCl 1 M, sat. NaHCO_3_ and brine, dried over anhydrous Na_2_SO_4_, filtered and solvent evaporated. The remaining residues were purified by flash chromatography using a DCM/MeOH 94:6 mixture as solvent in 52–94% yield.

#### 4.2.1 *tert*-Butyl-*N*-[(1*S*)-4-{1-[(2,2,4,6,7-pentamethyl-2,3-dihydro-1-benzo-5-furanyl)sulfonyl]carbamimidamido}-1-[(4-pyridinylmethyl)carbamoyl]butyl]carbamate (**3a**)

Aminopyridine used was 4-(aminomethyl)pyridine **2a** (35 μL; 0.34 mmol). Final product was a white solid (91 mg; 53%). R_f_ 0.5 (DCM/MeOH 9:1); ^1^H NMR (CDCl_3_, 400 MHz) *δ* 8.41–8.42 (d, *J* = 5.6 Hz, 2H), 7.73 (bs, 1H), 7.12–7.13 (m, 2H), 6.11 (bs, 2H), 5.50–5.52 (m, 1H), 4.35–4.40 (dd, *J* = 15.8, 5.7 Hz, 1H), 4.26–4.32 (dd, *J* = 15.8, 5.9 Hz, 1H), 4.20 (m, 1H), 3.13–3.23 (m, 2H), 2.87 (s, 2H), 2.47 (s, 3H), 2.39 (s, 3H), 2.01 (s, 3H), 1.75–1.79 (m, 1H), 1.52–1.54 (m, 3H),1.39 (s, 6H), 1.34 (s, 9H); ^13^C NMR (CDCl_3_, 100 MHz) *δ* 173.3, 158.9, 156.6, 156.1, 149.5, 148.1, 138.3, 132.5, 132.2, 124.8, 122.3, 117.7, 86.5, 79.9, 54.1, 43.2, 42.0, 40.2, 30.0, 29.7, 28.6, 28.3, 28.3, 25.7, 19.3, 18.0, 12.5; MS (ESI), *m*/*z* calculated for [C_30_H_44_N_6_O_6_S+H^+^] = 617.3, found 617.2; [α]_D_^20^ in CHCl_3_ = +7.0 (c = 1.0).

#### 4.2.2 *tert*-Butyl-*N*-[(1*S*)-4-{1-[(2,2,4,6,7-pentamethyl-2,3-dihydro-1-benzo-5-furanyl)sulfonyl]carbamimidamido}-1-{[1-(pyridine-4-yl)ethyl]carbamoyl}butyl]carbamate (**3b**)

Aminopyridine used was 4-(1-aminoethyl)pyridine **2b** (41 μL; 0.34 mmol). Final product was a white solid (130 mg; 74%). R_f_ 0.5 (DCM/MeOH 9:1); ^1^H NMR (CDCl_3_, 400 MHz) *δ* 8.47–8.51 (m, 2H), 7.76–7.86 (m, 1H), 7.25 (m, 2H), 6.33 (bs, 3H), 5.78–5.85 (m, 1H), 4.97–5.02 (q, *J* = 6.9 Hz, 1H), 4.26 (m, 1H), 3.23 (m, 2H), 2.97 (s, 1H), 2.59 (s, 3H), 2.51 (s, 3H), 2.11 (s, 3H), 1.63–1.80 (m, 1H), 1.53–1.55 (m, 3H), 1.41–1.48 (m, 18H); ^13^C NMR (CDCl_3_, 100 MHz) *δ* 173.3, 173.1, 158.5, 156.7, 156.4, 154.3, 148.8, 148.6, 138.0, 133.0, 132.1, 124.6, 121.6, 117.1, 86.3, 79.3, 54.4, 42.6, 40.0, 29.2, 29.0, 27.4, 25.7, 20.3, 20.2, 18.2, 17.1, 11.2; MS (ESI) *m*/*z* calculated for [C_31_H_46_N_6_O_6_S+H^+^] = 631.3, found 631.2.

#### 4.2.3 *tert*-butyl-*N*-[(1*S*)-1-[methyl(pyridine-4-ylmethyl)carbamoyl]-4-{1-[(2,2,4,6,7-pentamethyl-2,3-dihydro-1-benzofuran-5-yl)sulfonyl]carbamimidamido}butyl]carbamate (**3c**)

Aminopyridine used was 4-[(methylamino)methyl]pyridine **2c** in its dihydrochloride salt (66 mg; 0.34 mmol). 4.9 eq. of TEA (191 μL; 1.37 mmol) were added instead. Forms rotamers giving multiple peaks on the NMR. ^1^H NMR spectrum was acquired at 80 °C. ^13^C NMR reported is acquired at 25 °C. Final product was a white solid (121 mg; 69%). R_f_ 0.5 (DCM/MeOH 9:1); ^1^H NMR (DMSO-*d*_6_, 400 MHz, 80 °C) *δ* 8.50–8.51 (m, 2H), 7.19–7.20 (m, 2H), 6.54–6.58 (bs, 2H), 6.43 (s, 2H), 4.59–4.63 (m, 2H), 4.40–4.41 (m, 1H), 2.97–3.08 (m, 10 H), 2.47 (s, 3H), 2.04 (s, 3H), 1.48–1.64 (m, 4H), 1.43 (s, 6H), 1.39 (s, 9H); ^13^C NMR (CDCl_3_, 100 MHz) *δ* 172.8, 158.7, 156.3, 156.2, 155.8, 150.3, 150.1, 145.8, 145.6, 138.3, 133.0, 132.2, 124.6, 122.4, 121.7, 117.5, 86.4, 80.1, 52.3, 50.6, 49.9, 43.2, 40.8, 35.3, 34.4, 30.8, 30.4, 28.6, 28.3, 28.2, 25.0, 24.9, 19.3, 17.9, 12.5; MS (ESI) *m*/*z* calculated for [C_31_H_46_N_6_O_6_S+H^+^] = 631.3, found 631.2; [α]_D_^20^ in CHCl_3_ = 0 (c = 1.0).

#### 4.2.4 Methyl (2*S*)-2-[(2*S*)-2-{[(*tert*-butoxy)carbonyl]amino}-5-{1-[(2,2,4,6,7-pentamethyl-2,3-dihydro-1-benzofuran-5-yl)sulfonyl]carbamimidamido}pentanamido]-3-(pyridin-4-yl)propanoate (**3d**)

Aminopyridine used was methyl (2*S*)-2-amino-3-(4-pyridinyl)propanoate **2d** (61 mg; 0.34 mmol). Final product was a white solid (100 mg; 52%). R_f_ 0.6 (DCM/MeOH 9:1); ^1^H NMR (CDCl_3_, 400 MHz) *δ* 8.36–8.38 (m, 2H), 7.60 (bs, 1H), 7.05–7.07 (m, 2H), 6.04–6.25 (bs, 3H), 5.55–5.57 (m, 1H), 4.73–4.78 (q, *J* = 7.3 Hz, 1H), 4.10 (m, 1H), 3.63 (s, 3H), 3.08–3.13 (m, 3), 2.93–2.99 (m, 1H), 2.88 (s, 2H), 2.50 (s, 3H), 2.43 (s, 3.15), 2.02 (s, 3H), 1.66 (m, 1H), 1.43–1.66 (m, 3H), 1.38 (s, 6H), 1.32 (s, 9H); ^13^C NMR (CDCl_3_, 100 MHz) *δ* 172.7, 171.5, 158.8, 156.5, 155.9, 149.6, 146.1, 138.3, 132.7, 132.2, 124.7, 117.6, 86.4, 79.9, 53.9, 52.6, 52.5, 43.2, 40.4, 36.7, 29.9, 28.6, 28.3, 25.4, 19.3, 18.0, 12.5; MS (ESI) *m*/*z* calculated for [C_33_H_48_N_6_O_8_S+H^+^] = 689.3, found 689.2; [α]_D_^20^ in CHCl_3_ = -2.0 (c = 1.0).

#### 4.2.5 *tert*-Butyl-*N*-[(1*S*)-4-{1-[(2,2,4,6,7-pentamethyl-2,3-dihydro-1-benzofuran-5-yl)sulfonyl]carbamimidamido}-1-[(pyridine-4-yl)carbamoyl]butyl]carbamate (**3e**)

Aminopyridine used was 4-aminopyridine **2e** (32 mg; 0.34 mmol). Final product was a white solid (104 mg; 62%). R_f_ 0.5 (DCM/MeOH 9:1); ^1^H NMR (CDCl_3_, 400 MHz) *δ* 9.68 (bs, 1H), 8.44–8.45 (d, *J* = 6.1 Hz, 2H), 7.64 (m, 2H), 6.26 (bs, 2H), 5.67–5.69 (m, 1H), 4.59 (m, 1H), 3.33 (m, 2H), 2.98 (s, 2H), 2.61 (s, 3H), 2.54 (s, 3H), 2.12 (s, 3H), 1.93 (m, 1H), 1.69 (m, 3H), 1.49 (s, 6H), 1.45 (s, 9H); ^13^C NMR (CDCl_3_, 100 MHz) *δ* 172.4, 159.0, 156.6, 156.2, 150.2, 145.7, 138.4, 132.3, 132.2, 124.9, 117.8, 114.2, 86.6, 80.2, 54.6, 43.2, 40.2, 29.7, 28.6, 28.3, 25.7, 19.4, 18.0, 12.5; MS (ESI) *m*/*z* calculated for [C_29_H_42_N_6_O_6_S+H^+^] = 603.3, found 603.2; [α]_D_^20^ in CHCl_3_ = +6.0 (c = 1.0).

#### 4.2.6 *tert*-Butyl *N*-[(1*S*)-4-{1-[(2,2,4,6,7-pentamethyl-2,3-dihydro-1-benzofuran-5-yl)sulfonyl]carbamimidamido}-1-[(pyridine-3-ylmethyl)carbamoyl]butyl]carbamate (**3f**)

Aminopyridine used was 3-(aminomethyl)pyridine **2f** (35 μL; 0.34 mmol). Final product was a white solid (162 mg; 94%). R_f_ 0.5 (DCM/MeOH 9:1); ^1^H NMR (CDCl_3_, 400 MHz) *δ* 8.47 (s, 1H), 8.40–8.41 (m, 1H), 7.97 (bs, 1H), 7.58–7.60 (m, 1H), 7.16–7.19 (dd, *J* = 4.9, 7.6 Hz, 1H), 6.42 (bs, 3H), 5.85–5.87 (m, 1H), 4.38–4.43 (dd, *J* = 5.9, 15.3 Hz, 1H), 4.31–4.36 (dd, *J* = 5.9, 15.3 Hz, 1H), 4.21 (m, 1H), 3.12–3.26 (m, 2H), 2.94 (s, 2H), 2.54 (s, 3H), 2.46 (s, 3H), 2.08 (s, 3H), 1.78–1.80 (m, 1H), 1.51–1.61 (m, 3H), 1.46 (s, 6H), 1.37 (s, 9H); ^13^C NMR (CDCl_3_, 100 MHz) *δ* 173.1, 158.9, 156.6, 156.0, 148.7, 148.2, 138.3, 135.6, 134.4, 132.6, 132.2, 124.7, 123.6, 117.6, 86.5, 79.8, 53.9, 43.2, 40.7, 40.2, 30.1, 29.7, 28.6, 28.3, 25.7, 19.3, 18.0, 12.5; MS (ESI) *m*/*z* calculated for [C_29_H_42_N_6_O_6_S+H^+^] = 617.3, found 617.2; [α]_D_^20^ in CHCl_3_ = -5.0 (c = 1.0).

#### 4.2.7 *tert*-Butyl *N*-[(1*S*)-4-{1-[(2,2,4,6,7-pentamethyl-2,3-dihydro-1-benzofuran-5-yl)sulfonyl]carbamimidamido}-1-{[1-(pyridine-3-yl)ethyl]carbamoyl}butyl]carbamate (**3g**)

Aminopyridine used was 3-(1-aminoethyl)pyridine **2g** (41 μL; 0.34 mmol). Final product was a white solid (132 mg; 75%). R_f_ 0.5 (DCM/MeOH 9:1); ^1^H NMR (CDCl_3_, 400 MHz) *δ* 8.50 (s, 1H), 8.36–8.37 (m, 1H), 7.81–7.91 (m, 1H), 7.61 (m, 1H), 7.12–7.18 (m, 1H), 6.39 (bs, 3H), 5.85–5.95 (m, 1H), 4.95–4.98 (m, 1H), 4.12–4.18 (m, 1H), 3.14–3.37 (m, 2H), 2.91 (s, 2H), 2.53 (s, 3H), 2.46 (s, 3H), 2.05 (s, 3H), 1.57–1.71 (m, 4H), 1.42 (s, 9H), 1.33–1.35 (m, 9H); ^13^C NMR (CDCl_3_, 100 MHz) *δ* 172.1, 158.8, 156.6, 156.6, 156.0, 148.1, 148.1, 147.9, 147.6, 139.6, 139.3, 138.3, 138.3, 134.2, 134.1, 132.7, 132.2, 130.9, 128.8 124.7, 123.6, 123.6, 117.6, 86.4, 79.8, 53.8, 47.1, 43.2, 40.4, 29.7, 28.6, 28.3, 28.3, 25.6, 21.8, 21.6, 19.4, 18.0, 12.5; MS (ESI) *m*/*z* calculated for [C_31_H_46_N_6_O_6_S+H^+^] = 631.3, found 631.2.

#### 4.2.8 *tert*-Butyl *N*-[(1*S*)-1-[methyl(pyridine-3-ylmethyl)carbamoyl]-4-{1-[(2,2,4,6,7-pentamethyl-2,3-dihydro-1-benzofuran-5-yl)sulfonyl]carbamimidamido}butyl]carbamate (**3h**)

Aminopyridine used was 3-[(methylamino)methyl]pyridine **2h** (42 μL; 0.34 mmol). Forms rotamers. ^1^H NMR spectrum was acquired at 80 °C. ^13^C NMR reported is acquired at 25 °C. Final product was a white solid (132 mg; 75%). R_f_ 0.5 (DCM/MeOH 9:1); ^1^H NMR (DMSO-*d*_6_, 400 MHz, 80 °C) *δ* 8.48 (m, 2H), 7.61–7.63 (d, *J* = 7.8 Hz, 1H), 7.31–7.34 (dd, *J* = 4.7, 7.6 Hz, 1H), 6.43–6.58 (m, 4H), 4.40–4.63 (m, 3H), 3.05–3.09 (m, 4H), 2.95–2.97 (m, 5H), 2.47 (s, 3H), 2.04 (s, 3H), 1.47–1.66 (m, 4H), 1.44 (s, 6H), 1.39 (s, 9H); ^13^C NMR (CDCl_3_, 100 MHz) *δ* 172.6, 158.7, 156.3, 156.1, 149.2, 149.0, 148.5, 138.3, 135.7, 134.8, 133.0, 132.4, 132.2, 131.8, 130.9, 128.8, 124.6, 123.8, 117.4, 86.4, 80.0, 50.9, 49.9, 49.1, 43.2, 40.8, 35.0, 34.0, 30.8, 30.5, 28.6, 28.3, 28.3, 24.9, 19.3, 17.9, 12.5; MS (ESI) *m*/*z* calculated for [C_31_H_46_N_6_O_6_S+H^+^] = 631.3, found 631.2; [α]_D_^20^ in CHCl_3_ = -6.0 (c = 1.0).

#### 4.2.9 Methyl (2*S*)-2-[(2*S*)-2-{[(*tert*-butoxy)carbonyl]amino}-5-{1-[(2,2,4,6,7-pentamethyl-2,3-dihydro-1-benzofuran-5-yl)sulfonyl]carbamimidamido}pentanamido]-3-(pyridine-3-yl)propanoate (**3i**)

Amounts used were doubled: Boc-l-Arg(Pbf)-OH (300 mg; 0.57 mmol), HATU (304 mg; 0.80 mmol), TEA (232 μL; 1.66 mmol), methyl (2*R*)-2-amino-3-(3-pyridinyl)propanoate **2i** (122 mg; 0.68 mmol).White solid (237 mg; 60%). R_f_ 0.6 (DCM/MeOH 9:1); ^1^H NMR (CDCl_3_, 400 MHz) *δ* 8.40–8.42 (m, 2H), 7.63–7.65 (bs, 1H), 7.56–7.58 (d, *J* = 7.8 Hz, 1H), 7.19–7.22 (dd, *J* = 7.8, 4.9 Hz, 1H), 6.30–6.53 (bs, 3H), 5.79–5.80 (bs, 1H), 4.77–4.83 (m, 1H), 4.12 (m, 1H), 3.70 (s, 3H), 3.17–3.22 (m, 3H), 2.99–3.05 (m, 1H), 2.96 (s, 2H), 2.60 (s, 3H), 2.52 (s, 3H), 2.10 (s, 3H), 1.55–1.73 (m, 4H), 1.47 (s, 6H), 1.39 (s, 9H); ^13^C NMR (CDCl_3_, 100 MHz) *δ* 172.6, 171.5, 158.8, 156.5, 155.8, 150.2, 147.9, 138.4, 137.3, 132.8, 132.6, 132.3, 124.7, 123.6, 117.6, 86.4, 79.9, 54.3, 53.1, 52.5, 43.2, 40.4, 34.7, 29.7, 28.6, 28.3, 25.5, 19.3, 18.0, 12.5; MS (ESI) *m*/*z* calculated for [C_31_H_46_N_6_O_6_S+H^+^] = 689.3, found 689.2; [α]_D_^20^ in CHCl_3_ = -5.0 (c = 1.0).

### 4.3 General procedure for the synthesis of compounds **4a**–**i**

Precursor was dissolved in 1 mL of TFA and stirred at r.t. for 2 h, unless stated otherwise. Solvent was evaporated under a current of air, triturated with Et_2_O, dissolved in water and passed through a Sep-Pak Plus® C18 cartridge previously conditioned by passing methanol (10 mL) followed by water (10 mL). Solvent was finally freeze dried and TFA salt exchanged to HCl salt by adding some drops of HCl 2 M prior to freeze-drying. Products were obtained in 38%-quant yield.

#### 4.3.1 (2*S*)-2-Amino-5-carbamimidamido-*N*-(pyridin-4-ylmethyl)pentanamide (**4a**)

Precursor was **3a** (63 mg; 0.10 mmol). Final product was a white solid (27 mg; quant.). ^1^H NMR (D_2_O, 400 MHz) *δ* 8.67–8.68 (d, *J* = 6.4 Hz, 2H), 7.90–7.92 (d, *J* = 6.2 Hz, 2H), 4.12–4.15 (t, *J* = 6.5 Hz, 1H), 3.18–3.21 (t, *J* = 6.7 Hz, 2H), 1.91–1.98 (quin, *J* = 6.9 Hz, 2H), 1.59–1.66 (quin, *J* = 7.4 Hz, 2H); ^13^C NMR (D_2_O, 100 MHz) *δ* 170.3, 159.4, 156.8, 141.1, 125.3, 52.9, 42.7, 40.3, 28.0, 23.7; MS (ESI) *m*/*z* calculated for [C_12_H_20_N_6_O+H^+^] = 265.1699, found 265.1774; [α]_D_^20^ in CH_3_OH = +10.0 (c = 1.0).

#### 4.3.2 (2*S*)-2-Amino-5-carbamimidamido-*N*-[1-(pyridin-4-yl)ethyl]pentanamide (**4b**)

Precursor was **3b** (102 mg; 0.16 mmol). Final product was a white solid (39 mg; 87%). Two different diastereoisomers, both reported. Minor diastereoisomer is marked with an asterisk (∗) on spectra. Major diastereoisomer: ^1^H NMR (D_2_O, 400 MHz) *δ* 8.66–8.67 (d, *J* = 6.7 Hz, 2H), 7.92–7.95 (m, 2H), 5.06–5.11 (m, 1H), 4.02–4.07 (m, 1H), 3.10–3.14 (t, *J* = 6.8 Hz, 2H), 1.82–1.92 (m, 2H), 1.57–1.65 (m, 1H), 1.46–1.48 (m, 4H). Minor diastereoisomer: ^1^H NMR (D_2_O, 400 MHz) *δ* 8.63–8.65 (d, *J* = 6.7 Hz, 2H), 7.92–7.95 (m, 2H), 5.06–5.11 (m, 1H), 4.02–4.07 (m, 1H), 3.15–3.18 (t, *J* = 6.8 Hz, 2H), 1.82–1.92 (m, 2H), 1.57–1.65 (m, 1H), 1.46–1.48 (m, 4H); ^13^C NMR (D_2_O, 100 MHz) *δ* 169.4, 169.3, 164.0, 163.9, 156.7, 156.7, 141.5, 141.3, 124.6, 124.5, 52.8, 52.6, 49.8, 49.7, 40.3, 40.3, 28.0, 23.8, 23.4, 20.1, 19.9; MS (ESI) *m*/*z* calculated for [C_12_H_20_N_6_O+H^+^] = 279.1855, found 279.1927; [α]_D_^20^ in CH_3_OH = +7.0 (c = 1.0).

#### 4.3.3 (2*S*)-2-Amino-5-carbamimidamido-N-methyl-N-(pyridin-4-ylmethyl)pentanamide (**4c**)

Precursor was **3c** (104 mg; 0.16 mmol). Final product was a white solid (27 mg; 59%). Product forms rotamers. ^1^H NMR spectrum reported was acquired at 80 °C. ^13^C NMR spectrum was acquired at 25 °C. ^1^H NMR (D_2_O, 400 MHz) *δ* 9.24–9.25 (d, *J* = 6.1 Hz, 2H), 8.24–8.25 (m, 2H), 5.20 (m, 1H), 3.83–3.86 (t, *J* = 6.2 Hz, 2H), 3.78 (s, 3H), 2.57–2.59 (m, 2H), 2.30–2.32 (m, 2H); ^13^C NMR (D_2_O, 100 MHz) *δ* 170.4, 163.1, 162.7, 158.1, 156.8, 141.6, 141.2, 125.2, 125.0, 117.8, 114.9, 51.6, 50.5, 40.3, 36.1, 26.9, 23.4; MS (ESI) *m*/*z* calculated for [C_12_H_20_N_6_O+H^+^] = 279.1981, found 279.2053; [α]_D_^20^ in CH_3_OH = +20.0 (c = 1.0).

#### 4.3.4 Methyl (2*S*)-2-[(2*S*)-2-amino-5-carbamimidamidopentanamido]-3-(pyridin-4-yl)propanoate (**4d**)

Precursor was **3d** (79 mg; 0.11 mmol). Final product was a white solid (14 mg; 38%). ^1^H NMR (D_2_O, 400 MHz) *δ* 8.64–8.65 (d, *J* = 6.3 Hz, 2H), 7.92–7.94 (d, *J* = 6.2 Hz, 2H), 4.97–5.01 (dd, *J* = 9.1, 5.7 Hz, 1H), 3.94–3.97 (t, *J* = 6.3 Hz, 2H), 3.70 (s, 3H), 3.55–3.60 (dd, *J* = 14.6, 5.5 Hz, 1H), 3.30–3.36 (dd, *J* = 14.6, 9.3 Hz, 1H), 3.15–3.18 (t, *J* = 6.7 Hz, 2H), 1.83–1.89 (q, *J* = 7.7 Hz, 2H), 1.55–1.64 (m, 2H); ^13^C NMR (D_2_O, 100 MHz) *δ* 171.6, 169.6, 158.7, 156.8, 140.8, 127.9, 53.3, 52.5, 52.3, 40.3, 36.4, 28.0, 23.4; MS (ESI) *m*/*z* calculated for [C_15_H_24_N_6_O_3_+H^+^] = 337.1910, found 337.1984; [α]_D_^20^ in CH_3_OH = +4.0 (c = 1.0).

#### 4.3.5 (2*S*)-2-Amino-5-carbamimidamido-N-(pyridin-4-yl)pentanamide (**4e**)

Precursor was **3e** (88 mg; 0.15 mmol). Final product was a white solid (26 mg; 71%). ^1^H NMR (D_2_O, 400 MHz) *δ* 8.56–8.58 (d, *J* = 6.8 Hz, 2H), 8.10–8.11 (d, *J* = 6.7 Hz, 2H), 4.28–4.31 (t, *J* = 6.1 Hz, 1H), 3.17–3.21 (t, *J* = 6.6 Hz, 2H), 1.97–2.10 (m, 2H), 1.69–1.71 (m, 2H); ^13^C NMR (D_2_O, 100 MHz) *δ* 169.7, 156.7, 152.5, 142.0, 115.9, 53.9, 40.2, 27.6, 23.4; MS (ESI) *m*/*z* calculated for [C_11_H_18_N_6_O+H^+^] = 251.1542, found 251.1616; [α]_D_^20^ in CH_3_OH = +24.0 (c = 1.0).

#### 4.3.6 (2*S*)-2-Amino-5-carbamimidamido-*N*-(pyridin-3-ylmethyl)pentanamide (**4f**)

Precursor was **3f** (154 mg; 0.25 mmol). Final product was a white solid (61 mg; 92%). ^1^H NMR (D_2_O, 400 MHz) *δ* 8.67 (s, 1H), 8.63–8.64 (d, *J* = 5.7 Hz, 1H), 8.45–8.47 (d, *J* = 8.1 Hz, 1H), 7.95–7.98 (dd, *J* = 5.8, 7.7 Hz, 1H), 4.58–4.62 (d, *J* = 15.9 Hz, 1H), 4.51–4.55 (d, *J* = 15.9 Hz, 1H), 3.99–4.02 (t, *J* = 6.5 Hz, 1H), 3.10–3.13 (t, *J* = 6.9 Hz, 2H), 1.82–1.89 (m, 2H), 1.49–1.56 (m, 2H); ^13^C NMR (D_2_O, 100 MHz) *δ* 170.0, 162.2–163.3 (q, *J*_C-F_ = 35.0 Hz), 156.7 146.0, 140.2, 140.0, 138.3, 127.4, 112.0–120.7 (q, *J*_C-F_ = 290.7 Hz), 52.8, 40.2, 40.1, 27.9, 23.6; MS (ESI) *m*/*z* calculated for [C_12_H_20_N_6_O+H^+^] = 265.1699, found = 265.1762; [α]_D_^20^ in CH_3_OH = +23.0 (c = 1.0).

#### 4.3.7 (2*S*)-2-Amino-5-carbamimidamido-*N*-[1-(pyridin-3-yl)ethyl]pentanamide (**4g**)

Precursor was **3g** (115 mg; 0.18 mmol). Final product was a white solid (51 mg; quant.). Two different diastereoisomers, both reported. Minor diastereoisomer is marked with an asterisk (∗) on spectra. Major diastereoisomer: ^1^H NMR (D_2_O, 400 MHz) *δ* 8.75 (s, 1H), 8.67–8.68 (d, *J* = 5.7 Hz, 1H), 8.54–8.56 (d, *J* = 8.1 Hz, 1H), 7.98–8.04 (m, 1H), 5.10–5.15 (q, *J* = 7.0 Hz, 1H), 3.98–4.01 (m, 1H), 3.08–3.11 (t, *J* = 6.8 Hz, 2H), 1.78–1.84 (q, *J* = 7.8 Hz, 2H), 1.52 (s, 3H), 1.35–1.47 (m, 2H); ^13^C NMR (D_2_O, 100 MHz) *δ* 169.2, 156.7, 144.7, 143.2, 140.4, 139.4, 127.7, 52.6, 47.4, 40.2, 28.0, 23.8, 20.1. Minor diastereoisomer: ^1^H NMR (D_2_O, 400 MHz) *δ* 8.73 (s, 1H), 8.63–8.65 (d, *J* = 5.7 Hz, 1H), 8.54–8.56 (d, *J* = 8.1 Hz, 1H), 7.98–8.04 (m, 1H), 5.10–5.15 (q, *J* = 7.0 Hz, 1H), 3.98–4.01 (m, 1H), 3.17–3.20 (t, *J* = 6.8 Hz, 2H), 1.87–1.93 (q, *J* = 7.8 Hz, 2H), 1.59–1.66 (m, 2H), 1.53 (s, 3H); ^13^C NMR (D_2_O, 100 MHz) *δ* 169.2, 156.8, 144.8, 143.1, 140.1, 139.2, 127.5, 52.8, 40.3, 28.0, 23.6, 19.8; MS (ESI) *m*/*z* calculated for [C_13_H_22_N_6_O+H^+^] = 279.1855, found 279.1928; [α]_D_^20^ in CH_3_OH = +12.0 (c = 1.0).

#### 4.3.8 (2*S*)-2-Amino-5-carbamimidamido-N-methyl-*N*-(pyridin-3-ylmethyl)pentanamide (**4h**)

Precursor was **3h** (114 mg; 0.18 mmol). Final product was a white solid (50 mg; quant.). Product forms rotamers, ^1^H NMR spectrum reported was acquired at 80 °C. ^13^C was acquired at 25 °C. ^1^H NMR (D_2_O, 400 MHz, 80 °C) *δ* 9.29–9.31 (m, 2H), 9.07–9.09 (d, *J* = 8.1 Hz, 1H), 8.63–8.66 (m, 1H), 5.43–5.47 (d, *J* = 15.8 Hz, 1H), 5.26–5.30 (d, 15.8 Hz, 1H), 5.12–5.15 (t, *J* = 6.1 Hz), 3.77–3.81 (t, *J* = 6.8 Hz, 2H), 3.74 (s, 3H), 2.49–2.54 (m, 2H), 2.20–2.28 (m, 2H); ^13^C NMR (D_2_O, 100 MHz) *δ* 170.7, 157.7, 147.1, 141.2, 140.9, 137.7, 128.4, 51.4, 49.8, 41.2, 36.6, 27.8, 24.1; HRMS (ESI) *m*/*z* calculated for [C_13_H_22_N_6_O+H^+^] = 279.1855, found 279.1918; [α]_D_^20^ in CH_3_OH = +24.0 (c = 1.0).

#### 4.3.9 Methyl (2*S*)-2-[(2*S*)-2-amino-5-carbamimidamidopentanamido]-3-(pyridin-3-yl)propanoate (**4i**)

Precursor was **3i** (201 mg; 0.29 mmol). Final product was a white solid (85 mg; 86%). ^1^H NMR (D_2_O, 400 MHz) *δ* 8.68 (s, 1H), 8.62–8.63 (d, *J* = 5.6 Hz, 1H), 8.47–8.49 (d, *J* = 7.8 Hz, 1H), 7.94–7.98 (m, 1H), 4.86–4.90 (dd, *J* = 8.7, 5.7 Hz, 1H), 3.95–3.98 (t, *J* = 6.3 Hz, 1H), 3.67 (s, 3H), 3.44–3.49 (dd, *J* = 14.6, 5.4 Hz, 1H), 3.22–3.28 (dd, *J* = 14.6, 8.9 Hz, 1H) 3.12–3.16 (t, *J* = 6.6 Hz, 2H), 1.81–1.87 (m, 2H), 1.52–1.61 (m, 2H); ^13^C NMR (D_2_O, 100 MHz) *δ* 171.6, 169.6, 156.7, 147.8, 141.2, 139.9, 137.2, 127.3, 53.3, 52.8, 52.6, 40.4, 33.2, 28.0, 23.4; MS (ESI) *m*/*z* calculated for [C_12_H_20_N_6_O+H^+^] = 337.1910, found 337.1983; [α]_D_^20^ in CH_3_OH = +1.0 (c = 1.0).

### 4.4 (2*S*)-2-[(2*S*)-2-Amino-5-carbamimidamidopentanamido]-3-(pyridin-3-yl)propanoic acid (**4j**)

Methyl (2*S*)-2-[(2*S*)-2-{[(*tert*-butoxy)carbonyl]amino}-5-{1-[(2,2,4,6,7-pentamethyl-2,3-dihydro-1-benzofuran-5-yl)sulfonyl]carbamimidamido}pentanamido]-3-(pyridin-3-yl)propanoate **2i** (170 mg; 0.25 mmol) and LiOH (15 mg; 0.62 mmol) were dissolved in 2 mL of a THF/H_2_O 4:1 mixture, and allowed to react overnight to yield **3j**. Upon reaction completion, crude mixture was dissolved in TFA, and general procedure for Boc and Pbf cleavage was followed. Final product was a white solid (54 mg; 68%). ^1^H NMR (D_2_O, 400 MHz) *δ* 8.35 (s, 1H), 8.29–8.30 (d, *J* = 5.7 Hz, 1H), 8.16–8.18 (d, *J* = 7.8 Hz, 1H), 7.61–7.65 (dd, *J* = 6.2, 8.3 Hz, 1H), 4.47–4.51 (m, 1H), 3.65–3.68 t, *J* = 6.3 Hz, 1H), 3.10–3.15 (dd, *J* = 5.5, 14.7 Hz, 1H), 2.91–2.97 (dd, *J* = 8.8, 14.7 Hz, 1H), 2.78–2.81 (t, *J* = 6.8 Hz, 2H), 1.49–1.54 (m, 2H), 1.19–1.28 (m, 2H); ^13^C NMR (D_2_O, 100 MHz) *δ* 172.5, 169.2, 156.3, 147.6, 140.9, 139.6, 137.0, 127.1, 52.5, 52.4, 40.1, 32.9, 27.7, 23.1; MS (ESI) *m*/*z* calculated for [C_12_H_20_N_6_O+H^+^] = 323.2, found 323.1; [α]_D_^20^ in CH_3_OH = -18.0 (c = 1.0).

### 4.5 *tert*-Butyl-*N*-[(1*S*)-4-(carbamoylamino)-1-[(pyridine-4-ylmethyl)carbamoyl]butyl]carbamate (**8**)

To a solution of l-Boc-Cit-OH **7** (750 mg; 2.72 mmol) in 15 mL of DCM was added, in the stated order, HATU (1148 mg; 3.81 mmol), TEA (1100 μL; 7.89 mmol) and 4-picolylamine (331 μL; 3.26 mmol). This mixture was stirred at r.t. for 5 h. Precipitate formed was filtered, washed with DCM and dried under vacuum to yield a white solid (776 mg; 78%). R_f_ 0.5 (DCM/MeOH 8:2); ^1^H NMR (MeOD, 400 MHz) *δ* 8.46–8.47 (d, *J* = 5.7 Hz, 2H), 7.38–7.39 (d, *J* = 5.7 Hz, 2H), 4.46–4.47 (m, 2H), 4.06–4.0 (m, 1H), 3.12–3.17 (q, *J* = 6.5 Hz, 2H), 1.77–1.84 (m, 1H), 1.52–1.69 (m, 3H), 1.47 (s, 9H); ^13^C NMR (MeOD, 100 MHz) *δ* 174.4, 160.9, 156.6, 149.4, 148.6, 122.4, 79.3, 54.8, 41.4, 39.0, 29.0, 27.3, 26.5; MS (ESI), *m*/*z* calculated for [C_17_N_27_N_5_O_4_+H^+^] = 366.2, found 366.2; [α]_D_^20^ in MeOH = +5.0 (c = 1.0).

### 4.6 *tert*-Butyl *N*-[(1*S*)-4-(cyanoamino)-1-[(pyridine-4-ylmethyl)carbamoyl]butyl]carbamate (**9**)

A solution of *tert*-butyl-*N*-[(1*S*)-4-(carbamoylamino)-1-[(pyridine-4-ylmethyl)carbamoyl]butyl]carbamate **8** (358 mg; 0.98 mmol) in 5 mL of pyridine was treated with CH_3_SO_2_Cl dropwise (152 μL; 1.96 mmol). Mixture was left to react at r.t. for 2 h. Pyridine was evaporated, and crude mixture purified by flash chromatography using a DCM/MeOH 95:5 mixture as eluent, to afford a waxy yellow solid (104 mg; 31%). R_f_ 0.5 (DCM/MeOH 9:1); ^1^H NMR (MeOD, 400 MHz) *δ* 8.46–8.48 (m, 2H), 7.38–7.39 (d, *J* = 5.8 Hz, 2H), 4.47 (s, 2H), 4.08–4.10 (m, 1H), 3.05–3.08 (t, *J* = 6.8 Hz, 2H), 1.85–1.89 (m, 1H), 1.64–1.74 (m, 3H), 1.48 (s, 9H); ^13^C NMR (MeOD, 100 MHz) *δ* 174.0, 156.6, 149.4, 148.6, 122.4, 117.2, 79.4, 54.6, 44.8, 41.4, 28.6, 27.3, 26.1; MS (ESI), *m*/*z* calculated for [C_17_H_25_N_5_O_3_+H^+^] = 347.2, found 348.2; [α]_D_^20^ in MeOH = +4.0 (c = 1.0).

### 4.7 *tert*-Butyl *N*-[(1*S*)-4-(2-hydroxycarbamimidamido)-1-[(pyridine-4-ylmethyl)carbamoyl]butyl]carbamate (**10**)

Precursor *tert*-butyl *N*-[(1*S*)-4-(cyanoamino)-1-[(pyridine-4-ylmethyl)carbamoyl]butyl]carbamate **9** (98 mg; 0.31 mmol) was dissolved in 1.5 mL of EtOH. To this solution, finely ground K_2_CO_3_ (85 mg; 0.61 mmol) and NH_2_OH·HCl (43 mg; 0.61 mmol) were added, and mixture stirred for 1 h at r.t. Solvent was evaporated, and crude mixture purified by HPLC. Final product was a white solid (89 mg; 83%). ^1^H NMR (MeOD, 400 MHz) *δ* 8.78 (m, 2H), 7.99–8.00 (m, 2H), 4.69–4.70 (m, 2H), 4.08–4.12 (m, 1H), 3.25–3.30 (t, *J* = 6.5 Hz, 2H), 1.85–1.90 (m, 1H), 1.71–1.78 (m, 3H), 1.48 (s, 9H); ^13^C NMR (MeOD, 100 MHz) *δ* 174.6, 160.4, 159.1, 156.7, 141.5, 124.9, 79.6, 54.7, 42.1, 40.6, 28.4, 27.3, 25.1; MS (ESI), *m*/*z* calculated for [C_17_H_28_N_6_O_4_+H^+^] = 381.2, found 381.2; [α]_D_^20^ in MeOH = +6.0 (c = 1.0).

### 4.8 (2*S*)-2-Amino-5-(2-hydroxycarbamimidamido)-*N*-(pyridin-4-ylmethyl)pentanamide (**11a**)

*tert*-Butyl *N*-[(1*S*)-4-(2-hydroxycarbamimidamido)-1-[(pyridin-4-ylmethyl)carbamoyl]butyl]carbamate **10** (78 mg; 0.21 mmol) was dissolved in 1 mL of a 1 M HCl solution and left to react overnight. Solvent was freeze-dried, and a white solid was obtained (70 mg; quant.). ^1^H NMR (MeOD, 400 MHz) *δ* 8.85–8.86 (d, *J* = 5.2 Hz, 2H), 8.09–8.11 (d, *J* = 5.3 Hz, 2H), 4.83 (m, under H_2_O signal, 1H), 4.70–4.75 (d, *J* = 17.3 Hz, 1 H), 4.14–4.19 (t, *J* = 5.8 Hz, 2H), 3.35–3.37 (m, under MeOD signal, 2H), 1.97–2.07 (m, 2H), 1.78–1.80 (m, 2H); ^13^C NMR (MeOD, 100 MHz) *δ* 169.4, 160.4, 159.0, 141.3, 125.5, 52.8, 42.3, 40.3, 28.3, 24.3; MS (ESI), *m*/*z* calculated for [C_14_H_21_N_5_O_3_+H^+^] = 281.1648, found 281.1722; [α]_D_^20^ in MeOH = +24.0 (c = 1.0).

### 4.9 Methyl 5-aminovalerate hydrochloride (**13**)

5-Aminovaleric acid **12** (2 g; 17.07 mmol) was dissolved in CH_3_OH (25 mL). The solution was cooled down to -10 °C, and SOCl_2_ (2.84 mL; 39.06 mmol) was added dropwise onto the stirred solution. Mixture was warmed to room temperature and left to react overnight. Solvent was evaporated under vacuum and the solid residue triturated in Et_2_O. Final product was a white solid (2.751 mg; 96%). R_f_ 0.7 (DCM/MeOH/NH_4_OH 85:14:1); ^1^H NMR (MeOD, 400 MHz) *δ* 3.69 (s, 3H), 2.96 (m, 2H), 2.41–2.45 (m, 2H), 1.70–1.74 (m, 4H); ^13^C NMR (MeOD, 100 MHz) *δ* 173.9, 50.9, 39.1, 32.7, 26.5, 21.5; MS (ESI), *m*/*z* calculated for [C_6_H_13_NO_2_+H^+^] = 132.1, found 132.1.

### 4.10 Methyl 5-[(*tert*-butoxicarbonyl)amino]valerate (**14**)

Methyl 5-aminovalerate **13** (1.575 g; 9.43 mmol) was suspended in CH_2_Cl_2_ (20 mL). Boc_2_O (2.468 g; 11.31 mmol) and TEA (1.577 mL; 11.31 mmol) were added successively, turning mixture into a solution. Mixture was stirred at room temperature for 1.5 h. Crude mixture was washed with HCl 0.5 N and brine, organic phase dried over anhydrous Na_2_SO_4_, filtered and solvent evaporated under vacuum. Crude residue was purified by flash chromatography in Hex/EtOAc 7:3 as eluent. Final product was a colourless thick oil (1.858 g; 85%). R_f_ 0.6 (Hex/EtOAc 1:1); ^1^H NMR (CDCl_3_, 400 MHz) *δ* 3.67 (s, 3H), 3.10–3.15 (q, *J* = 6.8 Hz, 2H), 2.32–2.35 (t, *J* = 7.4 Hz, 2H), 1.62–1.69 (m, 2H), 1.49–1.55 (m, 2H), 1.44 (s, 9H); ^13^C (CDCl_3_, 100 MHz) *δ* 173.9, 155.9, 79.1, 51.5, 40.1, 33.6, 29.5, 28.4, 22.0; MS (ESI), *m*/*z* calculated for [C_11_H_21_NO_4_ + Na^+^] = 254.1, found 254.1.

### 4.11 5-[(*tert*-Butoxicarbonyl)amino]valeric acid (**15**)

Methyl 5-[(*tert*-butoxicarbonyl)amino]valerate **14** (1.858 g; 8.04 mmol) was dissolved in 40 mL of a THF/H_2_O 4:1 mixture. LiOH (231 mg; 9.65 mmol) was added to the solution. Mixture was allowed to react overnight at room temperature·THF was evaporated under vacuum, H_2_O (8 mL) added to the resulting mixture and acidified to pH = 1–2. Aqueous phase was extracted with EtOAc (3 × 25 mL). Combined organic phases were washed with brine, dried over anhydrous Na_2_SO_4_, filtered and solvent evaporated under vacuum. Final product yielded a white solid (1.648 g; 94%). R_f_ 0.4 (Hex/EtOAc 1:1); ^1^H NMR (MeOD, 400 MHz) *δ* 3.05–3.08 (t, *J* = 6.9 Hz, 2H), 2.31–2.34 (t, *J* = 7.1 Hz, 2H), 1.60–1.67 (m, 2H), 1.50–1.55 (m, 2H), 1.45 (s, 9H); ^13^C NMR (MeOD, 100 MHz) *δ* 176.0, 157.1, 78.4, 39.5, 33.1, 29.0, 27.4, 21.8; MS (ESI), *m*/*z* calculated for [C_10_H_19_NO_4_ + Na^+^] = 240.1; found 240.1.

### 4.12 5-[(*N*-Boc)amino]-*N*-(4-pyridinylmethyl)pentanamide (**16**)

5-[(*tert*-Butoxicarbonyl)amino]valeric acid **15** (499 mg; 2.30 mmol) was dissolved in 7.5 mL of DCM. HATU (1230 g; 3.22 mmol), TEA (930 μL; 6.67 mmol) and 4-picolylamine (280 μL; 2.76 mmol) were added successively in the stated order. Mixture was allowed to react at room temperature for 5 h. Crude mixture was washed with sat. NaHCO_3_ and brine, dried over anhydrous Na_2_SO_4_, filtered and solvent evaporated under vacuum. Product was purified by flash chromatography in a DCM/MeOH 96:4 mixture. Final product yielded a white solid (604 mg; 85%). R_f_ 0.5 (DCM/MeOH 9:1); ^1^H NMR (CDCl_3_, 400 MHz) *δ* 8.42 (s, 2H), 7.09–7.10 (d, *J* = 4.9 Hz, 2H), 6.89 (bs, 1H), 4.80 (bs, 1H), 4.33–4.34 (d, *J* = 6.5 Hz, 2H), 3.01–3.06 (q, *J* = 6.2 Hz, 2H), 2.19–2.23 (t, *J* = 7.5 Hz, 2H), 1.57–1.64 (p, *J* = 7.5 Hz, 2H), 1.39–1.46 (p, *J* = 7.0 Hz, 2H), 1.33 (s, 9H); ^13^C NMR (CDCl3, 100 MHz) *δ* 173.4, 156.3, 149.8, 147.9, 122.3, 79.2, 42.2, 39.6, 35.6, 29.5, 28.4, 22.7; MS (ESI), *m*/*z* calculated for [C_16_H_25_N_3_O_3_+H^+^] = 308.4; found 308.3.

### 4.13 5-(Carbamoylamino)-*N*-(4-pyridinylmethyl)pentanamide (**17**)

5-[(*N*-Boc)amino]-*N*-(4-pyridinylmethyl)pentanamide **16** (360 mg; 1.17 mmol) was dissolved in 1 mL of methanol, to which 7 mL of a 4 M solution of HCl in dioxane was added. Mixture was left to react for 1.5 h at room temperature. Solvent was evaporated under vacuum. Crude mixture was cooled down to 0 °C and treated with concentrated HCl (201 μL; 2.41 mmol). 2.5 mL of hot ethanol at 75 °C was added to the mixture. In a separated flask, KCNO (586 mg; 7.23 mmol) was dissolved in 2.5 mL of water, and this solution added to the mixture containing the precursor, HCl and ethanol. The resulting mixture was warmed to 70 °C and left to react for 1 h. Ethanol was evaporated, the remaining aqueous phase was basified to pH = 10 and freeze dried. The product was then taken up in DCM, filtered and solvent evaporated under vacuum. Final product was a pale solid (217 mg; 74%). R_f_ 0.5 (DCM/MeOH 84:16); ^1^H NMR (DMSO-*d*_6_, 400 MHz) *δ* 8.51–8.52 (d, *J* = 6.2 Hz, 2H), 8.42–8.44 (bs, 1H), 7.25–7.27 (d, *J* = 5.8 Hz, 2H), 5.92–5.94 (bs, 1H), 5.38–5.42 (bs, 4H), 4.29–4.31 (d, *J* = 6.0 Hz, 2H), 2.94–2.99 (m, 2H), 2.17–2.21 (t, *J* = 7.3, 2H), 1.49–1.57 (m, 2H), 1.33–1.40 (m, 2H); ^13^C (DMSO-*d*_6_, 100 MHz) *δ* 172.9, 160.0, 159.2, 149.7, 122.6, 41.5, 35.5, 30.2, 23.2; MS (ESI), *m*/*z* calcd for [C_12_H_18_N_4_O_2_+H^+^] = 251.3, [C_12_H_18_N_4_O_2_ + Na^+^] = 273.3, [C_12_H_18_N_4_O_2_ + K^+^] = 289.3; found 251.2, 273.1, 289.1.

### 4.14 5-(Cyanoamino)-*N*-(4-pyridinylmethyl)pentanamide (**18**)

5-(Carbamoylamino)-*N*-(4-pyridinylmethyl)pentanamide **17** (712 mg; 2.85 mmol) was suspended in 10 mL of pyridine. CH_3_SO_2_Cl (441 μL; 5.69 mmol) was added dropwise to mixture, and left to react for 2 h at r.t.. Pyridine was evaporated, crude residue dissolved in commercial NH_4_OH and washed with DCM. Aqueous phase was evaporated and product purified by flash chromatography using DCM/MeOH 92:8 as eluent mixture. Final product was a waxy yellow solid (330 mg; 50%). R_f_ 0.3 (DCM/MeOH 9:1); ^1^H NMR (MeOD, 400 MHz) *δ* 8.48–8.50 (m, 2H), 7.35–7.37 (m, 2H), 4.44 (s, 2H), 3.04–3.07 (t, *J* = 6.8 Hz, 2H), 2.33–2.37 (t, *J* = 7.0 Hz, 2H), 1.70–1.79 (m, 2H), 1.60–1.68 (m, 2H); ^13^C NMR (MeOD, 100 MHz) *δ* 174.5, 149.5, 148.7, 122.5, 117.3, 44.9, 41.5, 34.9, 29.0, 22.3; MS (ESI) *m*/*z* calculated for [C_12_H_16_N_4_O+H^+^] = 232.1; found 232.1.

### 4.15 5-(*N*″-Hydroxyguanidine)-*N*-(4-pyridinylmethyl)pentanamide (**11b**)

5-(Cyanoamino)-*N*-(4-pyridinylmethyl)pentanamide **18** (325 mg; 1.40 mmol) was dissolved in 6 mL of ethanol. NH_2_OH·HCl (195 mg; 2.80 mmol) and finely ground K_2_CO_3_ (387 mg; 2.80 mmol) were added to the solution, and the mixture stirred for 1 h at r.t. Solvent was evaporated, and crude purified by semipreparative HPLC. Final product was a white solid (293 mg; 79%). ^1^H NMR (D_2_O, 400 MHz) *δ* 8.80–8.82 (m, 2H), 7.98–7.99 (m, 2H), 4.68 (s, 2H), 3.24–3.27 (t, *J* = 7.0, 2H), 2.41–2.44 (t, *J* = 7.0, 2H), 1.62–1.77 (m, 4H); ^13^C (MeOD, 400 MHz) *δ* 175.0, 161.1, 159.1, 141.2, 125.1, 42.2, 40.5, 34.6, 28.0, 22.2; MS (ESI), *m*/*z* calcd for [C_12_H_18_N_4_O_2_+H^+^] = 266.1539; found 266.1603.

### 4.16 (2*S*)-2-(Acetylamino)-5-(carbamoylamino)-*N*-(pyridin-4-ylmethyl)pentanamide (**19**)

*tert*-Butyl-*N*-[(1*S*)-4-(carbamoylamino)-1-[(pyridine-4-ylmethyl)carbamoyl]butyl]carbamate **8** (776 mg; 2.12 mmol) was dissolved in 15 mL of a mixture 2:1 dioxane/MeOH containing 2.66 M of HCl. Mixture was left to react at r.t. for 1 h. Solvents were evaporated under vacuum. Crude was dissolved in 12 mL of H_2_O and pH adjusted to pH = 9–10 using an 8 M solution of NaOH. Mixture was cooled to 0 °C and Ac_2_O (401 μL; 4.24 mmol) was added dropwise. pH was adjusted again to 9–10, and mixture left to react at r.t. for 3 h. Water was evaporated, and crude residue purified by flash chromatography using DCM/MeOH 82:18 as eluent. Final product was a white solid (386 mg; 59%). R_f_ 0.2 (DCM/MeOH 82:18); ^1^H NMR (MeOD, 400 MHz) *δ* 8.47–8.48 (m, 2H), 7.37–7.38 (d, *J* = 5.6 Hz, 2H), 4.48–4.52 (d, *J* = 16.5 Hz, 1H), 4.40–4.44 (d, *J* = 16.5 Hz, 1H),4.34–4.37 (dd, *J* = 8.8 Hz, 5.4 Hz, 1H), 3.10–3.21 (m, 2H), 2.03 (s, 3H), 1.82–1.90 (m, 1H), 1.51–1.76 (m, 3H); ^13^C NMR (MeOD, 100 MHz) *δ* 173.6, 172.3, 160.9, 149.6, 148.5, 122.4, 53.6, 41.5, 38.9, 28.8, 26.5, 21.0; MS (ESI), *m*/*z* calculated for [C_14_H_21_N_5_O_3_+H^+^] = 308.2, found 308.1; [α]_D_^20^ in MeOH = +3.0 (c = 1.0).

### 4.17 (2*S*)-5-(Cyanoamino)-2-acetamido-*N*-(pyridin-4-ylmethyl)pentanamide (**20**)

(2*S*)-2-(Acetylamino)-5-(carbamoylamino)-*N*-(pyridin-4-ylmethyl)pentanamide **19** (60 mg; 0.20 mmol) was dissolved in 1 mL of pyridine and warmed up to 40 °C in order to facilitate dilution. CH_3_SO_2_Cl (30 μL; 0.39 mmol) was added dropwise, and mixture left to react at 40 °C for 2 h. Pyridine was evaporated and crude residue purified by flash chromatography using a mixture of DCM/MeOH 92:8 as eluent. Final product was a yellow oil (21 mg; 36%). R_f_ 0.6(DCM/MeOH 85:15); ^1^H NMR (MeOD, 400 MHz) *δ* 8.49–8.48 (m, 2H), 7.39–7.38 (m, 2H), 4.53–4.48 (d, *J* = 16.3 Hz, 1H), 4.45–4.41 (d, *J* = 16.3 Hz, 1H), 4.39–4.36 (m, 1H), 3.08–3.05 (t, *J* = 6.7 Hz, 2H), 2.04 (s, 3H), 1.95–1.88 (m, 1H), 1.79–1.63 (m, 3H); ^13^C (MeOD, 100 MHz) *δ* 173.2, 172.2, 149.5, 148.6, 122.4, 117.2, 53.3, 44.7, 41.5, 28.4, 26.0, 21.0; MS (ESI), *m*/*z* calculated for [C_14_H_19_N_5_O_2_+H^+^] = 290.1, found 290.1; [α]_D_^20^ in MeOH = +5.0 (c = 1.0).

### 4.18 (2*S*)-2-Acetamido-5-(2-hydroxycarbamimidamido)-*N*-(pyridin-4-ylmethyl)pentanamide (**11c**)

(2*S*)-5-(Cyanoamino)-2-acetamido-*N*-(pyridine-4-ylmethyl)pentanamide **20** (21 mg; 0.07 mmol) was dissolved in 0.5 mL of EtOH. NH_2_OH·HCl (10 mg; 0.14 mmol) and TEA (10 μL; 0.07 mmol) were added to the solution and mixture stirred for 1 h at r.t. Solvent was evaporated and mixture purified by HPLC. Final product was a white solid (17 mg; 75%). ^1^H NMR (MeOD, 400 MHz) *δ* 8.80 (m, 2H), 8.02–8.01 (m, 2H), 4.76–4.72 (d, *J* = 17.7 Hz, 1H), 4.67–4.63 (d, *J* = 17.7 Hz, 1H), 4.35–4.32 (dd, *J* = 8.0 Hz, 5.2 Hz, 1H), 3.29–3.26 (t, *J* = 7.0 Hz, 3H), 2.06 (s, 3H), 1.97–1.88 (m, 1H), 1.83–1.65 (m, 3H); ^13^C NMR (MeOD, 100 MHz) *δ* 173.8, 172.7, 161.0, 159.1, 141.1, 125.0, 53.7, 42.1, 40.3, 28.1, 25.1, 21.0; MS (ESI), *m*/*z* calculated for [C_14_H_22_N_6_O_3_+H^+^] = 323.1753, found = 323.1806; [α]_D_^20^ in MeOH = +4.0 (c = 1.0).

### 4.19 (2*S*)-2-Amino-5-(carbamoylamino)-*N*-(pyridin-4-ylmethyl)pentanamide (**21a**)

*tert*-Butyl-*N*-[(1*S*)-4-(carbamoylamino)-1-[(pyridine-4-ylmethyl)carbamoyl]butyl]carbamate **8** (271 mg; 0.74 mmol) was dissolved in 2 mL of TFA. Mixture was left to react at r.t. for 1 h. Solvent was evaporated under vacuum, and product triturated in Et_2_O. Final product yielded a white solid (278 mg; quant.). ^1^H NMR (D_2_O, 400 MHz) *δ* 8.65–8.66 (m, 2H), 7.87–7.88 (m, 2H), 4.67 (s, 2H), 4.07–4.10 (t, *J* = 6.31 Hz, 1H), 3.05–3.08 (t, *J* = 6.8 Hz, 2H), 1.84–1.92 (m, 2H), 1.45–1.52 (m, 2H); ^13^C NMR (D_2_O,100 MHz) *δ* 170.4, 162.1–163.2 (q, *J* = 33.6 Hz, TFA), 161.3, 159.4, 141.0, 125.2, 111.8–120.5 (q, *J* = 290.2 Hz, TFA), 52.9, 42.5, 38.9, 28.1, 24.7; MS (ESI) *m*/*z* calculated for [C_12_H_19_N_5_O_2_+H^+^] = 266.1539, found 266.1608; [α]_D_^20^ in H_2_O = +10.0 (c = 1.0).

### 4.20 *tert*-Butyl *N*-[(1*S*)-4-(carbamoylamino)-1-[(pyridine-3-ylmethyl)carbamoyl]butyl]carbamate (**22**)

To a solution of the amino acid Boc-l-Cit-OH **7** (250 mg; 0.91 mmol) in DCM (5 mL) was added, in the stated order, HATU (483 mg; 1.27 mmol), TEA (368 μL; 2.64 mmol) and 3-picolylamine (111 μL; 1.09 mmol). This mixture was stirred at r.t. for 5 h. Solvent was evaporated, and the remaining residue was purified by flash chromatography using a DCM/MeOH 9:1 mixture as solvent. Final product was obtained as white solid (276 mg; 83%). R_f_ 0.25 (DCM/MeOH 9:1); ^1^H NMR (CDCl_3_, 400 MHz) *δ* 8.41–4.86 (m, 2H), 7.55–7.57 (m, 1H), 7.51 (bs, 1H), 7.16–7.18 (m, 1H), 5.42–5.43 (bs, 1H), 5.13 (bs, 1H), 4.53 (bs, 1H), 4.42–4.47 (dd, *J* = 15.1, 6.2 Hz, 1H), 4.28–4.33 (dd, *J* = 15.5, 5.8 Hz, 1H), 4.24 (bs, 1H),3.30–3.31 (m, 1H), 3.01–3.06 (m, 2H), 1.69–1.76 (m, 1H), 1.39–1.58 (m, 3H), 1.34 (s, 9H); ^13^C NMR (CDCl_3_, 100 MHz) *δ* 173.2, 160.2, 156.1, 148.6, 148.2, 135.6, 134.4, 123.6, 79.8, 54.0, 40.7, 39.2, 30.0, 28.3, 26.5; MS (ESI) *m*/*z* calculated for [C_17_H_27_N_5_O_4_+H^+^] = 366.4, found 366.5; [α]_D_^20^ in CHCl_3_ = -4.0 (c = 1.0)

### 4.21 (2*S*)-2-Amino-5-(carbamoylamino)-*N*-(pyridin-3-ylmethyl)pentanamide (**21b**)

*tert*-Butyl *N*-[(1*S*)-4-(carbamoylamino)-1-[(pyridine-3-ylmethyl)carbamoyl]butyl]carbamate **22** (270 mg; 0.74 mmol) was dissolved in 2 mL of TFA. Mixture was left to react at r.t. for 1 h. Solvent was evaporated under vacuum, and product triturated in Et_2_O. Final product yielded a white solid (261 mg; 93%). ^1^H NMR (D_2_O, 400 MHz) *δ* 8.64–8.67 (m, 2H), 8.45–8.47 (m, 1H), 7.97–8.00 (dd, *J* = 7.9, 6.0 Hz, 1H), 4.57 (s, 2H), 3.98–4.01 (t, *J* = 6.6 Hz, 2H), 3.00–3.04 (t, *J* = 6.7 Hz), 1.79–1.85 (m, 2H), 1.38–1.45 (m, 2H), ^13^C NMR (D_2_O, 100 MHz) *δ* 170.1, 162.1–163.2 (q, *J* = 36.5 Hz, TFA), 161.3, 146.0, 140.2, 139.9, 138.3, 127.4, 111.8–120.5 (q, *J* = 284.6 Hz, TFA), 52.8, 40.0, 38.9, 28.0, 24.7; MS (ESI) *m*/*z* calculated for [C_12_H_19_N_5_O_2_+H^+^] = 266.1539, found 266.1612; [α]_D_^20^ in H_2_O = +13.0 (c = 1.0).

### 4.22 *N*-[(1*S*)-4-(Carbamoylamino)-1-[(pyridin-4-yl)carbamoyl]butyl]carbamate (**23**)

To a solution of the amino acid Boc-l-Cit-OH **7** (150 mg; 0.54 mmol) in DCM (2.5 mL) was added, in the stated order, HATU (290 mg; 0.76 mmol), TEA (218 μL; 1.57 mmol) and 4-aminopyridine (61 mg; 0.65 mmol). This mixture was stirred at r.t. for 5 h. Solvent was evaporated, and the remaining residue was purified by flash chromatography using a DCM/MeOH 88:12 mixture as solvent. Final product was obtained as white solid (140 mg; 74%). R_f_ 0.4 (DCM/MeOH 85:15); ^1^H NMR (MeOD, 400 MHz) *δ* 8.40–8.41 (m, 2H), 7.69–7.70 (m, 2H), 4.24–4.27 (m, 1H), 3.21–3.27 (m, 1H), 3.08–3.15 (m, 1H), 1.82–1.85 (m, 1H), 1.56–1.74 (m, 3H), 1.46 (bs, 9H); ^13^C NMR (MeOD, 100 MHz) *δ* 173.2, 161.0, 156.7, 149.3, 146.6, 113.8, 79.3, 55.0, 38.7, 29.1, 27.3, 26.5; MS (ESI) *m*/*z* calculated for [C_16_H_25_N_5_O_4_+H^+^] = 352.4, found 352.5; [α]_D_^20^ in CHCl_3_ = -5.0 (c = 1.0).

### 4.23 (2*S*)-2-Amino-5-(carbamoylamino)-*N*-(pyridin-4-yl)pentanamide (**21c**)

*N*-[(1*S*)-4-(Carbamoylamino)-1-[(pyridine-4-yl)carbamoyl]butyl]carbamate **23** (125 mg; 0.36 mmol) was dissolved in 2.5 mL of a mixture 2:1 dioxane/methanol containing 2.66 M of HCl. Mixture was left to react at r.t. for 1 h. Solvents were evaporated under vacuum. Final product was yielded as a white solid (102 mg; quant.). ^1^H NMR (D_2_O, 400 MHz) *δ* 8.49–8.50 (d, *J* = 6.9 Hz, 2H), 8.01–8.03 (d, *J* = 6.9 Hz, 2H), 4.21–4.24 (m, 1H), 3.01–3.05 (t, *J* = 6.6 Hz, 2H), 1.88–1.98 (m, 2H), 1.50–1.53 (m, 2H); ^13^C NMR (D_2_O, 100 MHz) *δ* 169.8, 161.2, 152.4, 141.9, 115.8, 54.0, 38.9, 27.7, 24.4; MS (ESI) *m*/*z* calculated for [C_11_H_17_N_5_O_2_+H^+^] = 252.1382, found 252.1454; [α]_D_^20^ in H_2_O = +16.0 (c = 1.0).

### 4.24 Hyperpolarisation and NMR experiments

*para*-hydrogen was produced by cooling H_2_ gas over a spin-exchange catalyst (Fe_2_O_3_) at 30 K temperature. This method was able to provide *para*-hydrogen with more than 93% purity. Samples involved in the analysis were prepared with ∼ 6 mg of ligand (∼60 μM) and 2 mg of IMes precursor catalyst (3 μM) dissolved in 0.6 ml deuterated methanol solvent in a 5 mm NMR tube fitted with a J. Young’s tap. The resulting solutions were then degassed by 3 cycles of freeze-pumpthaw method before filling the tube with pH_2_ at a pressure of 3 bar. Once filled with pH_2_, the tubes were shaken vigorously for ∼10 s in a fringe field of ∼65 Gauss around a 9.4 T Bruker spectrometer or Earth’s magnetic field. Immediately after that, tubes were rapidly transported inside the spectrometer for subsequent NMR detections. Enhancement factor was calculated by taking the ratio of the integrals of peaks in the hyperpolarised spectra and thermal equilibrium spectra. Catalyst precursor was synthesised in our laboratory according to a literature procedure^1^ [IMes = 1,3-bis(2,4,6-trimethylphenyl) imidazole-2-ylidene and COD = *cis*,*cis*-1,5-cyclooctadiene]. *T*_1_ measurement experiments were performed using 8 mg of compound in the form of HCl salt (same amount used in the SABRE hyperpolarisation experiments) dissolved in 0.6 mL of PBS buffer pH = 7.4 and 37 °C.

### 4.25 Enzymatic tests

eNOS (bovine recombinant) was purchased from Cayman chemicals and used as received. 1 U of enzyme produced 1 nM/min of NO at 37 °C in 50 mM HEPES buffer (pH 7.4) with 1 mM CaCl_2_, 20 µg/ml CaM, 0.1 mM NADPH, 50 µM l-arginine and 12 µM tetrahydrobiopterin. The amount of enzyme in 1 U was calculated from the batch specific activity as shown on the data sheet. l-Arginine analogues **4a**–**i** and **12a**–**c** were tested using 1 U of enzyme in the stated conditions against an l-arginine positive control and a negative control solution in a total reaction volume of 100 μL. The reaction was optimised to 40 min. 3 μL of an ice-cold solution containing 20 mM HEPES (pH 5.5), 2 mM EDTA and 2 mM EGTA were added to stop the reaction. The concentration of NO was determined using the colorimetric Griess reaction by following the nitrate/nitrite colorimetric assay kit LDH method provided by Cayman chemicals.

## Figures and Tables

**Table 1:** NO_2_^−^ concentration produced by l-arginine, **4a**–**j** and **11a**–**c** after colorimetric LDH assay on eNOS.

Compound	l-arginine	**4a**	**4b**	**4c**	**4d**	**4e**	**4f**	**4g**	**4h**	**4i**	**4j**	**11a**	**11b**	**11c**
[NO_2_^−^] (μM)	25	48	20	21	N/A^a^	22	22	26	23	N/A^a^	38	26	29	27

a No enzymatic activity test was performed on **4d** and **4i**.

**Table 2:** Total NMR signal enhancements achieved for compounds **4a**–**j** and **12a**–**c**.^a^

	Without acetonitrile-*d*_3_		With acetonitrile-*d*_3_	
Magnetic field	Earth’s MF	65 G	Earth’s MF	65 G
**4a**	209	95	268	102
**4b**	26^b^	50^b^	162	147
**4c**	15	10	8	9
**4d**	<1	<1	<1	<1
**4e**	870	297	322	261
**4f**	112	229	270	314
**4g**	36	23	43	26
**4h**	38	37	43	45
**4i**	<1	<1	<1	<1
**4j**	<1	<1	<1	<1
**11a**	<1	<1	<1	<1
**11b**	51^c^	5	19^c^	N/A^d^
**11c**	<1	<1	<1	<1

a Compounds were hyperpolarized in methanol-*d*_4_, with a parahydrogen pressure = 3 bar, 10 s shaking time, [Ir(COD)(IMes)Cl] as catalyst precursor and concentrations of ligand and catalyst precursor of 40 mM and 5 mM respectively. Experiments were done with and without 60 mM of acetonitrile-*d*_3_ as co-ligand and at Earth’s MF and 65 G.

b Sample was partially deuterated.

c Experiments performed at 50 °C.

d No experiment was performed under these conditions.

## A Supplementary data

Supplementary data 1 NMR spectra and hyperpolarized NMR spectra samples.
