# Supplementary material for: Synthesis and hyperpolarisation of eNOS substrates for quantification of NO production by 1H NMR spectroscopy
Source: Bioorg Med Chem. 2017 May 15;25(10):2730–42. doi: 10.1016/j.bmc.2017.03.041 (PMC5399308; doi:10.1016/j.bmc.2017.03.041)
Supplement: Supplementary data 1 — NMR spectra and hyperpolarized NMR spectra samples. [file mmc1.docx]

**SUPPORTING INFORMATION**

Synthesis and hyperpolarisation of eNOS substrates for quantification of NO production by ^1^H-NMR spectroscopy

Fernando Fernandez Diaz-Rullo, Francesco Zamberlan, Ryan E. Mewis, Marianna Fekete, Lionel Broche, Lesley A. Cheyne, Sergio Dall’Angelo, Simon B. Duckett, Dana Dawson*, Matteo Zanda*

**Compound 2d.** ^1^H NMR, D_2_O, 400 MHz

**
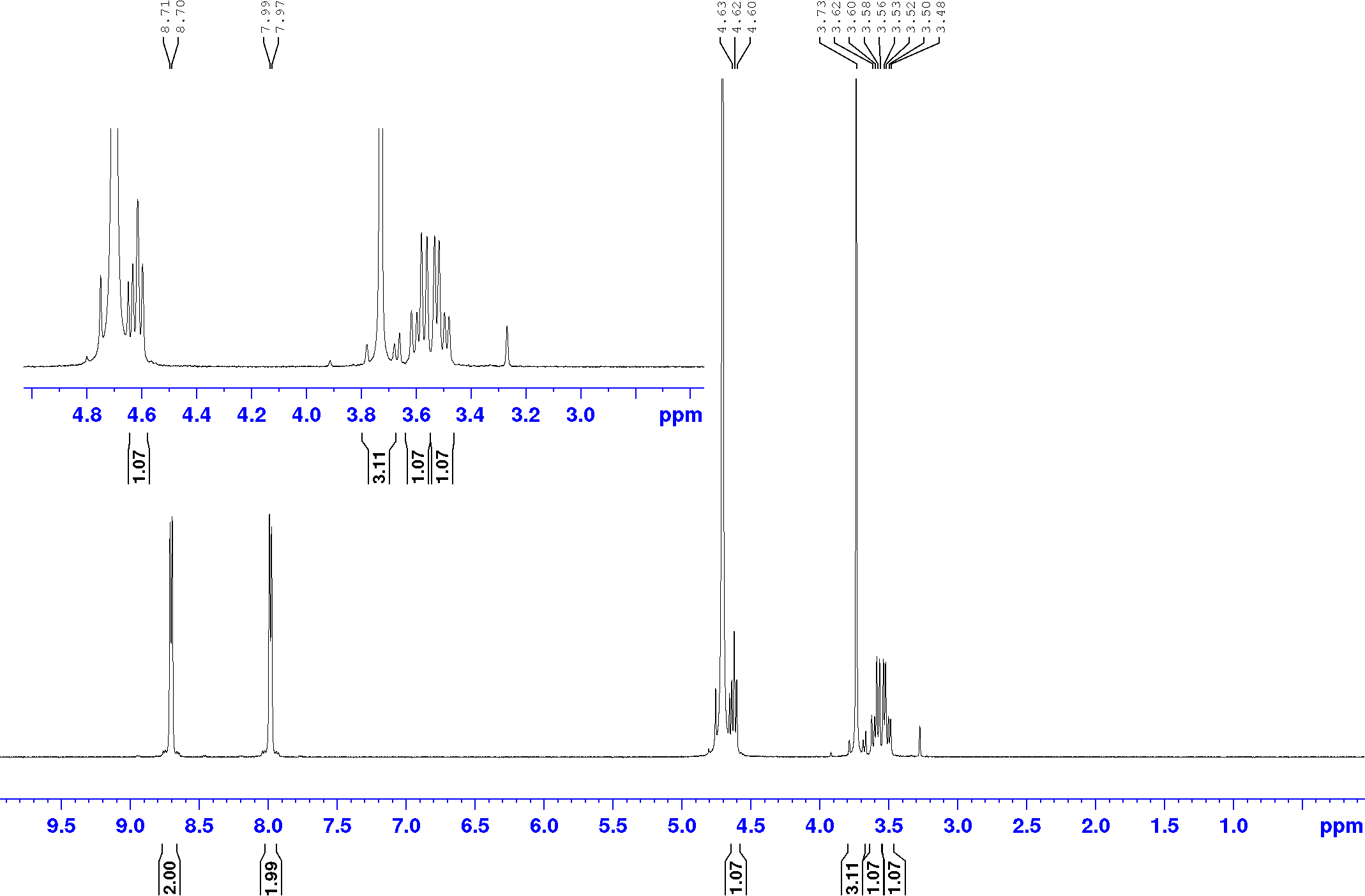
**

**Compound 2d.** ^13^C NMR, D_2_O, 100 MHz

**
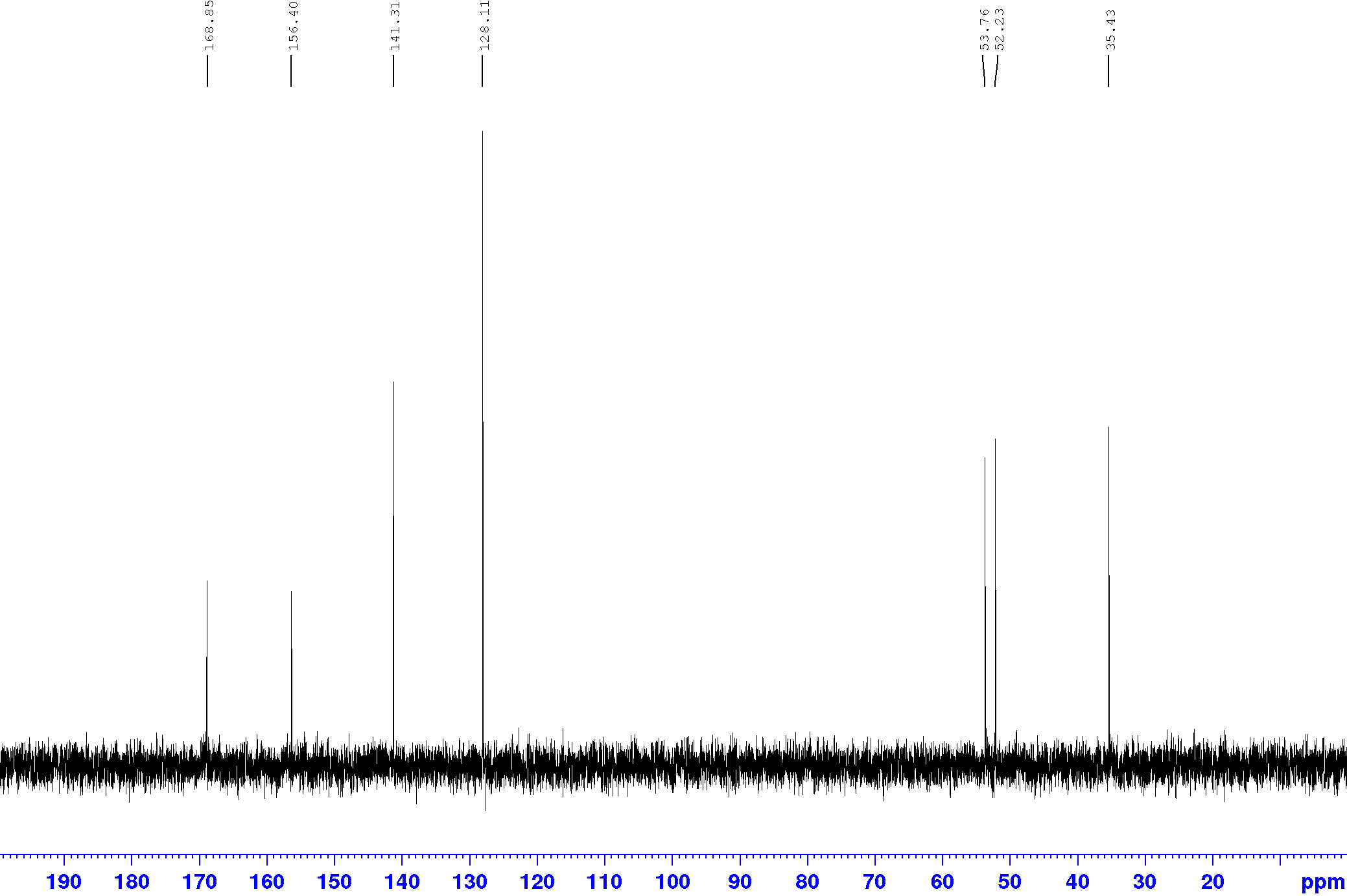
**

**Compound 2i.** ^1^H NMR, D_2_O, 400 MHz

**
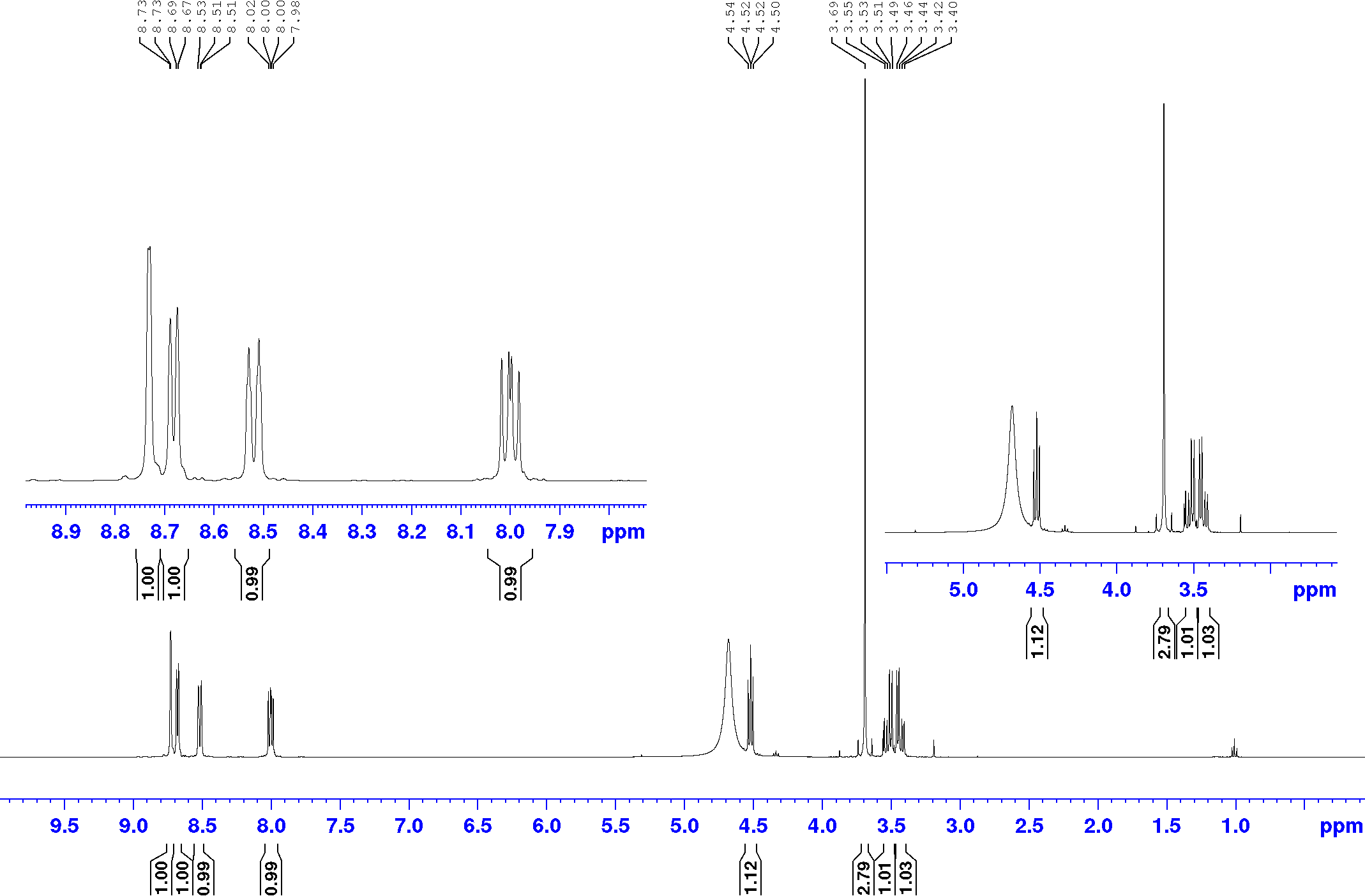
**

**Compound 2i.** ^13^C NMR, D_2_O, 100 MHz

**
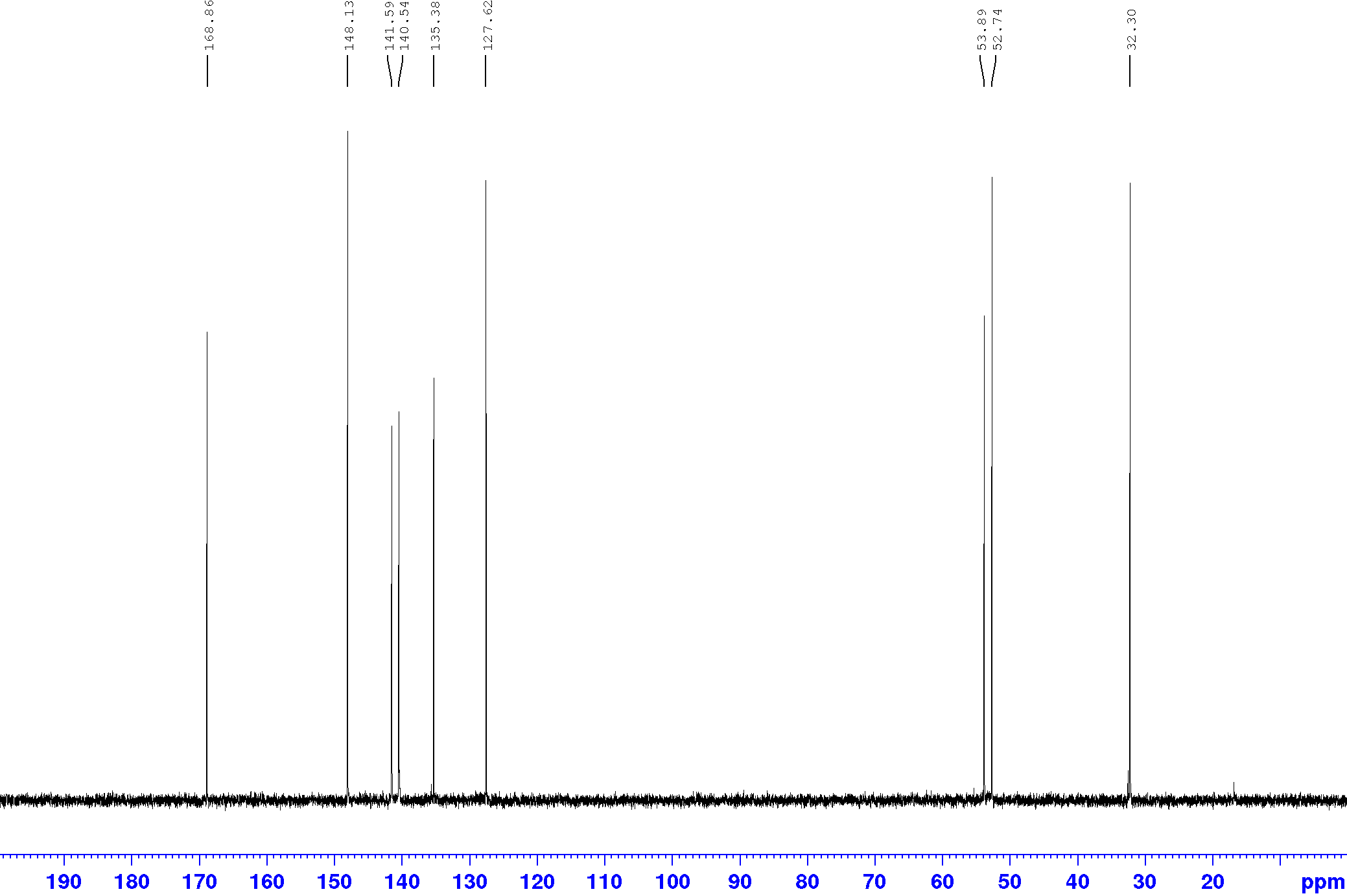
**

**Compound 3a.** ^1^H NMR, CDCl_3_, 400 MHz

**
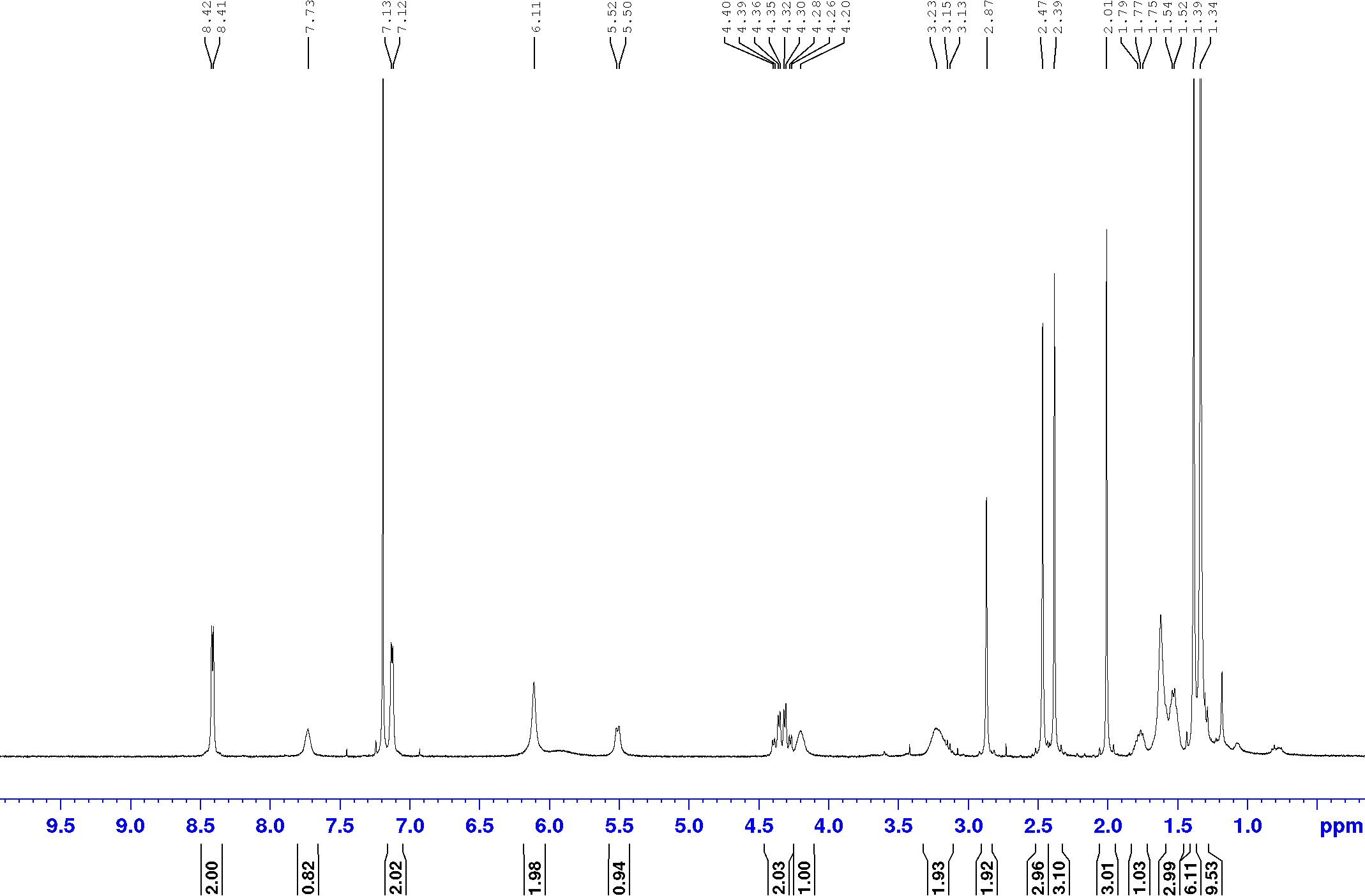
**

**Compound 3a.** ^13^C NMR, CDCl_3_, 100 MHz

**
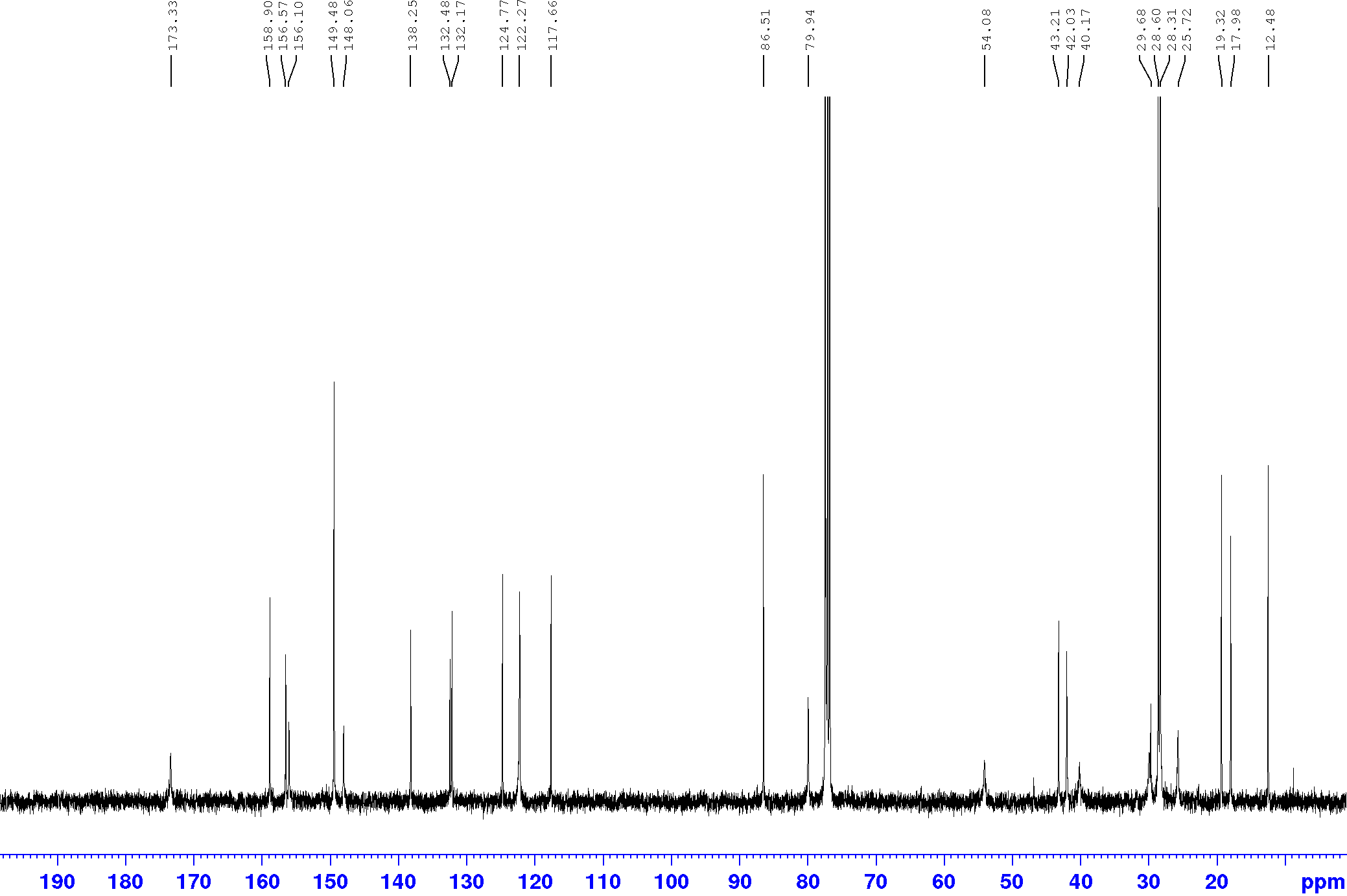
**

**Compound 3b.** ^1^H NMR, CDCl_3_, 400 MHz

**
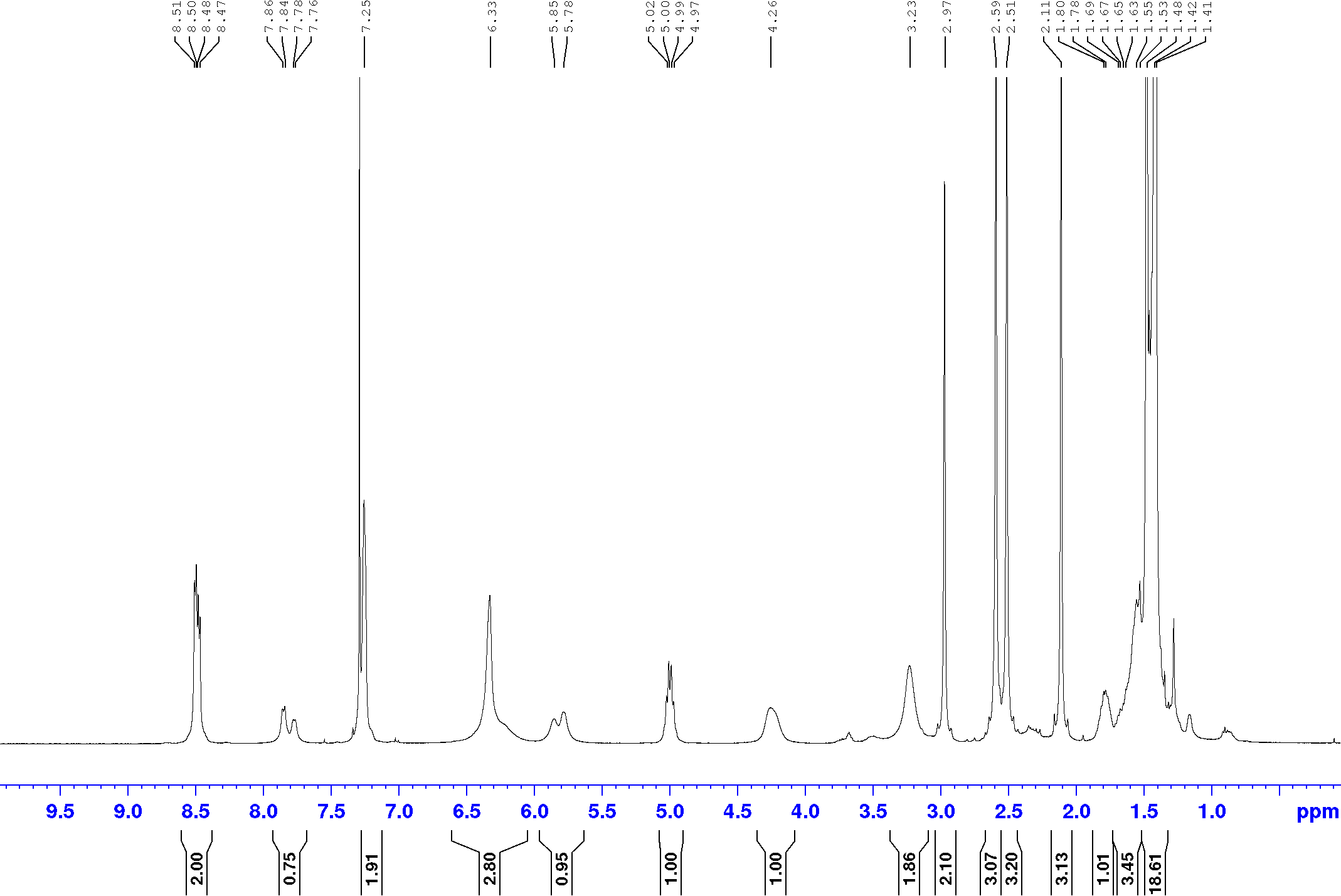
**

**Compound 3b.** ^13^C NMR, CDCl_3_, 100 MHz

**
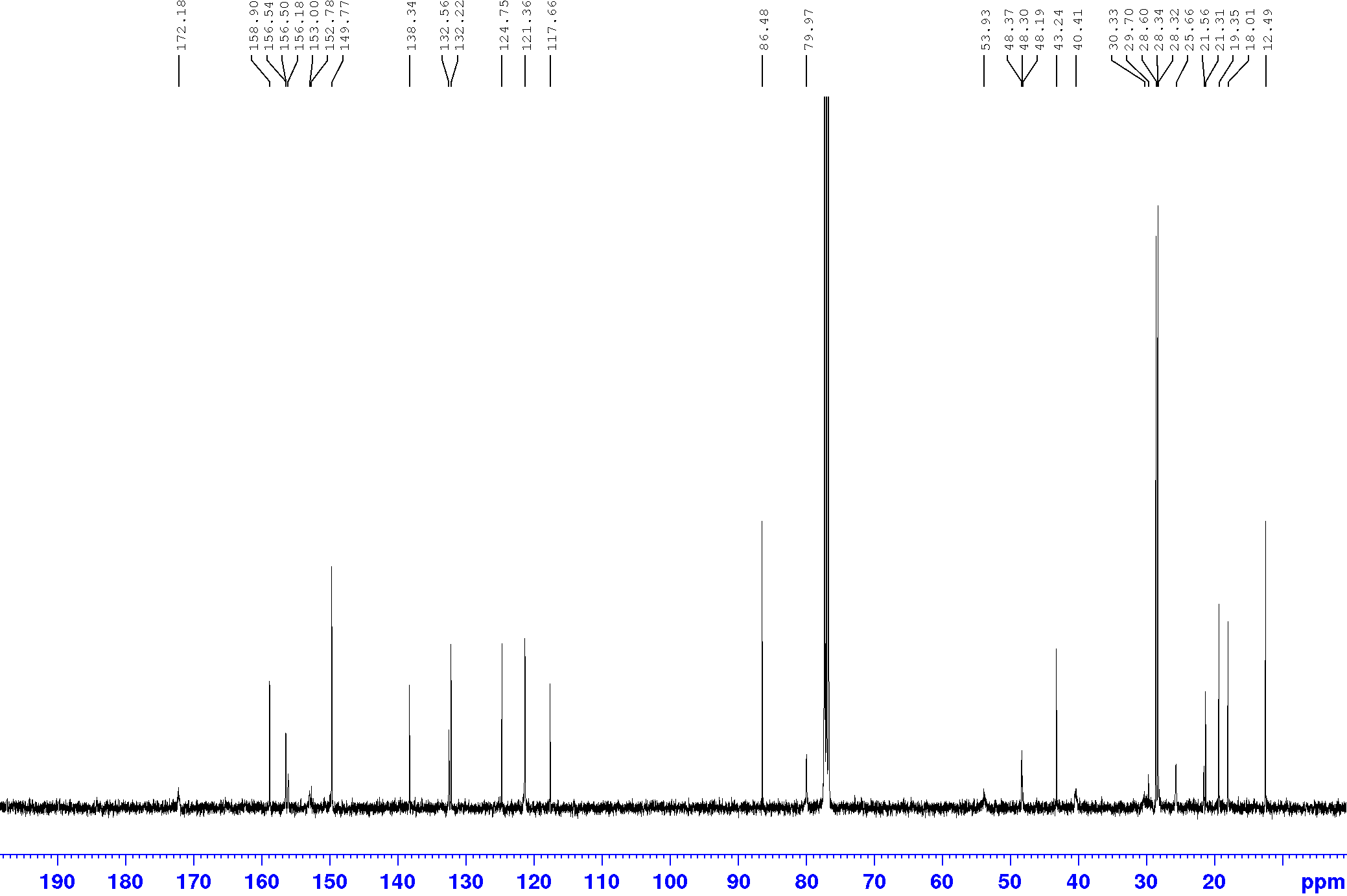
**

**Compound 3c.** ^1^H NMR, CDCl_3_, 400 MHz

**
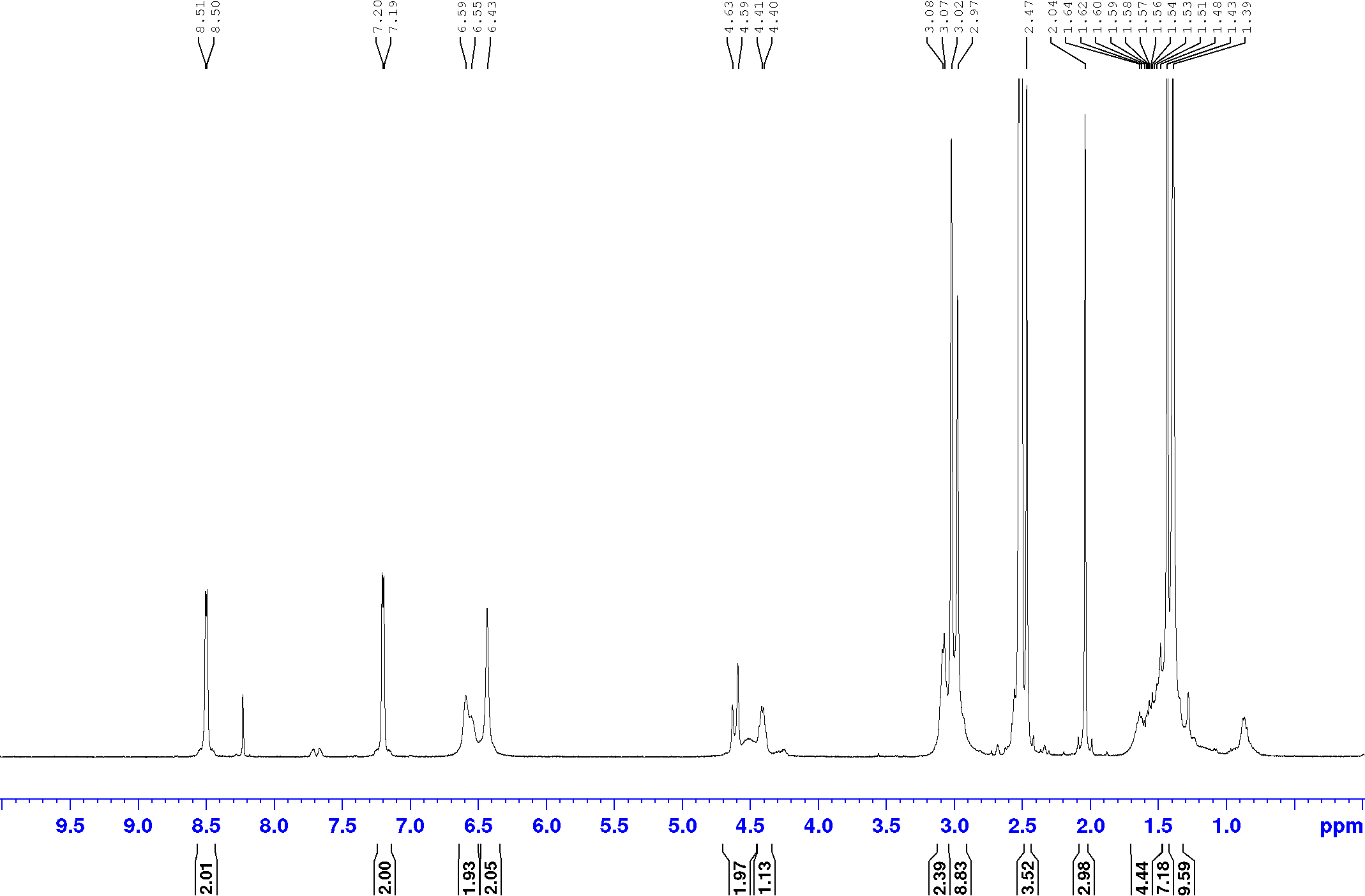
**

**Compound 3c.** ^13^C NMR, CDCl_3_, 100 MHz

**
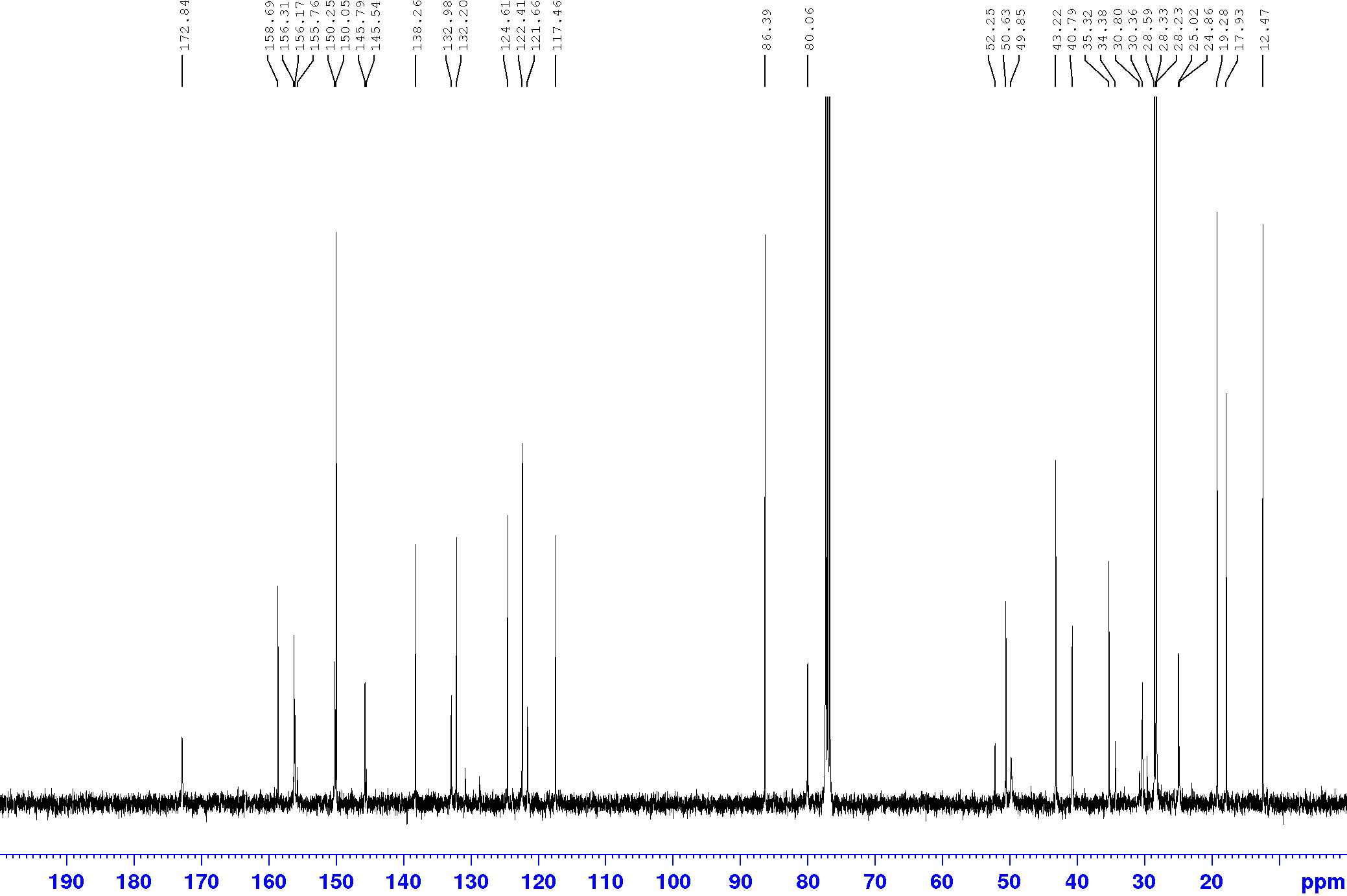
**

**Compound 3d.** ^1^H NMR, CDCl_3_, 400 MHz

**
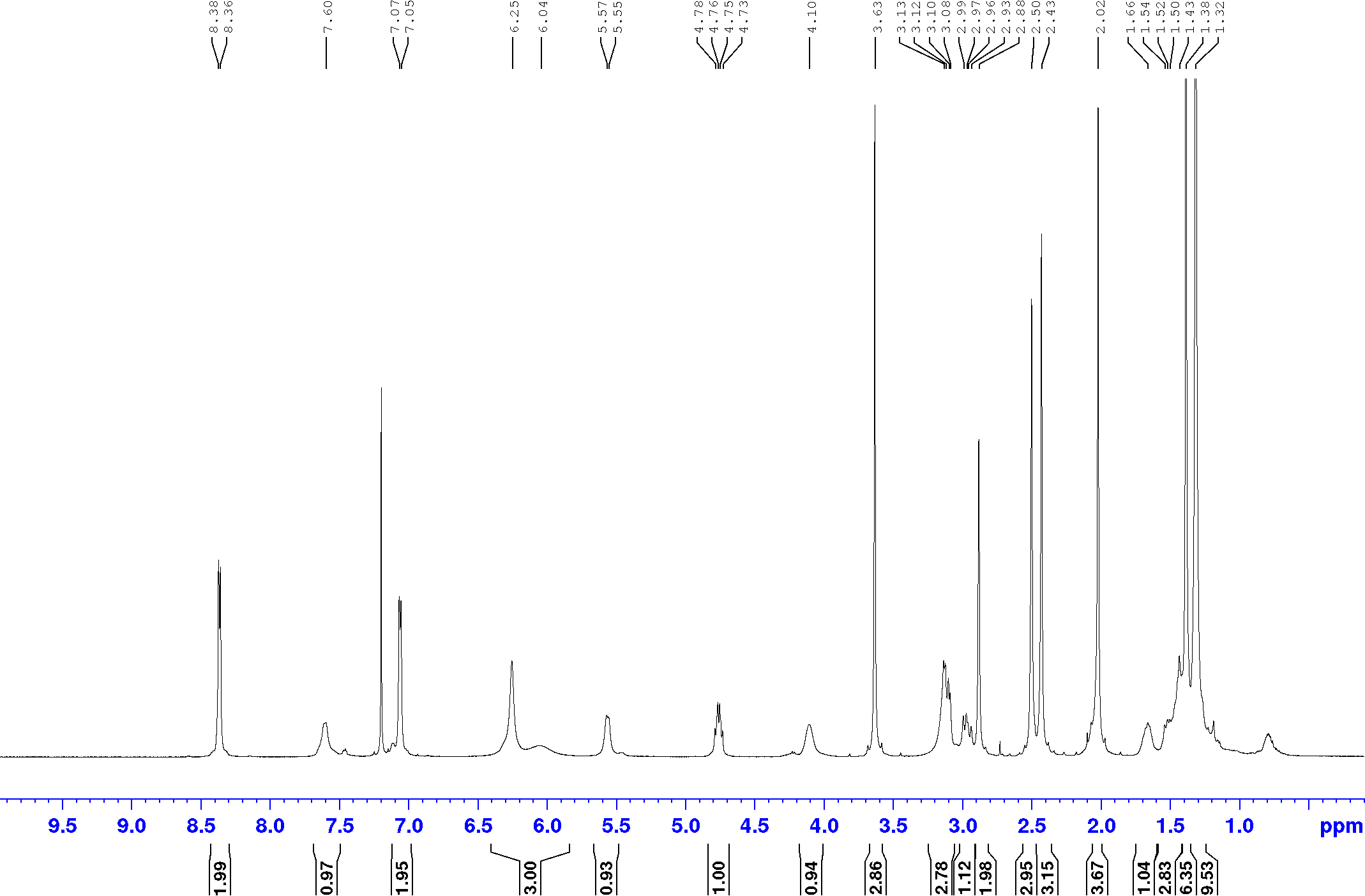
**

**Compound 3d.** ^13^C NMR, CDCl_3_, 100 MHz

**
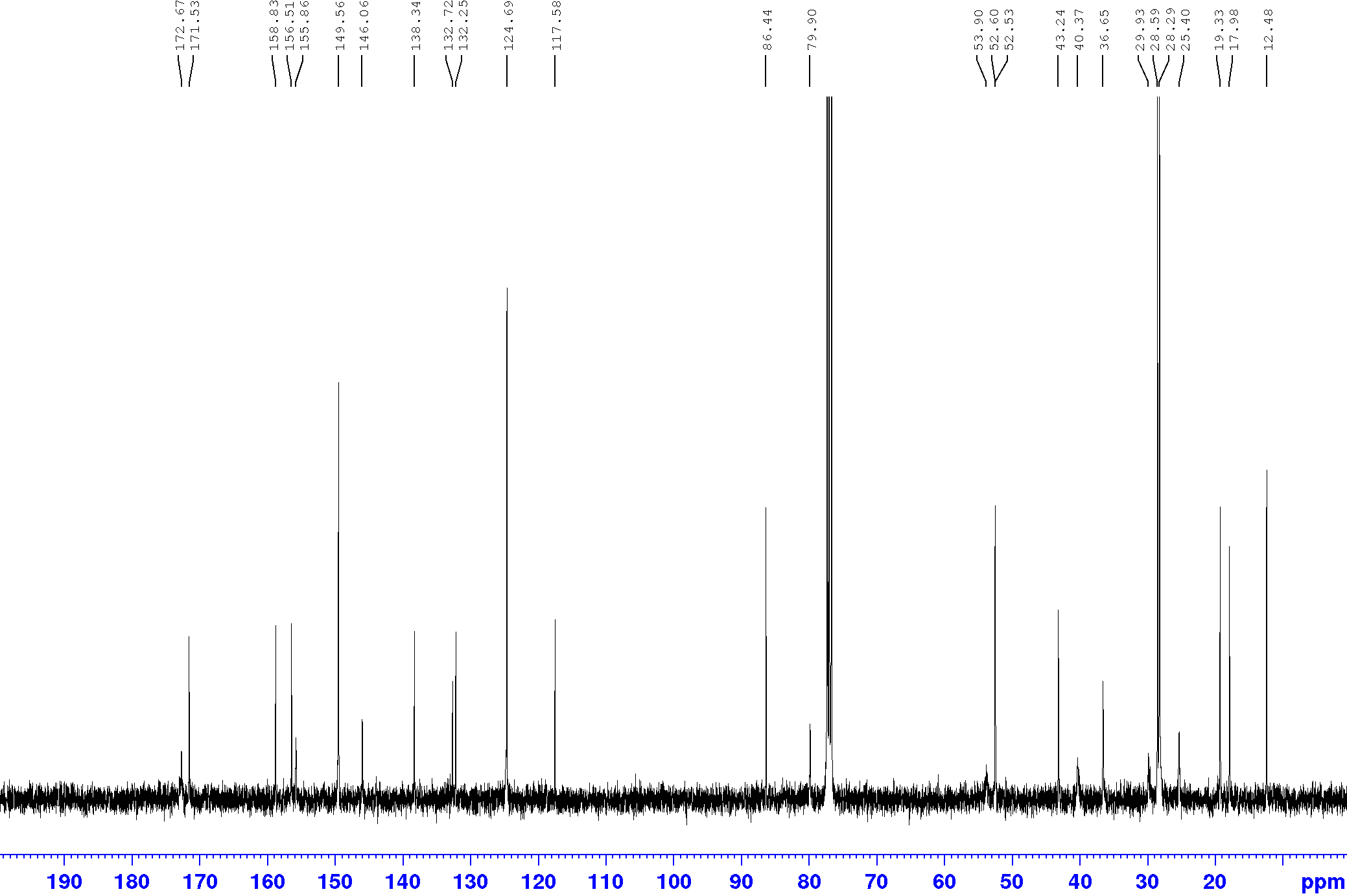
**

**Compound 3e.** ^1^H NMR, CDCl_3_, 400 MHz

**
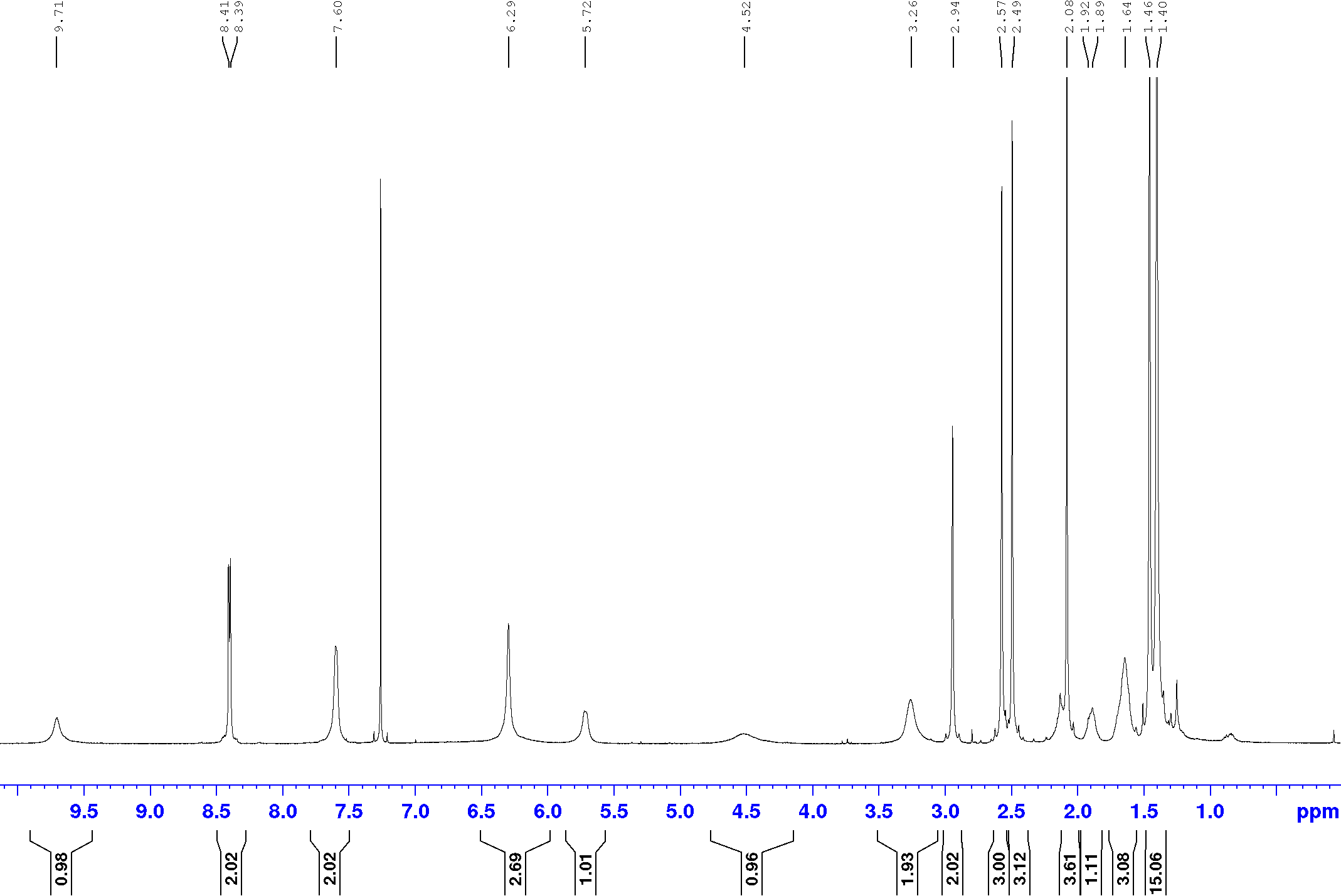
**

**Compound 3e.** ^13^C NMR, CDCl_3_, 100 MHz

**
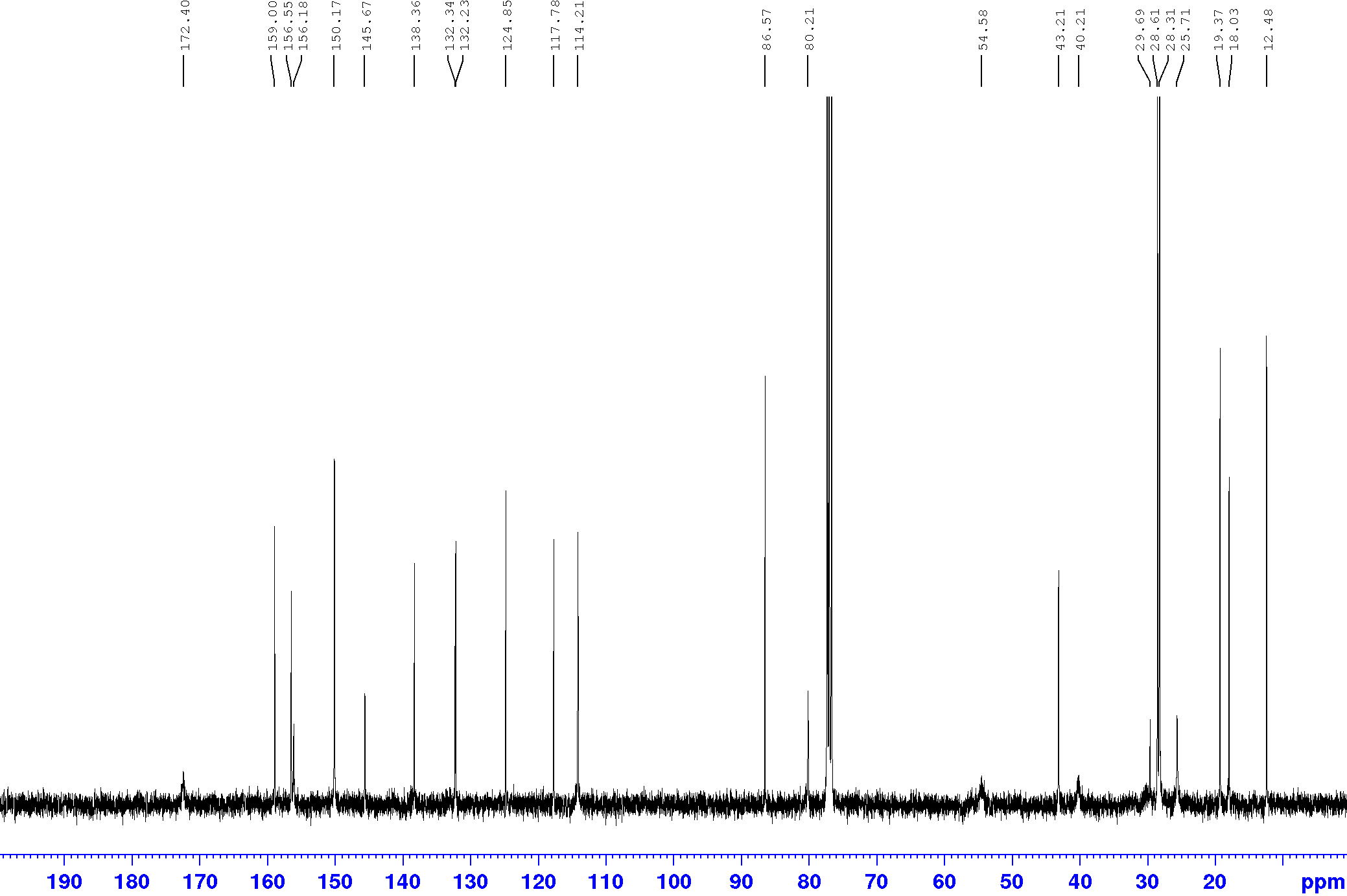
**

**Compound 3f.** ^1^H NMR, CDCl_3_, 400 MHz

**
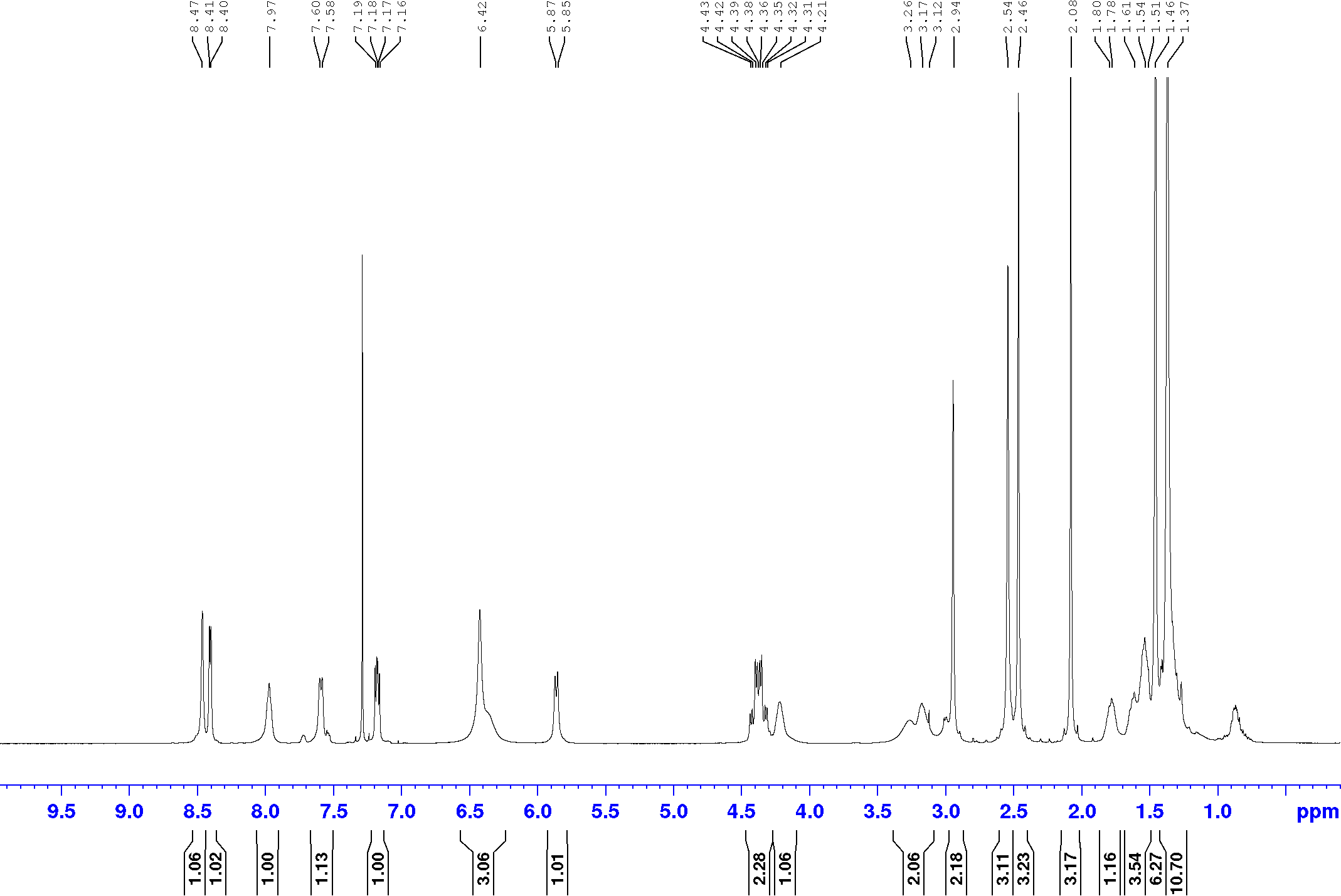
**

**Compound 3f.** ^13^C NMR, CDCl_3_, 100 MHz

**
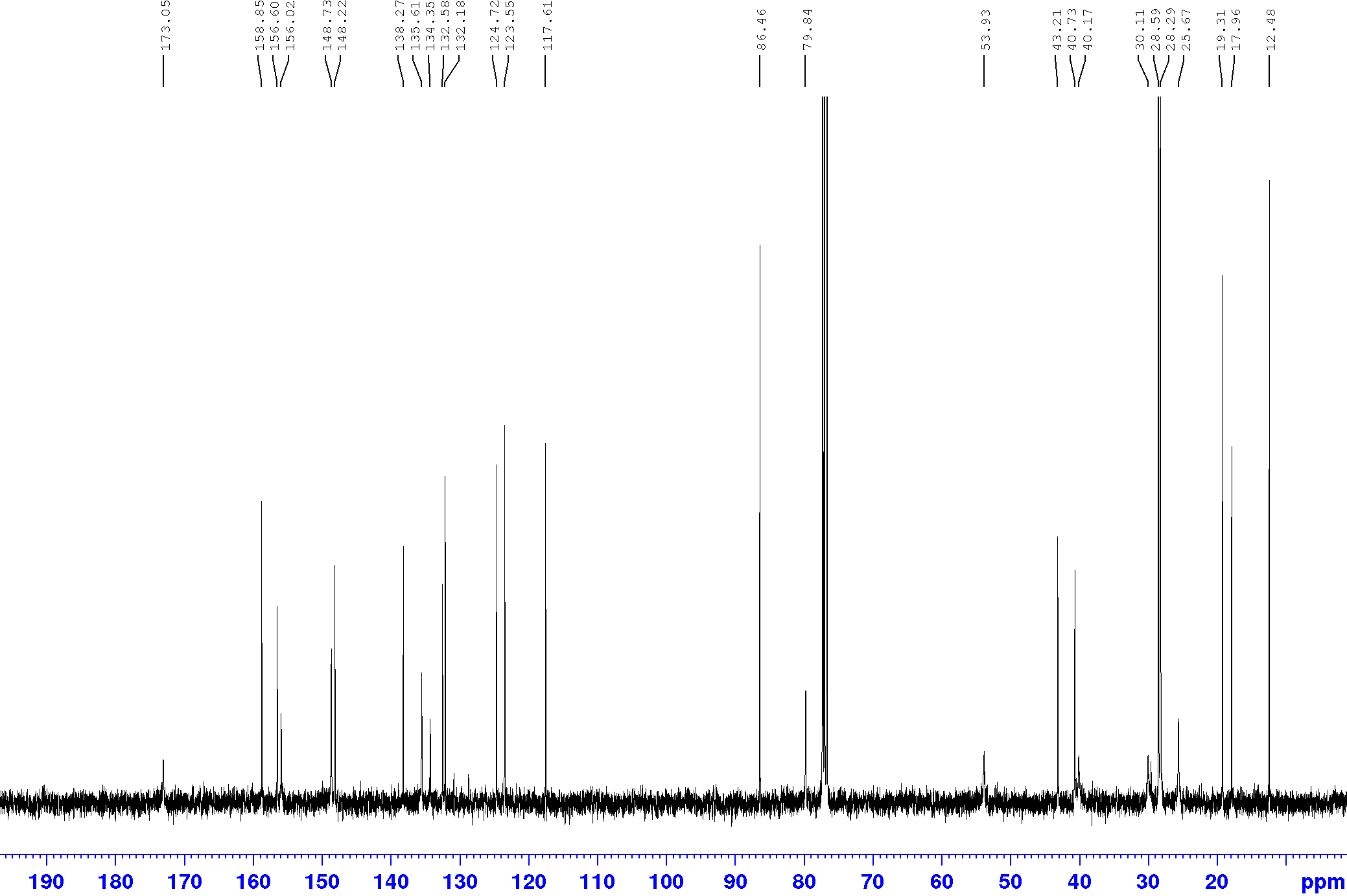
**

**Compound 3g.** ^1^H NMR, CDCl_3_, 400 MHz

**
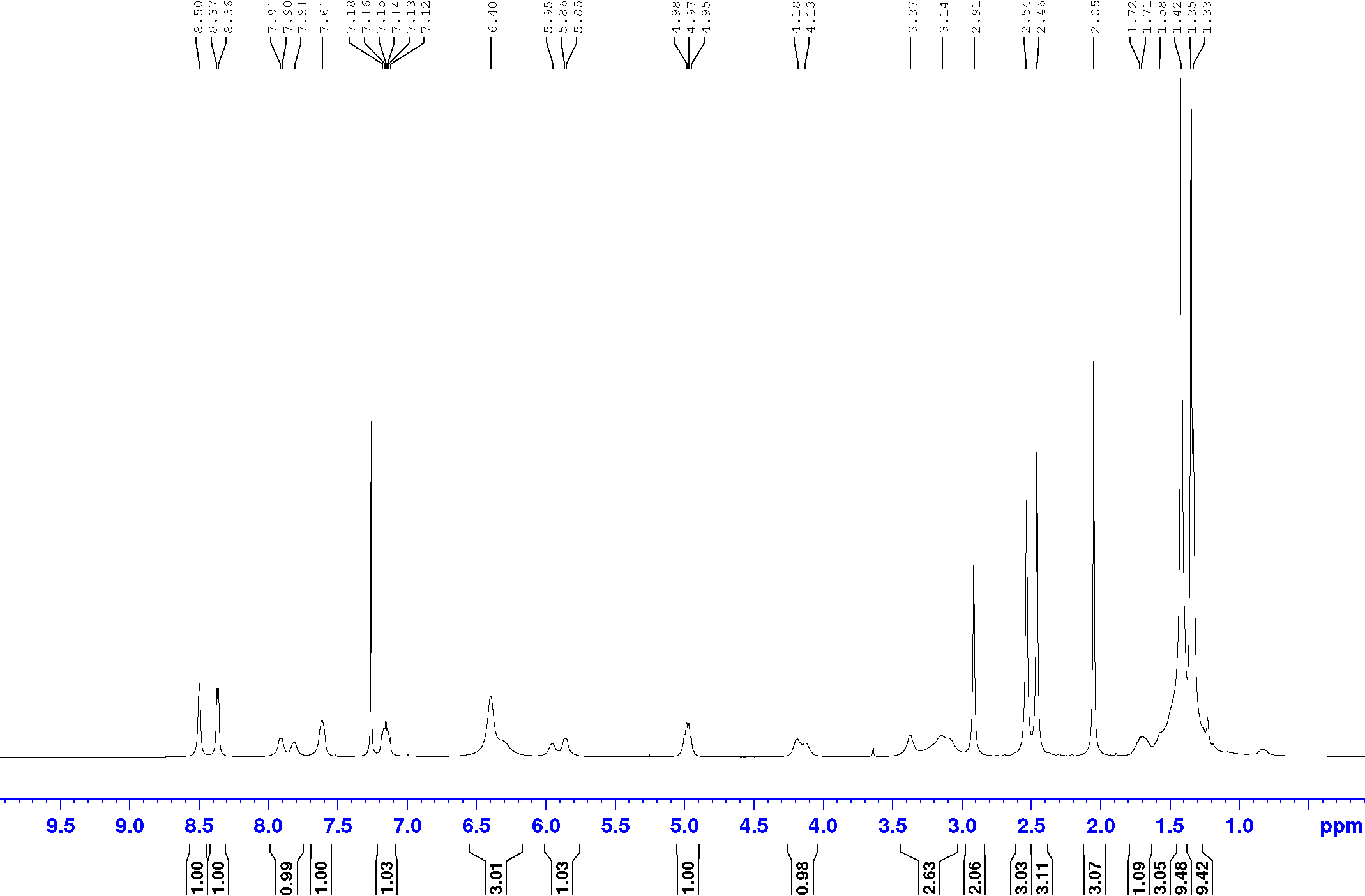
**

**Compound 3g.** ^13^C NMR, CDCl_3_, 100 MHz

**
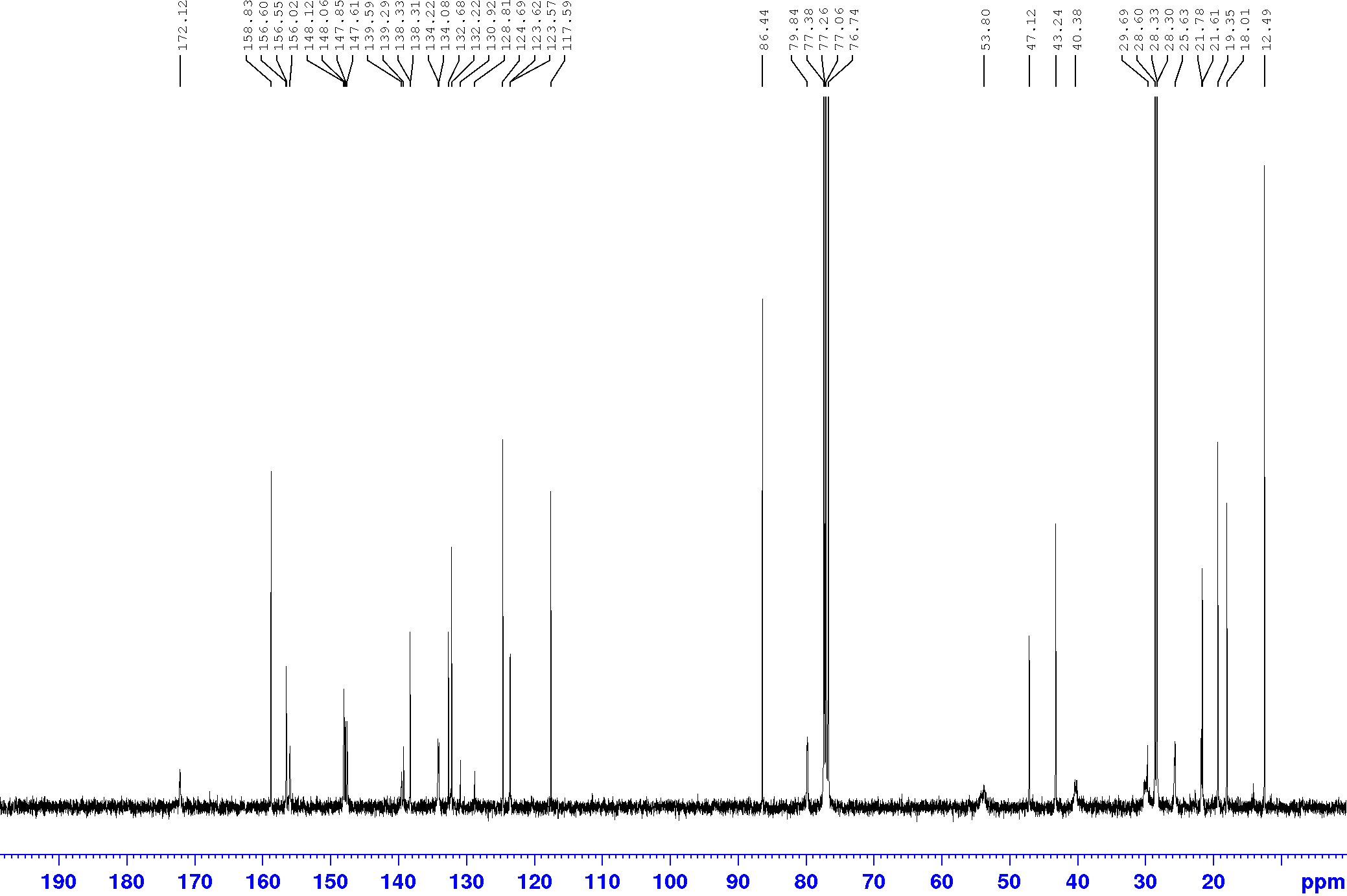
**

**Compound 3h.** ^1^H NMR, CDCl_3_, 400 MHz

**
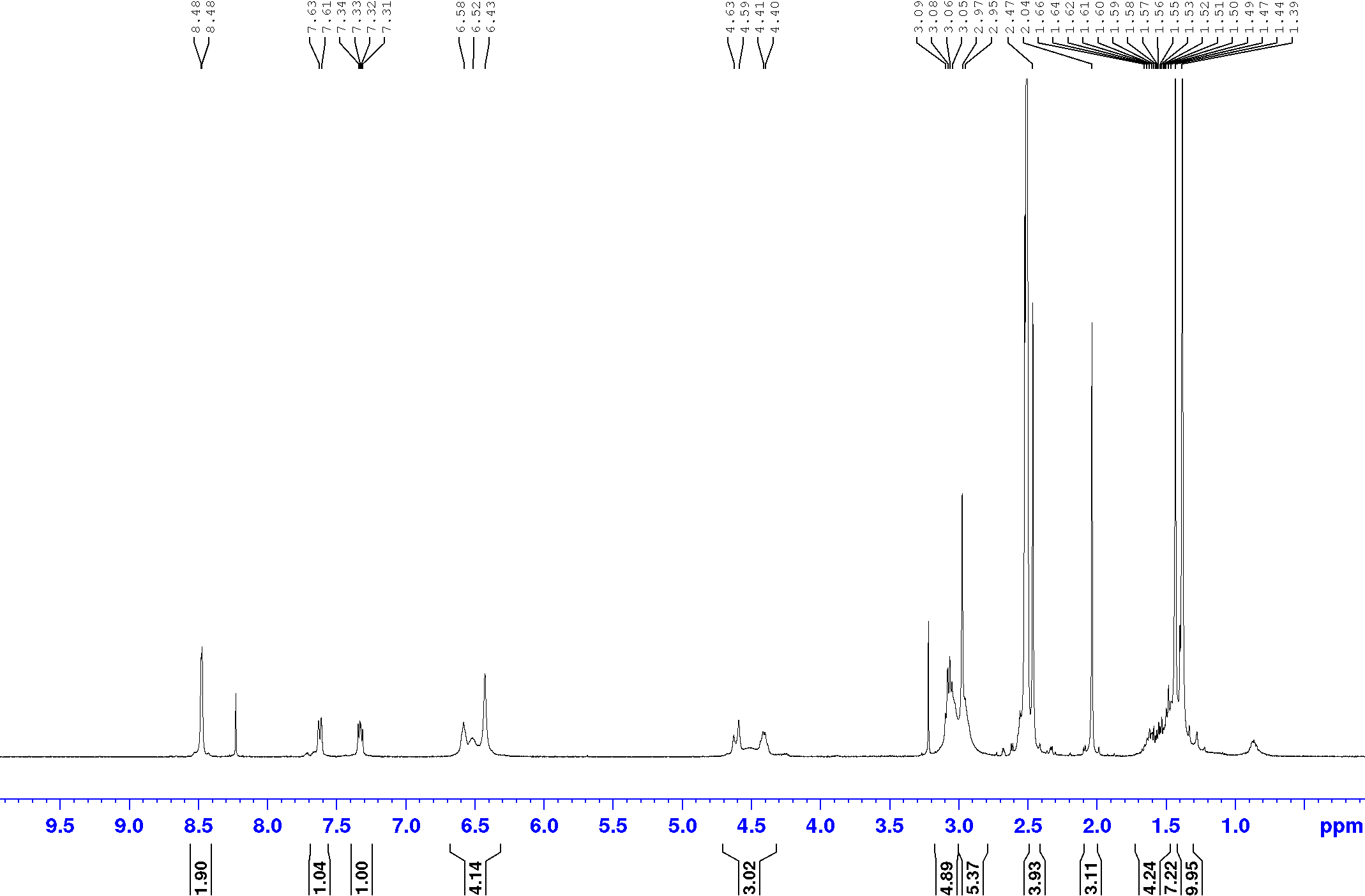
**

**Compound 3h.** ^13^C NMR, CDCl_3_, 100 MHz

**
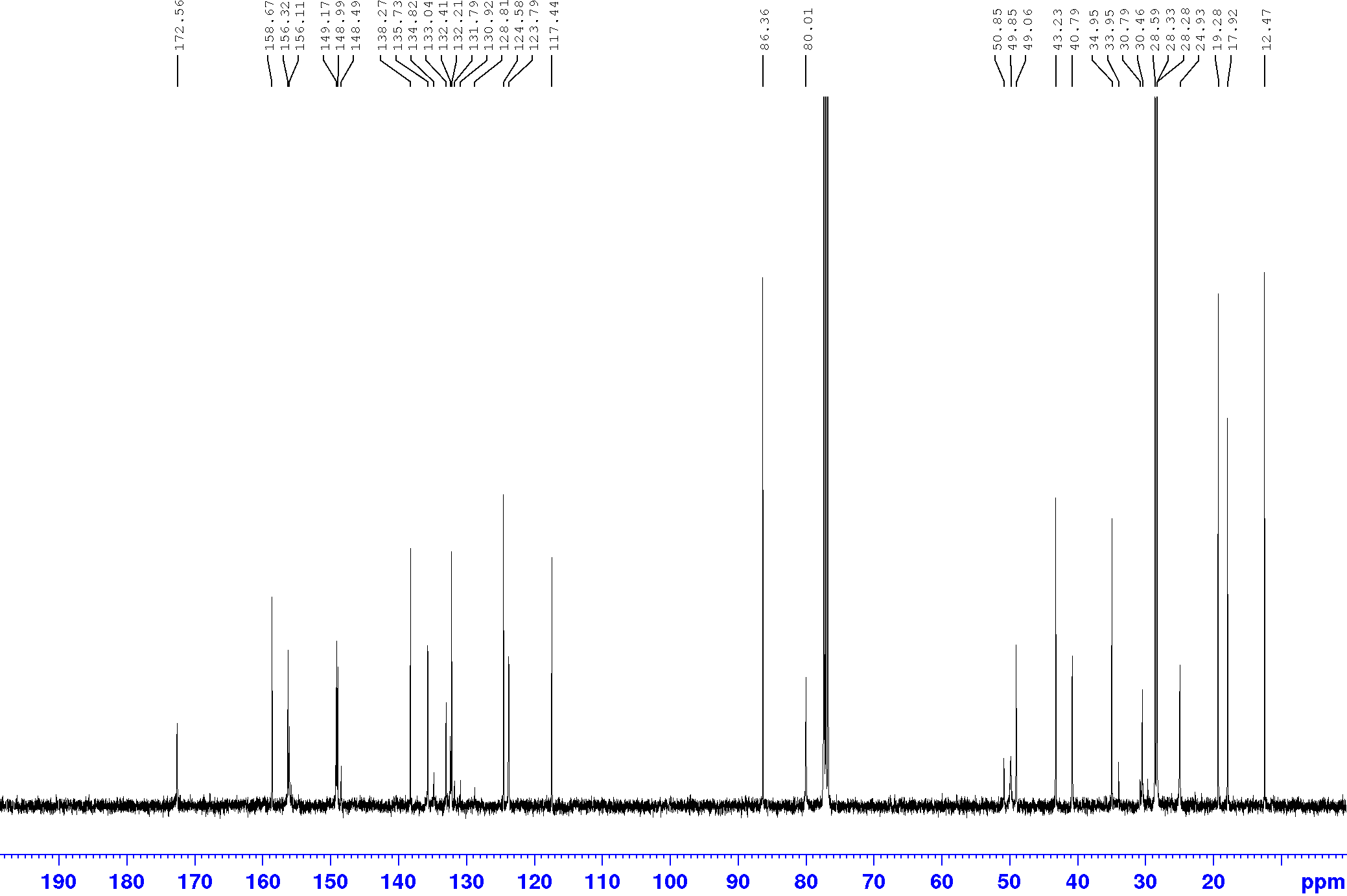
**

**Compound 3i.** ^1^H NMR, CDCl_3_, 400 MHz

**
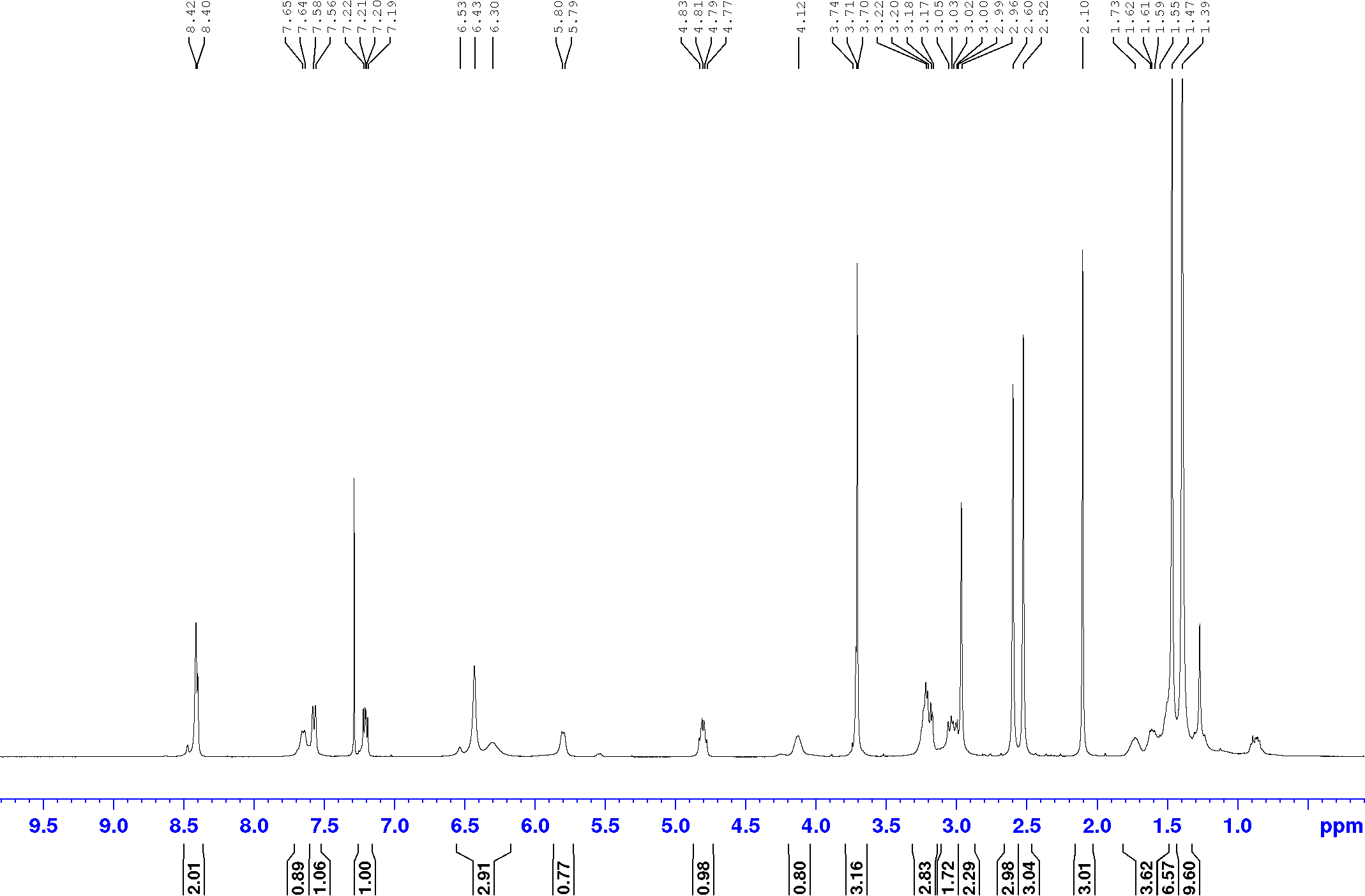
**

**Compound 3i.** ^13^C NMR, CDCl_3_, 100 MHz

**
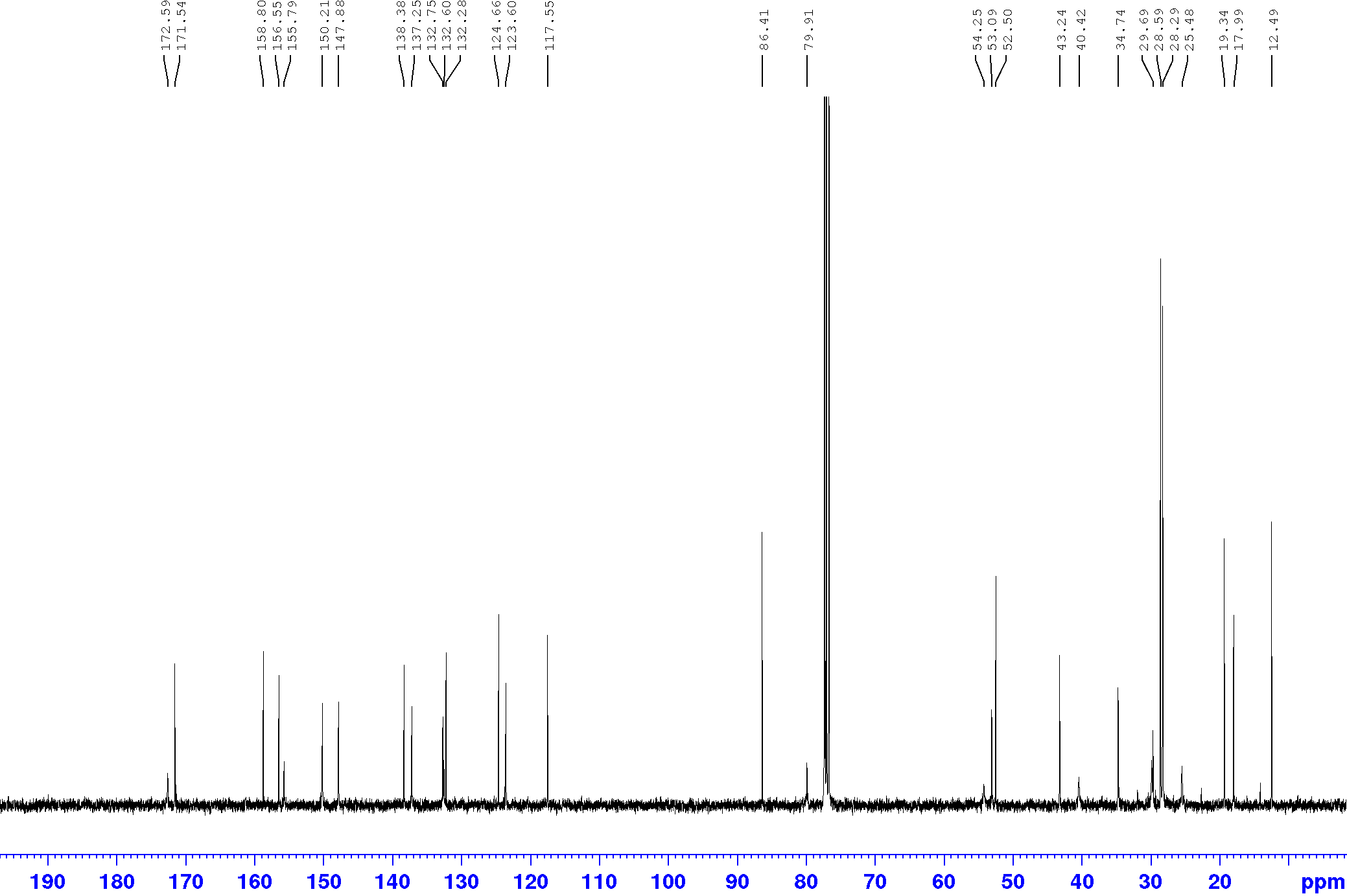
**

**Compound 4a.** ^1^H NMR, D_2_O, 400 MHz

**
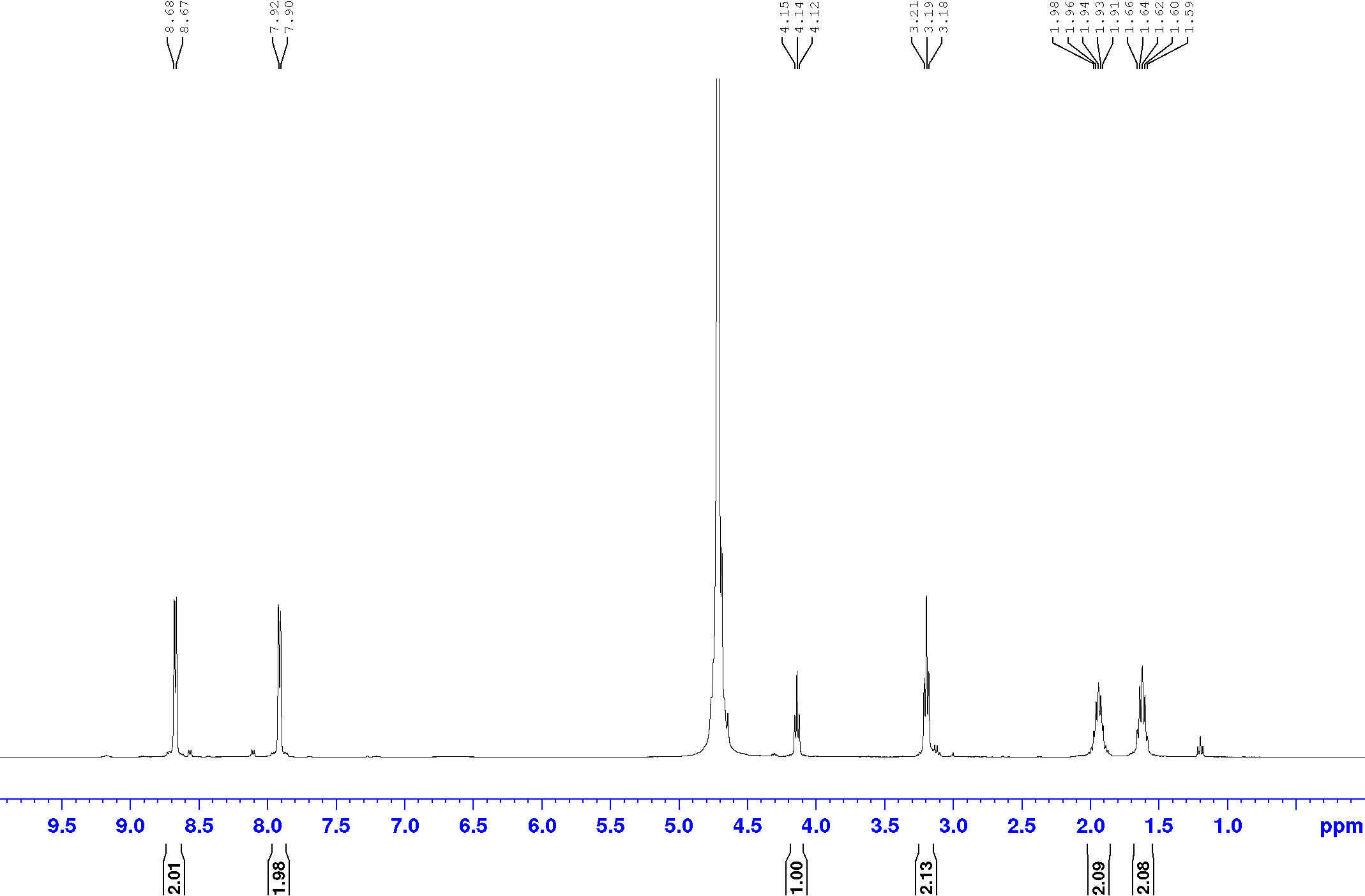
**

**Compound 4a.** ^13^C NMR, D_2_O, 100 MHz

**
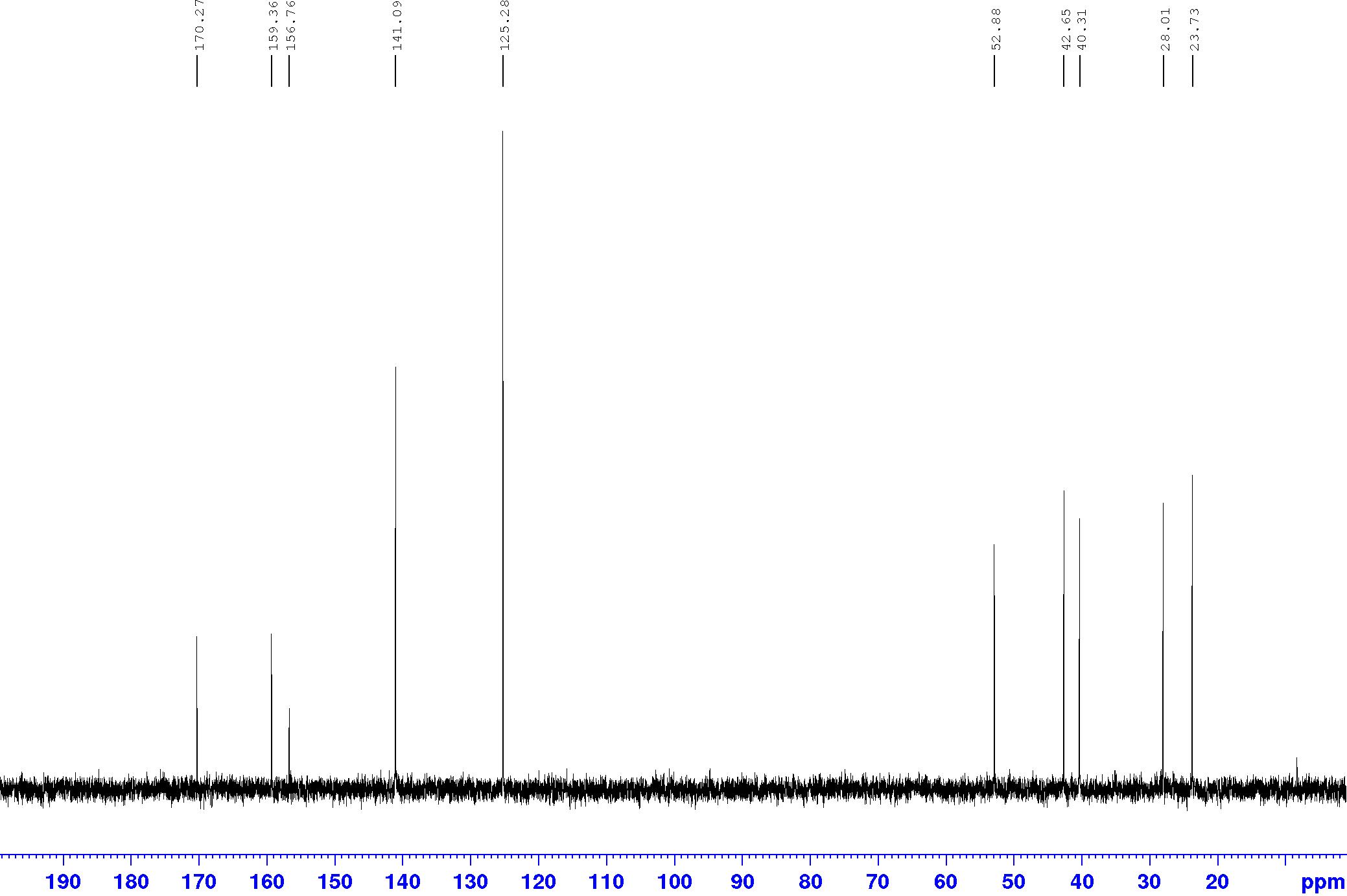
**

**Compound 4b.** ^1^H NMR, D_2_O, 400 MHz

**
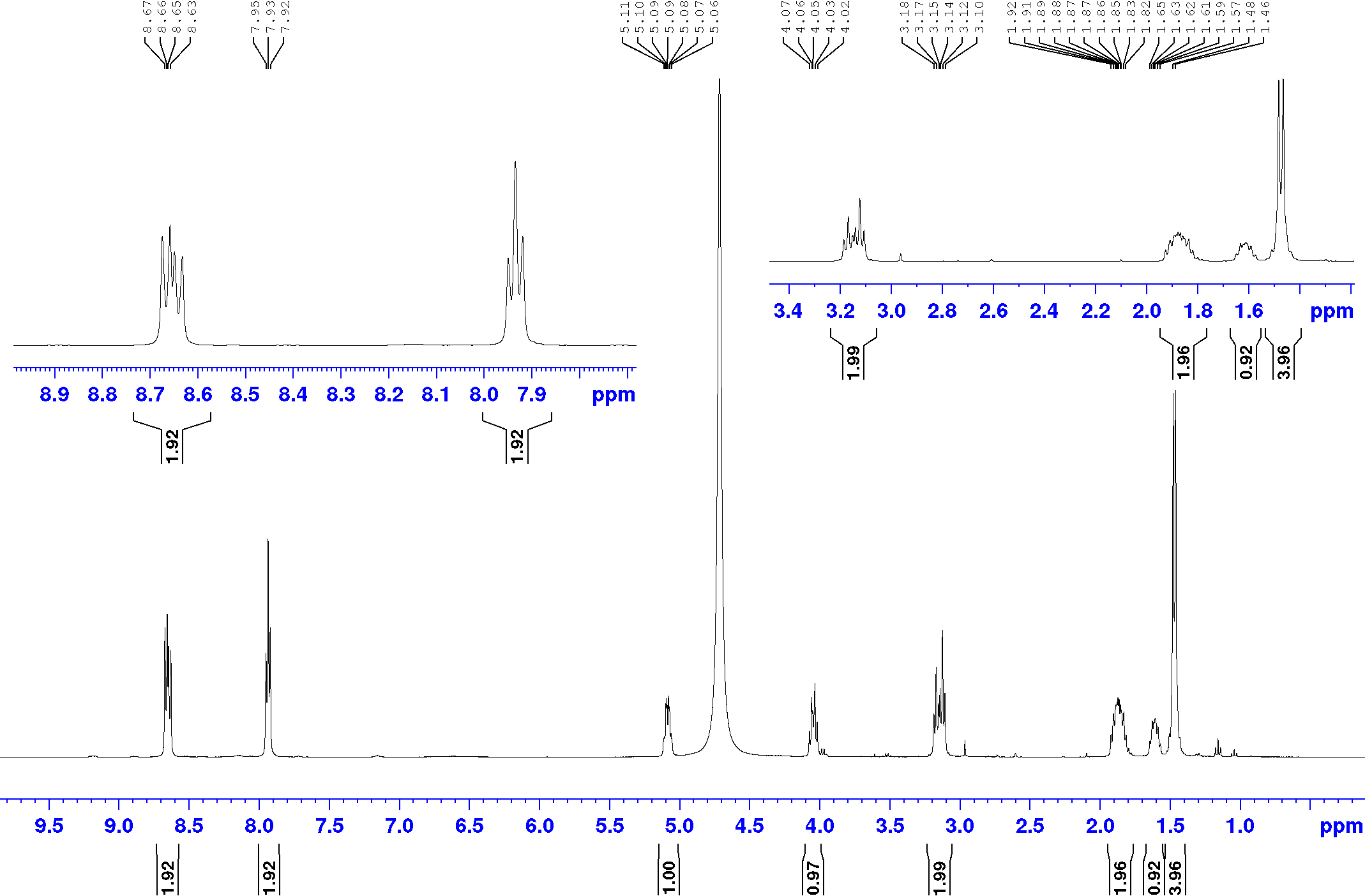
**

*****

*****

*****

**Compound 4b.** ^13^C NMR, D_2_O, 100 MHz

**
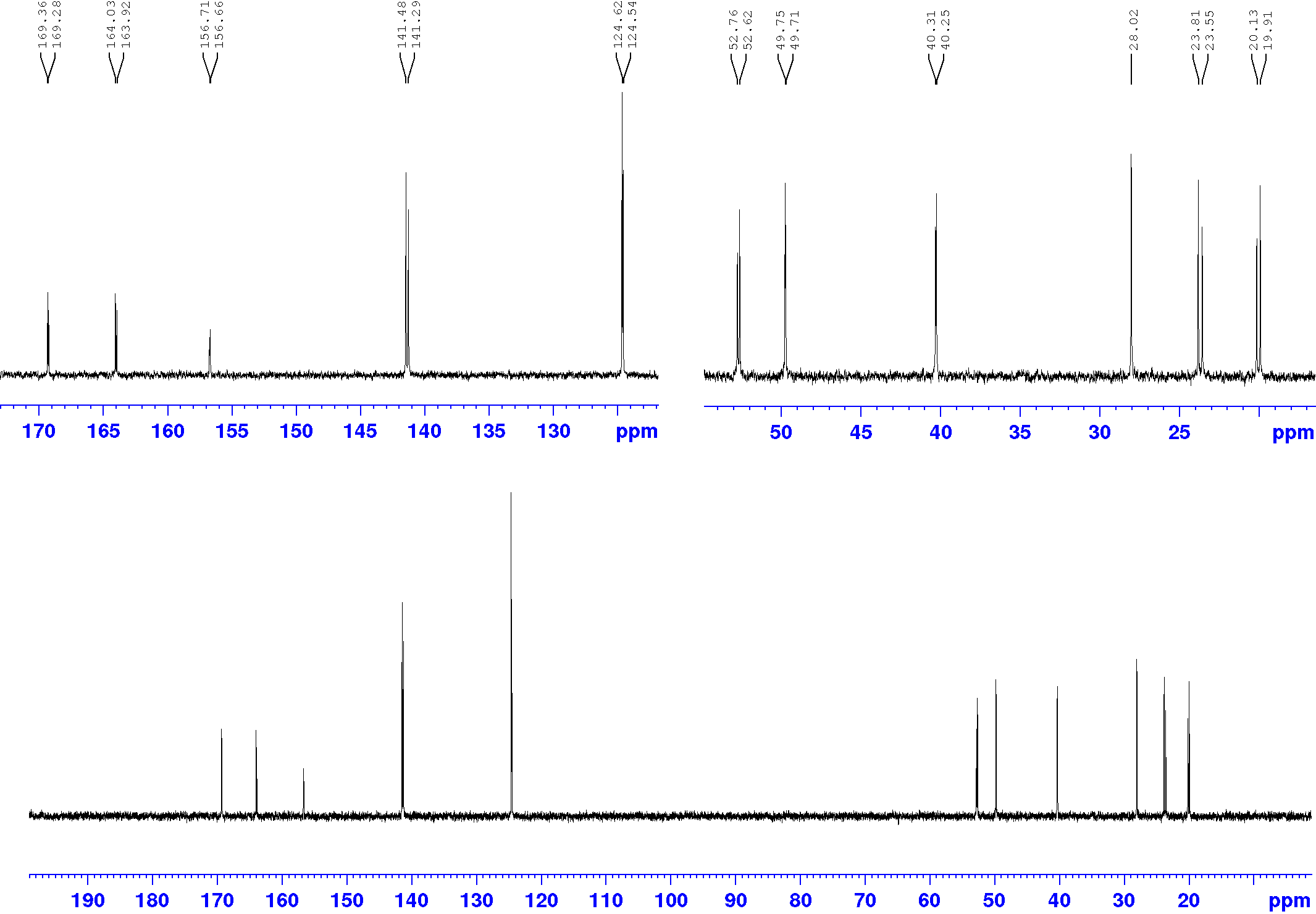
**

*****

*****

*****

*****

*****

*****

*****

*****

*****

*****

**Compound 4c.** ^1^H NMR, D_2_O, 400 MHz

**
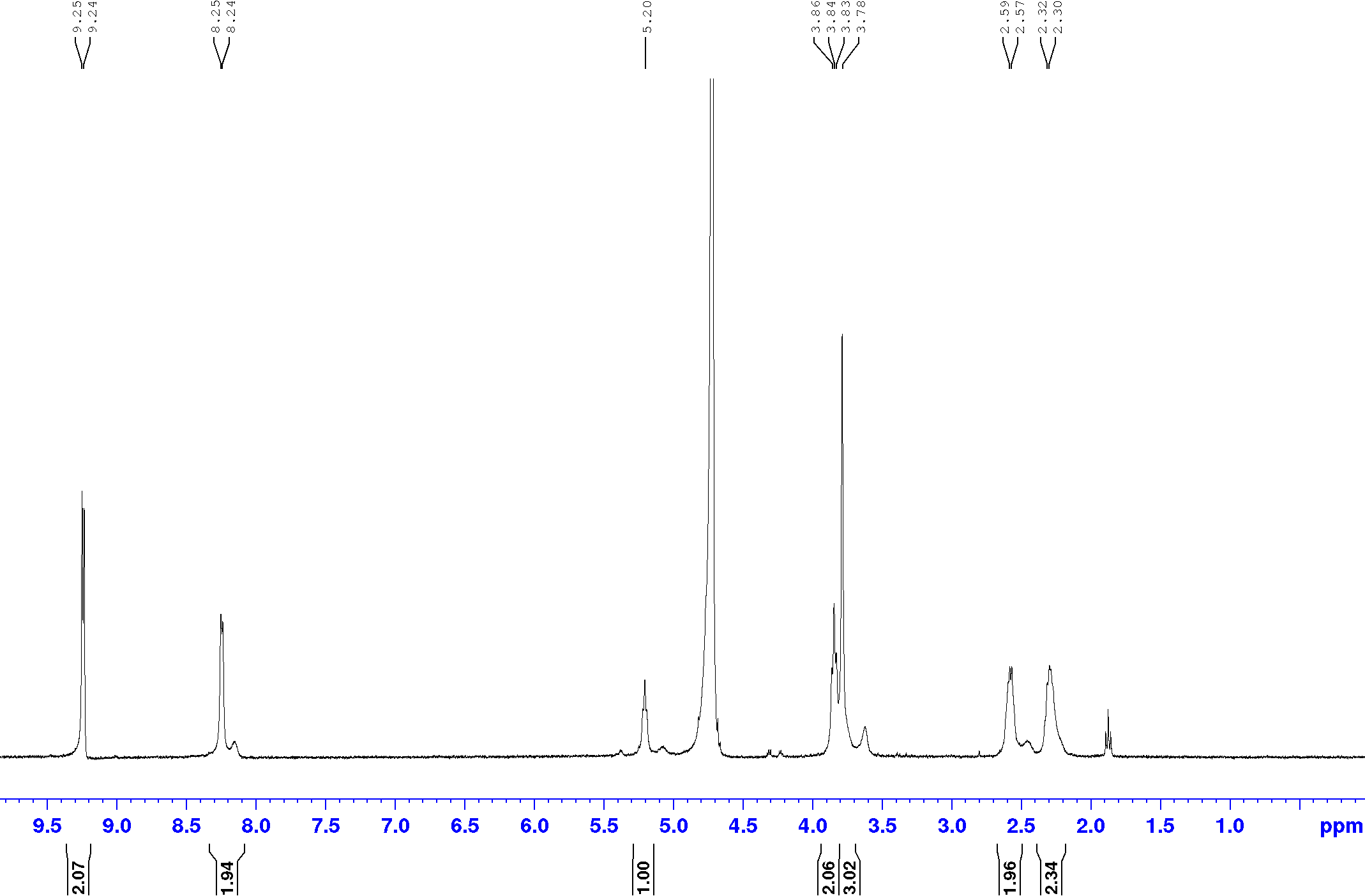
**

**Compound 4c.** ^13^C NMR, D_2_O, 100 MHz

**
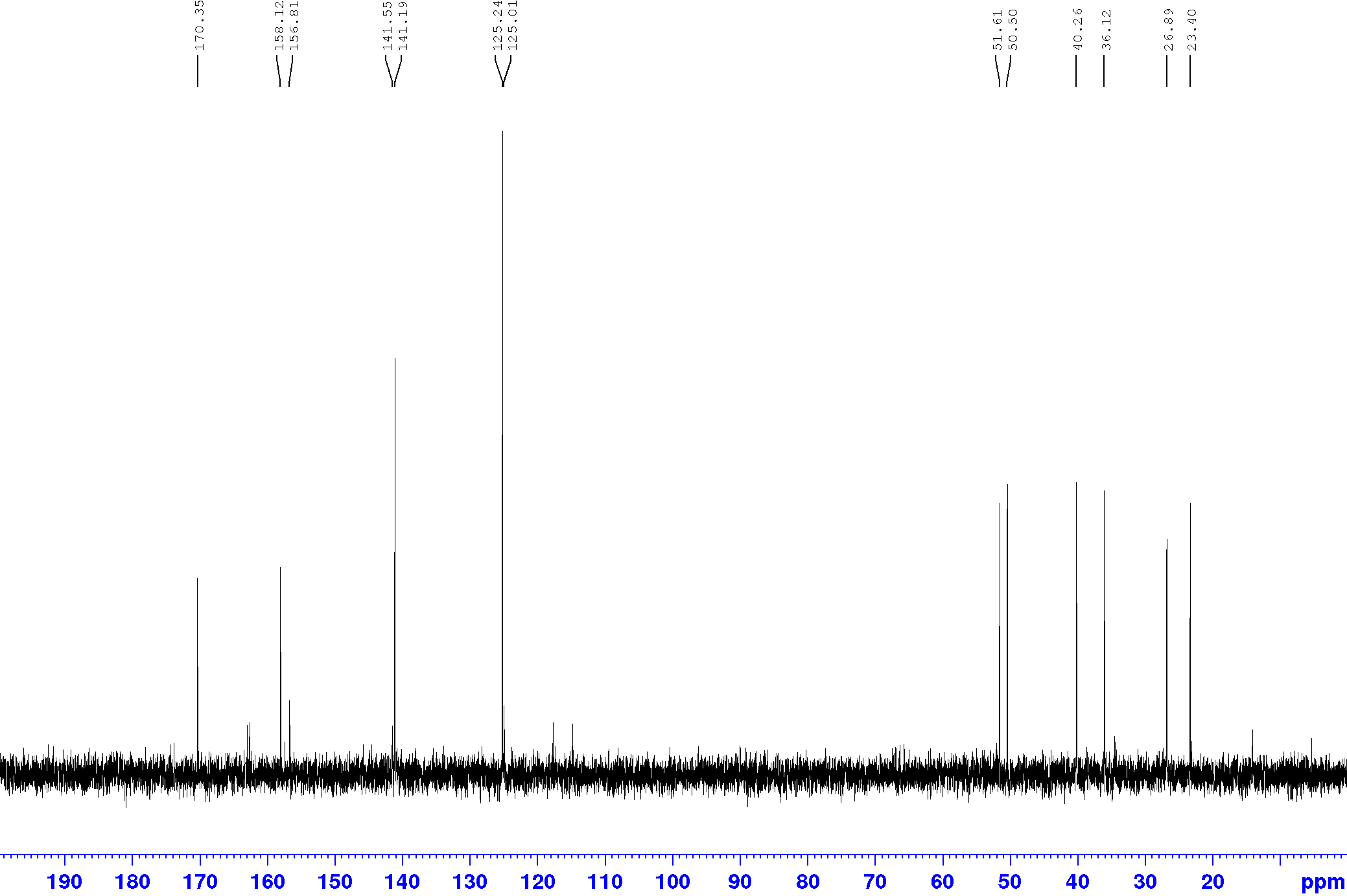
**

**Compound 4d.** ^1^H NMR, D_2_O, 400 MHz

 **
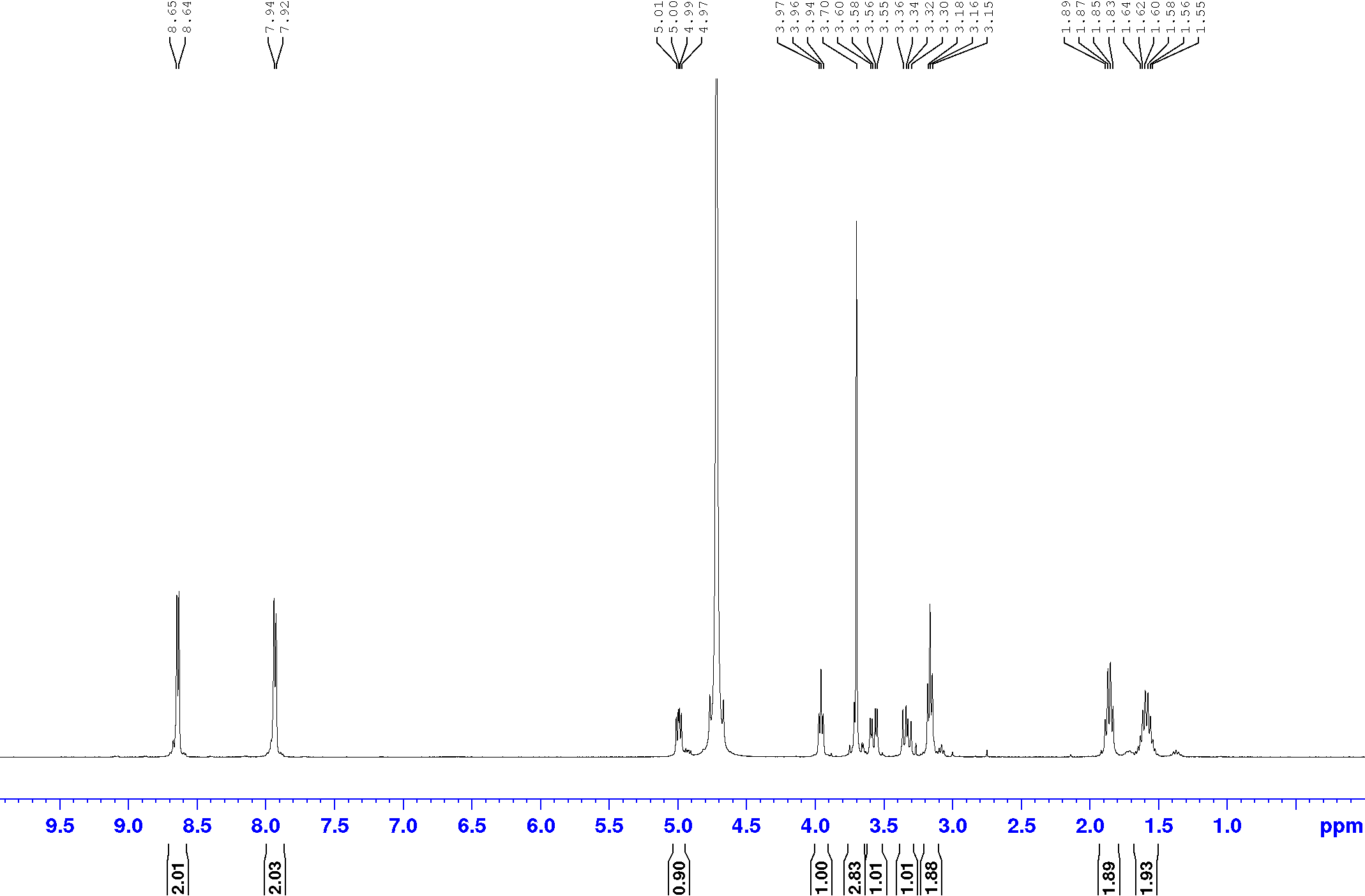
**

**Compound 4d.** ^13^C NMR, D_2_O, 100 MHz

**
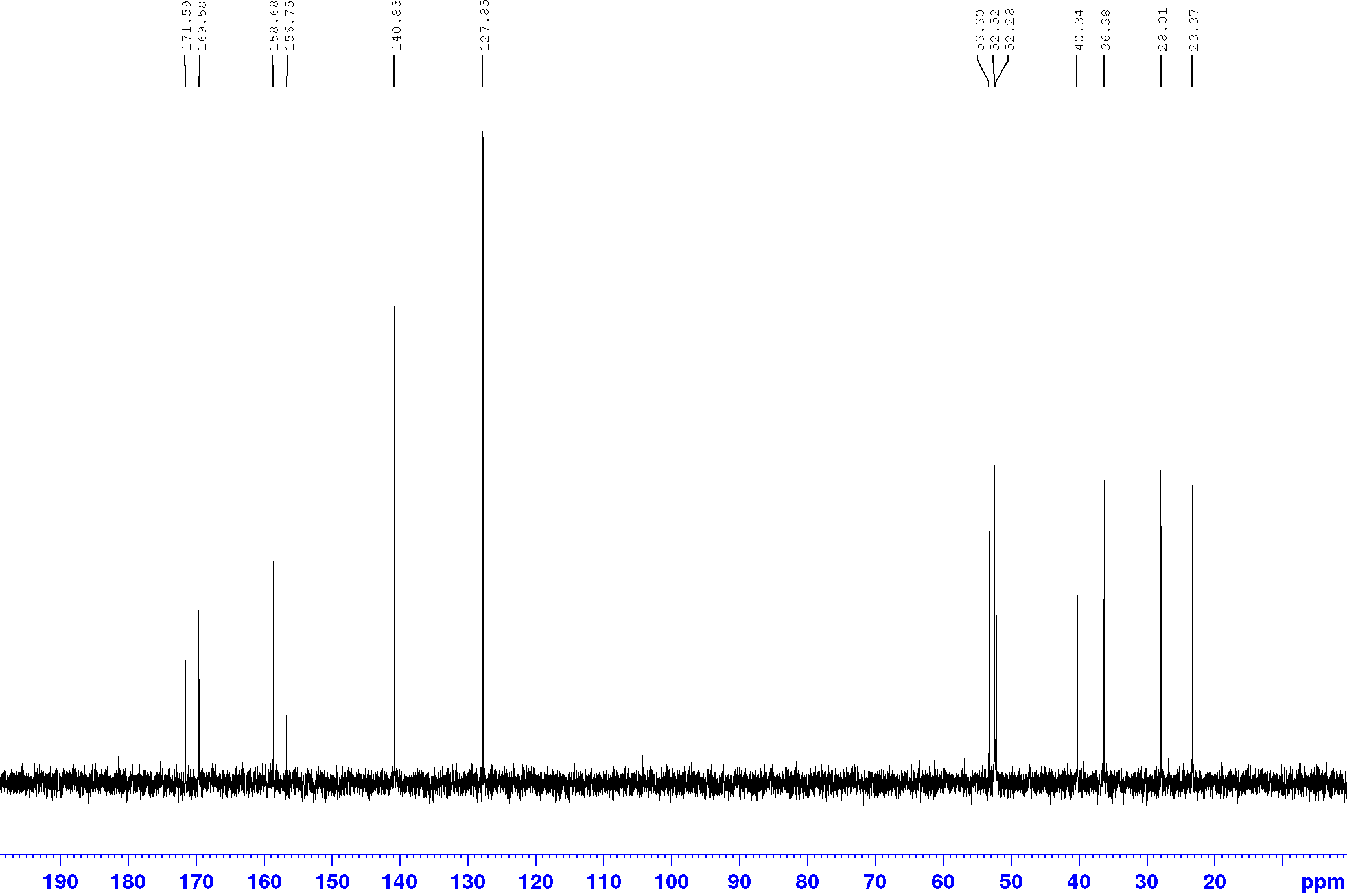
**

**Compound 4e.** ^1^H NMR, D_2_O, 400 MHz

**
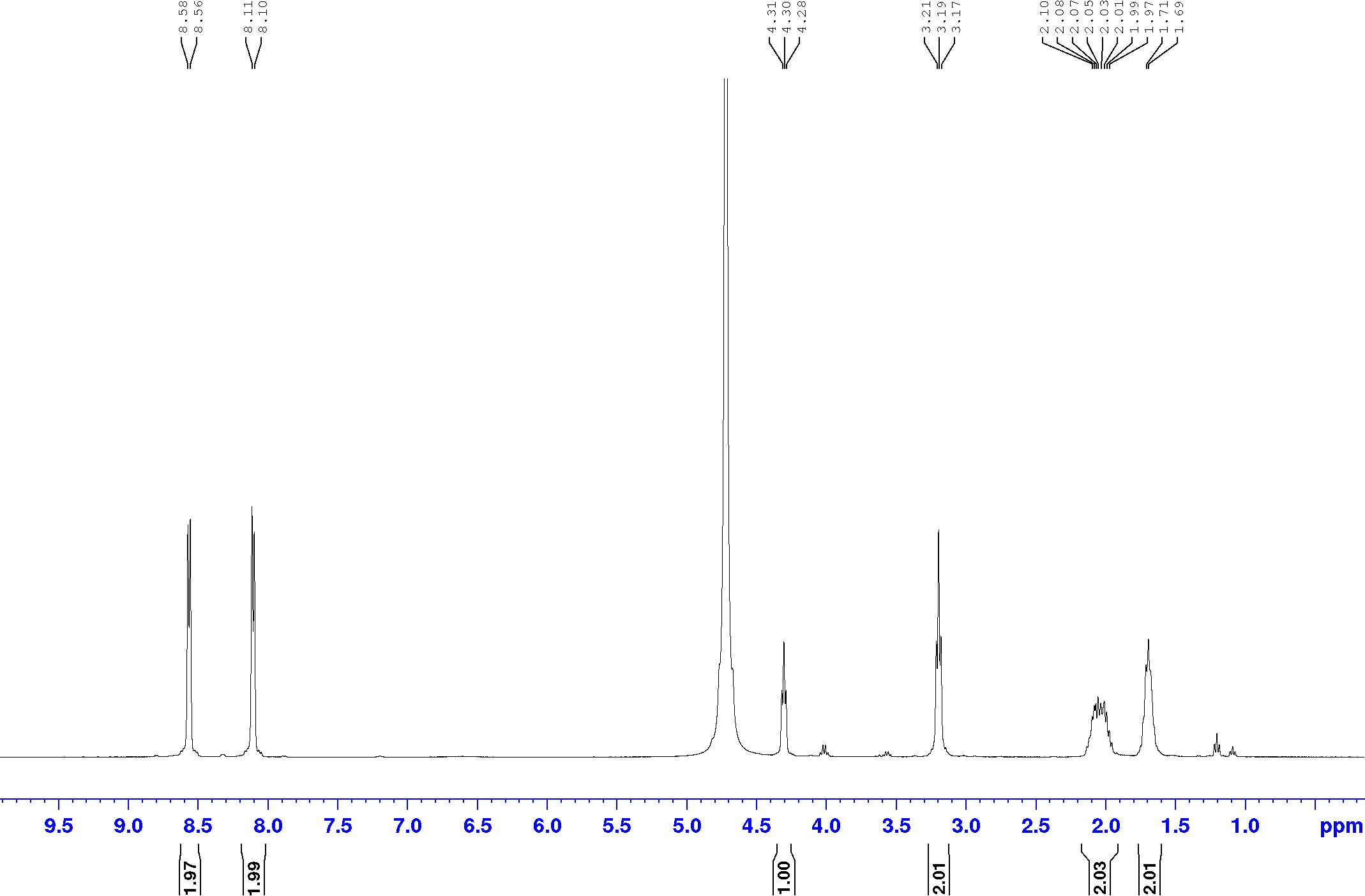
**

**Compound 4e.** ^13^C NMR, D_2_O, 100 MHz

**
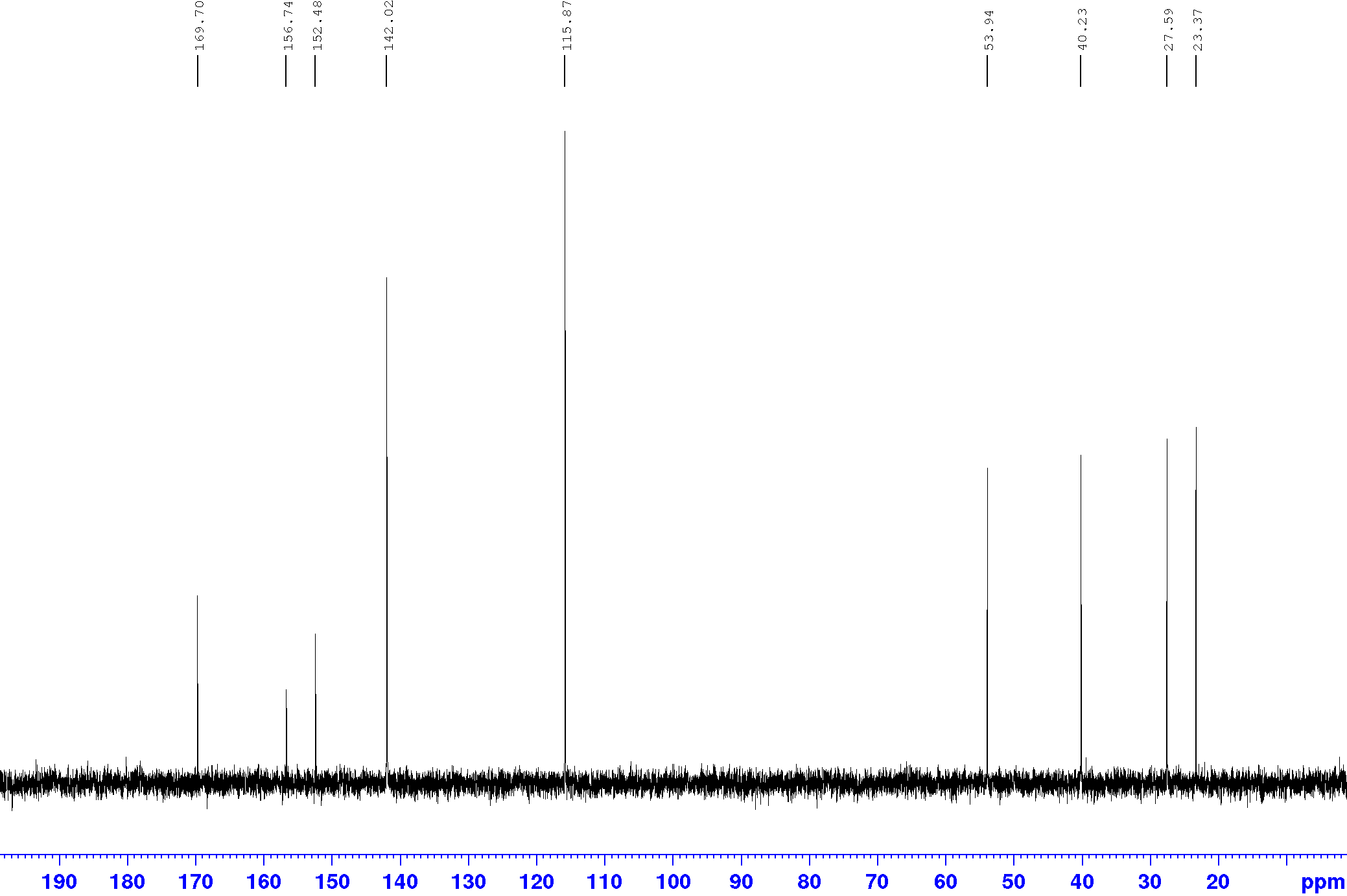
**

**Compound 4f.** ^1^H NMR, D_2_O, 400 MHz

**
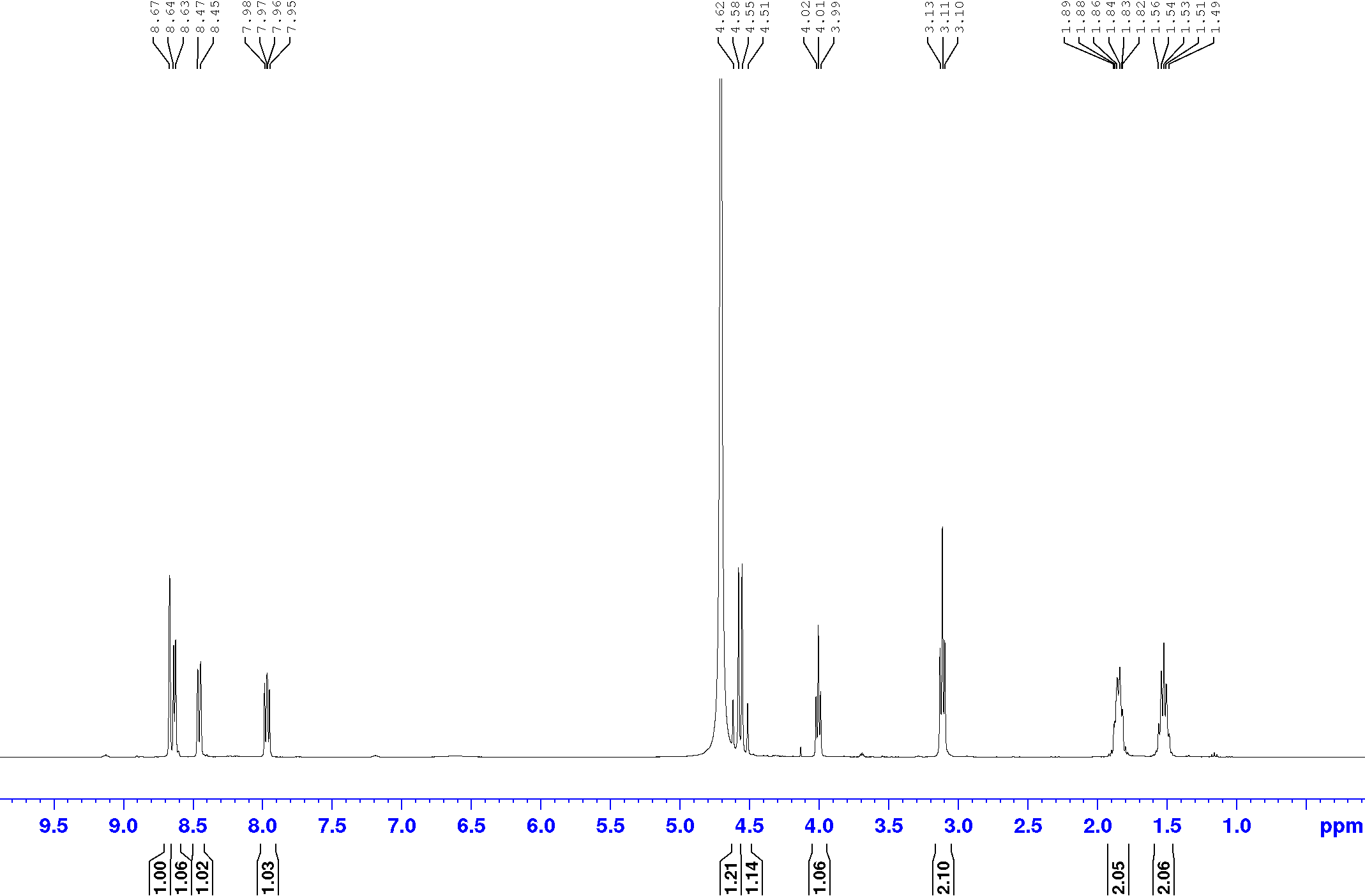
**

**Compound 4f.** ^13^C NMR, D_2_O, 100 MHz

**
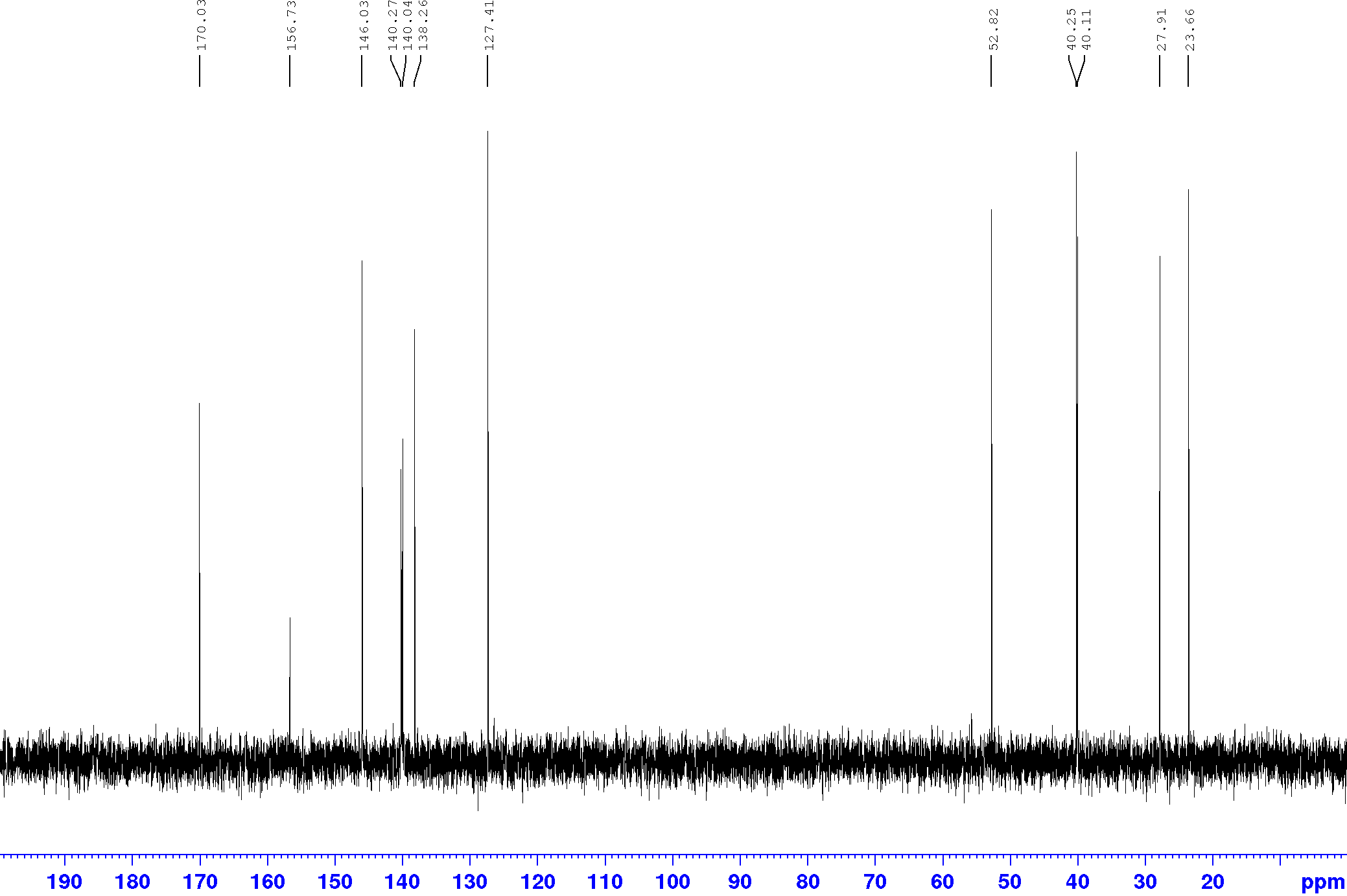
**

**Compound 4g.** ^1^H NMR, D_2_O, 400 MHz

**
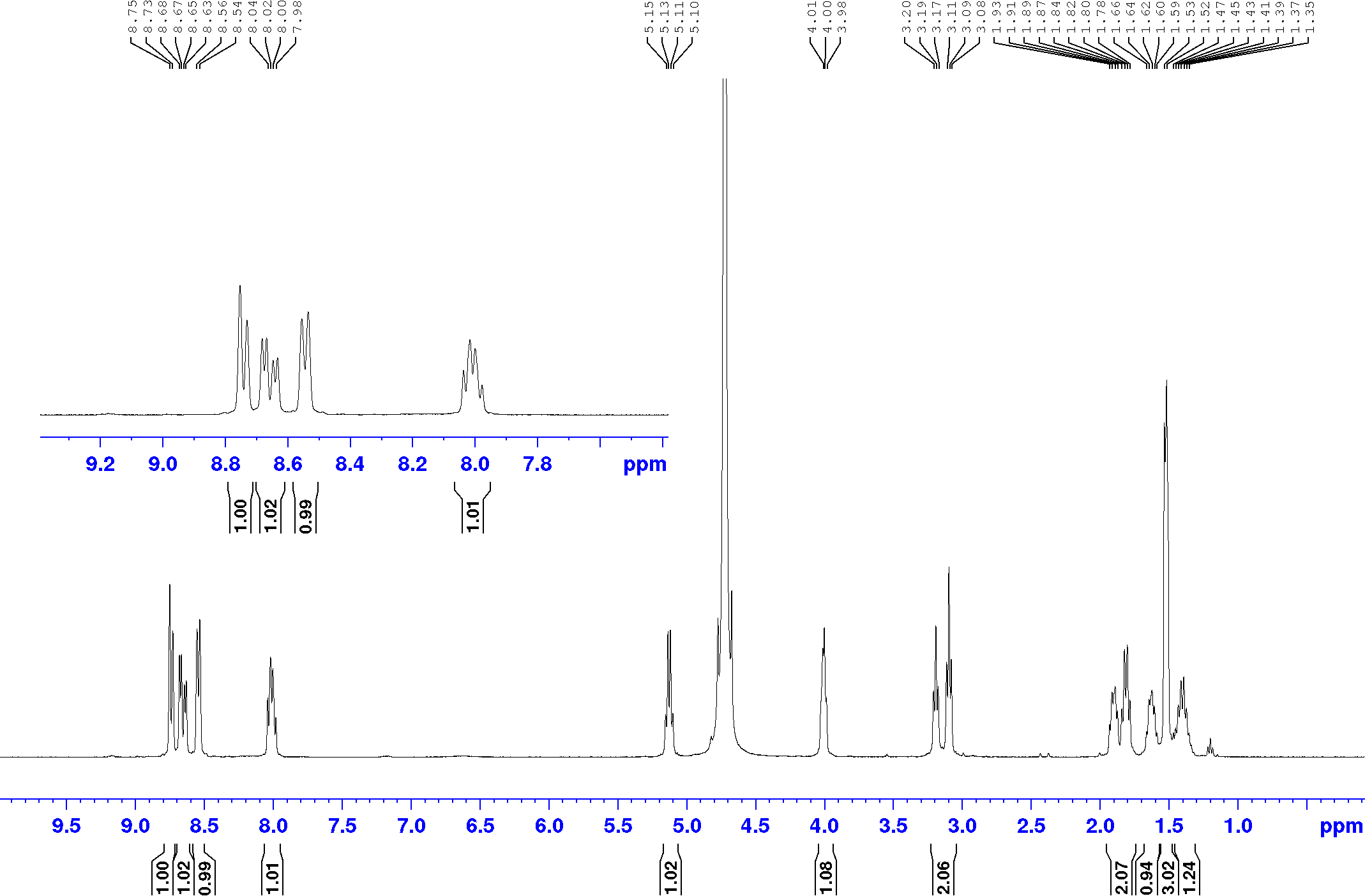
**

*****

*****

*****

*****

*****

*****

**Compound 4g.** ^13^C NMR, D_2_O, 100 MHz

**
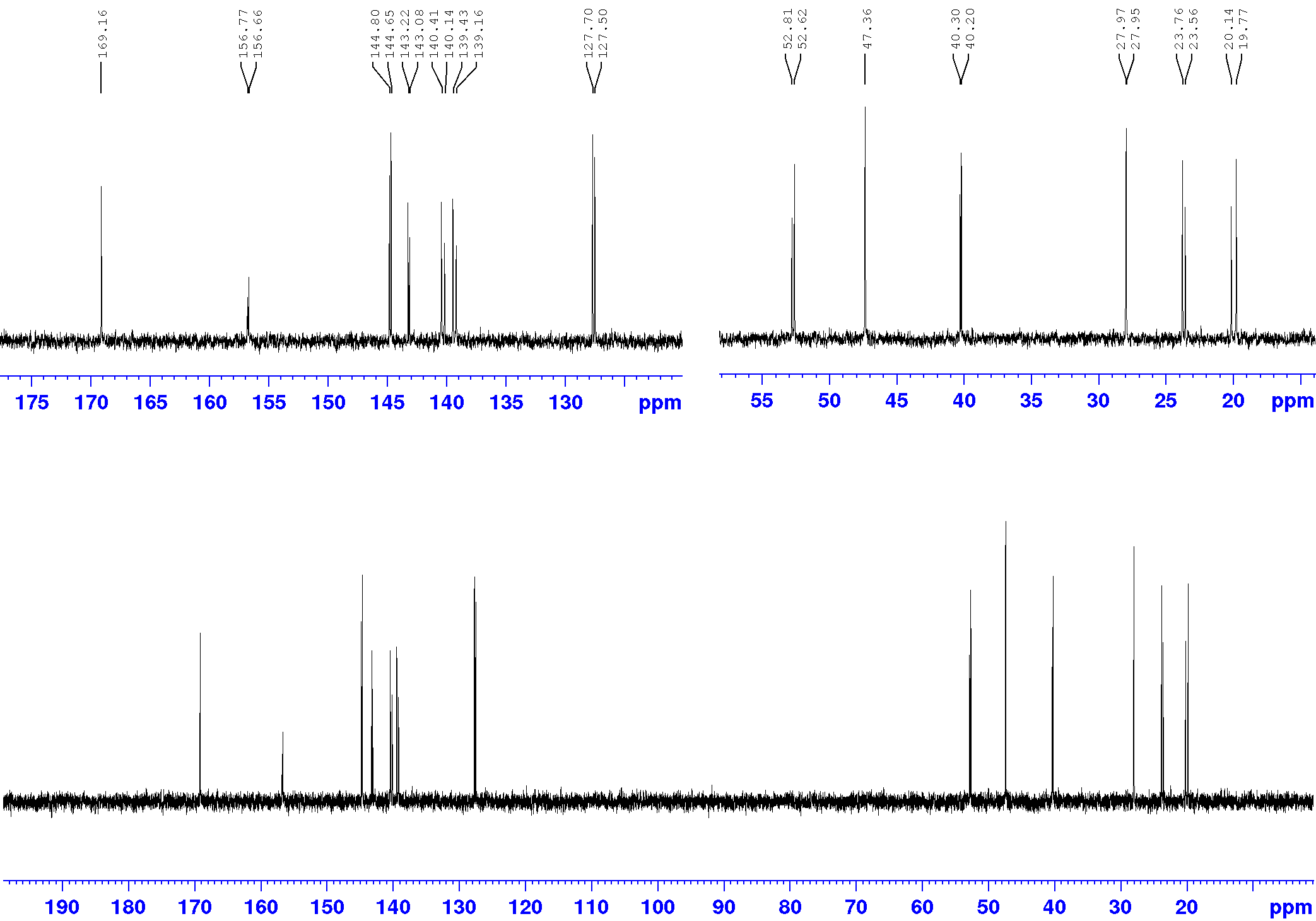
**

*****

*****

*****

*****

*****

*****

*****

*****

*****

*****

*****

**Compound 4h.** ^1^H NMR, D_2_O, 400 MHz

**
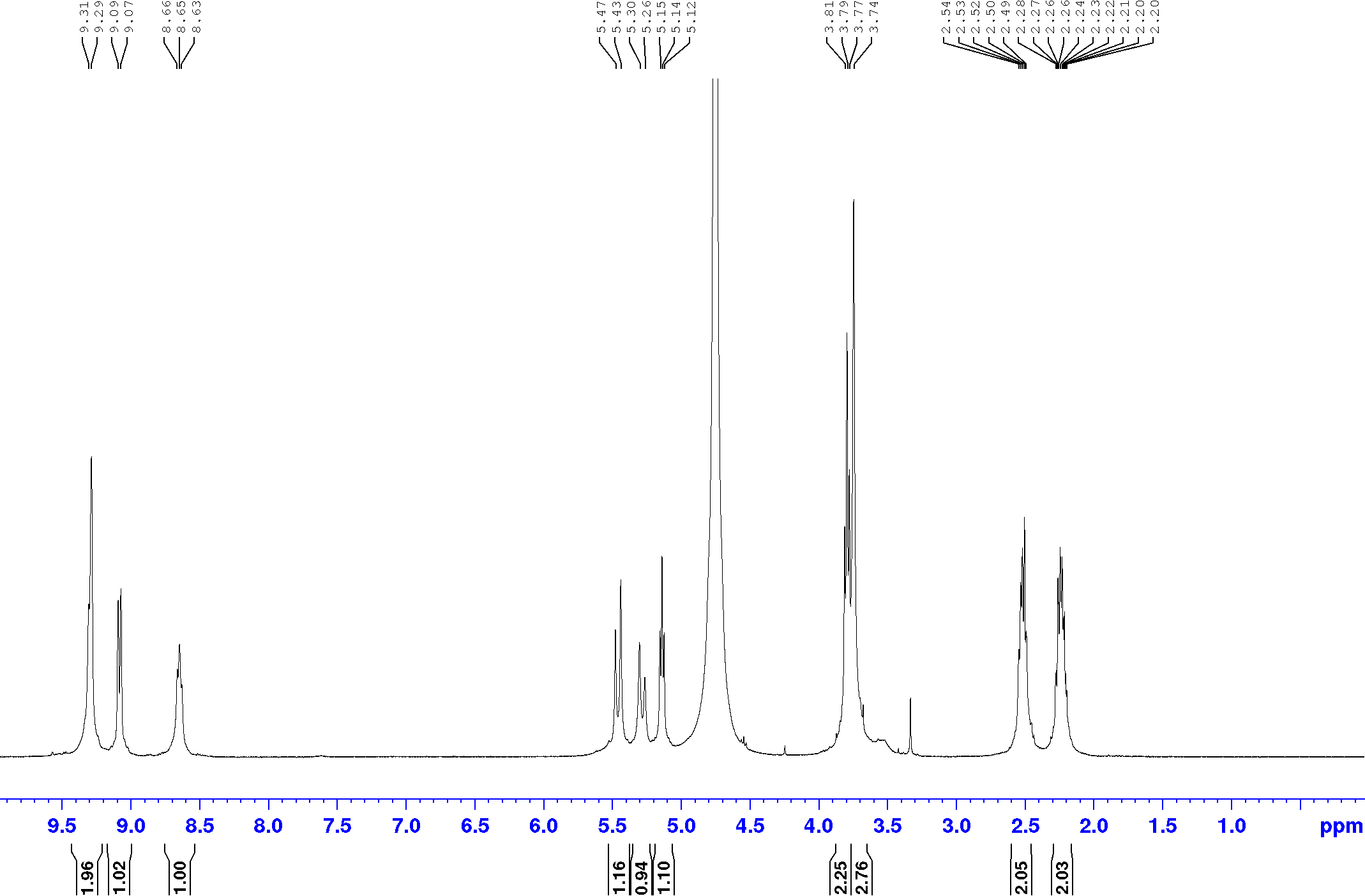
**

**Compound 4h.** ^13^C NMR, D_2_O, 100 MHz

**
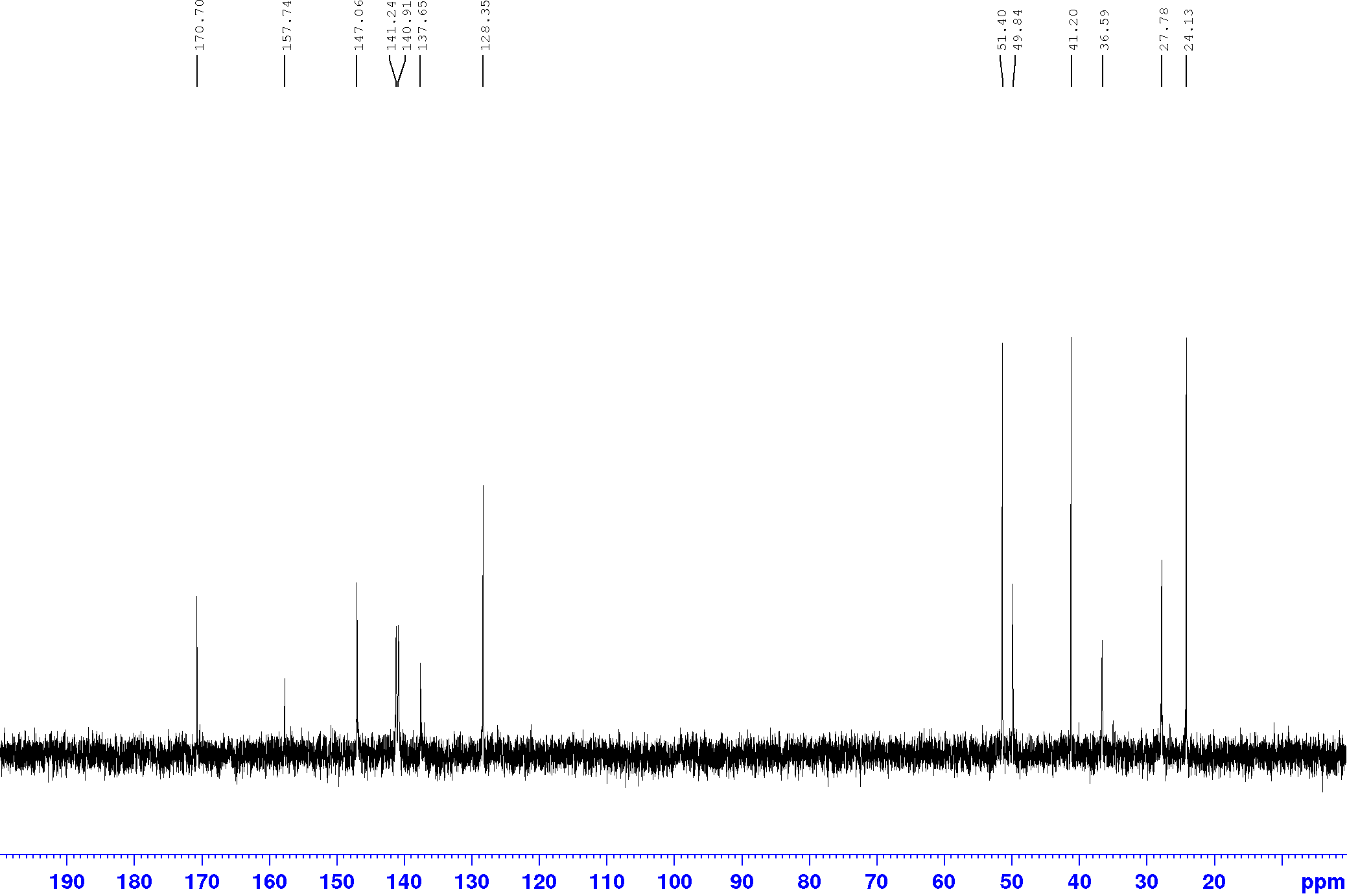
**

**Compound 4i.** ^1^H NMR, D_2_O, 400 MHz

**
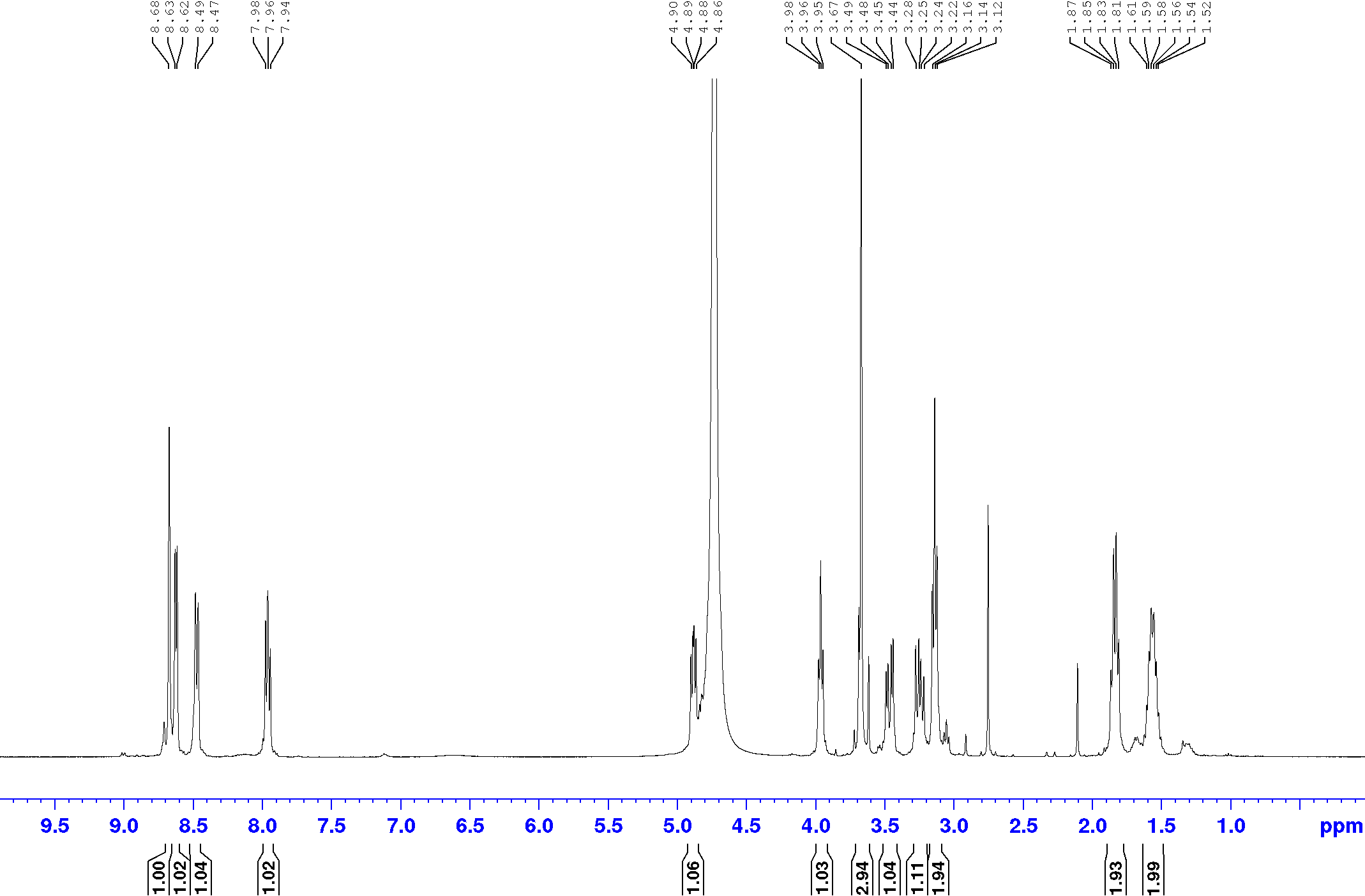
**

**Compound 4i.** ^13^C NMR, D_2_O, 100 MHz

**
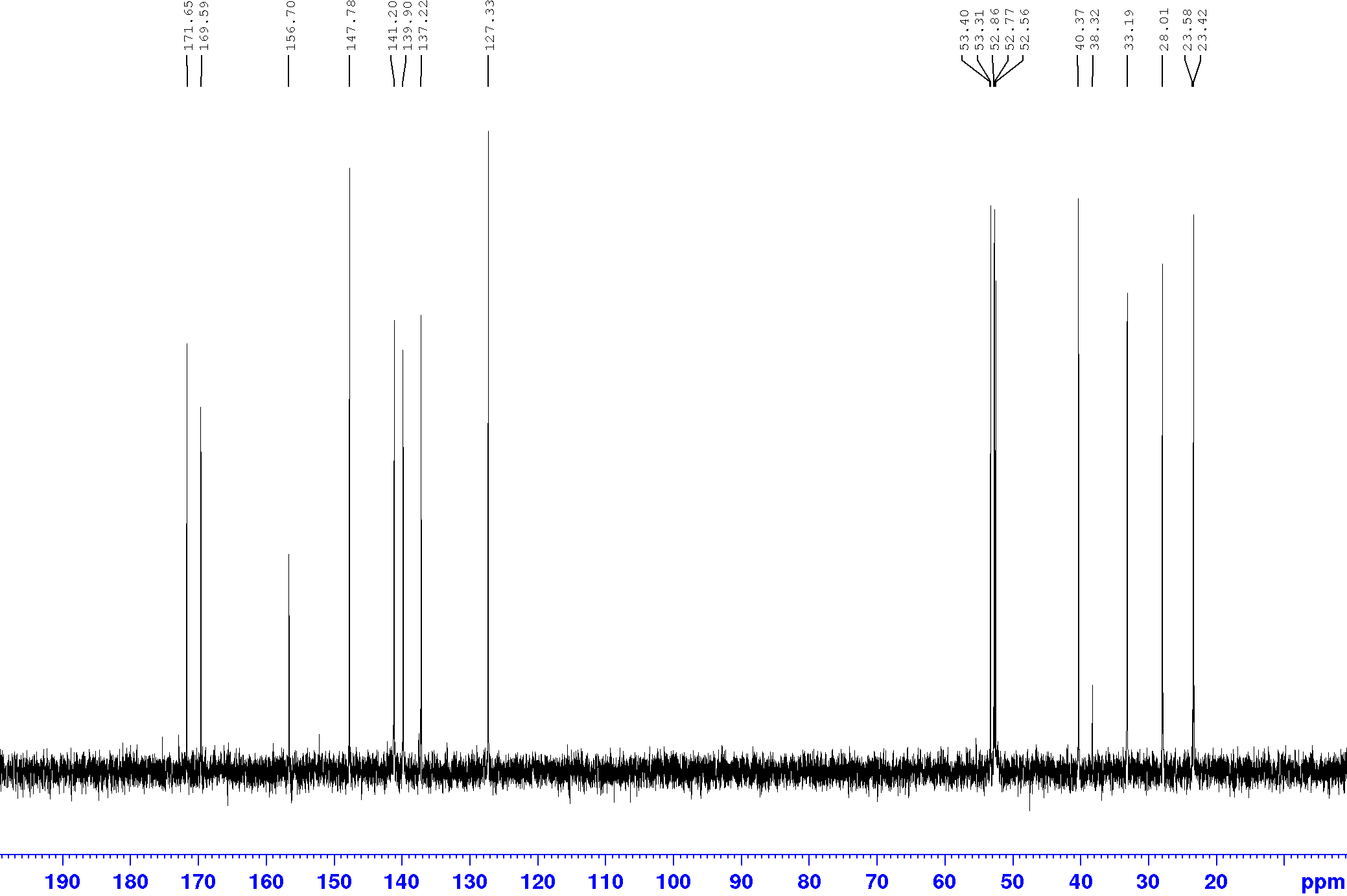
**

**Compound 4j.** ^1^H NMR, D_2_O, 400 MHz

**
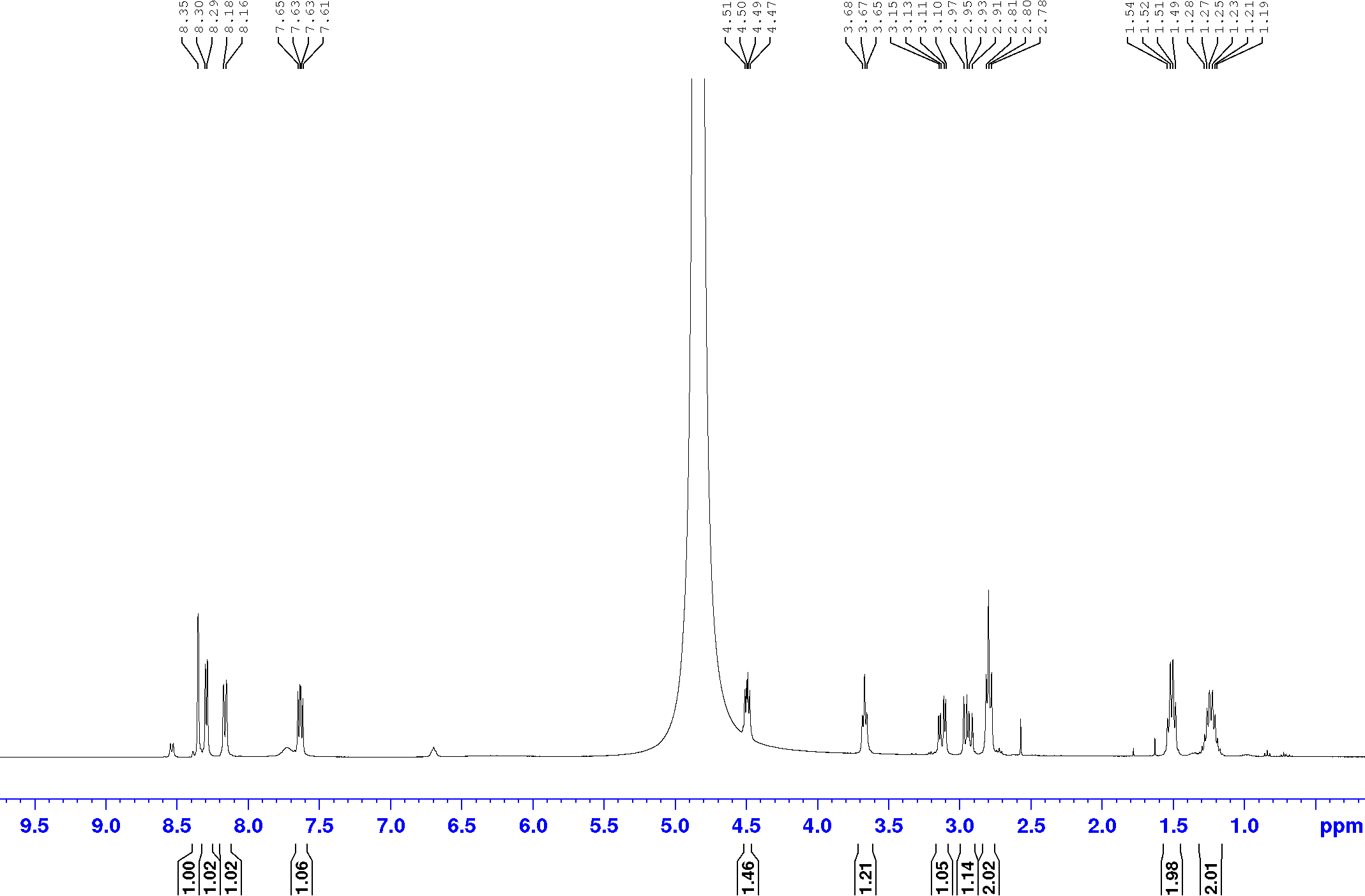
**

**Compound 4j.** ^13^C NMR, D_2_O, 100 MHz

**
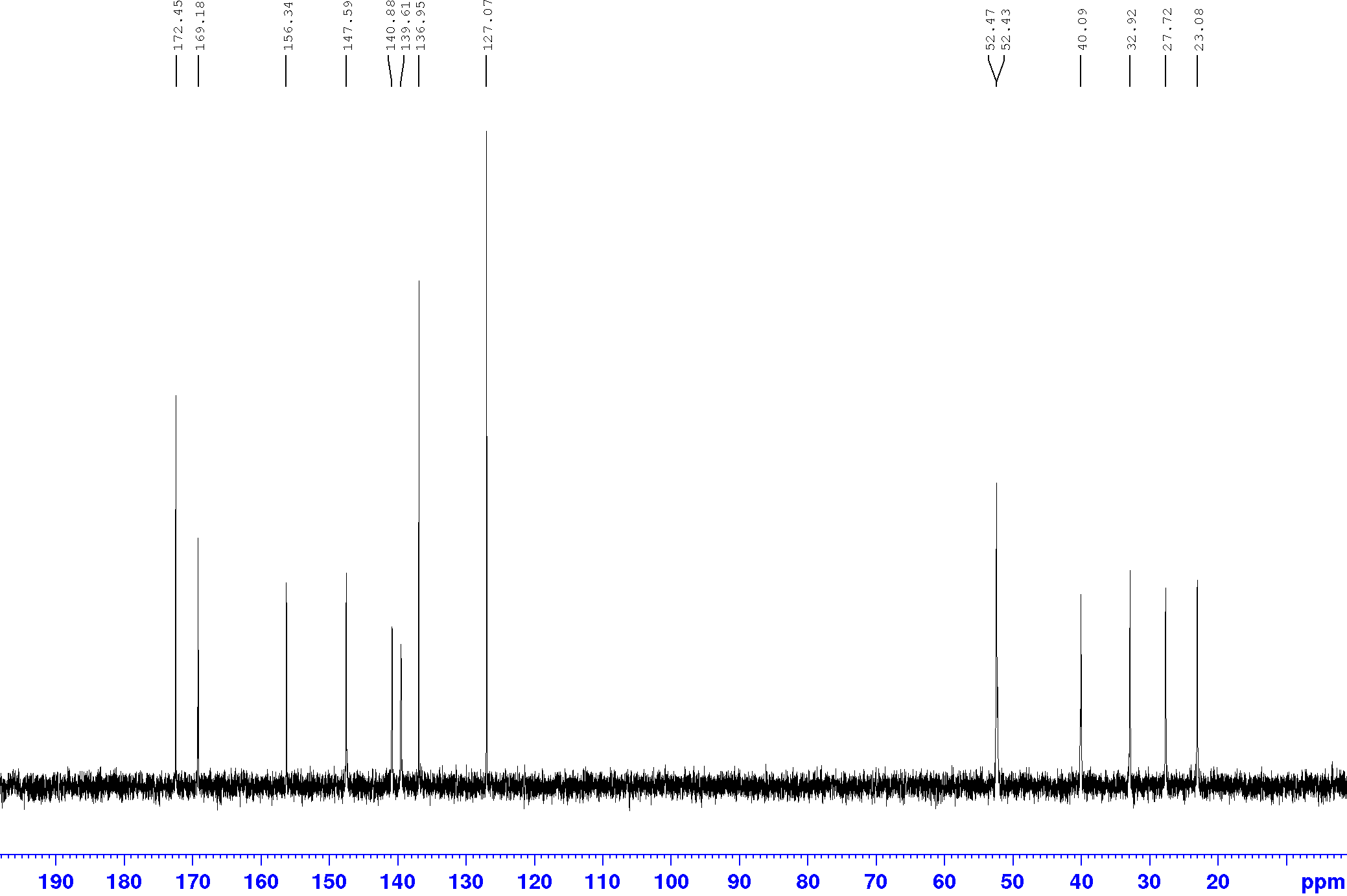
**

**Compound 8.** ^1^H NMR, CD_3_OD, 400 MHz


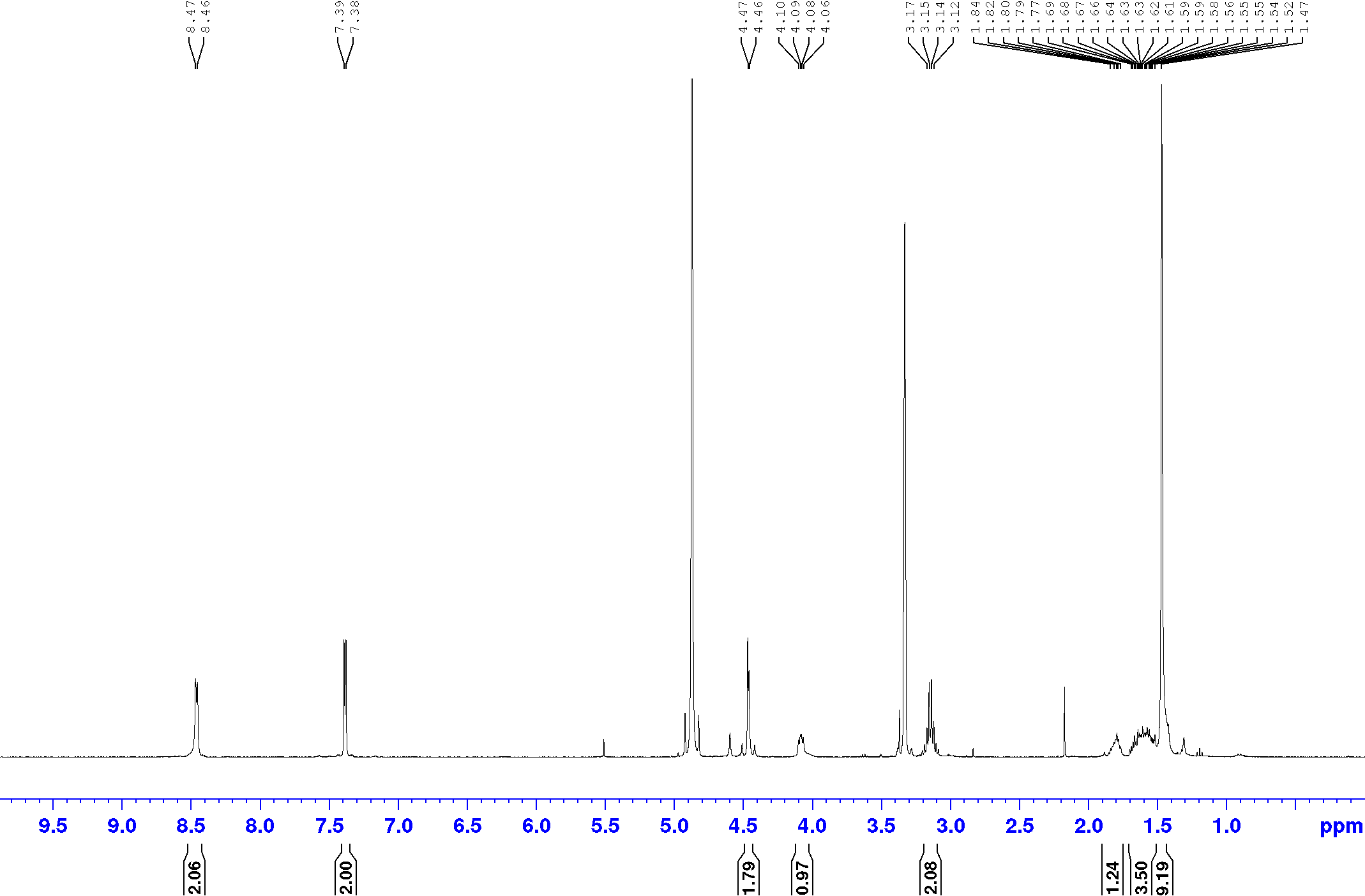


**Compound 8.** ^13^C NMR, CD_3_OD, 100 MHz


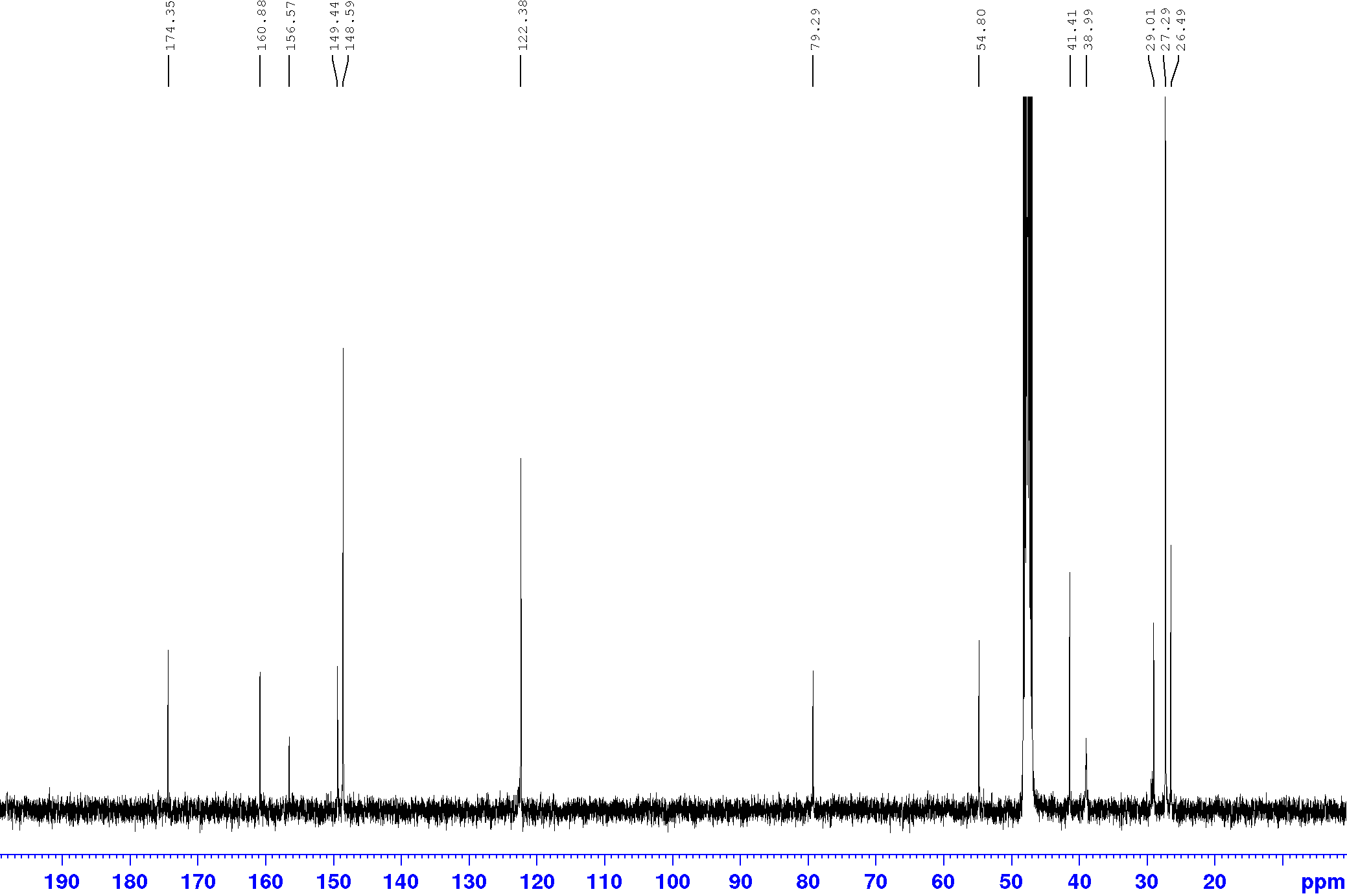


**Compound 9.** ^1^H NMR, CD_3_OD, 400 MHz


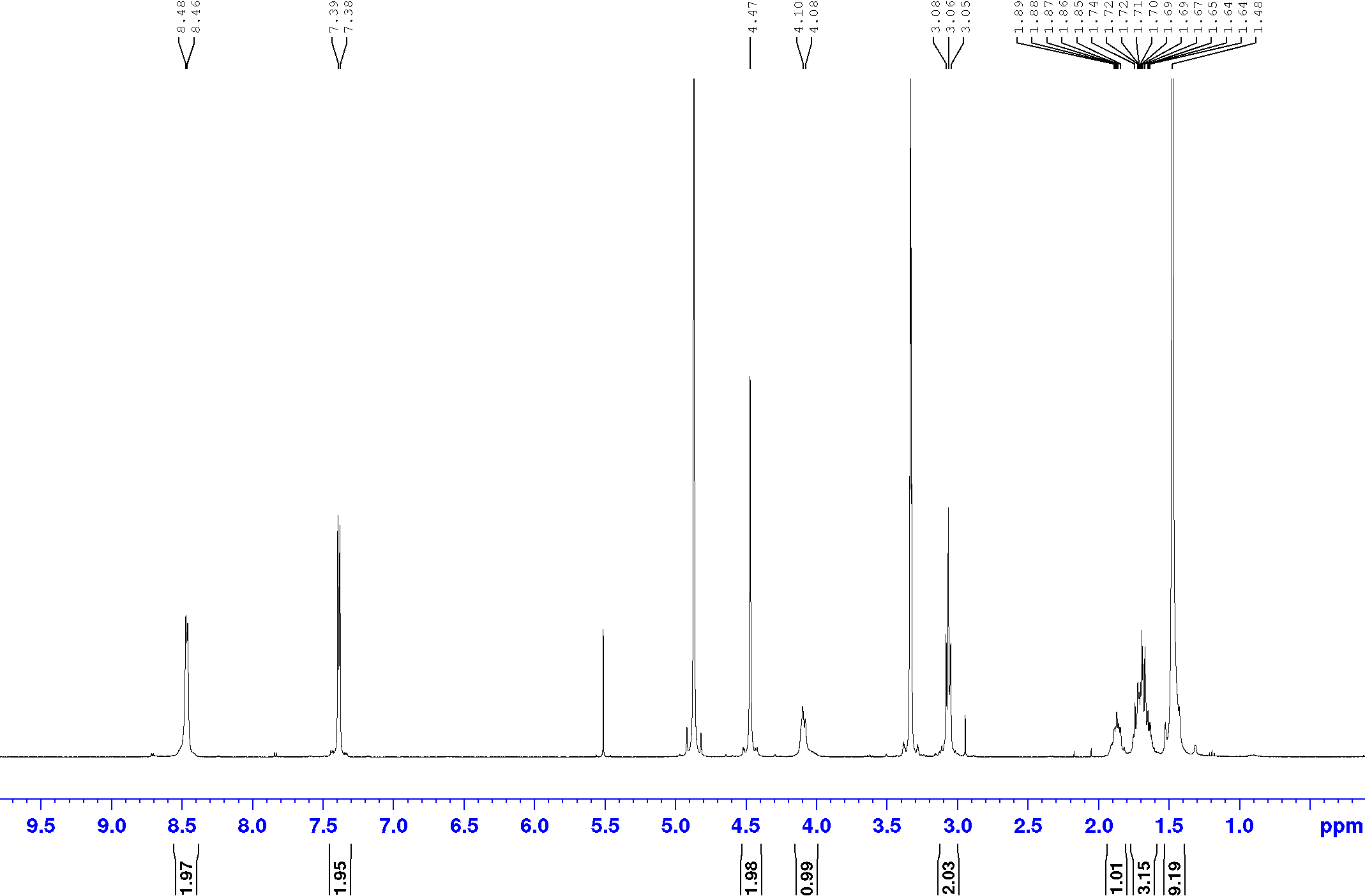


**Compound 9.** ^13^C NMR, CD_3_OD, 100 MHz


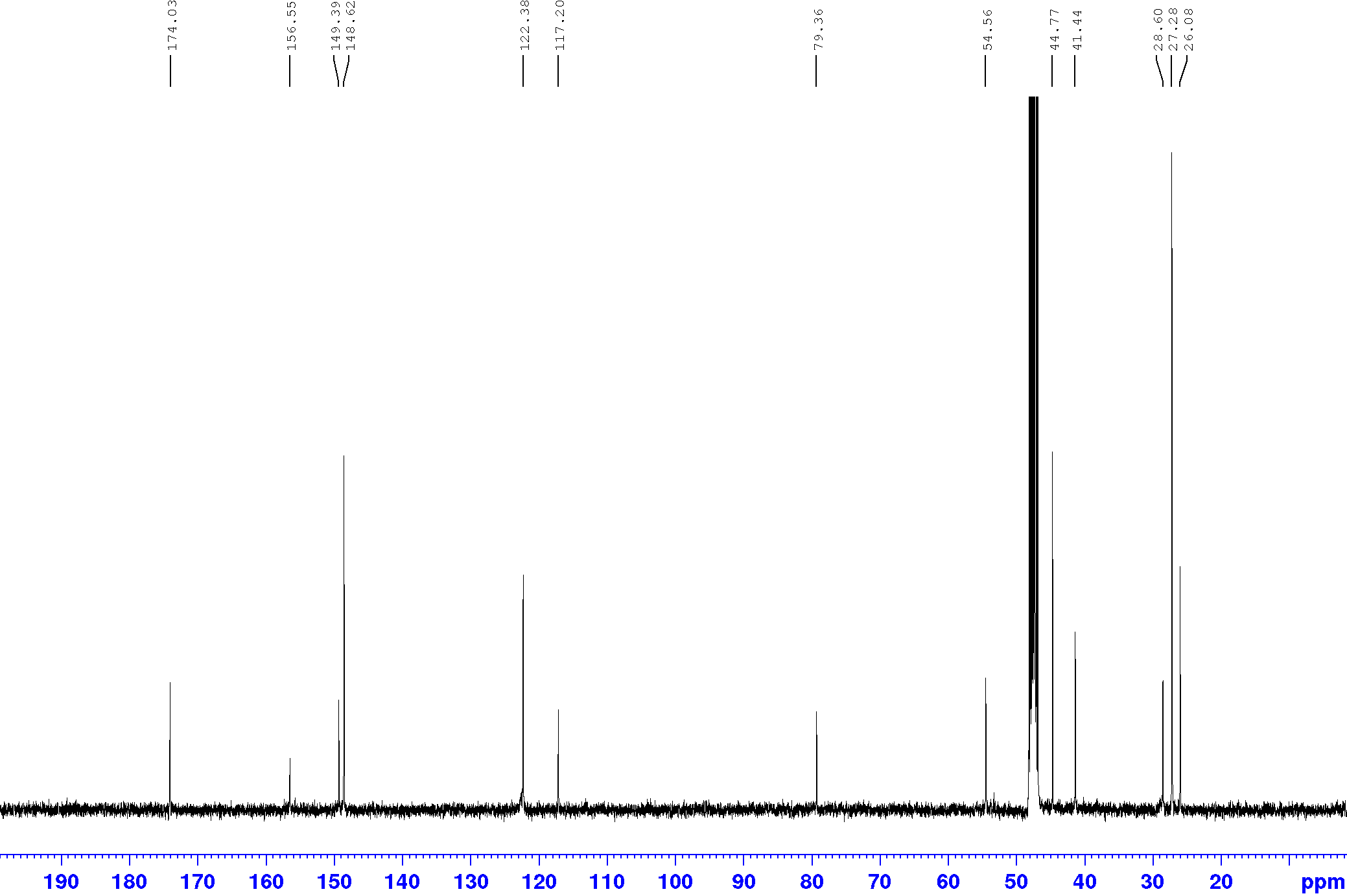


**Compound 10.** ^1^H NMR, CD_3_OD, 400 MHz


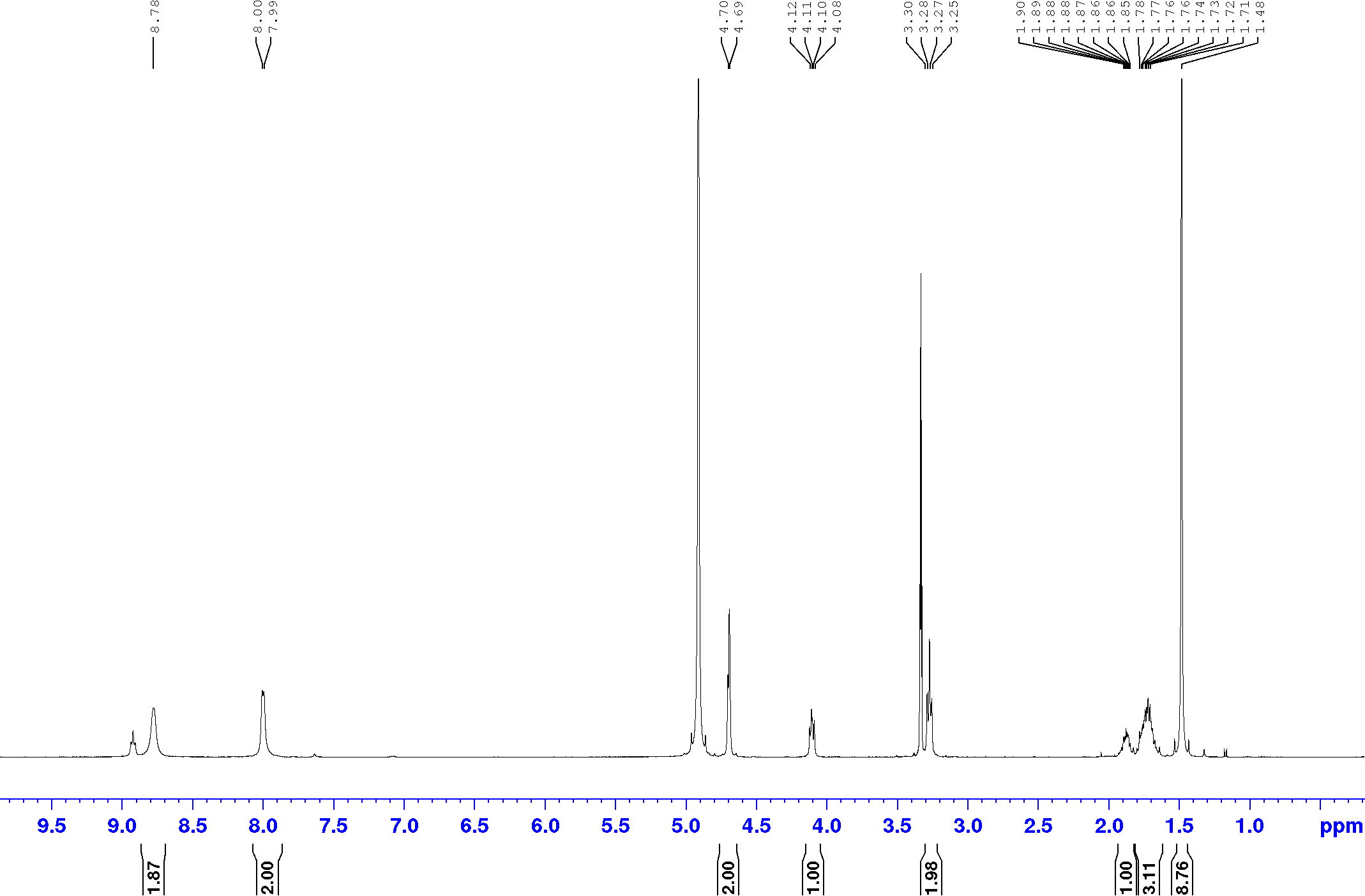


**Compound 10.** ^13^C NMR, CD_3_OD, 100 MHz


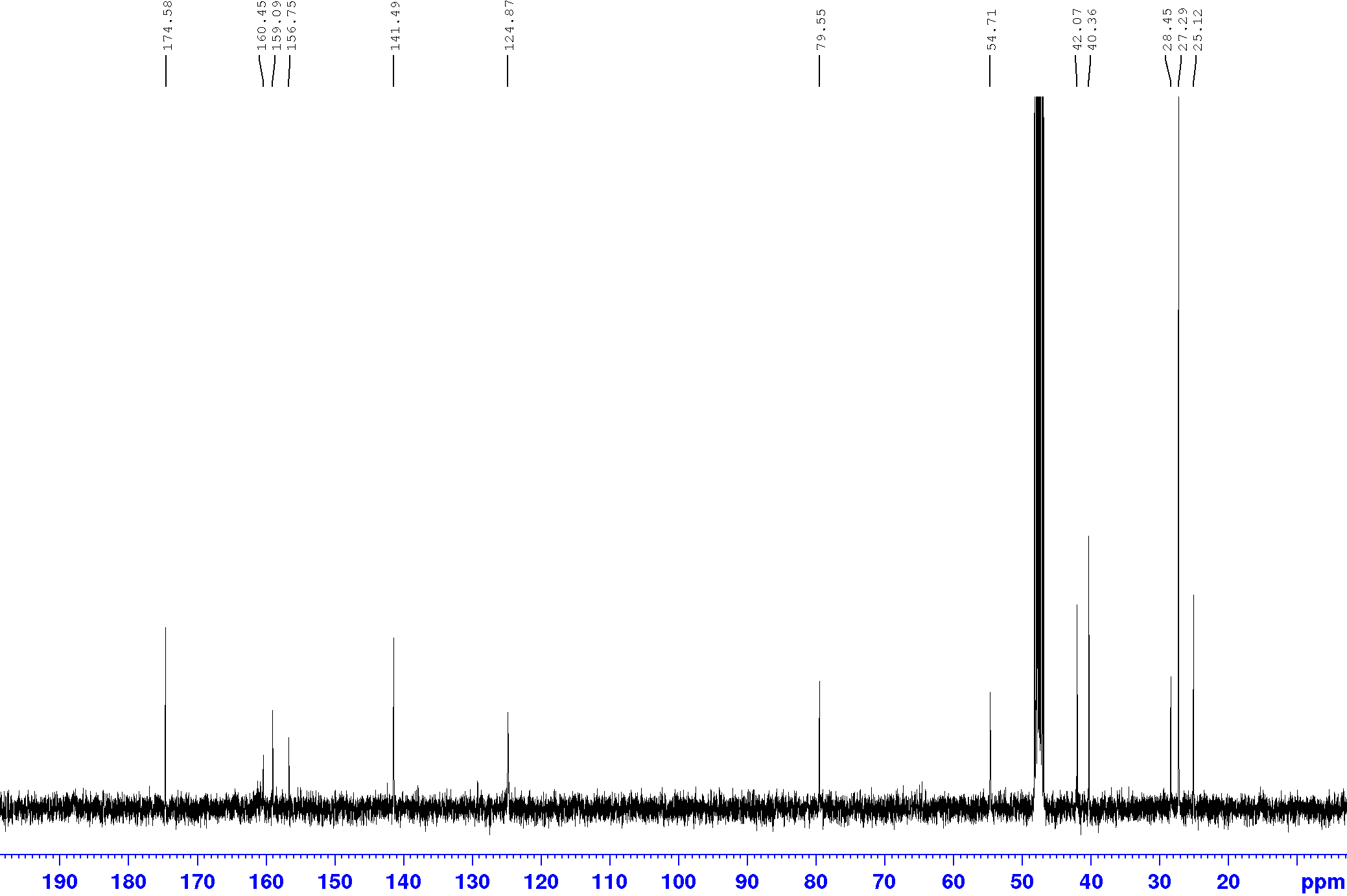


**Compound 11a.** ^1^H NMR, CD_3_OD, 400 MHz


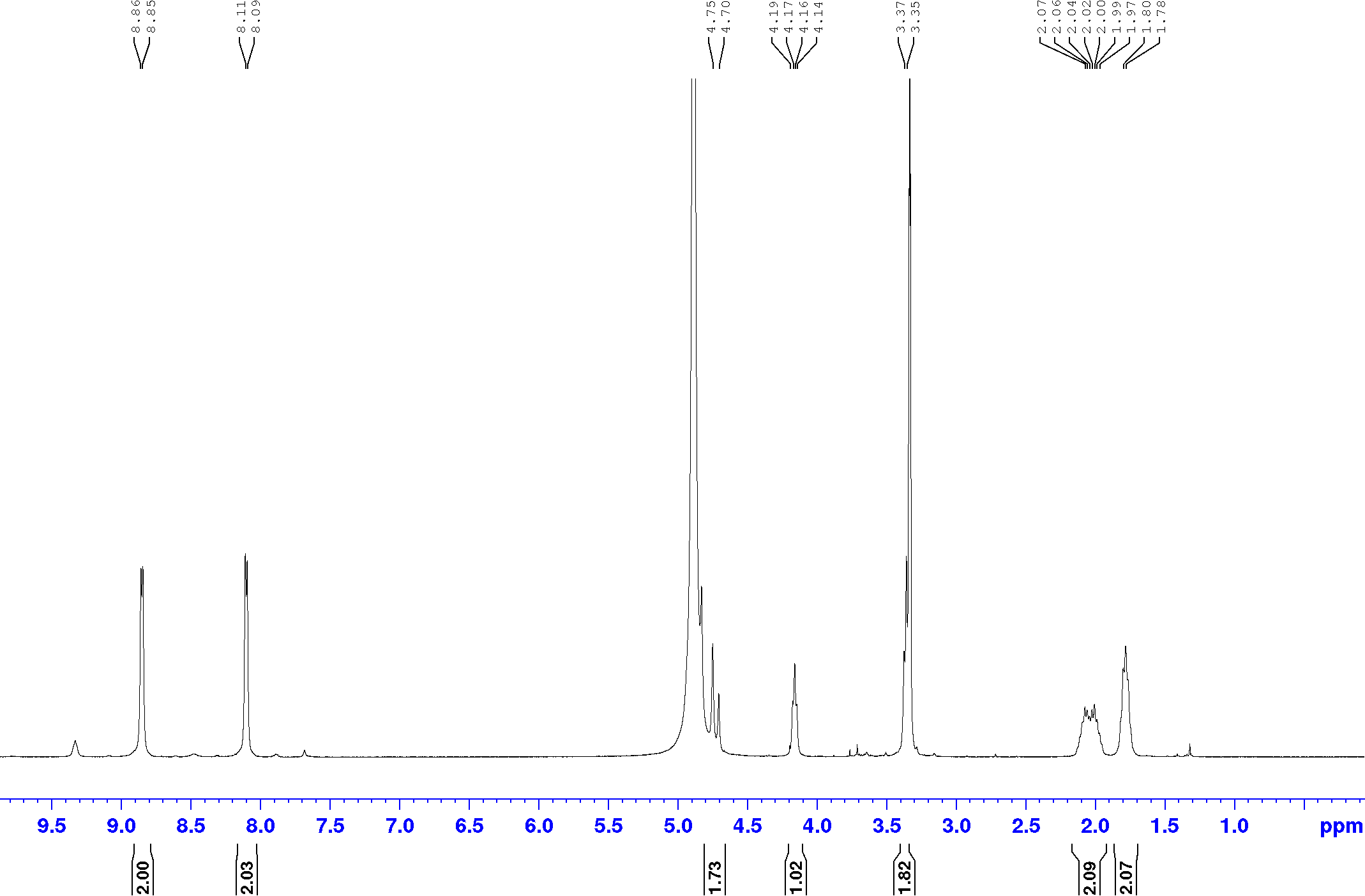


**Compound 11a.** ^13^C NMR, CD_3_OD, 100 MHz


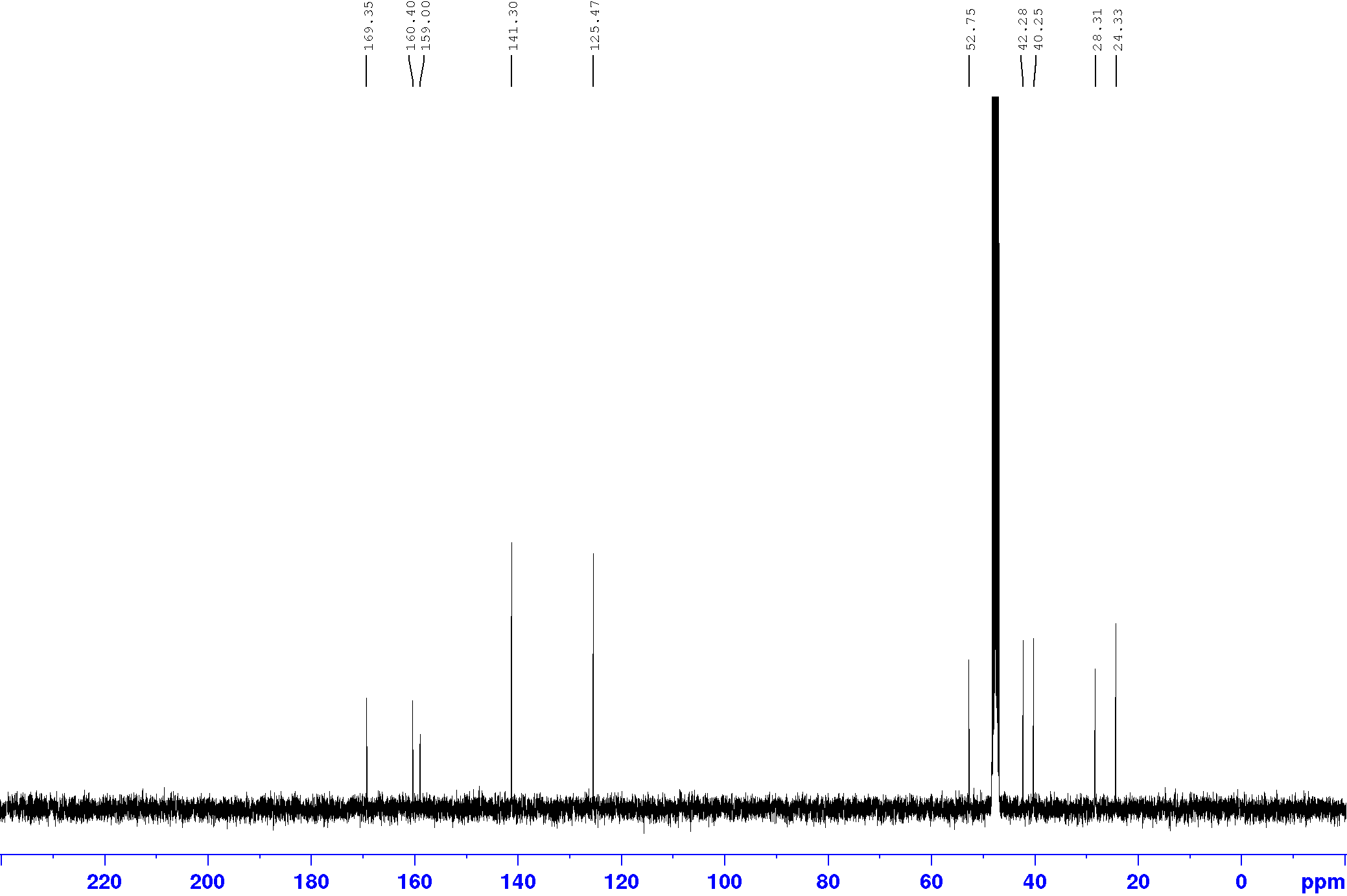


**Compound 13.** ^1^H NMR, CD_3_OD, 400 MHz


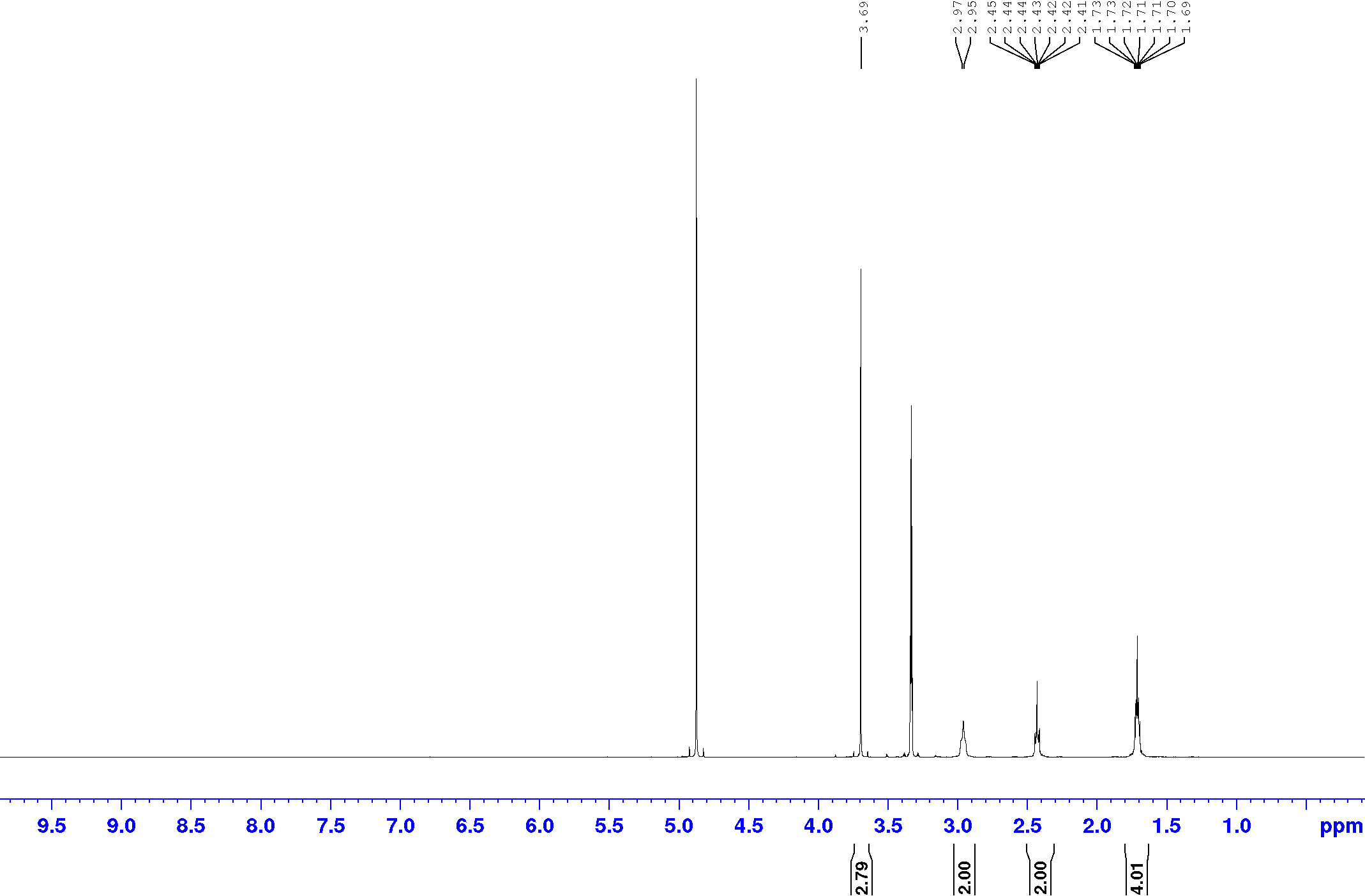


**Compound 13.** ^13^C NMR, CD_3_OD, 100 MHz


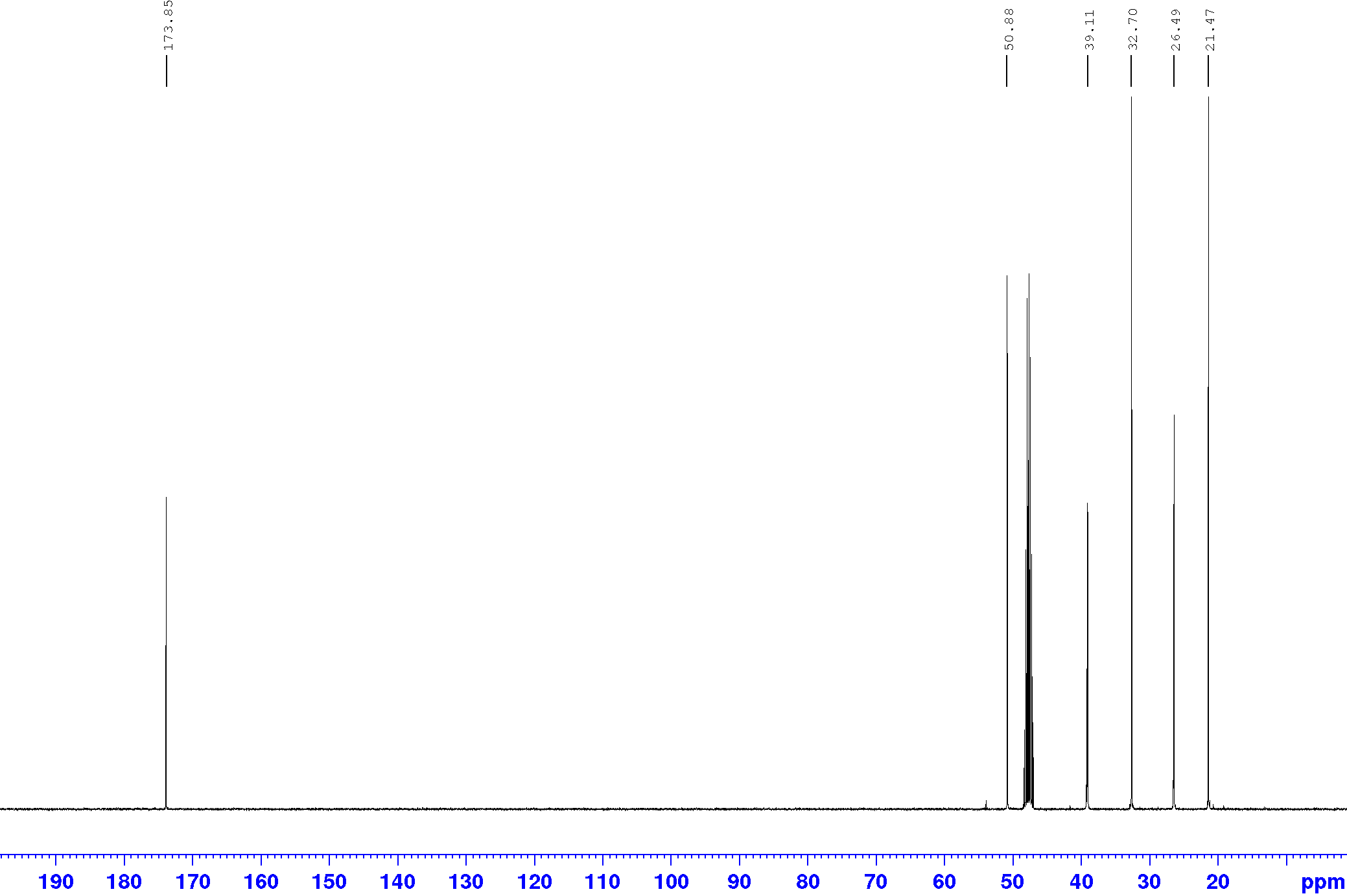


**Compound 14.** ^1^H NMR, CDCl_3_, 400 MHz


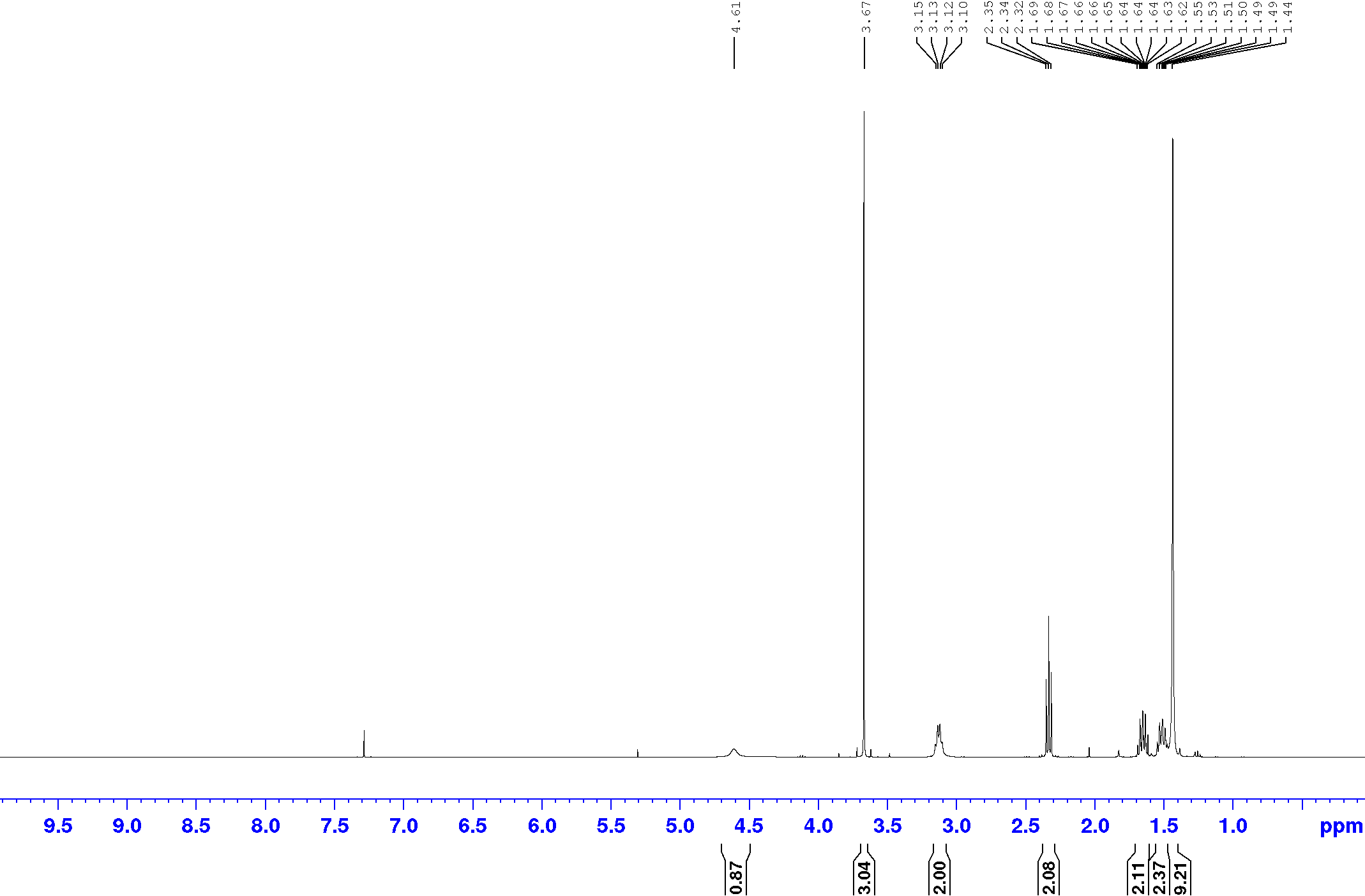


**Compound 14.** ^13^C NMR, CDCl_3_, 100 MHz


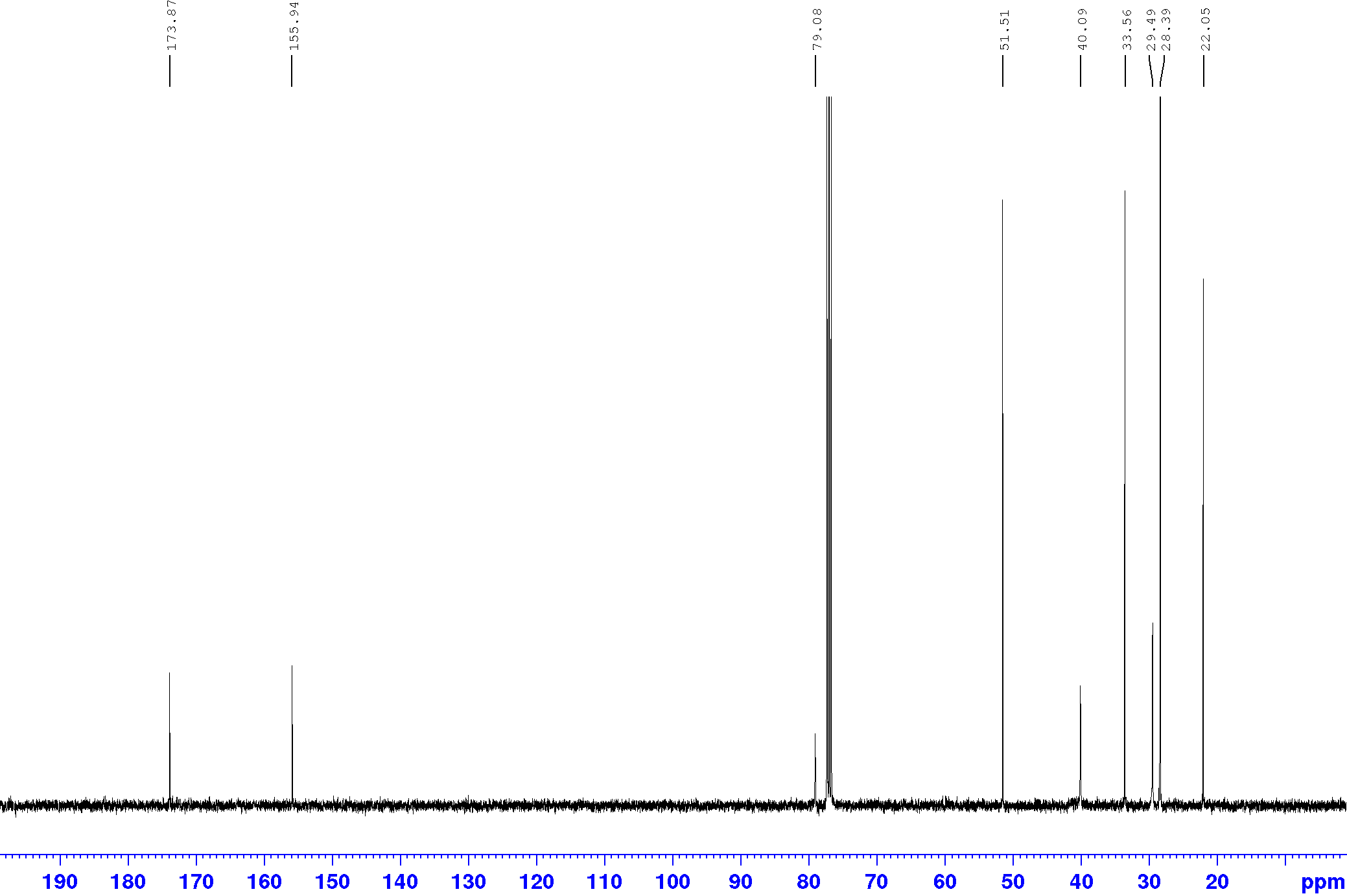


**Compound 15.** ^1^H NMR, CD_3_OD, 400 MHz


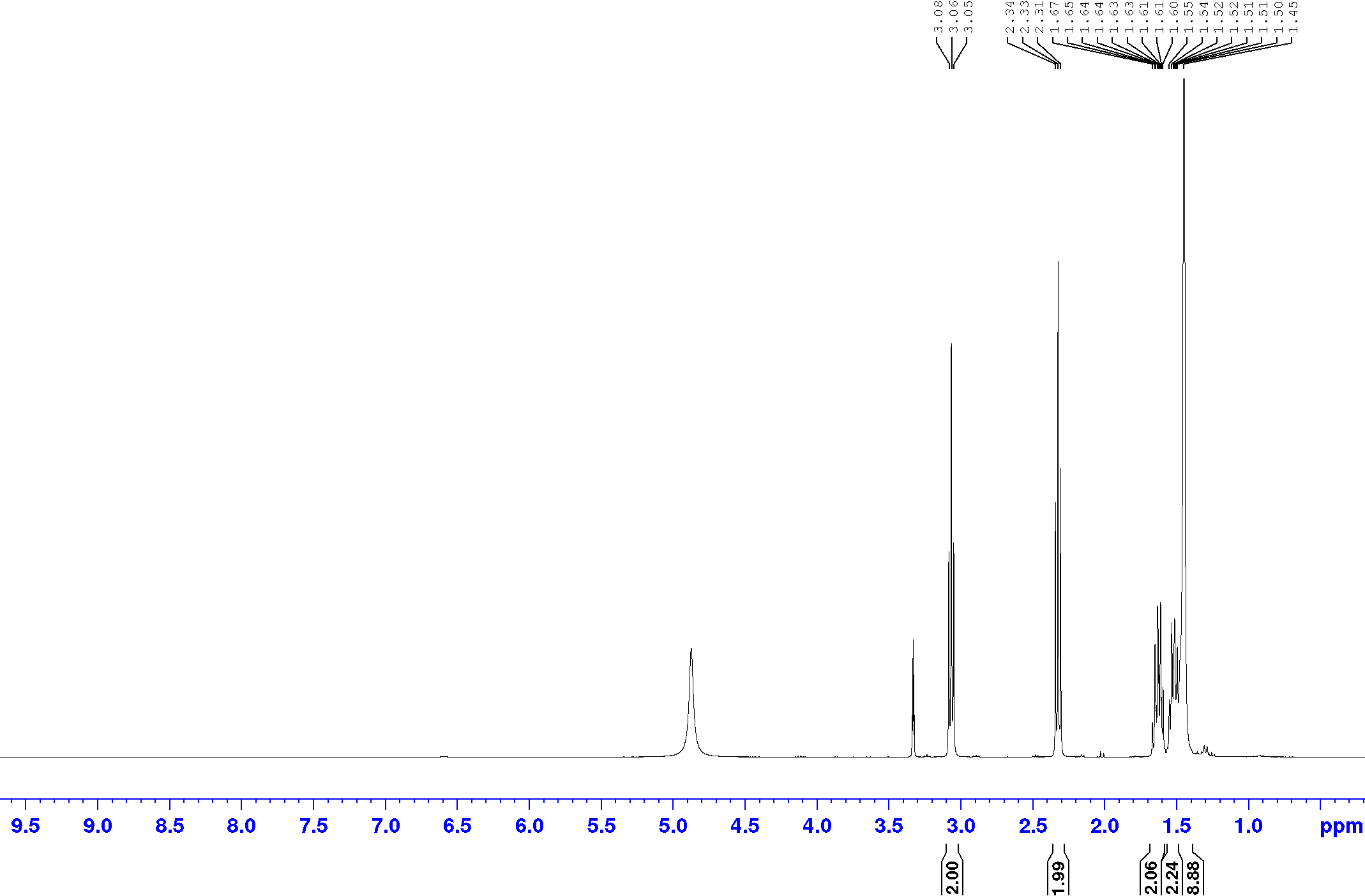


**Compound 15.** ^13^C NMR, CD_3_OD, 100 MHz


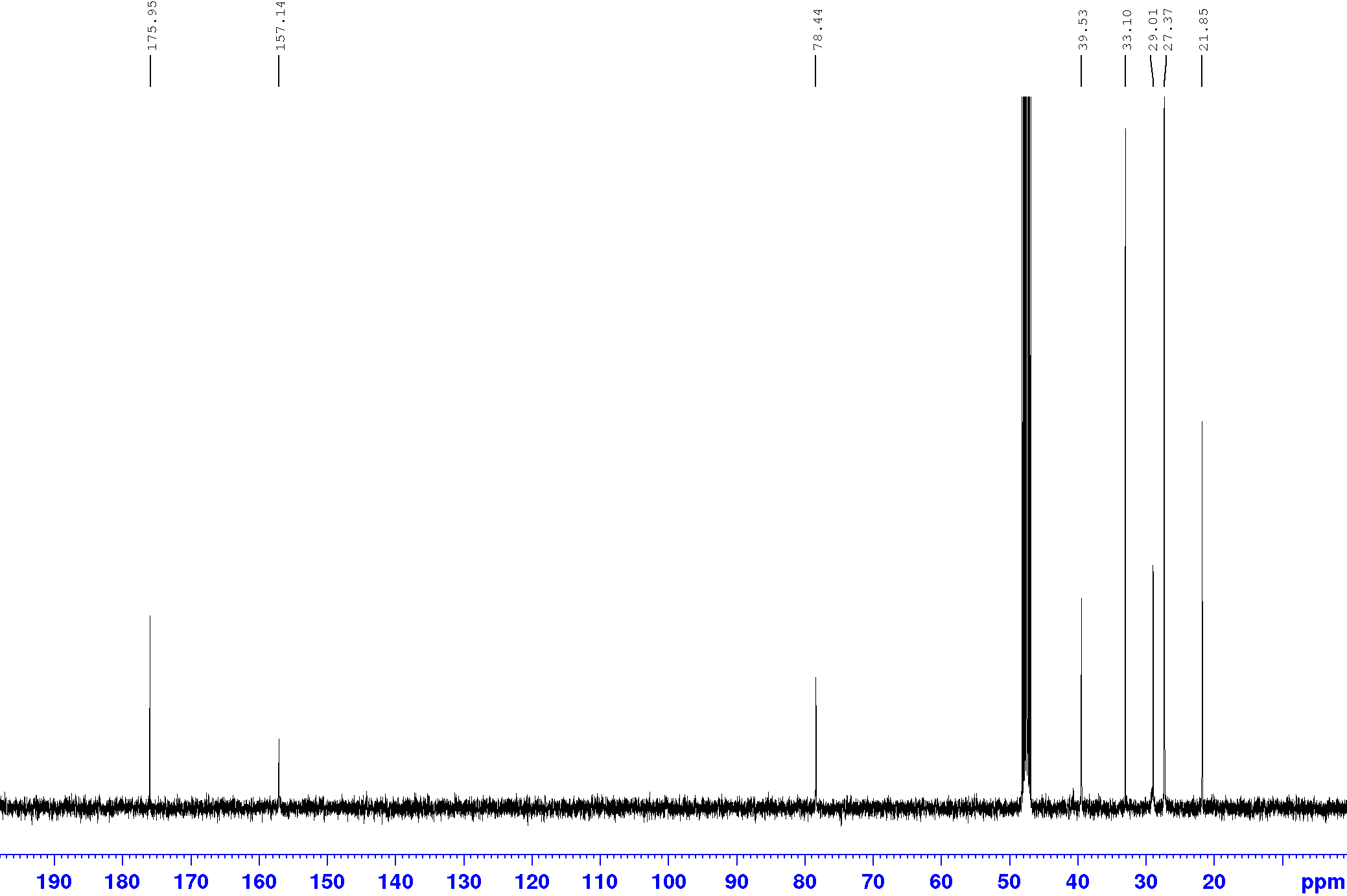


**Compound 16.** ^1^H NMR, CDCl_3_, 400 MHz


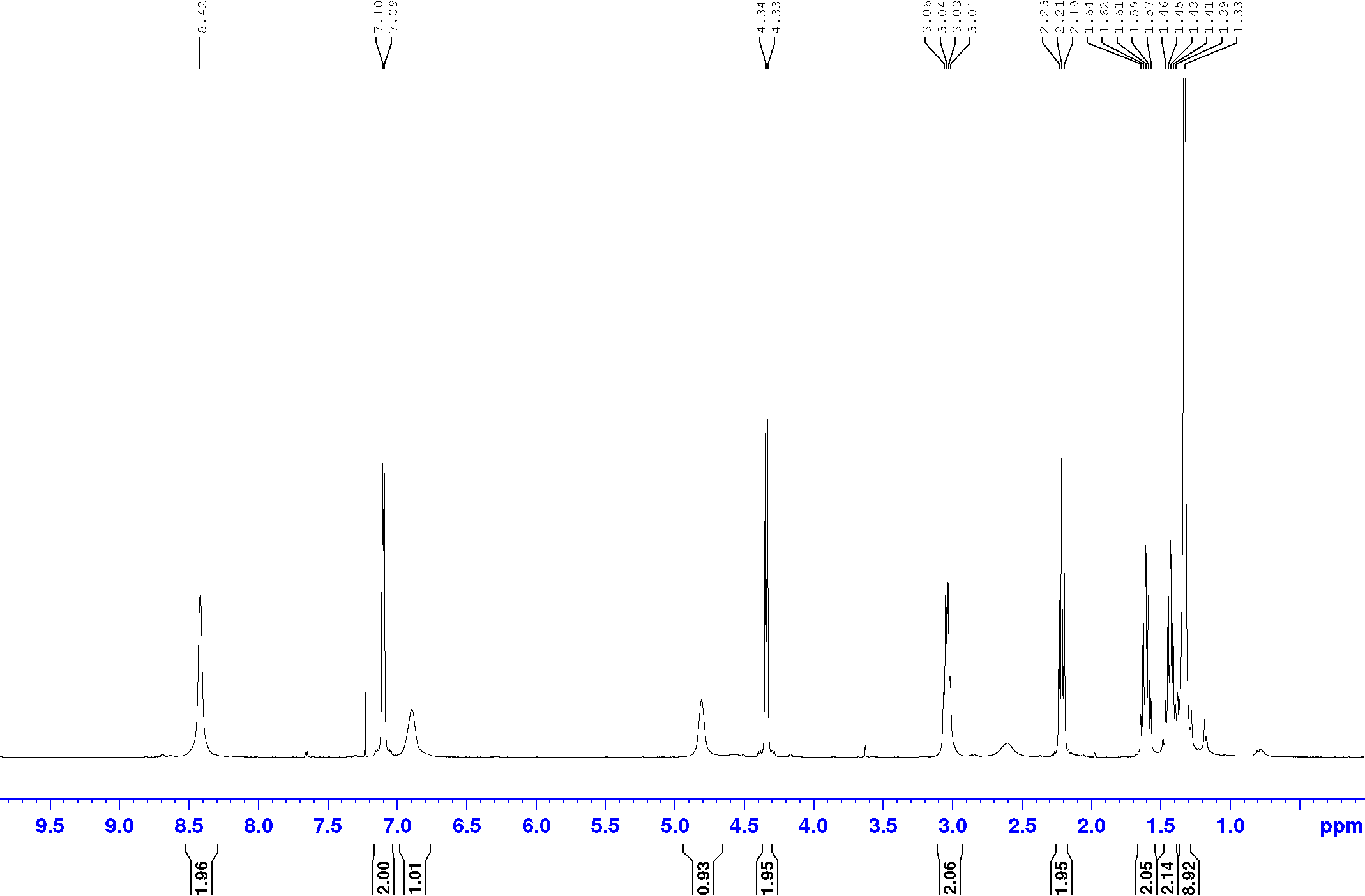


**Compound 16.** ^13^C NMR, CDCl_3_, 100 MHz


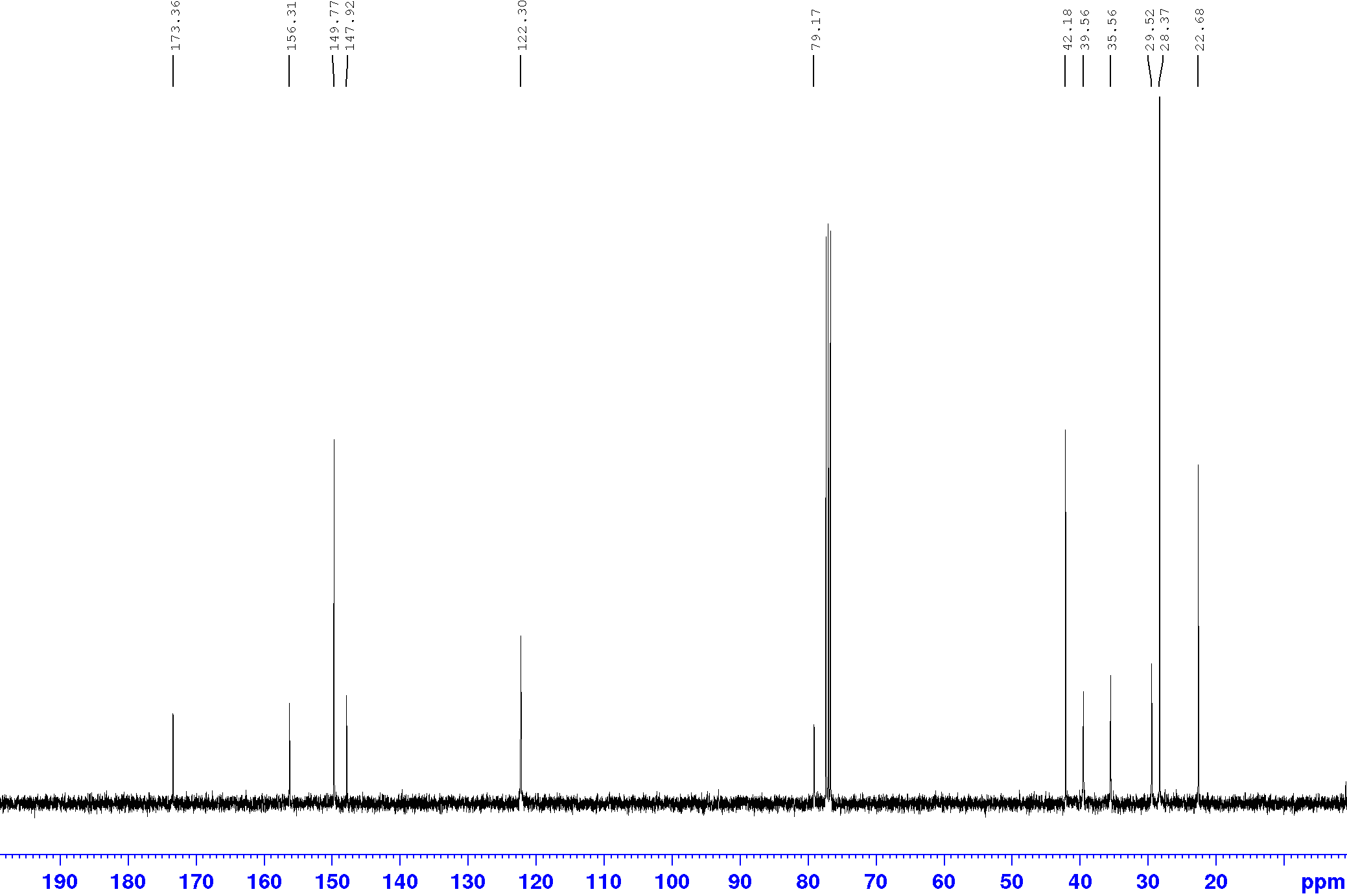


**Compound 17.** ^1^H NMR, DMSO-d_6_, 400 MHz


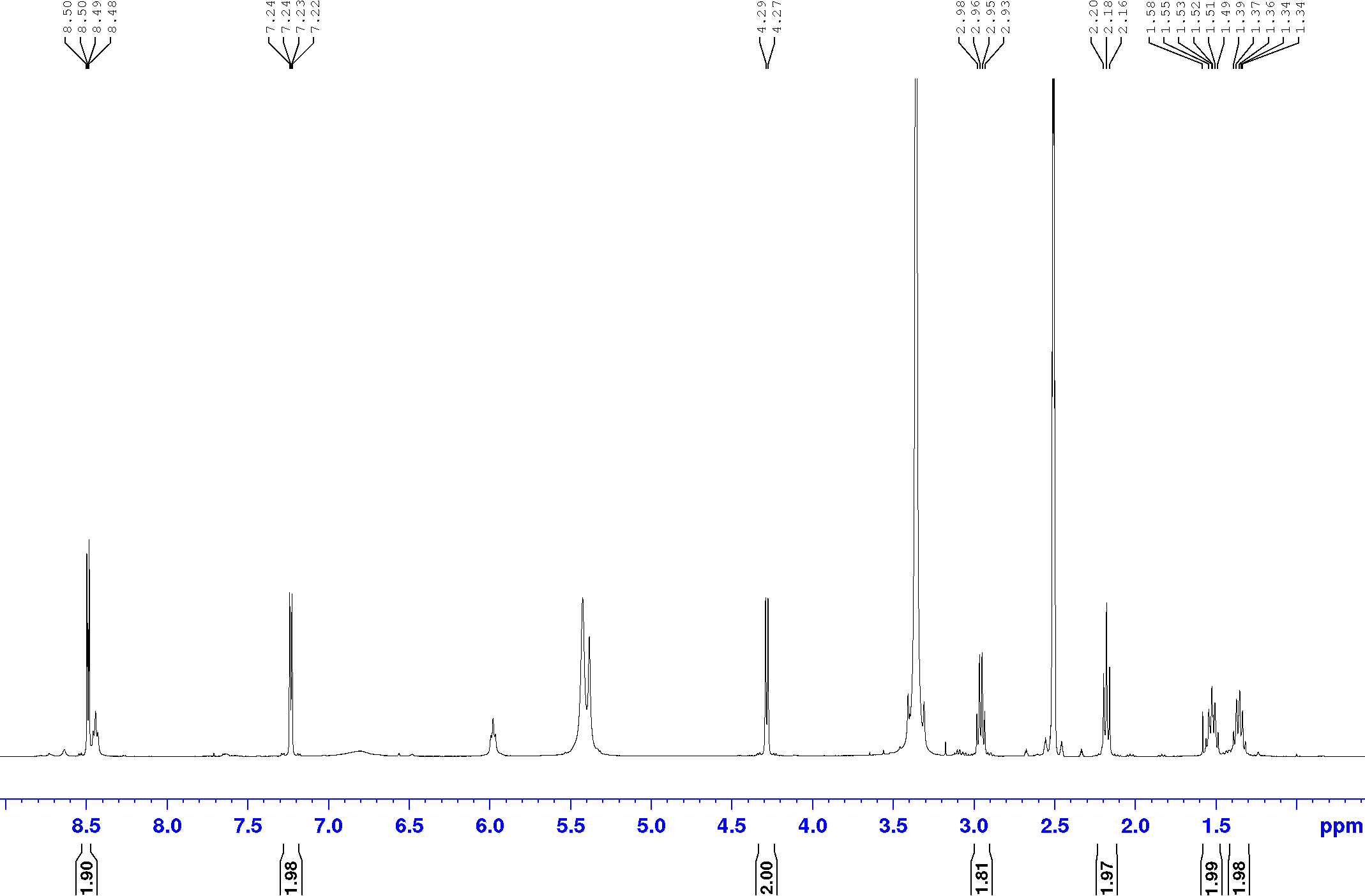


**Compound 17.** ^13^C NMR, DMSO-d_6_, 100 MHz


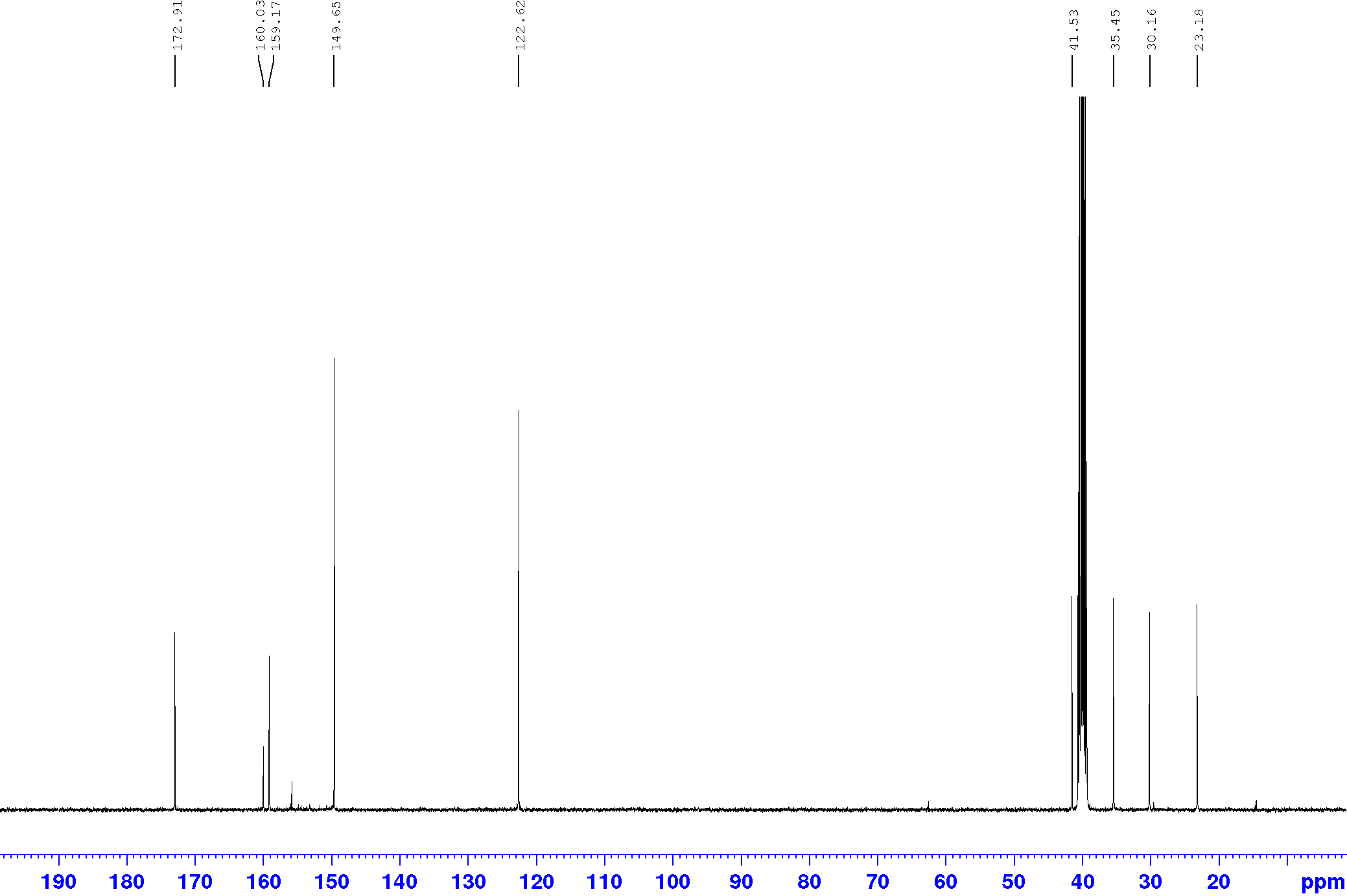


**Compound 18.** ^1^H NMR, CD_3_OD, 400 MHz


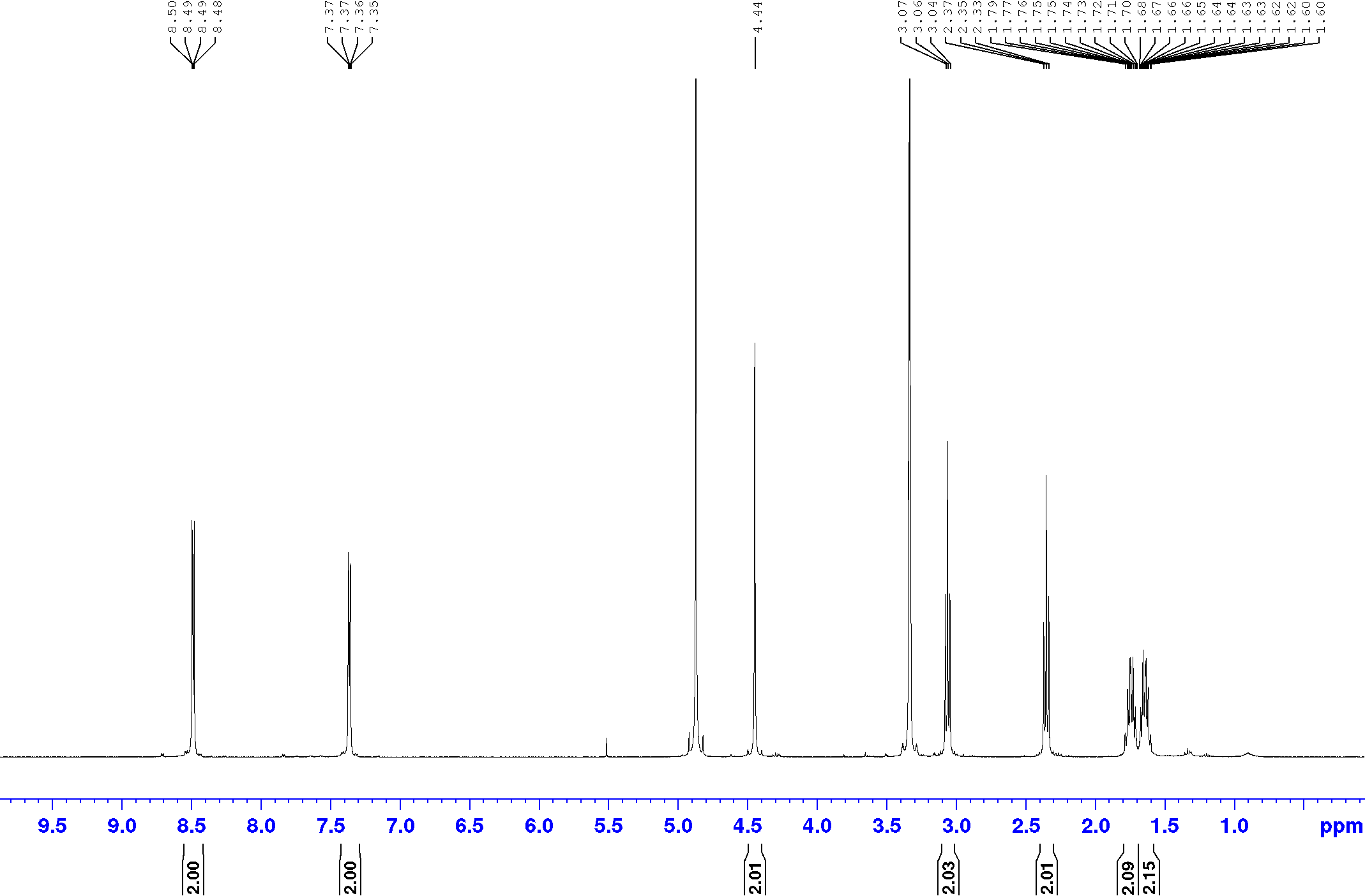


**Compound 18.** ^13^C NMR, CD_3_OD, 100 MHz


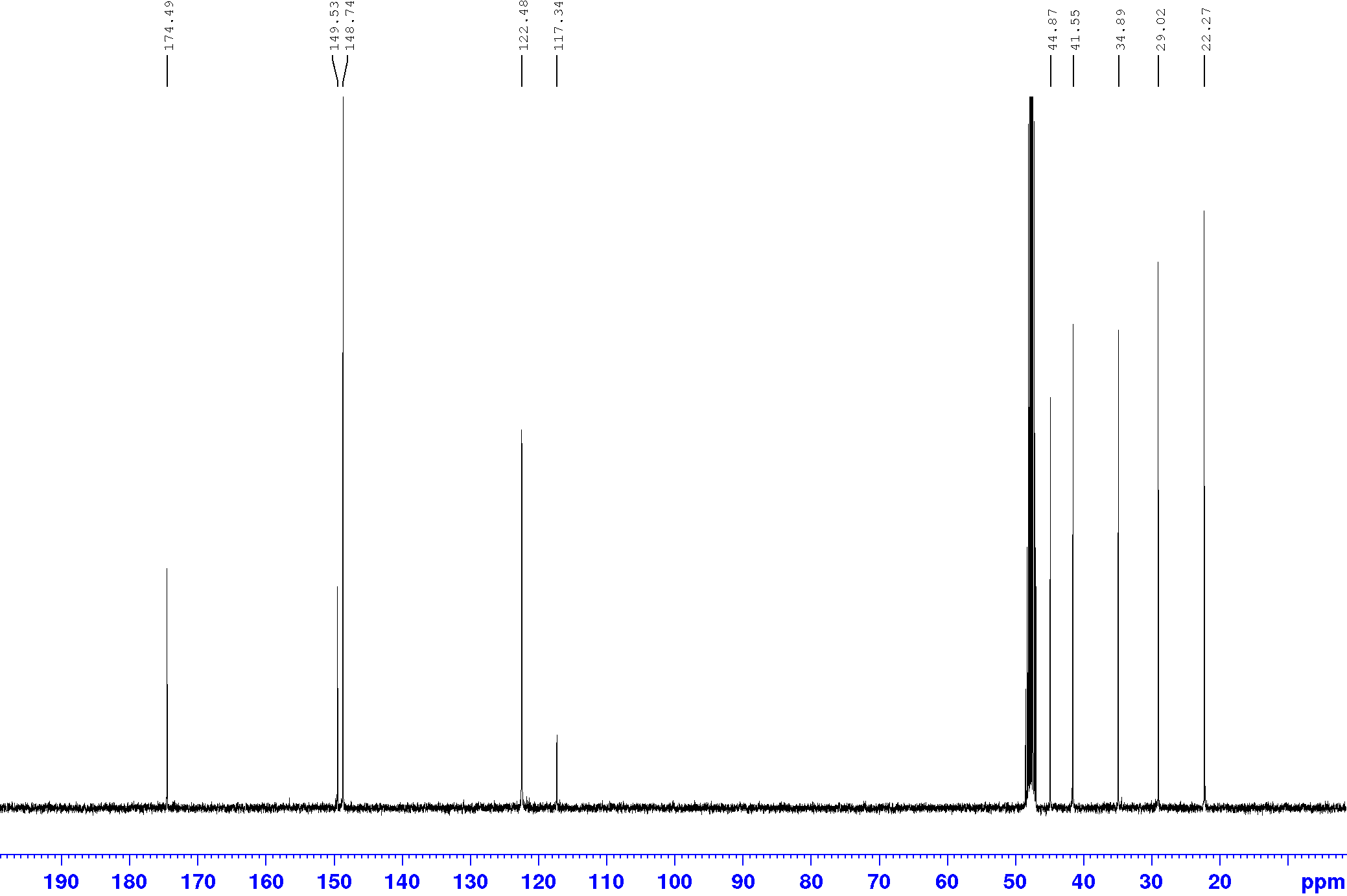


**Compound 11b.** ^1^H NMR, D_2_O, 400 MHz


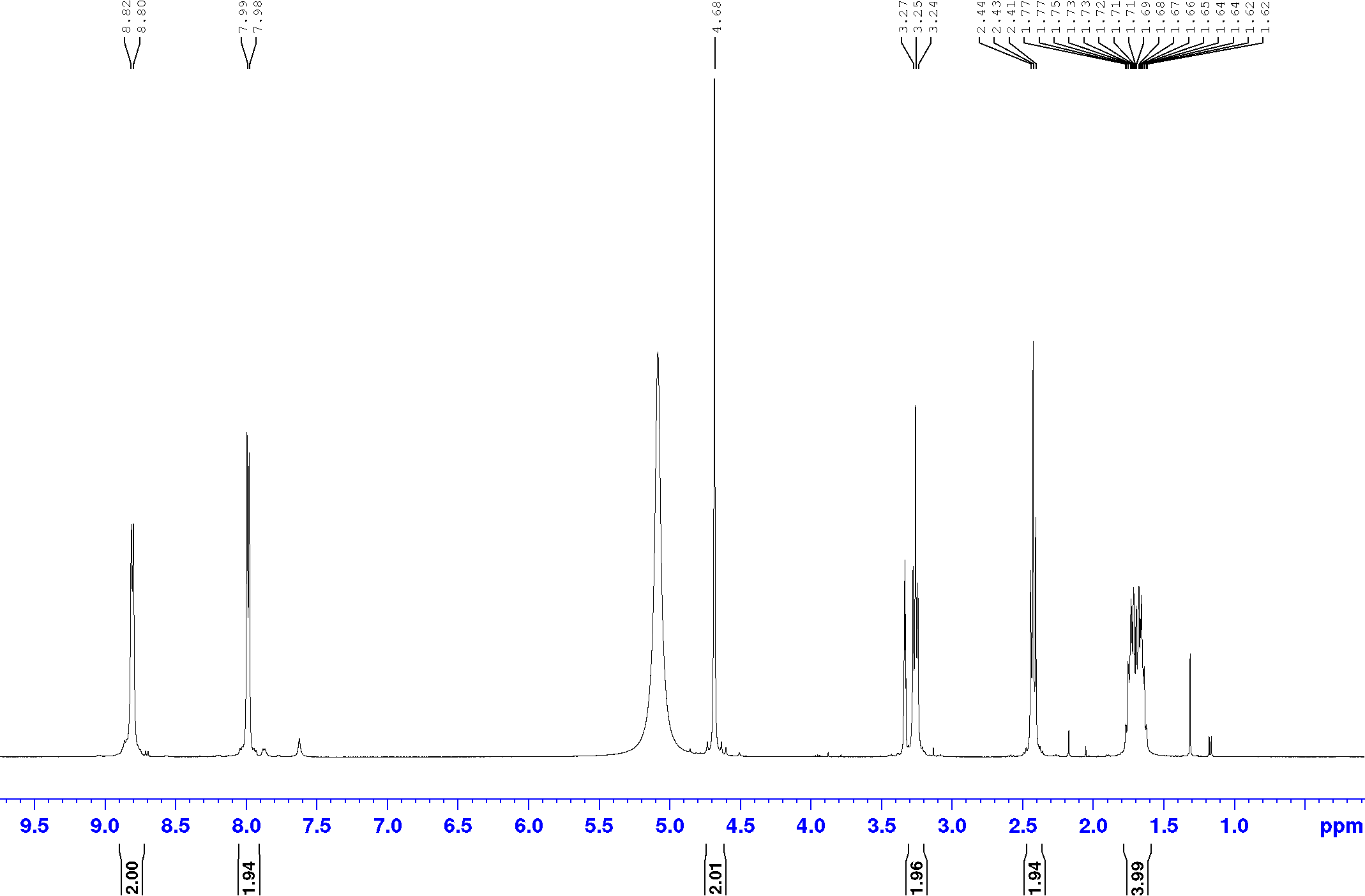


**Compound 11b.** ^13^C NMR, CD_3_OD, 100 MHz


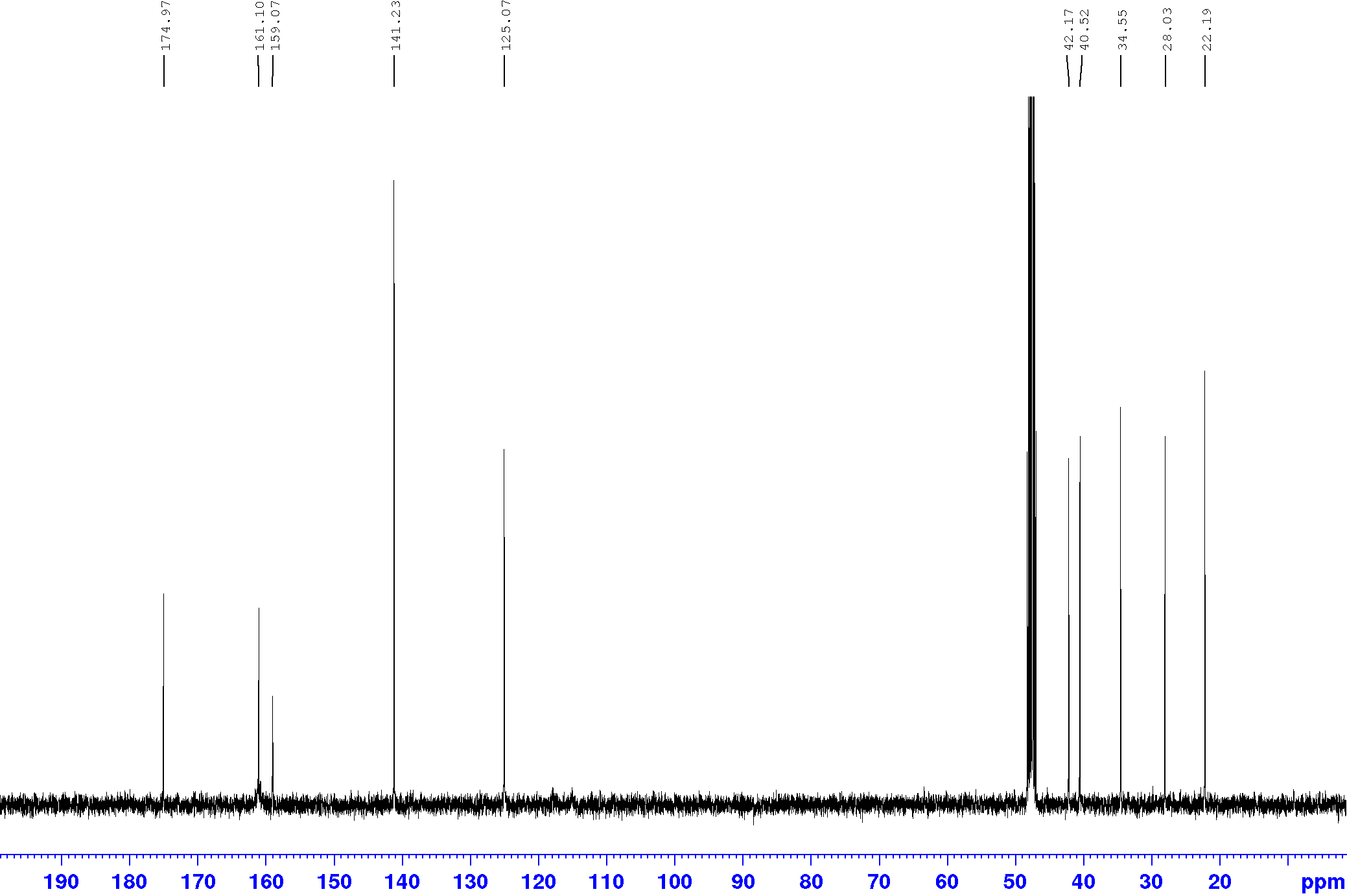


**Compound 19.** ^1^H NMR, CD_3_OD, 400 MHz


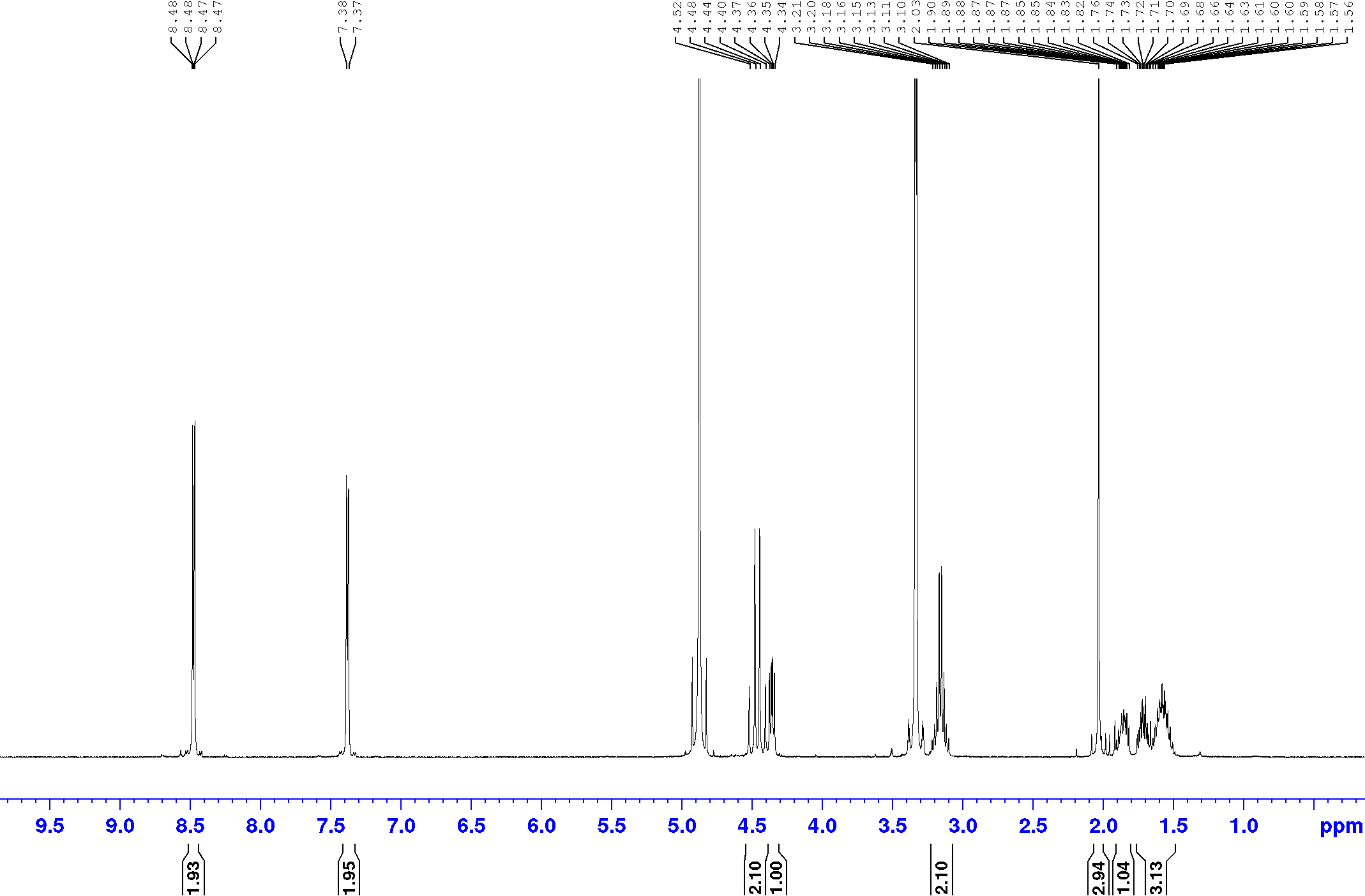


**Compound 19.** ^13^C NMR, CD_3_OD, 100 MHz


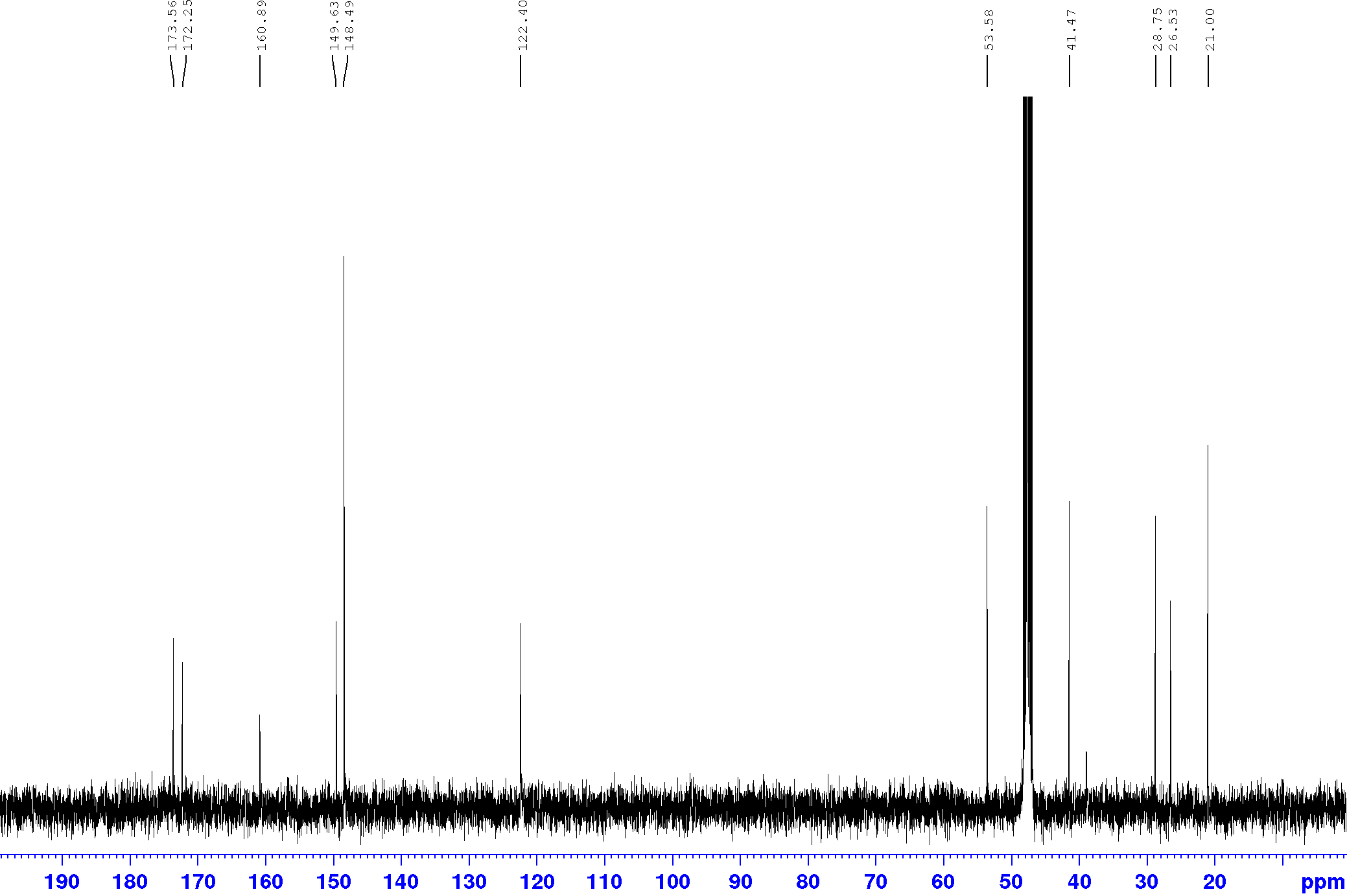


**Compound 20.** ^1^H NMR, CD_3_OD, 400 MHz


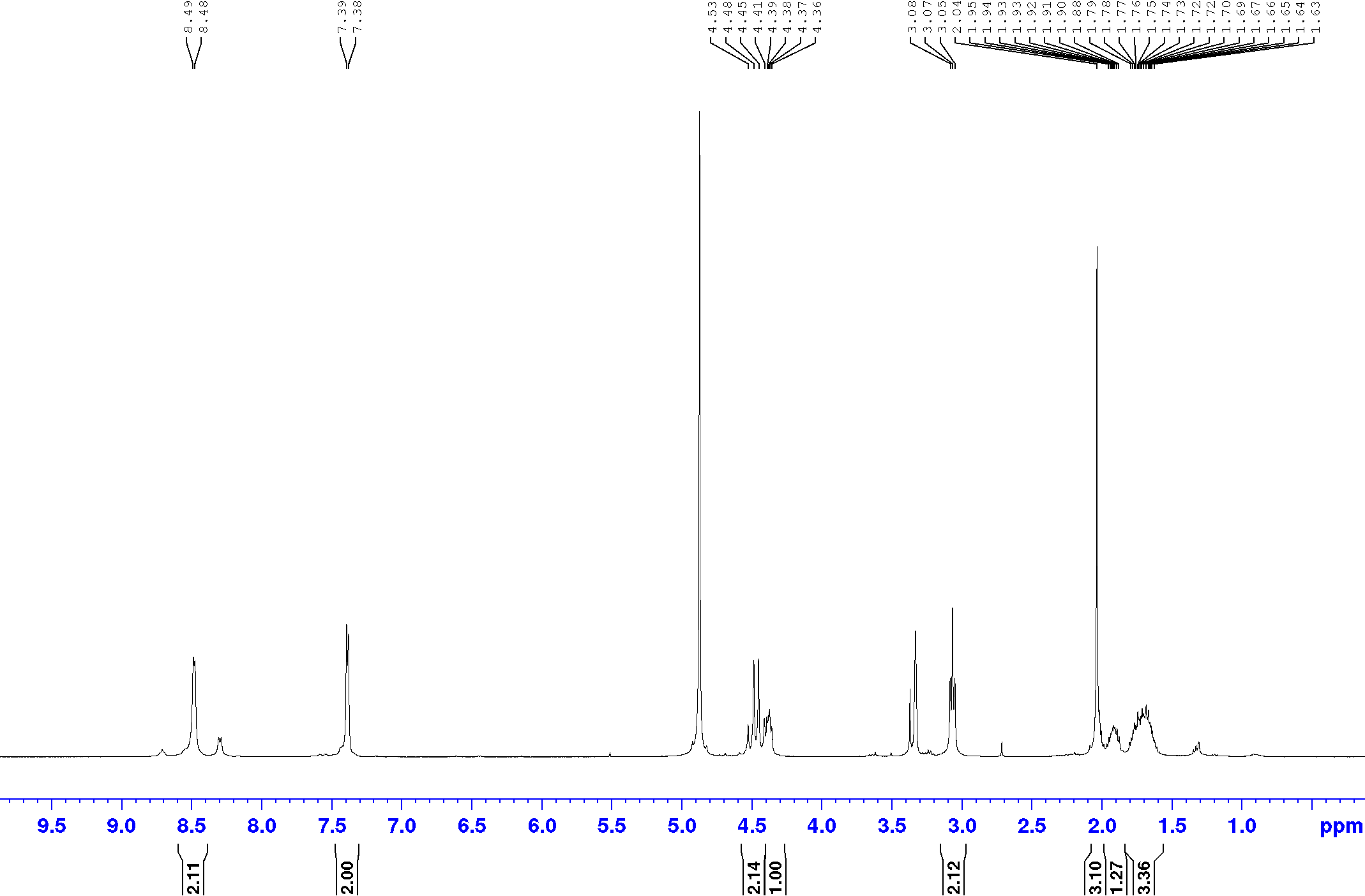


**Compound 20.** ^13^C NMR, CD_3_OD, 100 MHz


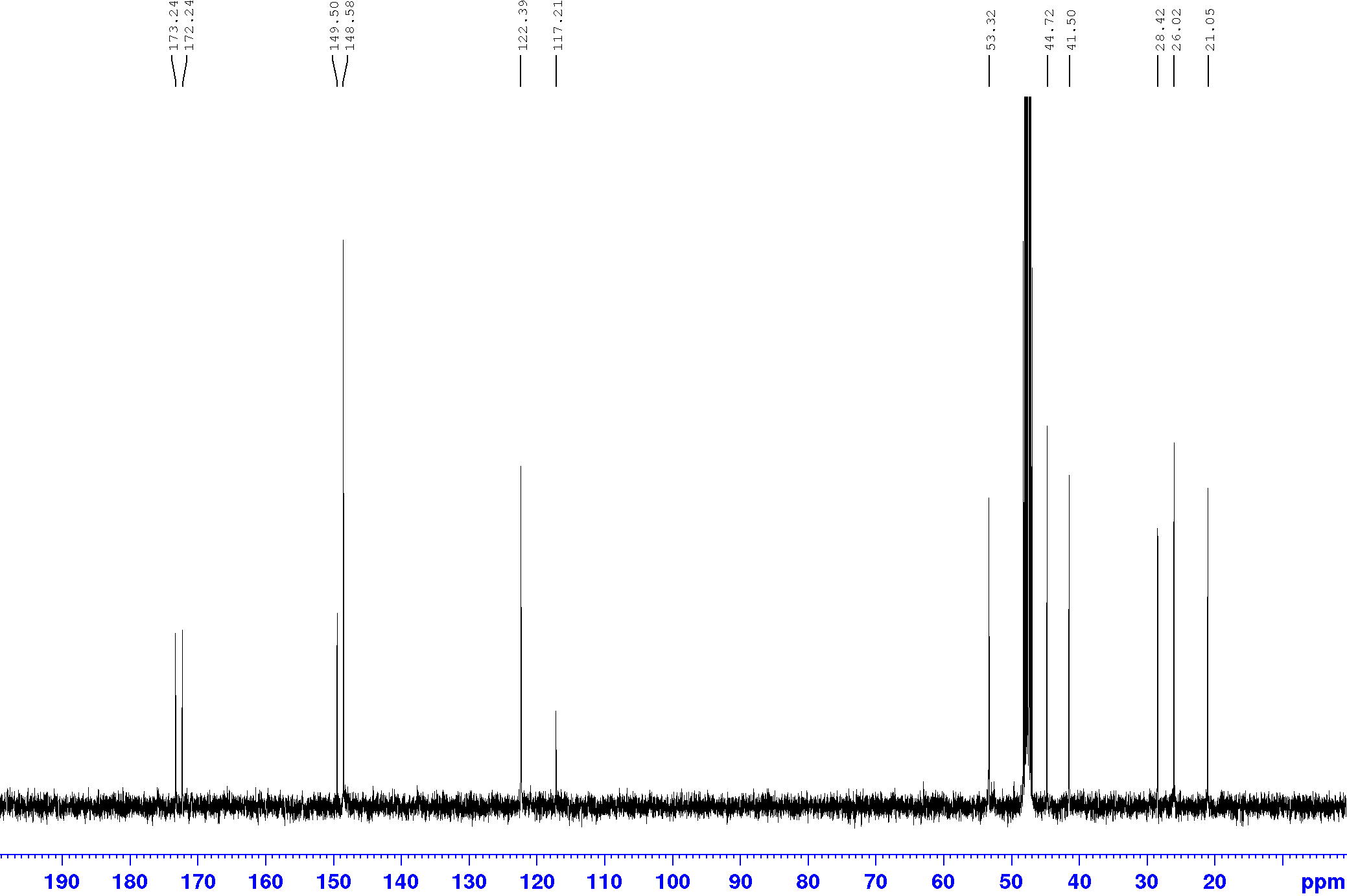


**Compound 11c.** ^1^H NMR, CD_3_OD, 400 MHz


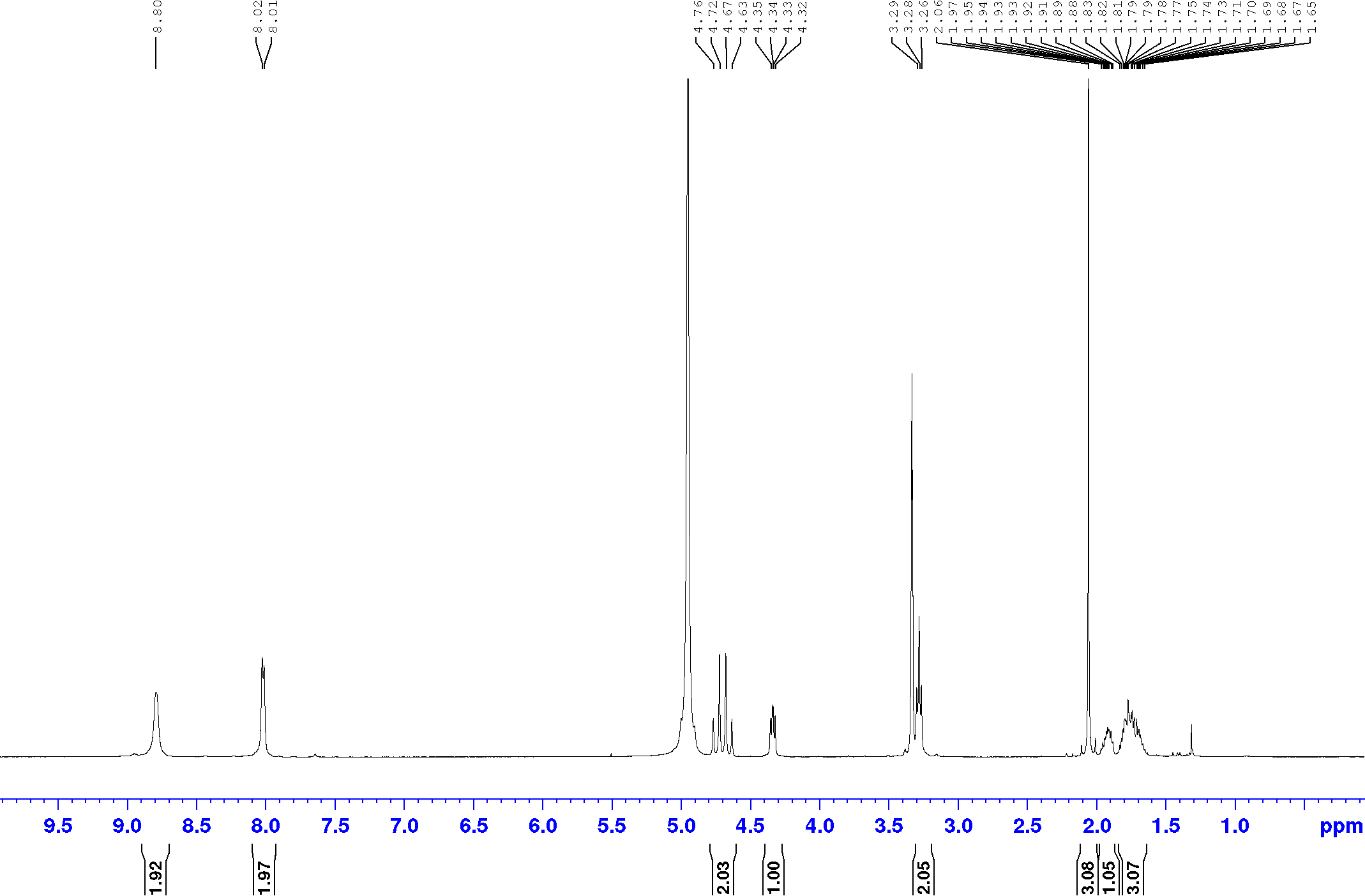


**Compound 11c.** ^13^C NMR, CD_3_OD, 100 MHz


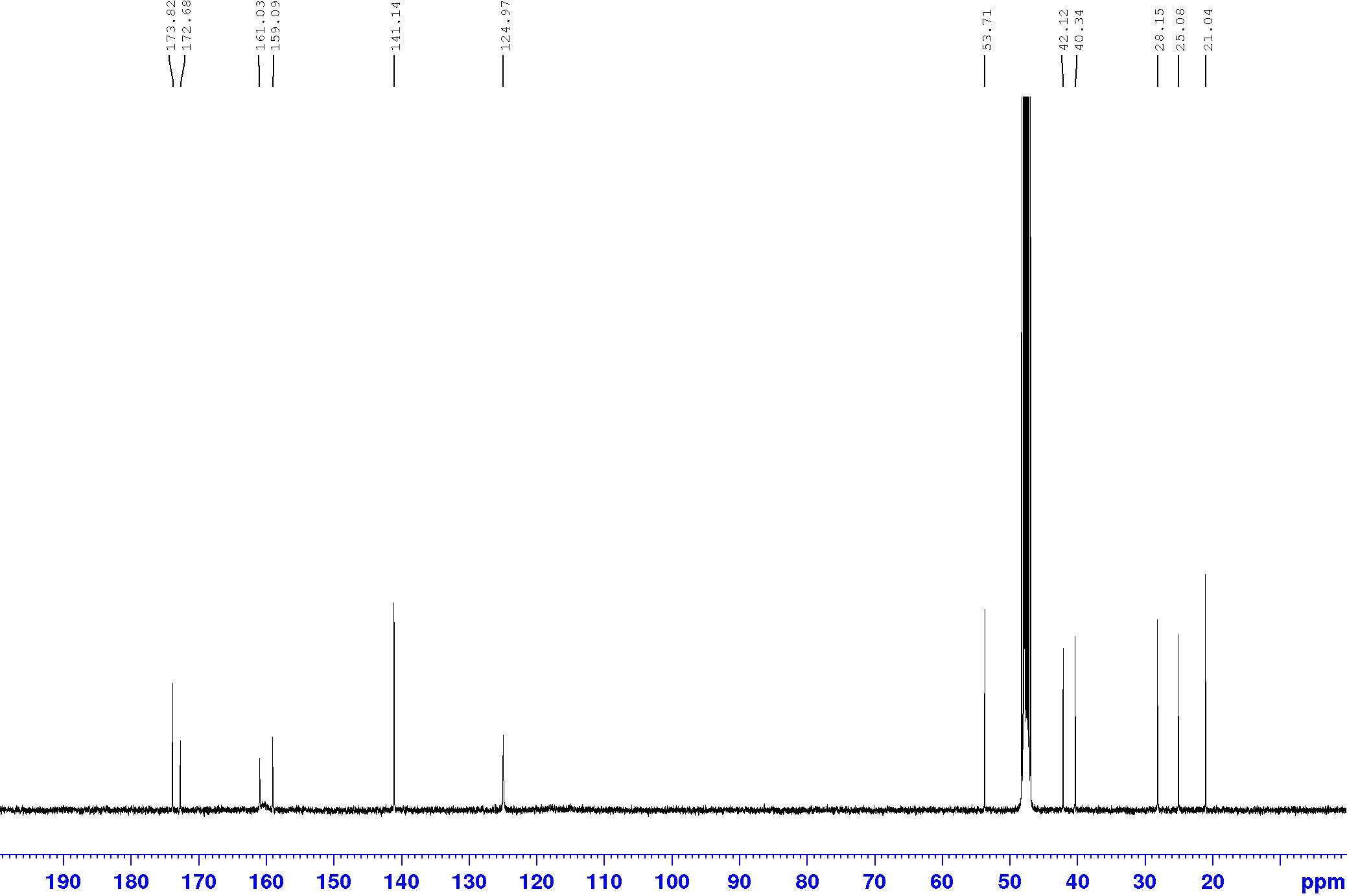


**Compound 21a.** ^1^H NMR, D_2_O, 400 MHz


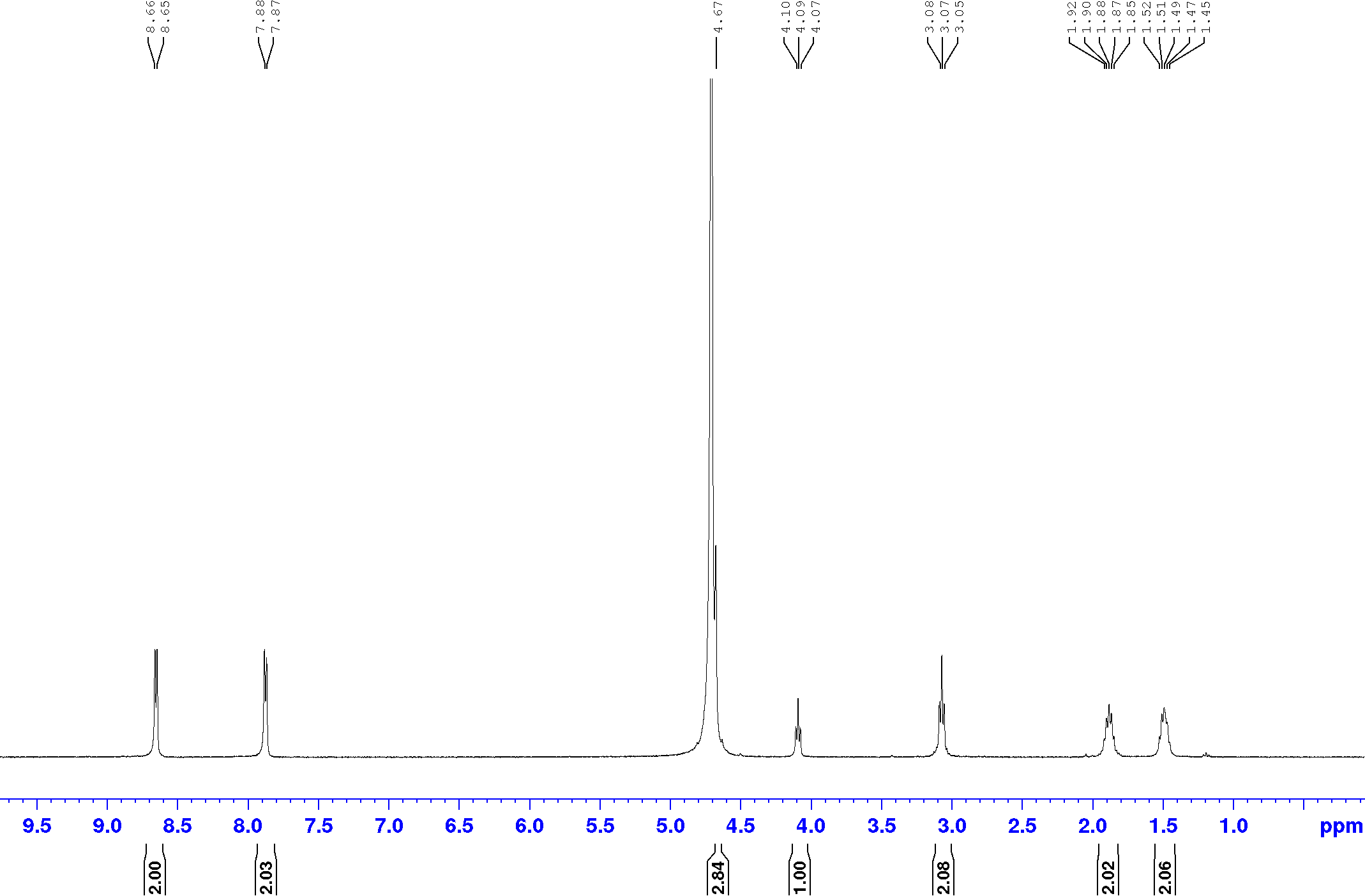


**Compound 21a.** ^13^C NMR, D_2_O, 100 MHz


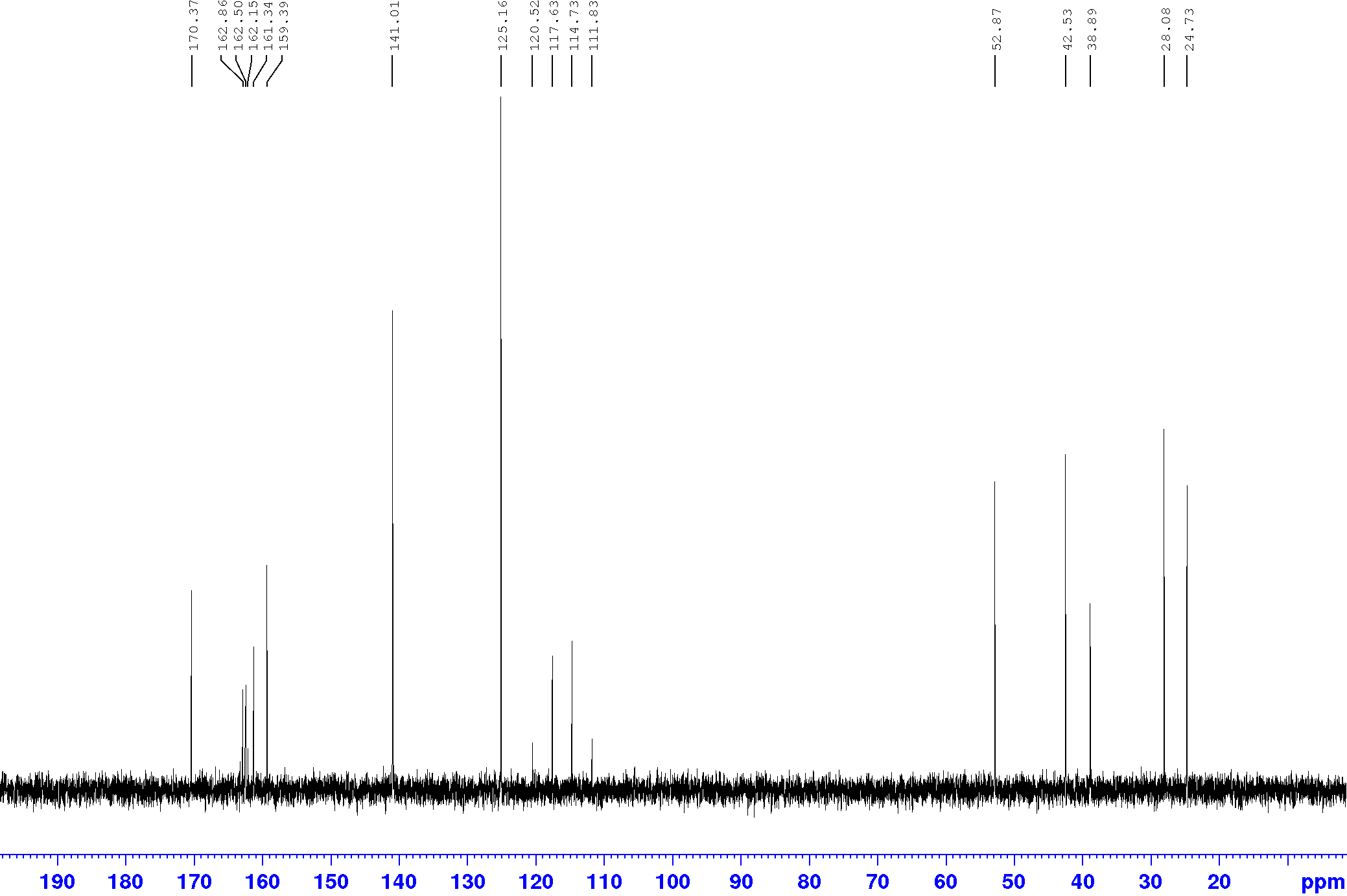


**Compound 22.** ^1^H NMR, CDCl_3_, 400 MHz


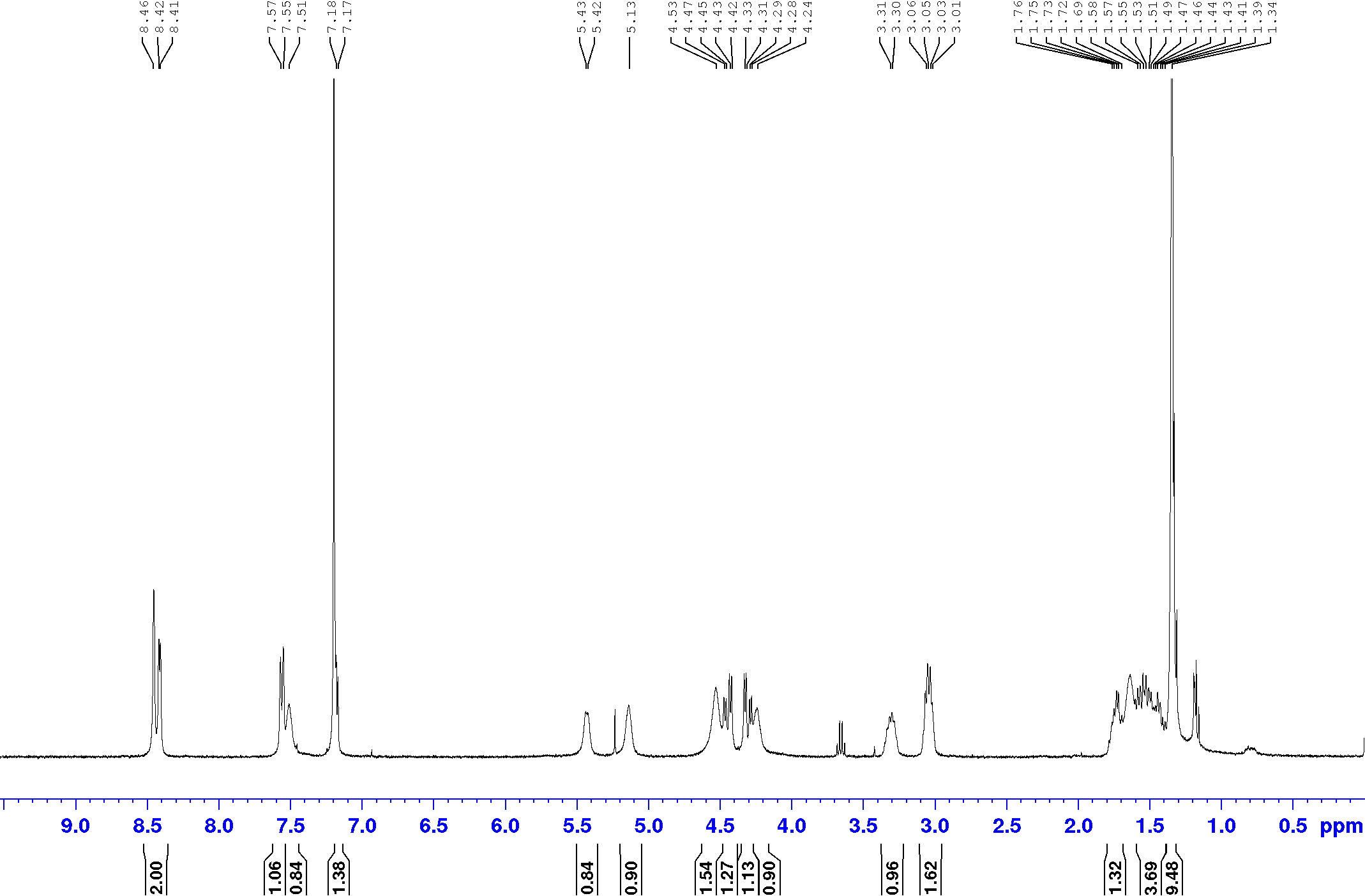


**Compound 22.** ^13^C NMR, CDCl_3_, 100 MHz


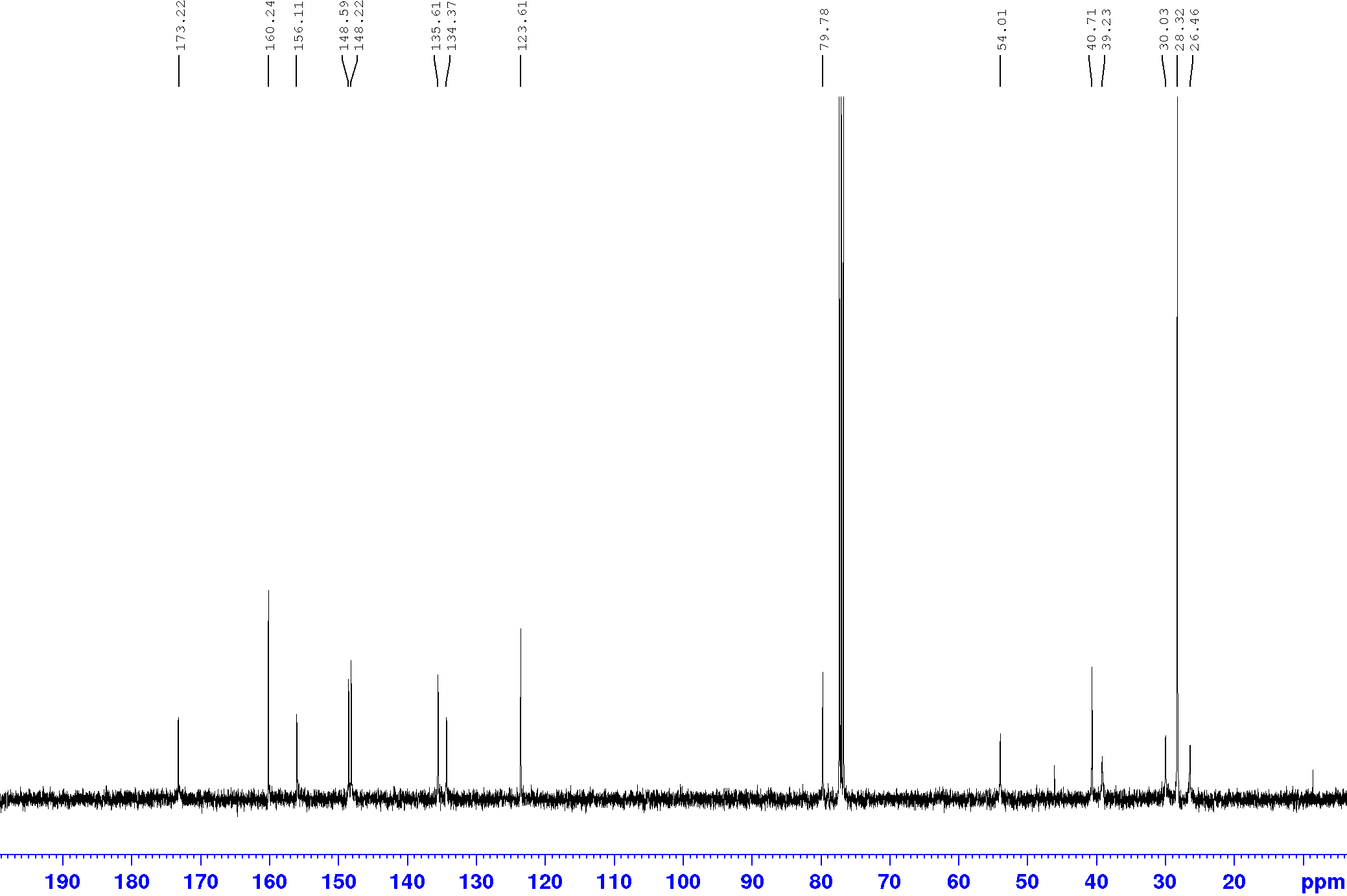


**Compound 21b.** ^1^H NMR, D_2_O, 400 MHz


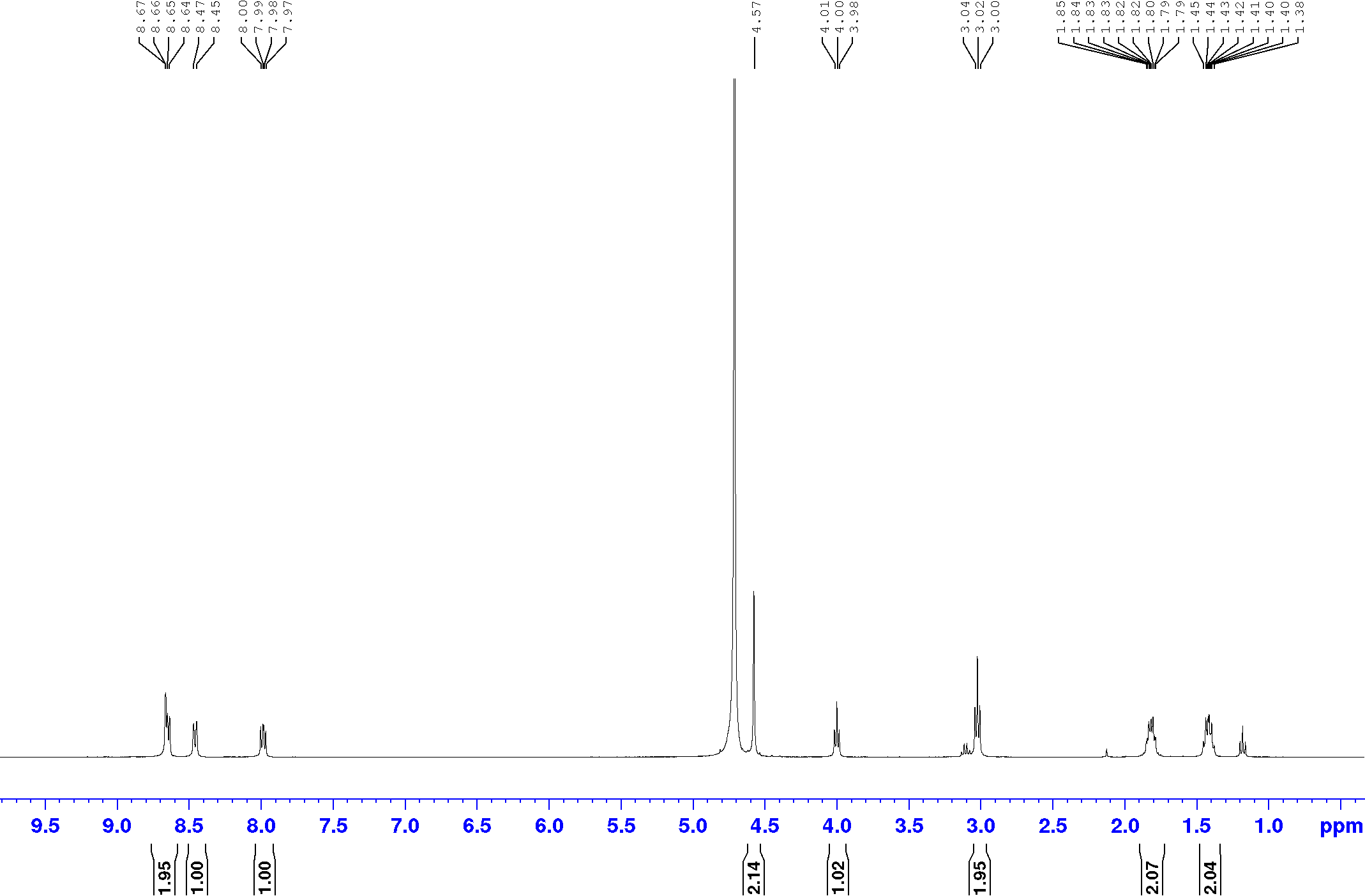


**Compound 21b.** ^13^C NMR, D_2_O, 100 MHz


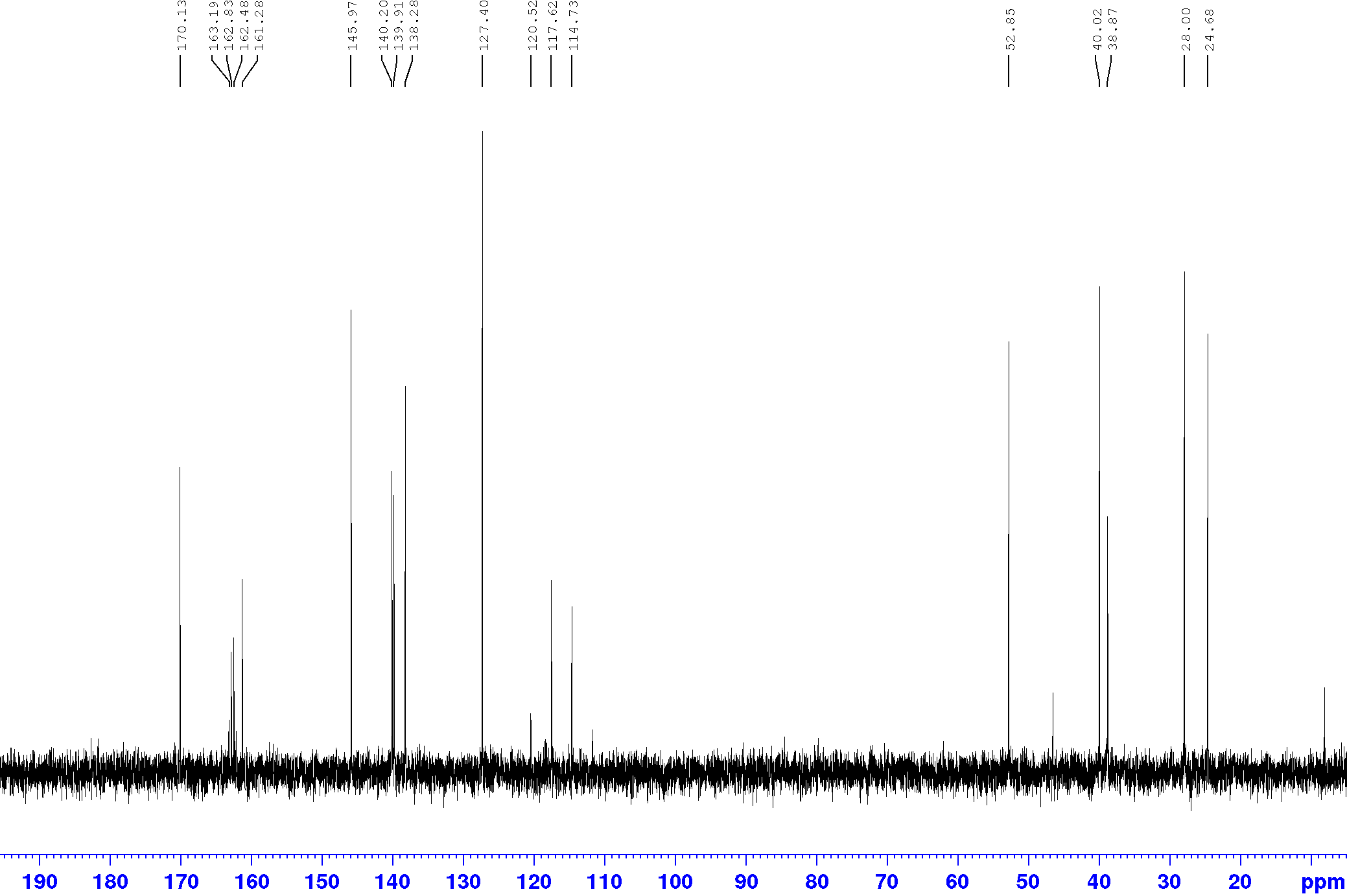


**Compound 23.** ^1^H NMR, CD_3_OD, 400 MHz


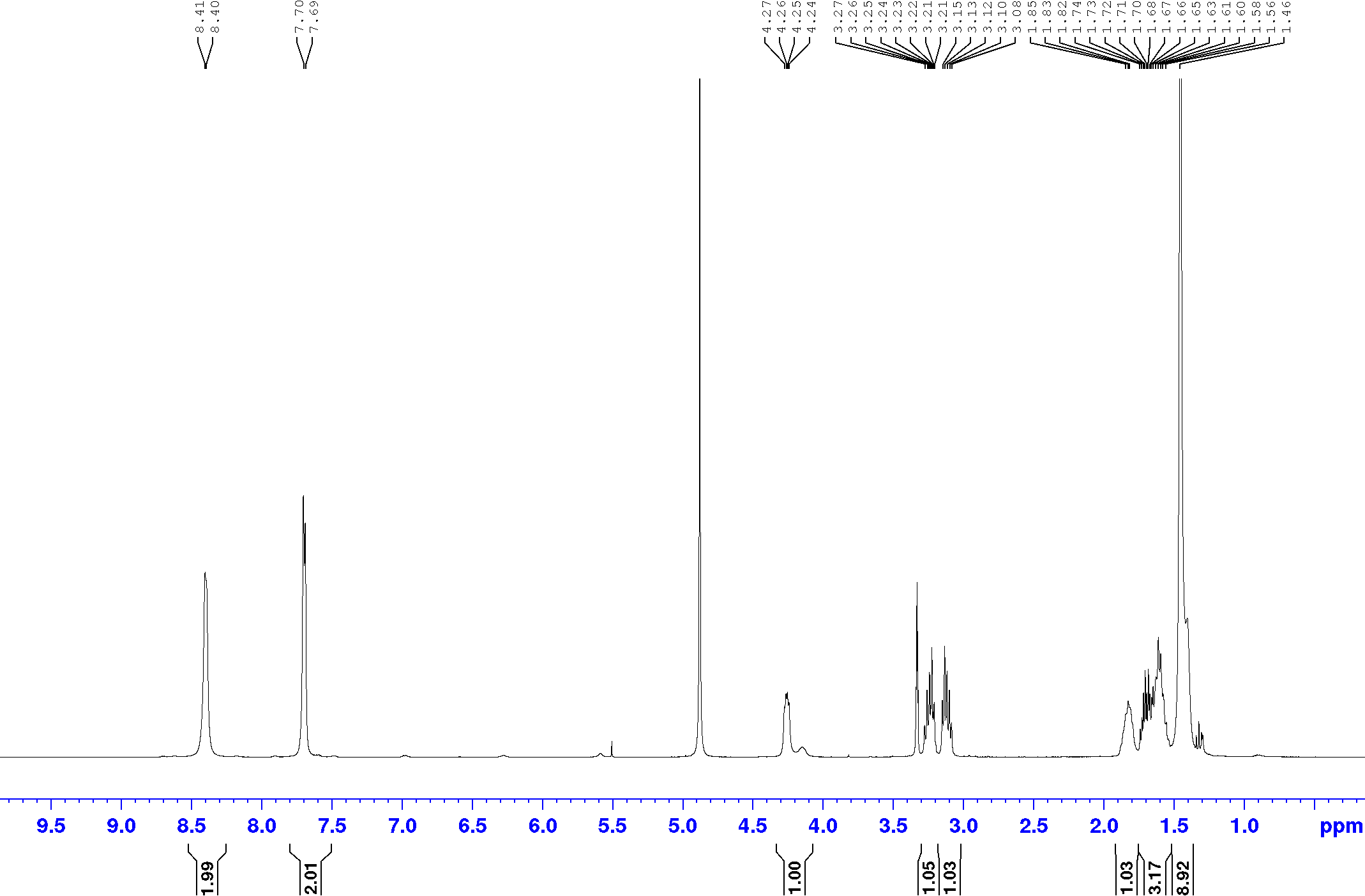


**Compound 23.** ^13^C NMR, CD_3_OD, 100 MHz


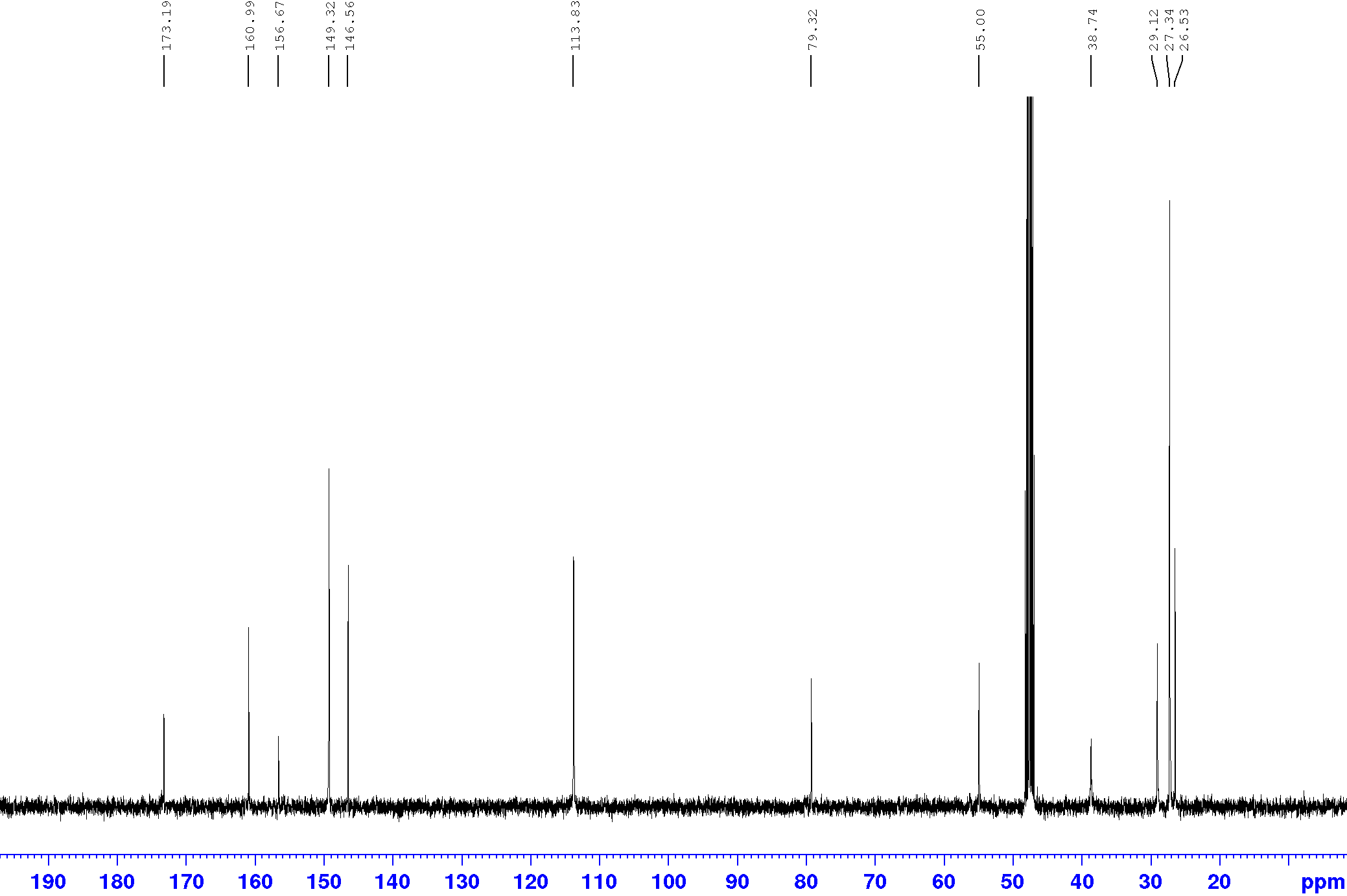


**Compound 21c.** ^1^H NMR, D_2_O, 400 MHz


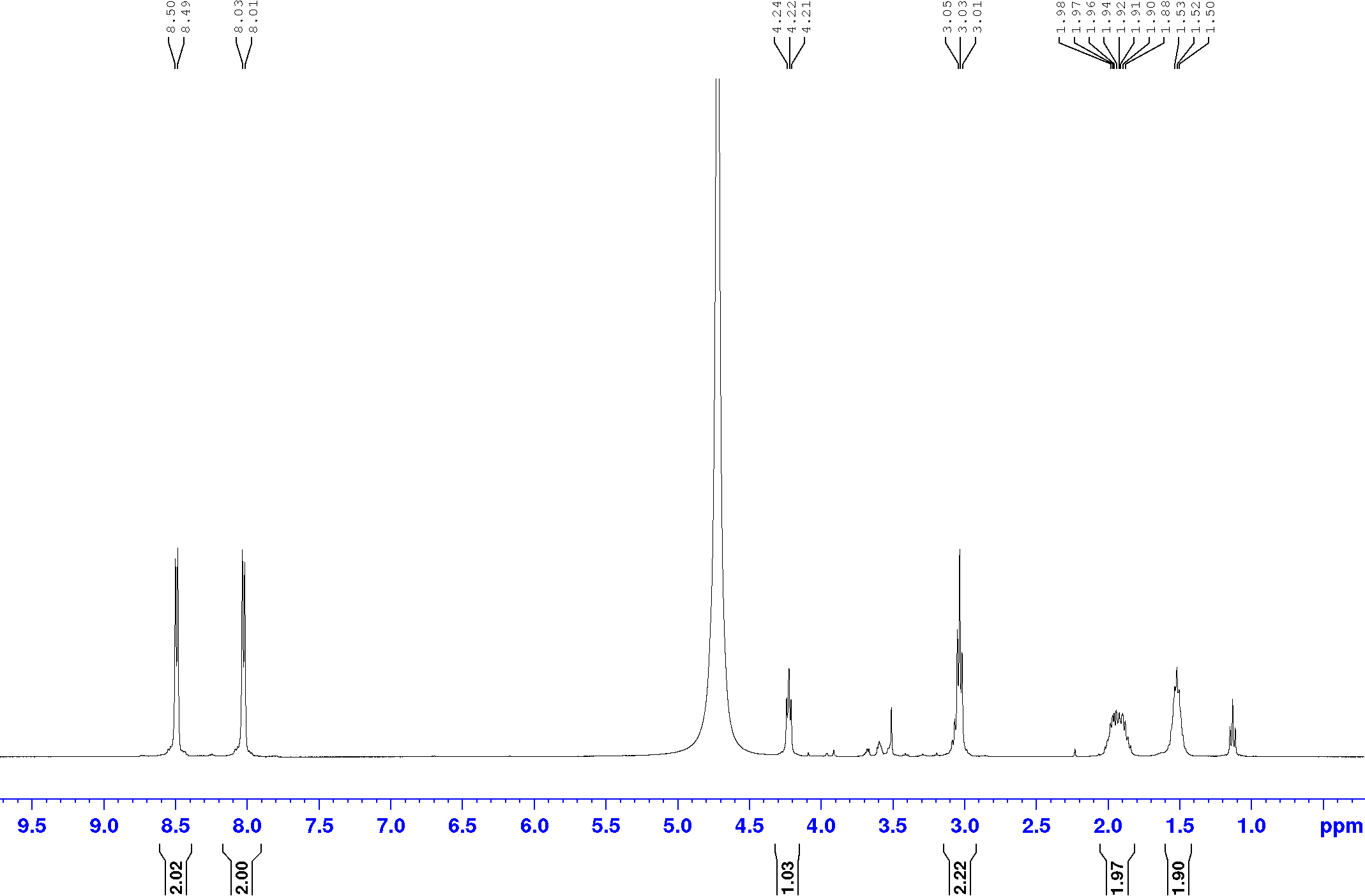


**Compound 21c.** ^13^C NMR, D_2_O, 100 MHz


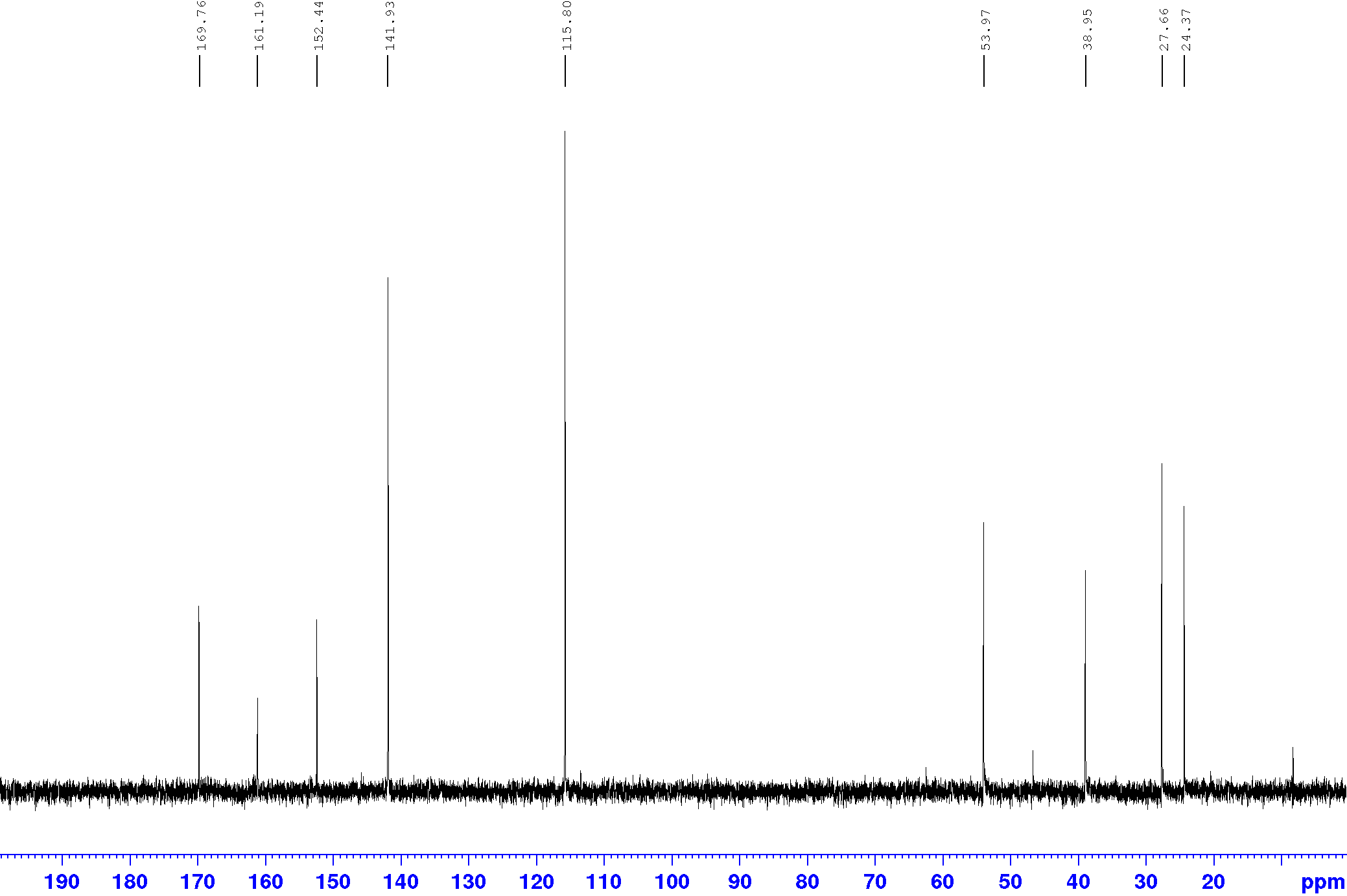


**Compound 4a.** Hyperpolarised ^1^H NMR, CD_3_OD, 400 MHz, polarisation transfer 65 G, without acetonitrile-d_3_


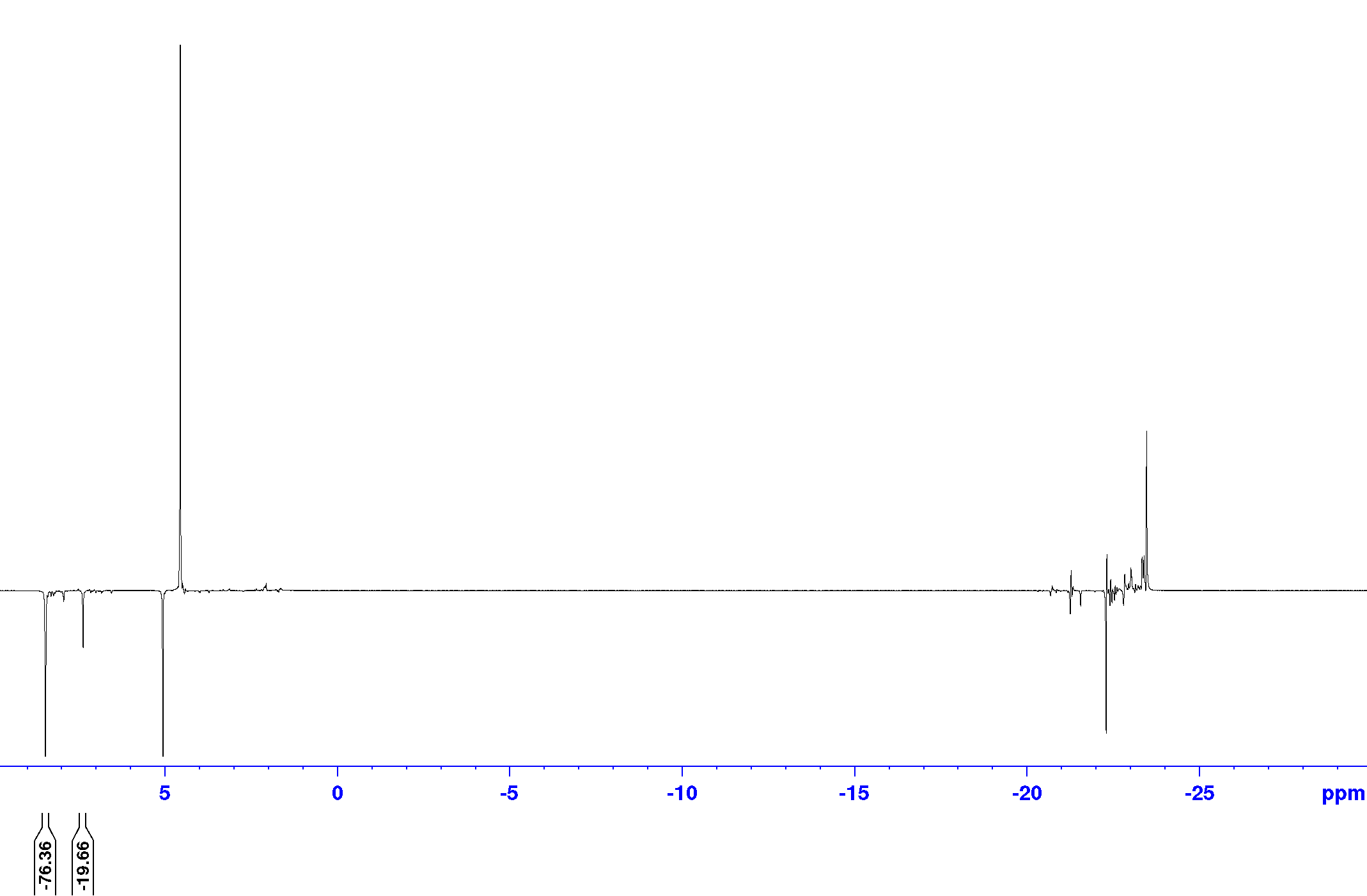


**Compound 4a.** Hyperpolarised ^1^H NMR, CD_3_OD, 400 MHz, polarisation transfer 0 G, without acetonitrile-d_3_


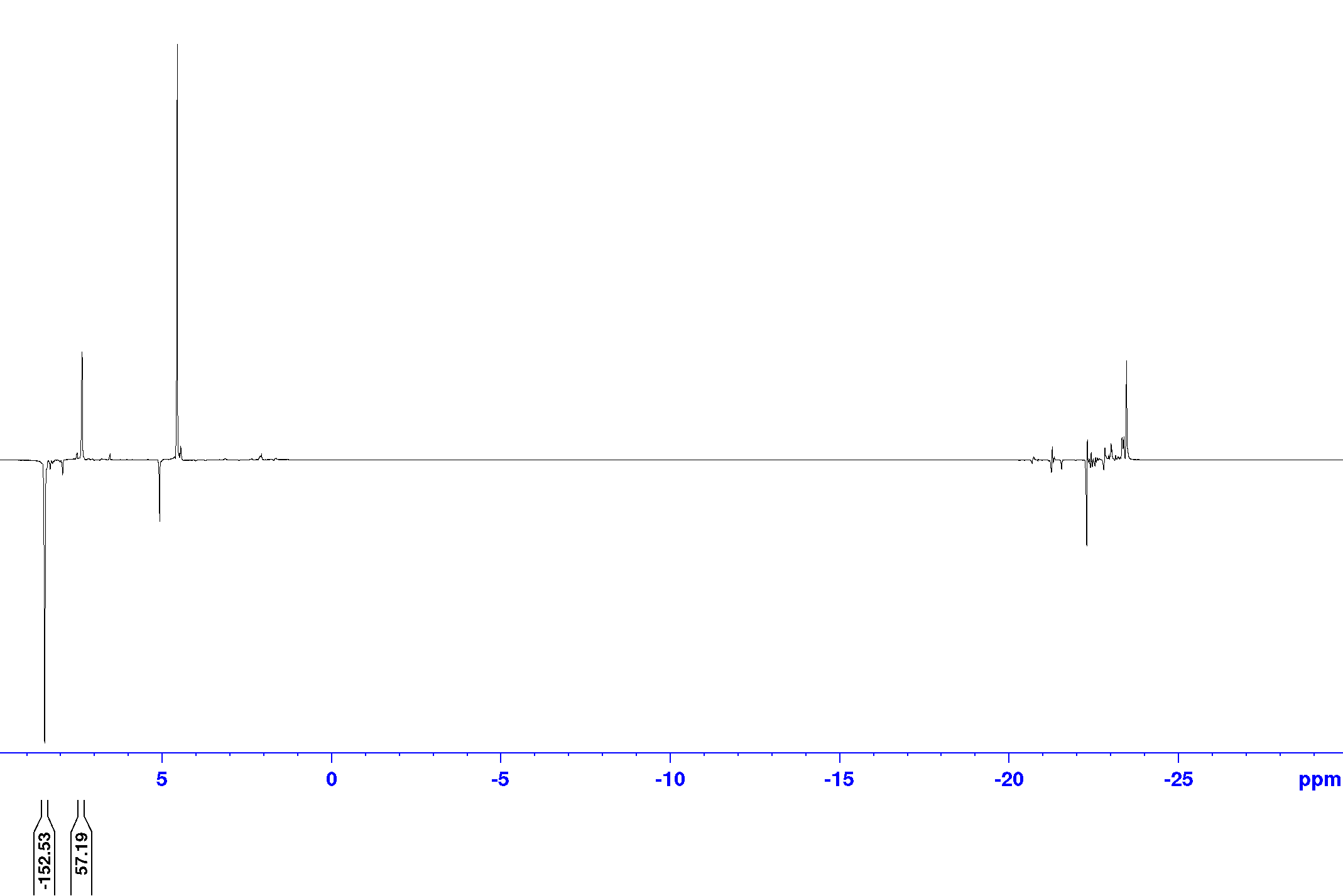


**Compound 4a.** Hyperpolarised ^1^H NMR, CD_3_OD, 400 MHz, polarisation transfer 65 G, with acetonitrile-d_3_


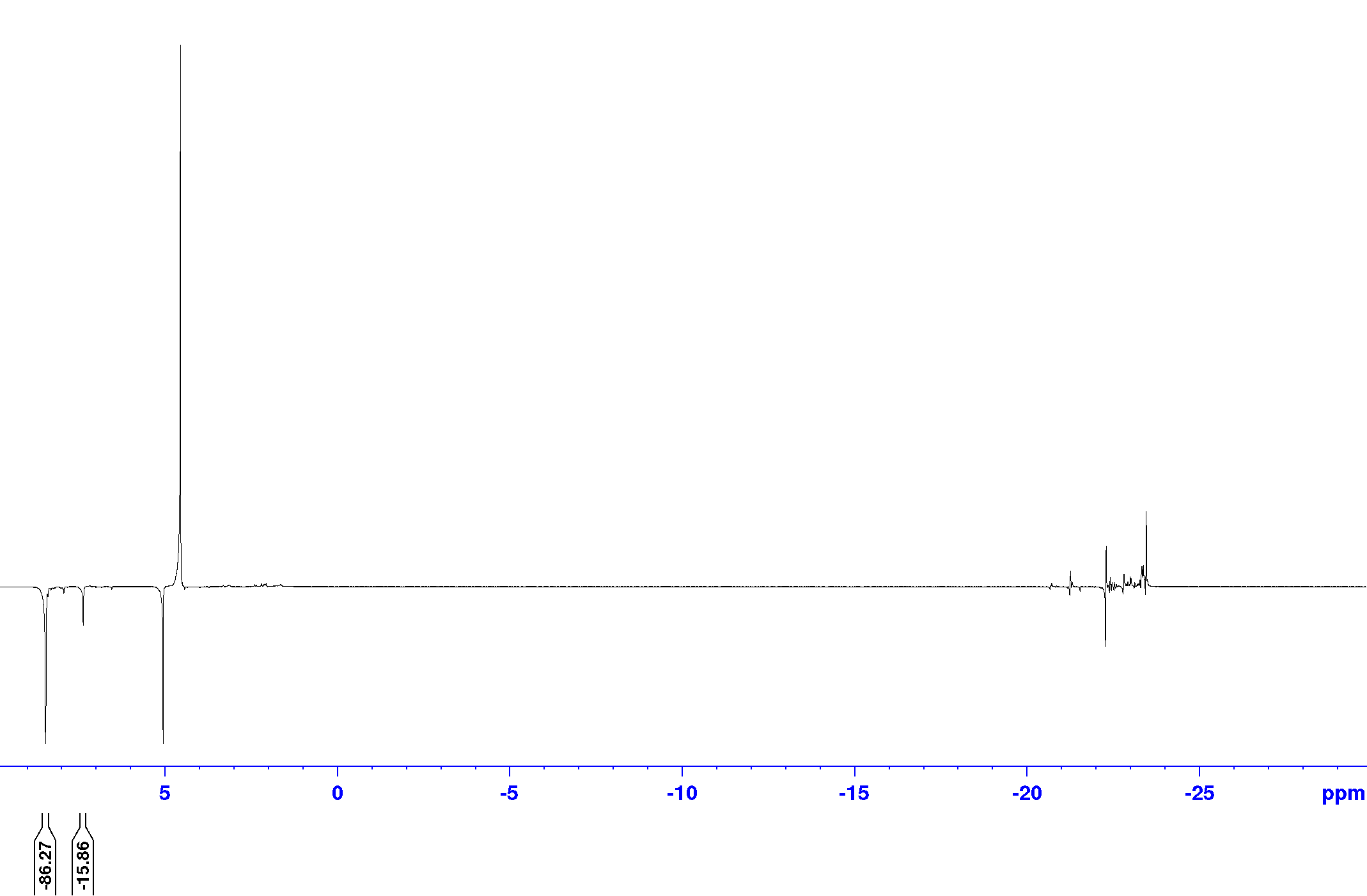


**Compound 4a.** Hyperpolarised ^1^H NMR, CD_3_OD, 400 MHz, polarisation transfer 0 G, with acetonitrile-d_3_


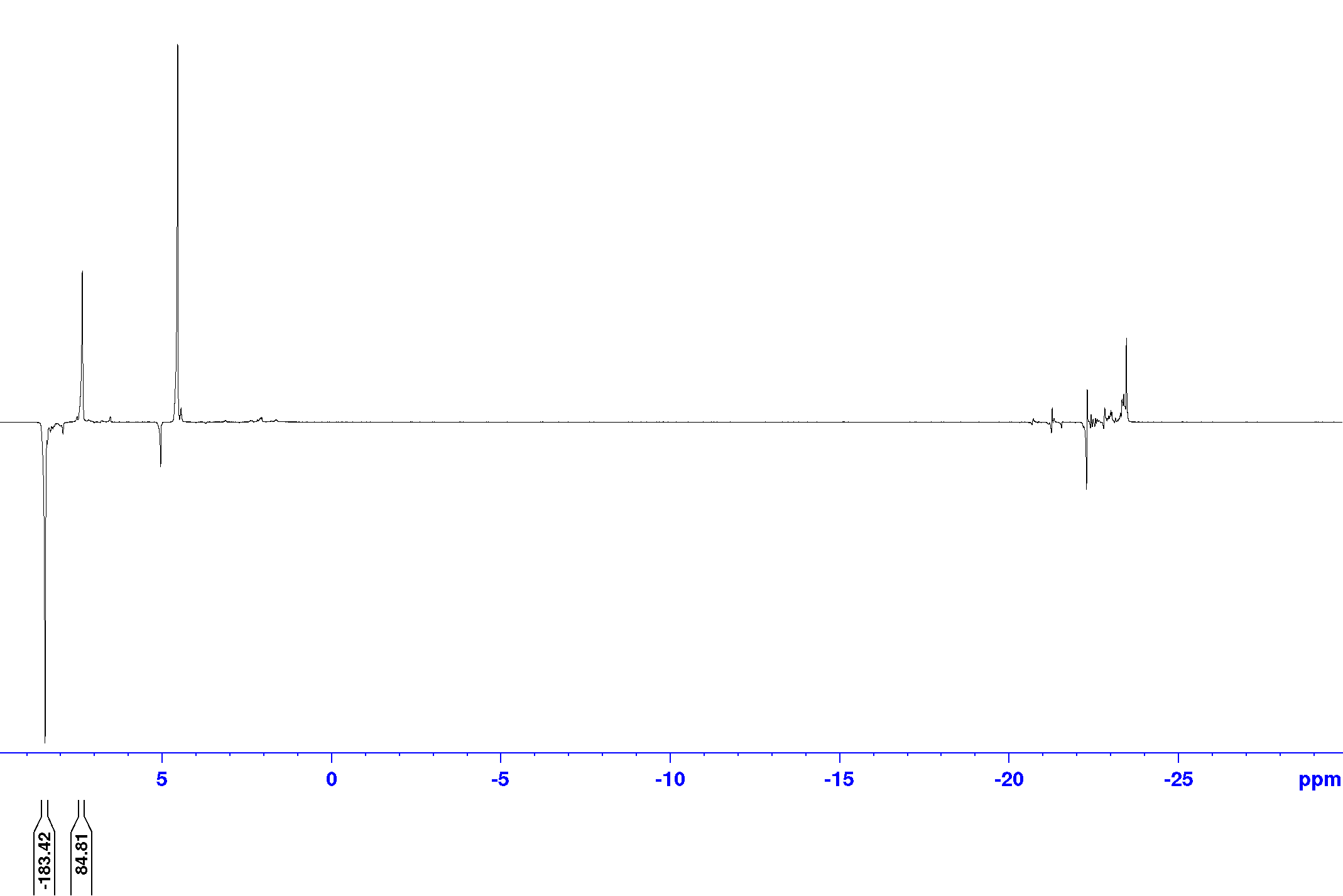


**Compound 4e.** Hyperpolarised ^1^H NMR, CD_3_OD, 400 MHz, polarisation transfer 65 G, without acetonitrile-d_3_


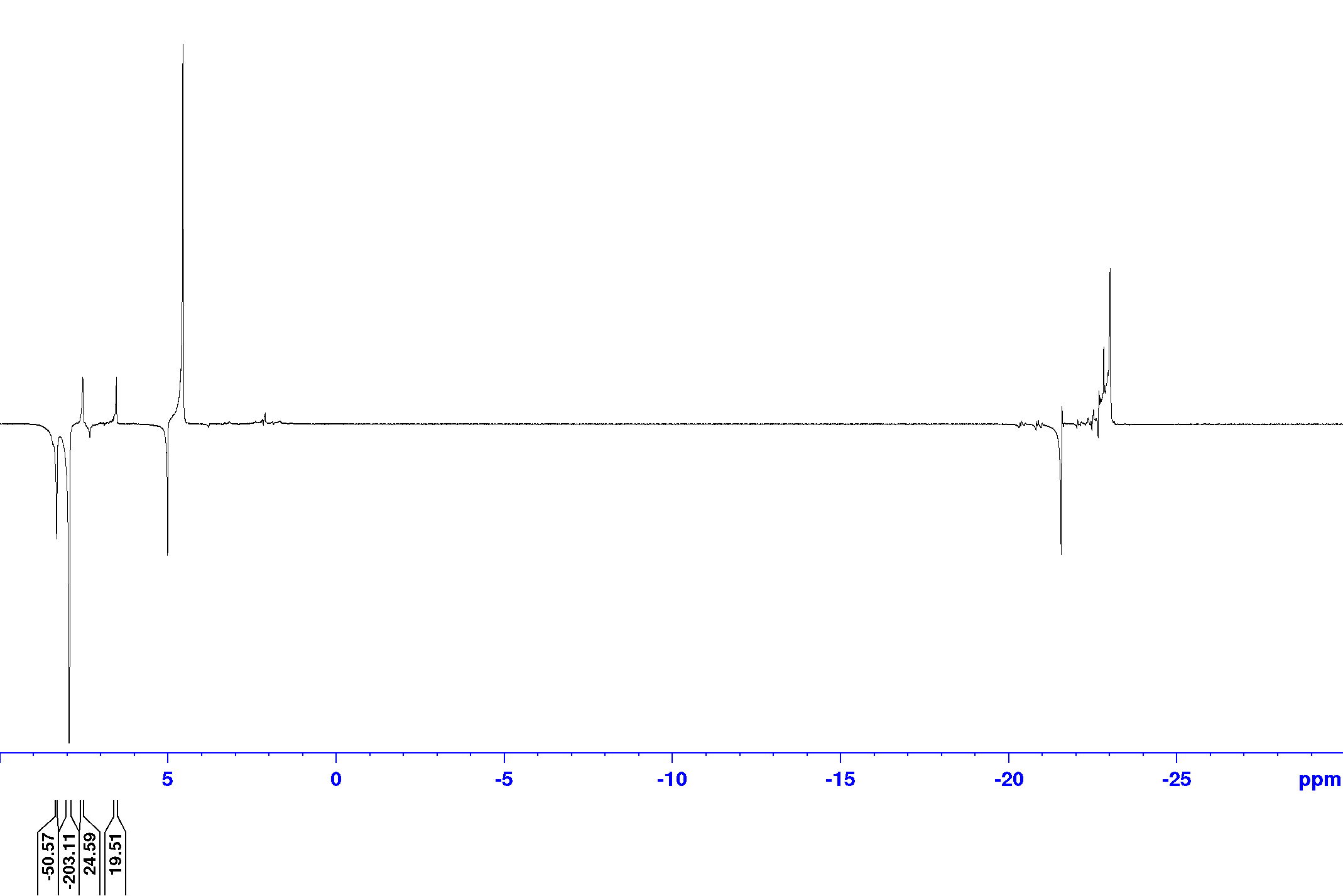


**Compound 4e.** Hyperpolarised ^1^H NMR, CD_3_OD, 400 MHz, polarisation transfer 0 G, without acetonitrile-d_3_


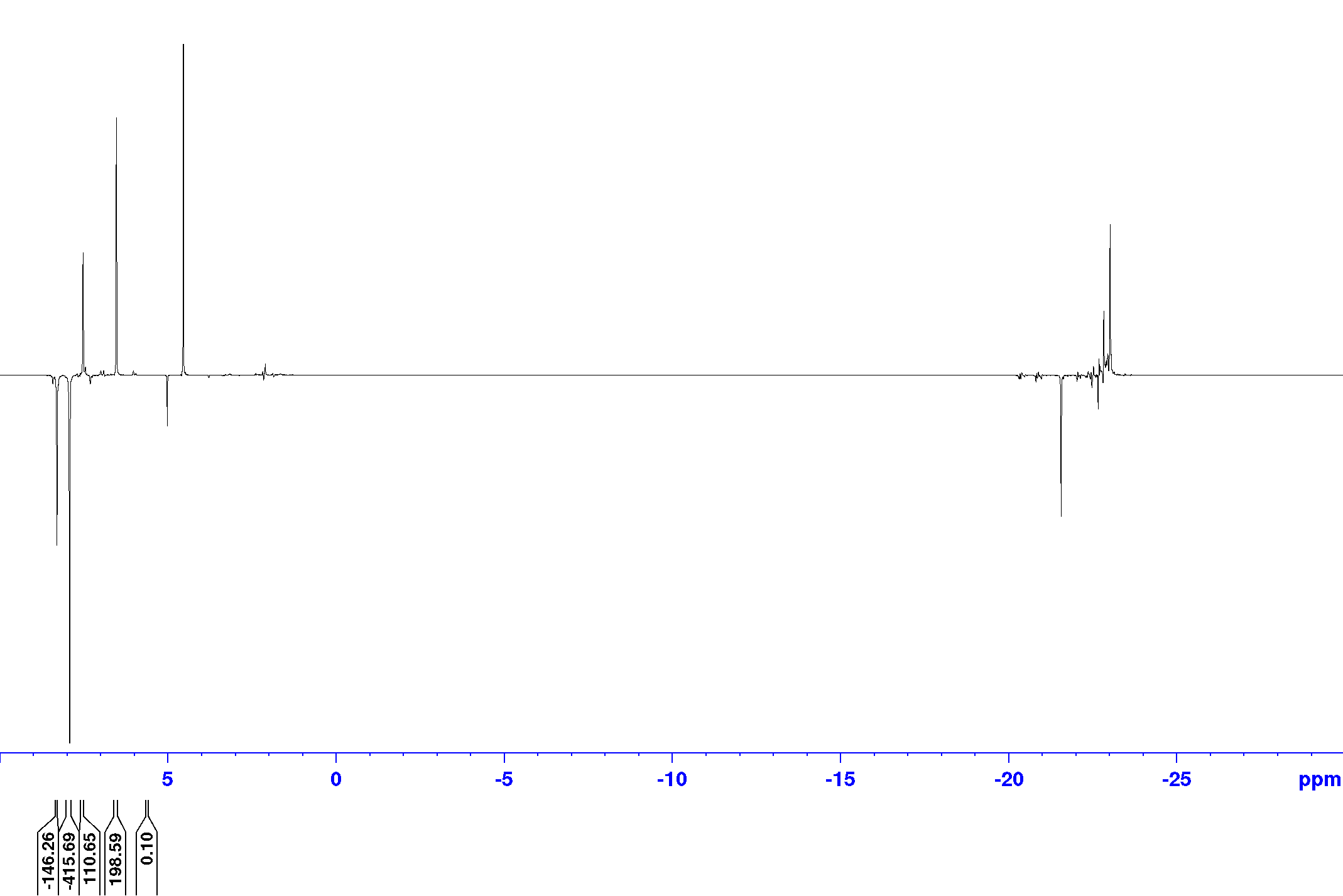


**Compound 4e.** Hyperpolarised ^1^H NMR, CD_3_OD, 400 MHz, polarisation transfer 65 G, with acetonitrile-d_3_


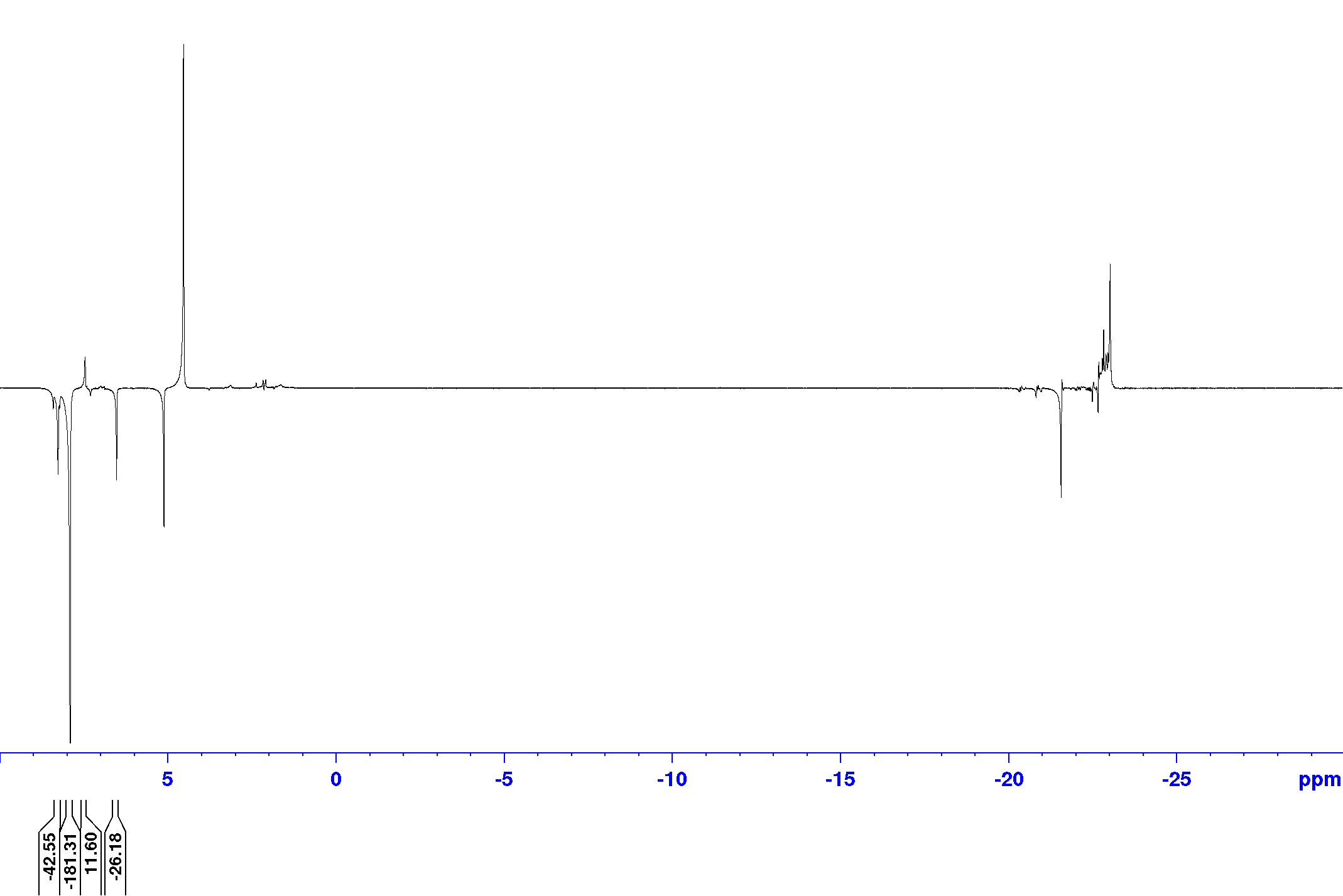


**Compound 4e.** Hyperpolarised ^1^H NMR, CD_3_OD, 400 MHz, polarisation transfer 0 G, with acetonitrile-d_3_


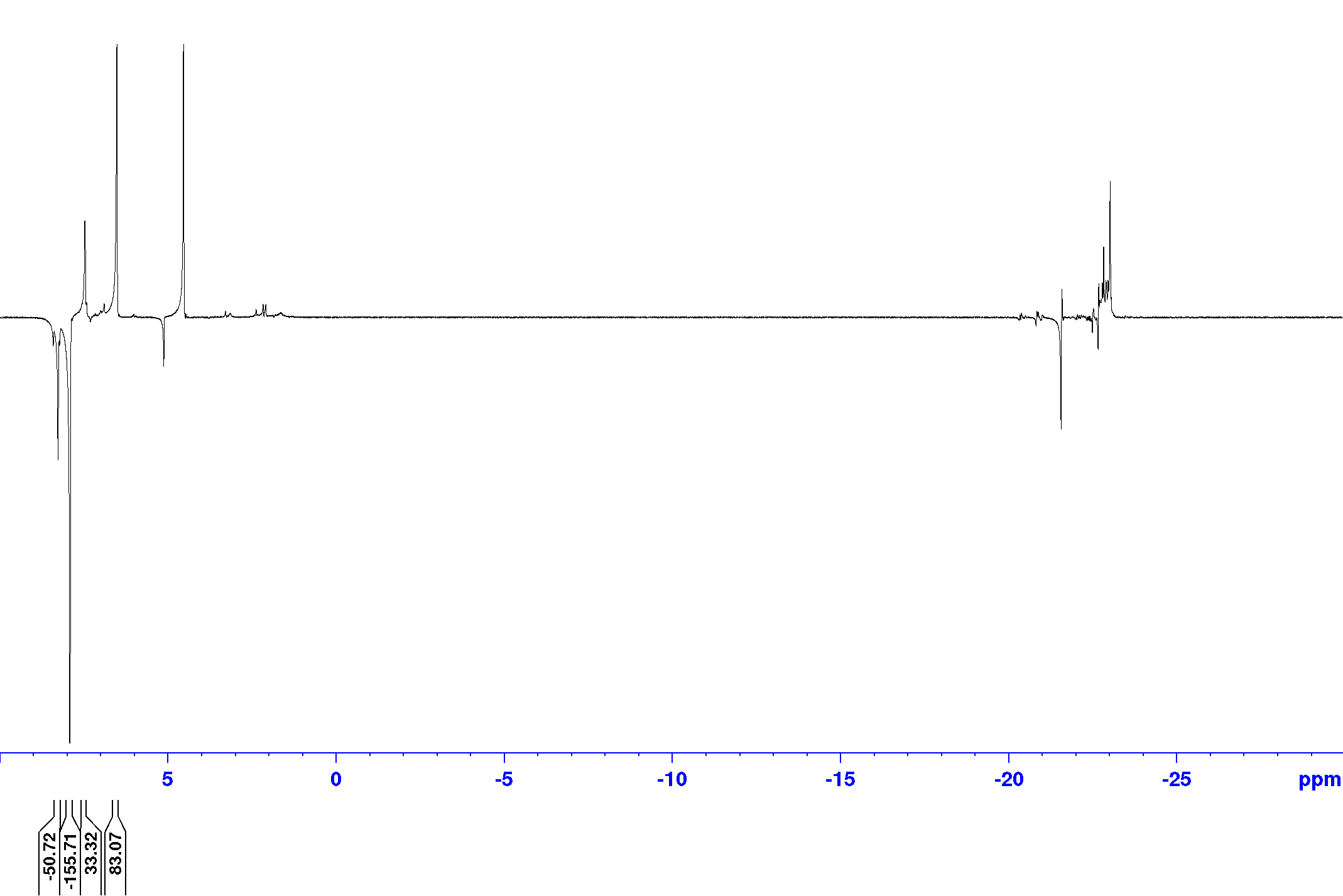


**Compound 4f.** Hyperpolarised ^1^H NMR, CD_3_OD, 400 MHz, polarisation transfer 65 G, without acetonitrile-d_3_


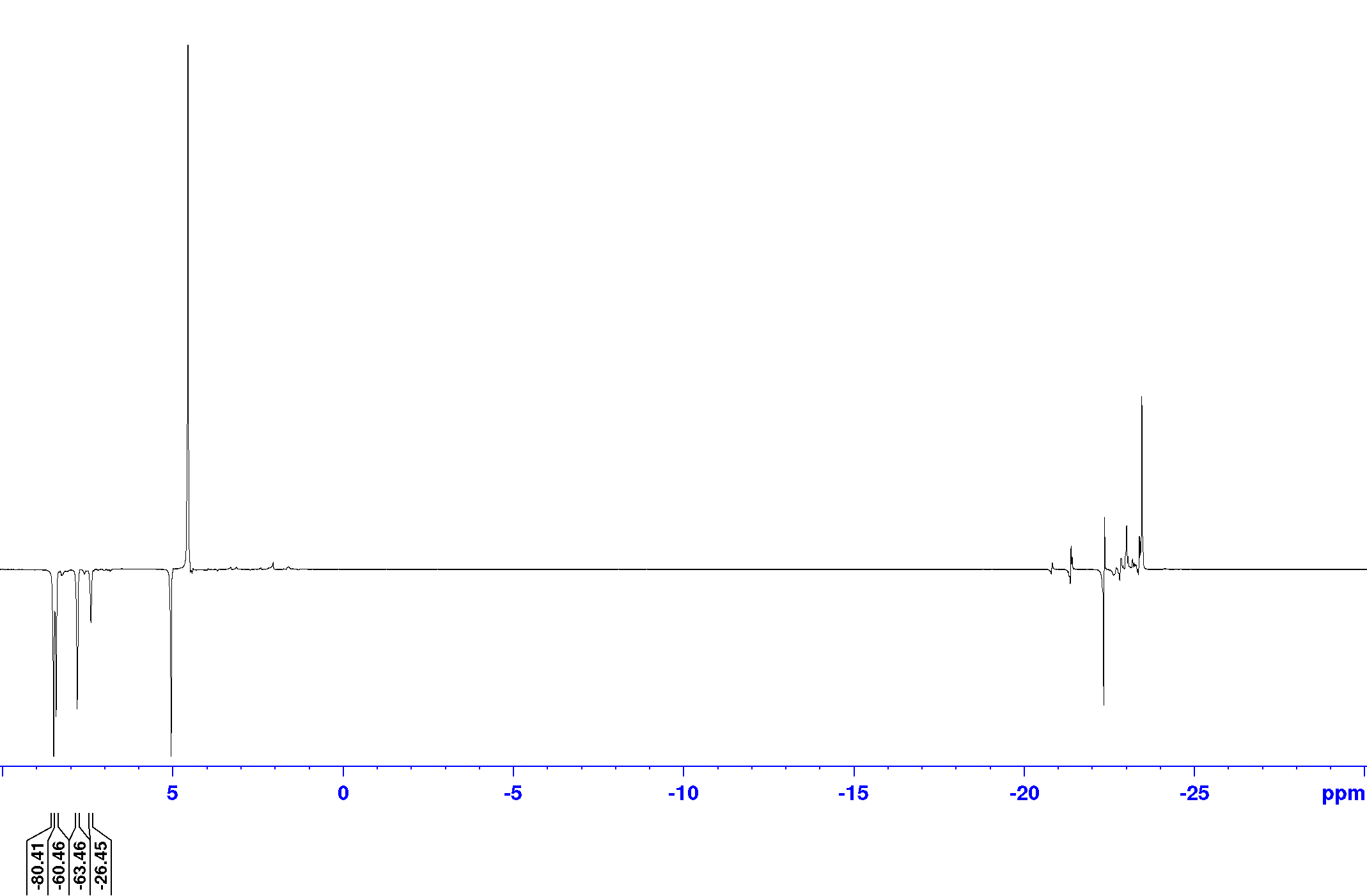


**Compound 4f.** Hyperpolarised ^1^H NMR, CD_3_OD, 400 MHz, polarisation transfer 0 G, without acetonitrile-d_3_


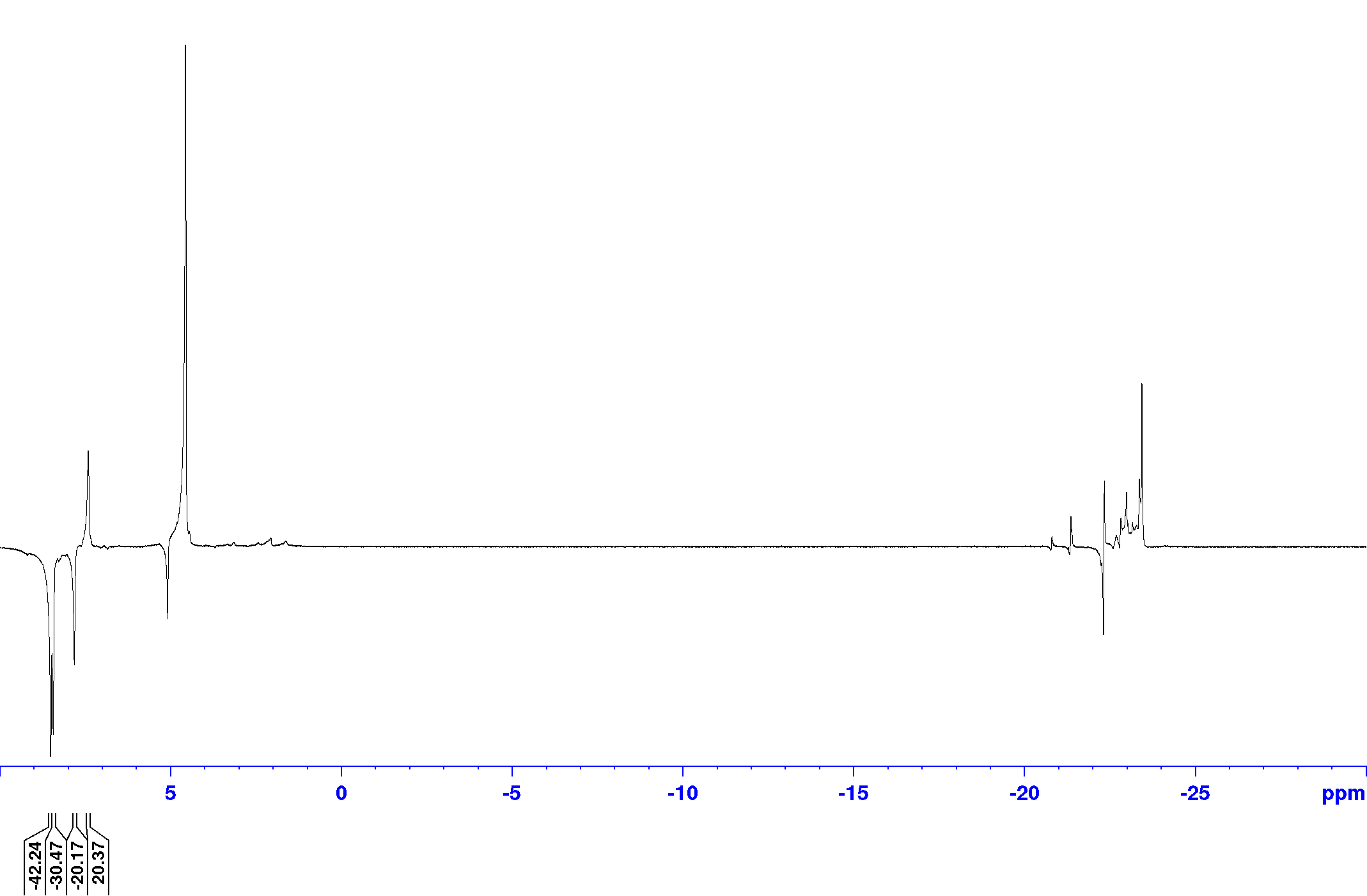


**Compound 4f.** Hyperpolarised ^1^H NMR, CD_3_OD, 400 MHz, polarisation transfer 65 G, with acetonitrile-d_3_


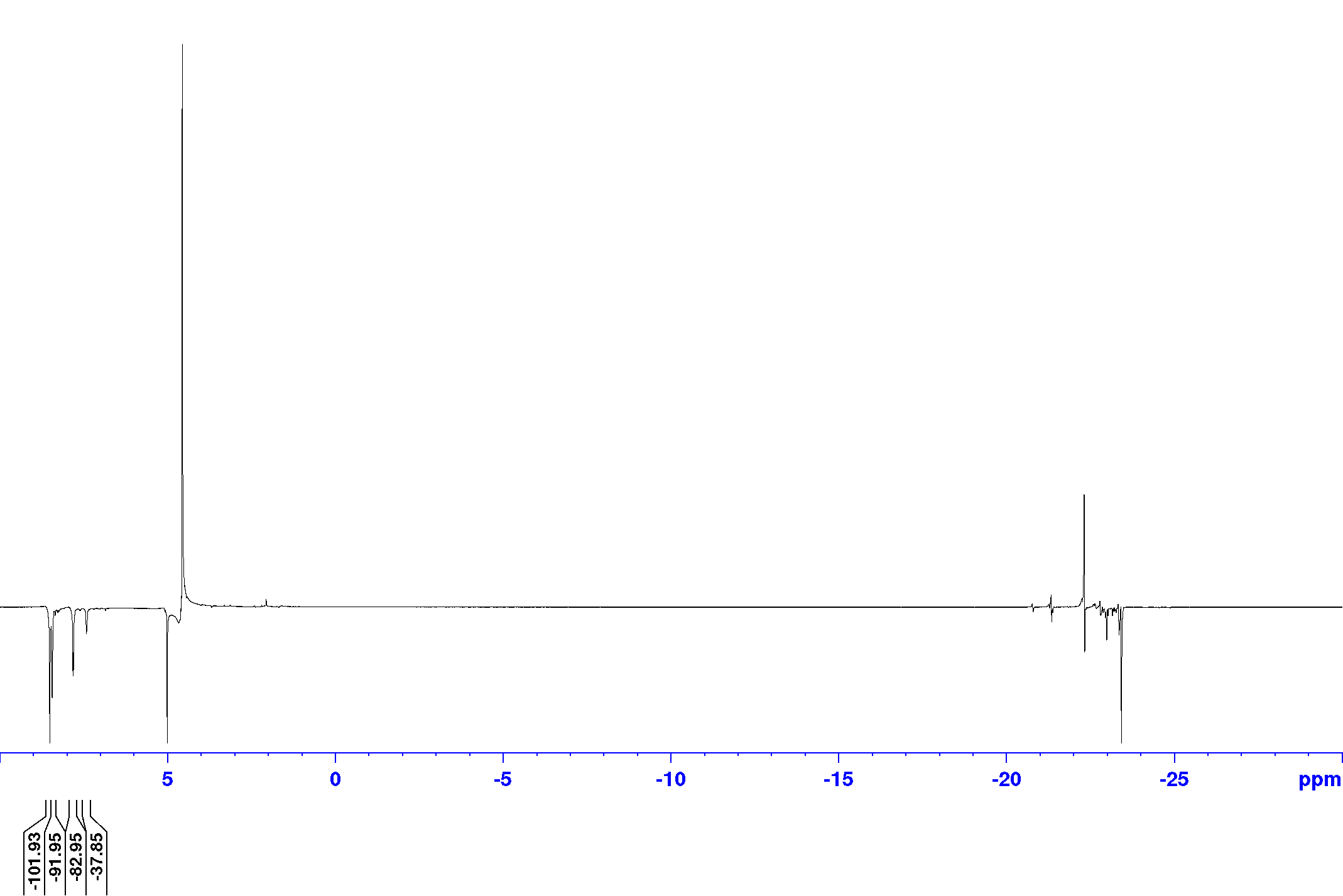


**Compound 4f.** Hyperpolarised ^1^H NMR, CD_3_OD, 400 MHz, polarisation transfer 0 G, with acetonitrile-d_3_


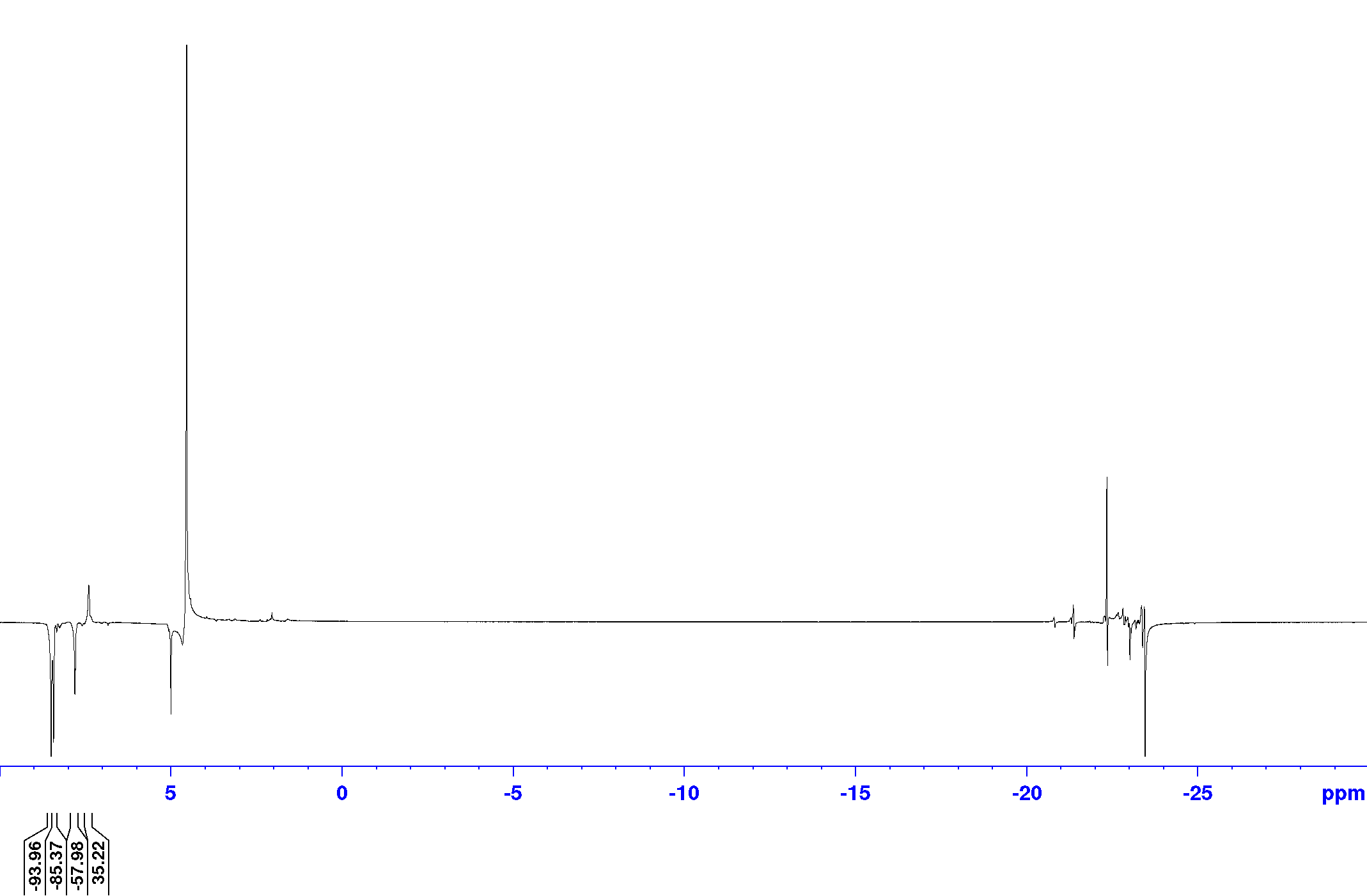


**References**

1 L. D. Vazquez-Serrano, B. T. Owens and J. M. Buriak, *Inorganica Chim. Acta*, 2006, **359**, 2786–2797.
